# Oligocene stratigraphy across the Eocene and Miocene boundaries in the Valley of Lakes (Mongolia)

**DOI:** 10.1007/s12549-016-0257-9

**Published:** 2017-03-07

**Authors:** Gudrun Daxner-Höck, Demchig Badamgarav, Rinchen Barsbold, Baatarjav Bayarmaa, Margarita Erbajeva, Ursula Bettina Göhlich, Mathias Harzhauser, Eva Höck, Volker Höck, Niiden Ichinnorov, Yondon Khand, Paloma López-Guerrero, Olivier Maridet, Thomas Neubauer, Adriana Oliver, Werner Piller, Khishigjav Tsogtbaatar, Reinhard Ziegler

**Affiliations:** 1 Natural History Museum Vienna, Burgring 7, 1010 Vienna, Austria; 2 Institute of Paleontology and Geology, Mongolian Academy of Sciences, S. Danzan street—3/1, Ulaanbaatar, 15160 P.O.B. 46/650, Mongolia; 3 Geological Institute, Siberian Branch, Russian Academy of Sciences, Ulan-Ude; Sahianova Str., 6a, 670047 Ulan-Ude, Russia; 4 Häusla 35, 8341 Paldau, Austria; 5 Department of Geography and Geology, University Salzburg, Hellbrunnerstr. 34, 5020 Salzburg, Austria; 6 Departamento de Paleontología, Facultad de Ciencias Geológicas, Universidad Complutense de Madrid, C/ José Antonio Novais, 2, 28040 Madrid, Spain; 7 Jurassica Museum, Fontenais 21, 2900 Porrentruy, Switzerland; 8 Paleobiology Department, Museo Nacional de Ciencias Naturales—CSIC, C/ José Gutiérrez Abascal, 2, 28006 Madrid, Spain; 9 Institute of Earth Sciences, Graz University, Heinrichstraße 26, 8010 Graz, Austria; 10 Staatliches Museum für Naturkunde Stuttgart, Rosensteinstraße 1, 70191 Stuttgart, Germany

**Keywords:** Mongolia, Oligocene, Miocene, Correlation, Stratigraphy, Mammals

## Abstract

Cenozoic sediments of the Taatsiin Gol and TaatsiinTsagaan Nuur area are rich in fossils that provide unique evidence of mammal evolution in Mongolia. The strata are intercalated with basalt flows. ^40^Ar/^39^Ar data of the basalts frame the time of sediment deposition and mammal evolution and enable a composite age chronology for the studied area. We investigated 20 geological sections and 6 fossil localities of Oligocene and early Miocene deposits from this region. Seventy fossil beds yielded more than 19,000 mammal fossils. This huge collection encompasses 175 mammal species: 50% Rodentia, 13% Eulipotyphla and Didelphomorphia, and 12% Lagomorpha. The remaining 25% of species are distributed among herbivorous and carnivorous large mammals. The representation of lower vertebrates and gastropods is comparatively poor. Several hundred SEM images illustrate the diversity of Marsupialia, Eulipotyphla, and Rodentia dentition and give insight into small mammal evolution in Mongolia during the Oligocene and early Miocene. This dataset, the radiometric ages of basalt I (∼31.5 Ma) and basalt II (∼27 Ma), and the magnetostratigraphic data provide ages of mammal assemblages and time ranges of the Mongolian biozones: letter zone A ranges from ∼33 to ∼31.5 Ma, letter zone B from ∼31.5 to ∼28 Ma, letter zone C from ∼28 to 25.6 Ma, letter zone C1 from 25.6 to 24 Ma, letter zone C1-D from 24 to ∼23 Ma, and letter zone D from ∼23 to ∼21 Ma.

**Electronic supplementary material:**

The online version of this article (doi:10.1007/s12549-016-0257-9) contains supplementary material, which is available to authorized users.

## Introduction

**Fig. 1:**
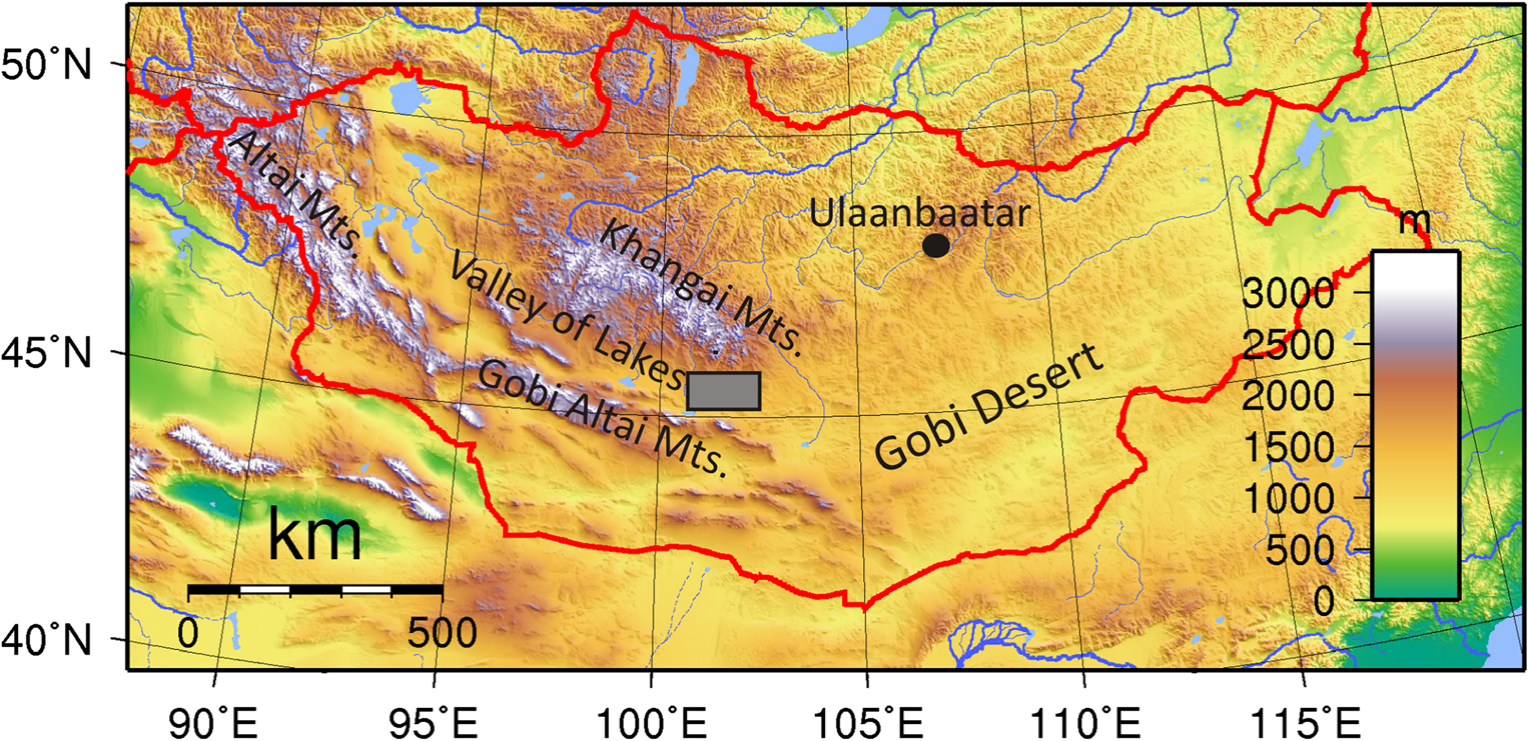
Location of the working area in the Taatsiin Gol and Taatsiin Tsagaan Nuur region, which is part of the Valley of Lakes in Central Mongolia. “Taatsiin Gol” means River Taatsiin (gol = river), but also the area around the river. “Tsagaan Nuur” means White Lake (tsagaan = white, nuur = lake)

The Valley of Lakes is an intermontane depression with a NW–SE longitudinal axis. It is bounded by the Khangai Mountains in the north and the Gobi Altai Mountains in the south. Our working area, the Taatsiin Gol region and Taatsiin Tsagaan Nuur region, ranging from 100° 55′ to 102° 05′ longitude and 45° 11′ to 45° 45′ latitude, is part of the Valley of Lakes (Fig. 1).

This region has been intensively explored during the past two decades. In a collaboration between the Mongolian Academy of Sciences and the Natural History Museum Vienna, fieldwork was conducted during eight field seasons between 1995 and 2012.

The present study focuses on the stratigraphically lower part, the Oligocene and lower Miocene sediment sequences of the Hsanda Gol and Loh Formations. Here, 20 geological sections and 6 fossil sites are described and illustrated for the first time. Along these sections, fossils were collected from 70 fossil horizons. They contain the richest small mammal assemblages ever found in Mongolia and outline Cenozoic mammalian evolution (Daxner-Höck 2000, 2001; Daxner-Höck and Wu 2003; Erbajeva 2007; Schmidt-Kittler et al. 2007; Ziegler et al. 2007; Erbajeva 2013; Erbajeva and Daxner-Höck 2014; Wessels et al. 2014; Daxner-Höck et al. 2014, 2015; Maridet et al. 2014a, b, 2015; Erbajeva et al. 2017, this issue; López-Guerrero et al. 2017a, b, this issue; Maridet et al. 2017, this issue; Oliver et al. 2017, this issue; Harzhauser et al. 2016). In contrast, the record of large mammals (Vislobokova and Daxner-Höck 2002; Morlo and Nagel 2002, 2006, 2007; Nagel and Morlo 2003; Heissig 2007), lower vertebrates (Böhme 2007), and gastropods (Stworzewicz 2007; Neubauer et al. 2013) is comparatively scarce. It has to be noted that fossils which were collected before from the studied area (by American-Mongolian, Soviet-Mongolian, and Polish-Mongolian expeditions) are not included in the present dataset because their precise stratigraphic position remains questionable. Consequently, descriptions of these fossils were not considered in the present study.

The Cenozoic strata are intercalated with basalt flows, and ^40^Ar/^39^Ar data provide a timeframe for sediment deposition and the included fossils. Thus, basalt ages and Mongolian letter zones yield a composite age chronology for the studied area (Daxner-Höck et al. 1997; Höck et al. 1999; Daxner-Höck et al. 2010; Daxner-Höck and Badamgarav 2007; Harzhauser et al. 2017, this issue). Additional basalt data were provided by Devjatkin et al. (2002). Finally, magnetostratigraphic measurements (Kraatz and Geisler 2010; Sun and Windley 2015) were performed along the Taatsiin West plateau (sections—TGR below basalt I and TGR-C; Figs. 13, 14, and 15) and in Tatal Gol (see Kraatz and Geisler 2010).

## Materials and methods

Fieldwork comprised geological mapping and studying geological sections based on lithology, structures, tectonics, and the fossil content. Basalt samples were dated by the ^40^Ar/^39^Ar method at the University of Vienna. Magnetic susceptibility and Gamma log measurements of sediments were carried out along five key sections, and sediment samples were taken for geochemical analyses and to determine the δ^18^O and δ^13^C patterns (Richoz et al. 2017, this issue). Along the geological sections, more than 100 palaeontological test samples and 60 bulk-samples of one to several tons of sediment were taken for wet screening in the field laboratory at the Taatsiin Gol camp. Sieves with 0.5, 2.5, and 5.0 mm mesh sizes were used.

In the field camp, the teeth, jaws, and bones were picked out from the dry residual using head lenses and field microscopes. The subsequent process of cleaning, identifying, and arranging the fossils took place at the NHMW (Natural History Museum Vienna). SEM images of small mammal teeth were taken using a Philips XL 20 scanning electron microscope at the Biocenter, University of Vienna. The fossils are stored in the collections of the NHMW and the MPC (Institute of Paleontology and Geology, Academy of Sciences of Mongolia).

## Geological setting and stratigraphy

**Fig. 2:**
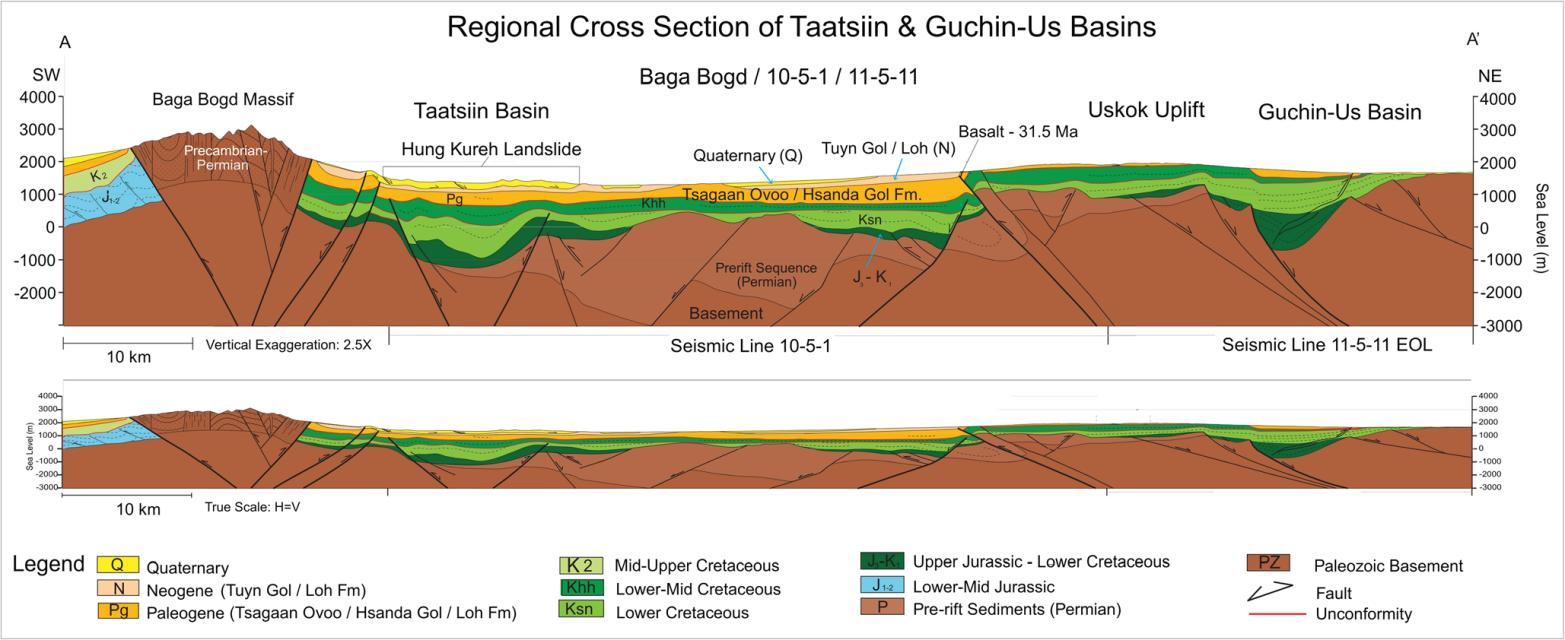
Unpublished geologic cross-section of the Taatsiin Gol and Guchin-Us basins (Mongolia) that was made available to us for publication by Justin Tully, Petro Matad LLC

The Taatsiin Tsagaan Nuur Basin belongs to the Valley of Lakes, which is one of the Pre-Altai depressions in Mongolia, between the Gobi Altai mountains in the south and the Khangai mountains in the north. Here, above a Precambrian to Permian basement, the basin is filled by continental Jurassic, Cretaceous, and Cenozoic sediments. The basin tectonics is complex and beyond the scope of this study. Note, however, that several fault systems were observed in the course of geological mapping here (Höck et al. 1999). A prominent fault close to the northern margin of the basin, the Del fault, strikes NW–SE to W–E and was mapped from the Dzun Hsir in the east along the southern escarpment of the Uskok range (= Ushgoeg range) to the northwest close to Unzing Churum. As already described by Berkey and Morris (1927), the movement along the fault is a dip-slip towards the south (southwest) with an offset of at least 20 to 30 m. The fault plane varies from south dipping to vertical. Along the Del fault, sediments of the Tsagan Ovo Fm. and the Hsanda Gol Fm. including basalt I are inclined. In contrast, horizontally bedded sediments of the Loh Fm. on top of the Eocene–Oligocene strata date the Del fault as late Oligocene or earliest Miocene. Two younger fault systems striking NE–SW and E–W are overlain by the middle Miocene basalt III (Höck et al. 1999). The recent seismic activity south of the Valley of Lakes along the northern rim of the Gobi Altai, i.e. along the Gobi Altai or Ikh Bogd fault, has a sinistral sense of movement but also a dip-slip component towards the N. There, the last major earthquake took place in 1957 with a magnitude 8/9 (Baljinyam et al. 1993; Kurushin et al. 1997; Schlupp 1996). The recent Petro Matad’s exploration program of seismic, gravity, and stratigraphic core drilling demonstrates up to 4 km of folded and faulted basin fill, Mesozoic to Paleogene episodes of extension forming a half graben, and Neogene to recent episodes of compression (Fig. 2). The latter caused the ongoing uplift of the Gobi Altai range (Bag Bogd Massif).

This area is one of the best places in Mongolia to study Paleogene and Neogene sediment-basalt associations. Four lithological units can be identified: the Tsagan Ovo, Hsanda Gol, Loh, and Tuyn Gol formations (Daxner-Höck et al. 1997; Höck et al. 1999). The rich fossil content allows an update of the letter zones A, B, C, C1, and C1-D (Oligocene) and D (lowermost Miocene). These informal letter zones were defined as Biozones according to the International Stratigraphic Guide (Harzhauser et al. 2017, this issue).

### Lithological units

#### Tsagan Ovo Fm.

The basal unit, the Tsagan Ovo Fm., is dominated by alternating grey, green-grey, whitish gravels and partly cross-bedded sand layers. The hanging parts are generally finer clastic and frequently show trough and planar cross-bedding, channel fills, and ripples. Normal graded and inverse graded beds with rip up clasts in decimetre size occur. Normal graded sandy beds can pass into fine-grained ones, which show lamination and root traces. The Tsagan Ovo Fm. was interpreted as a braided fluvial fan with a palaeocurrent direction from N to S (Höck et al.^1999^: 92–95) and partly as lake deposits. The time of deposition was late Eocene based on magnetostratigraphic correlation (Kraatz and Geisler 2010; Sun and Windley 2015).

#### Hsanda Gol Fm.

In many outcrops of the study area, the Tsagan Ovo Fm. is topped by the Hsanda Gol Fm. The latter consists of the lower Hsanda Gol beds, basalt I, and the upper Hsanda Gol beds. The term Tatal Member was introduced for the Hsanda Gol beds below basalt I and Shand Member for Hsanda Gol beds above basalt I (Dashzeveg 1996). The lower Hsanda Gol beds are of early Oligocene age, including fossils of letter zone A. The upper Hsanda Gol beds, however, range from the early Oligocene (including fossils of letter zone B) to the late Oligocene (including fossils of letter zones C and C1) or even reach the Oligocene/Miocene transition (evidenced by fossils of letter zone C1-D in section TAT-E/32; Figs. 21 and 22). The sediments are poorly sorted clay and silty clay and are reddish brown, brick red, to dark brown. Rare sand lenses or layers can be imbedded locally, e.g. in the Hsanda Gol region (SHG-A/14 and SHG-D/12; Fig. 25). Within these sediments, caliche horizons with different features are present, including compact layers, nodules, caliche grading laterally into clay layers, or occurrences of calichized basalt (Höck et al. 1999: 95–97). The Hsanda Gol beds are well known for their fossil richness. Fossil concentrations were observed in, below, or/and above caliche layers, and partly articulated skeletons were found in fossil burrows. The caliche layers are interpreted as palaeosol horizons, but the origin of the fine-grained Hsanda Gol sediments is under discussion. The interpretations range from ephemeral lake deposits, and braided fluvial fan sediments of the Tsagan Ovo Fm. that were eroded and transported by wind and/or by ephemeral streams (Höck et al. 1999), to eolian loess transported by westerly winds (Sun and Windley 2015).

#### Loh Fm.

Sediments of the Loh Fm. are most widespread in the study area. In many outcrops, the Hsanda Gol beds are covered by sediments of the Loh Fm., and in other places we found Loh sediments immediately on top of the Tsagan Ovo Fm. Loh sediments are predominantly trough cross-bedded, poorly sorted, polymict, matrix-supported gravels and sands of fluvial origin, with structures and colours similar to the Tsagan Ovo Fm. The two formations mainly differ in the gravel spectra: the Loh Fm. contains basalt, carbonate, and carbonate-tuff components due to erosion of basalts (I, II, and III) and Hsanda Gol sediments. Moreover, red to beige silty sand and sandy layers of several metres thickness can alternate with caliche and/or light-coloured sand and gravel layers (Höck et al. 1999: 97–100). These red-rose silts and caliche layers contain mammal fossils of late Oligocene to late Miocene age. The middle Miocene basalt III (13 Ma) is part of the Loh Fm.; it is frequently exposed on top of the plateaus east, north, and northwest of Taatsiin Gol.

#### Tuyn Gol Fm.

This formation crops out rarely and is restricted to the plateaus west and east of the Taatsiin Gol. The sediments are poorly sorted, grey-brown gravels of ∼9 cm diametre. Quartz components with Fe_2_O_3_ coatings, along with basalt, siltstone, granite, quartzite, gneiss, rhyolite, sandstone, and pegmatite, dominate the gravel spectrum (Höck et al. 1999: 100).

### Basalt ages

#### Basalts I

The basalts have been dated by the ^40^Ar/^39^Ar method, providing a stratigraphic framework in which the biostratigraphic data are fitted. Based on 31 dated basalt samples, three main groups of basalt occurrences were identified by Höck et al. (1999: 108–113; Fig. 18). These are the early Oligocene basalt I group around 31.5 Ma (32.2–30.4 Ma), the late Oligocene basalt II group around 28 Ma (29–27 Ma), and the middle Miocene basalt III around 13 Ma (13.2–12.2 Ma). The geochemistry and mineralogy of basalts I–III was described by Höck et al. (1999: 104–108: Table 5, Figs. 12, 13, 14, 15, 16, and 17). Since then, additional basalt ages have become available, showing that the Oligocene basalt events (basalt I and basalt II groups) occurred more or less continuously (32.4–29.1 and 28.7–24.9 Ma, respectively). The middle Miocene volcanism (basalt III group), however, started after an interval of 10 million years (14.9–12.2 Ma) (Tables 1 and 2).

**Fig. 63:**
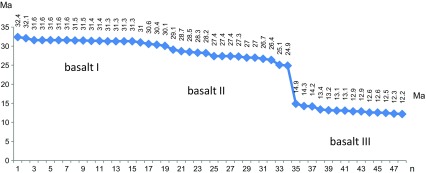
**a** ^40^Ar/^39^Ar data of basalt I–III from the Taatsiin Gol and Taatsiin Tsaagan Nuur region (Valley of Lakes, Mongolia). Basalt I (20 samples), basalt II (14 samples), basalt III (14 samples)

**Table 2:** Localization and age dating of basalt samples from the Valley of Lakes are given in columns 1–5: *1* sample codes; *2* coordinates; *3* basalt group I, II, III; *4* ^40^Ar/^39^Ar age of the basalt sample; *5* localization of the basalt sample/section or fossil locality [section ABO-A (Abzag Ovo); section TGL-A (Taatsiin Gol left side of the river); sections TGR-A, TGR-B, TGR-AB (Taatsiin Gol right side of the river), TGR-ZO/1, 2 (fossil site at Taatsiin Gol right side); section TAR-A (Unzing Churum), DEL (tilted basalt at Del); section TAT-C, TAT (Tatal Gol); section GRAB (Talyn Churum)]

Basalt sample code:	N	E	Basalt I–III	Range (Ma)	Section/pal. sample
Western region: Luuny Yas–Luugar Khudag–Abzag Ovo					
M132/97	45°34′52″	101°04′21″	II	27.0 ± 0.9	ABO-A top
M109/97	45°34′08″	101°08′19″	II	26.7 ± 0.7	
M52/97	45°33′16″	101°06′35″	II	25.1 ± 0.5	
M53/97	45°32′31″	101°06′57″	II	24.9 ± 0.5	
M44/97	45°32′24″	101°08′18″	III	12.5 ± 0.5	
M108/97	45°35′45″	101°07′54″	III	13.4 ± 0.4	
M113/97	45°37′17″	101°02′18″	III	12.3 ± 0.7	
M116/97	45°29′23″	101°01′47″	III	14.3 ± 0.4	
M131/97	45°30′48″	100°58′22″	III	12.6 ± 0.9	
M118/97	45°27′56″	100°57′13″	III	14.9 ± 0.5	
M1/96	45°24′41″	101°01′33″	III	14.2 ± 0.2	
Taatsiin region: Taatsiin Gol left and right					
M142/97	45°44′56″	101°12′28″	III	12.6 ± 0.3	
M143/97	45°44′56″	101°12′28″	III	12.2 ± 0.7	
TLA25/95	45°27′11″	101°16′39″	III	13.1 ± 0.2	TGL-A top
TLA10/95	45°26′59″	101°16′23″	I	31.6 ± 0.5	TGL-A basis
M46/96	45°27′31″	101°12′32″	I	32.1 ± 0.4	
M47/96	45°27′31″	101°12′32″	I	30.4 ± 0.7	
M68/96	45°25′27″	101°15′25″	I	29.1 ± 0.9	
TRB1/95	45°25′11″	101°15′35″	I	30.6 ± 0.6	Near TGR-A, B, AB
TRA19/95	45°25′11″	101°15′35″	I	32.4 ± 1.0	Near TGR-A, B, AB
M5/96	45°24′58″	101°15′42″	I	31.6 ± 0.6	Near TGR-A, B, AB
TRA20/95	45°24′54″	101°15′44″	I	31.0 ± 0.4	Near TGR-A, B, AB
M48/96	45°24′15″	101°15′52″	I	31.3 ± 0.5	TGR-ZO-1, 2
Unzing Churum and Del region					
DIV/95			III	12.9 ± 0.3	
DV/95	45°31′17″	101°18′12″	III	12.9 ± 0.1	TAR-A top
M17/96	45°31′22″	101°18′30″	III	13.1 ± 0.2	
M18/96	45°31′22″	101°18′30″	III	13.2 ± 0.3	
DIII2/95	45°31′13″	101°18′10″	II	28.2 ± 0.7	
M56/96	45°31′10″	101°18′09″	II	27.4 ± 0.4	TAR-A basis
M4/96	45°32′10″	101°18′15″	II	27.3 ± 0.5	
DA2/95	45°29′45″	101°17′50″	I	31.5 ± 0.4	
M4/96	45°29′39″	101°18′14″	I	31.6 ± 0.6	
M45/96	45°27′54″	101°20′28″	I	31.6 ± 0.5	
DA/95	45°27′29″	101°21′25″	I	31.4 ± 0.6	
M32/96	45°27′28″	101°21′31″	I	30.1 ± 0.7	
Tatal Gol region					
M5/97	45°22′46″	101°38′47″	II	27.4 ± 0.7	
M11/97	45°22′46″	101°38′47″	II	28.5 ± 0.8	
M28/97	45°20′13″	101°38′23″	II	26.4 ± 0.7	
M30/79	45°19′31″	101°39′53″	II	27.0 ± 0.6	
TAT3/95	45°18′21″	101°38′01″	I	31.6 ± 0.5	Close to TAT-C
TAT2/95	45°18′08″	101°37′53″	I	31.3 ± 0.5	
TAT1/95	45°17′50″	101°37′46″	I	31.4 ± 0.7	
M25/96	45°23′42″	101°34′05″	I	31.3 ± 0.5	
Eastern region: Ulan Tolgoi to Talyn Churum					
M37/97	45°28′56″	101°51′19″	II	28.3 ± 0.6	
M41/97	45°28′24″	101°52′01″	II	27.4 ± 1.1	
M17/97	45°20′03″	101°53′13″	II	28.7 ± 0.7	
UTO/95	45°20′49″	101°50′16″	I	31.3 ± 0.5	
GII/95	45°16′53″	101°57′30″	I	31.5 ± 0.7	GRAB-II top

**Fig. 3:**
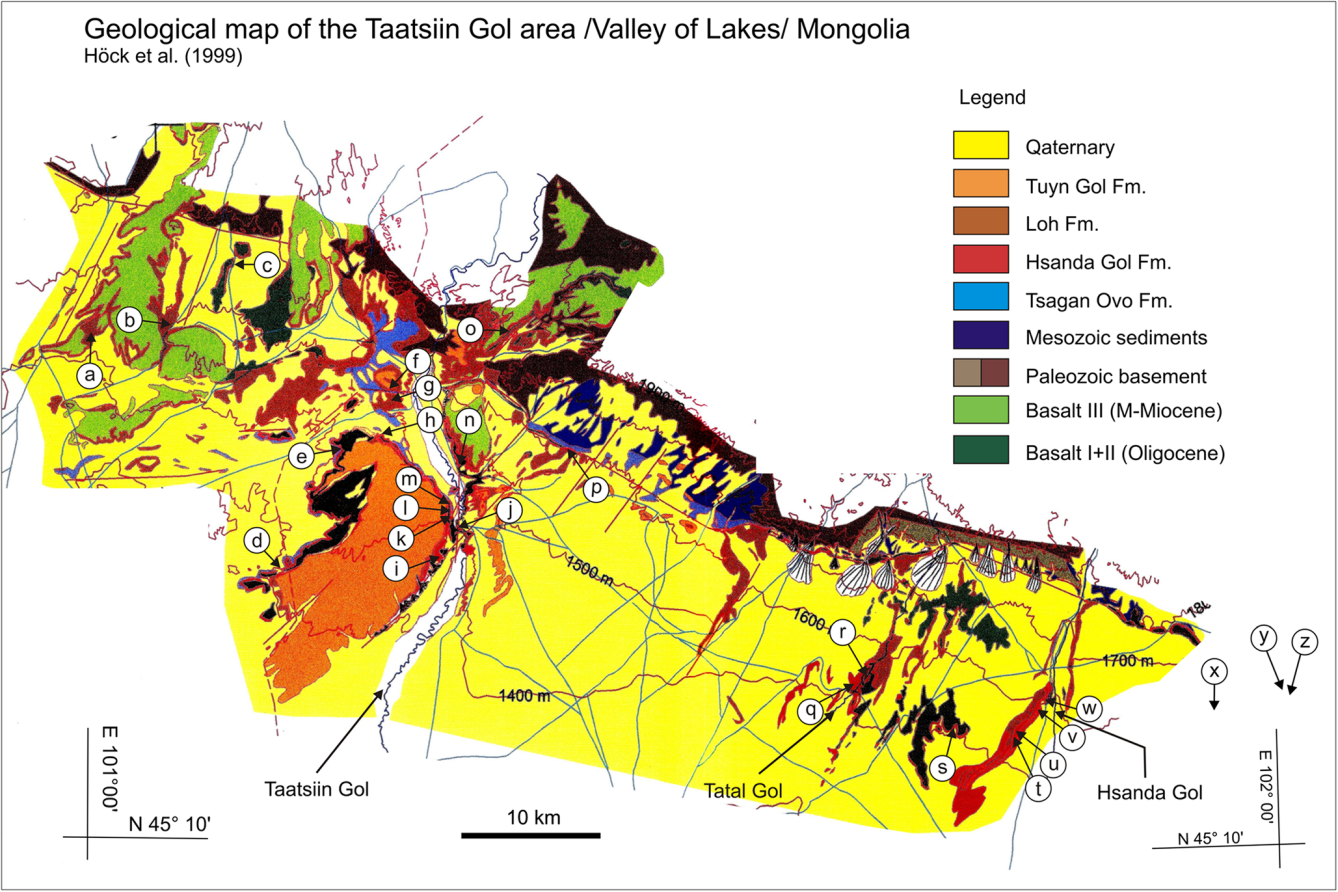
Geological map of the Taatsiin Gol and Taatsiin Tsagaan Nuur region in the Valley of Lakes (Höck et al. 1999). The letters *a*–*z* designate fossil places and investigated sections. ***a*** Luuny Yas (LUS), ***b*** Luugar Khudag (LOG-A), ***c*** Abzag Ovo (ABO-A), ***d*** Toglorhoi (TGW), ***e*** Khongil (HL), ***f*** Huch Teeg (RHN), *g* Hotuliin Teeg (HTE, HTS, HTSE), *h* Unkheltseg (UNCH-A), *i* Taatsiin Gol south (TGR-C), *j* Tsagan Ovo (TGR-ZO), *k* Taatsiin Gol right (TGR-B), *l* Taatsiin Gol right (TGR-AB), *m* Taatsiin Gol right (TGR-A), *n* Taatsiin Gol left (TGL-A), *o* Unzing Churum (TAR-A), *p* Del (DEL-B), *q* Tatal Gol (TAT-D+E), *r* Tatal Gol (TAT-C), *s* Hsanda Gol (SHG-C), *t* Hsanda Gol (SHG-A), *u* Hsanda Gol (SHG-D), *v* Loh (LOH-C), *w* Loh (LOH-B), *x* Talyn Churum (GRAB-II), *y* Ikh Argalatyn Nuruu (IKH-A), *z* Ikh Argalatyn Nuruu (IKH-B)

In contrast, the regional distribution of basalt I and II differs significantly. Basalt I occurrences are concentrated in the southern and central part of the study area. They extend from the western as far as the easternmost investigated regions. The most prominent outcrops are visible at the plateau west of Taatsiin Gol (sections TGR-A, TGR-B, TGR-AB, TGW-A, HL-A; Figs. 8, 14, and 15), the plateau east of Taatsiin Gol (section TGL-A; Fig. 16), along the Del fault (section DEL-B; Fig. 20) where the basalt I and tuff I are tilted, in Tatal Gol (section TAT-C; Fig. 23), and east of Tatal Gol (sections SHG-C and GRAB-II; Figs. 24 and 27). Basalt I is imbedded in red clay/silty clay of the Hsanda Gol Fm. In N–S direction, all basalt I occurrences are located south of basalt II. Basalt II is exposed in the northern parts of the study area in four main regions, the northwest region (section ABO-A; Fig. 6), the Unzing Churum region (section TAR-A; Figs. 18 and 19), the northern Tatal Gol region, and north of Ulan Tolgoi. Basalt II is bound to strata of the Loh Fm. For localization of sections, see Fig. 3.

Some basalt occurrences with fossil contact are chrono-stratigraphically important. In the Taatsiin Gol, Del, and Tatal Gol regions, basalt I is intercalated with strata of the Hsanda Gol Fm. Consequently, fossil beds below basalt I are older, and those above basalt I are younger than ∼31.5 Ma. In the northern regions, for example, basalt II (sample M132/97) of section ABO-A (Fig. 6) is located immediately above fossil bed ABO-A/3 and dates the fossils older than 27.9 ± 0.9 Ma. In the Unzing Churum region (Figs. 18 and 19), basalt II (sample M132/97) is located immediately below fossil bed TAR-A/2 and dates the fossils younger than 27.4 ± 0.4 Ma (Tables 1 and 2).

Basalt III is part of the Loh Fm. and forms the top layer of several plateaus, i.e. the plateau to the left side (east) of Taatsiin Gol, the Unzing Churum plateau, and extended plateaus in the northwest region. In the latter, fossil-bearing strata are locally sandwiched between basalt II and basalt III.

## Geological sections

The present study provides a detailed presentation and correlation of the elaborated sections comprising the Oligocene and lowermost Miocene strata. Middle and late Miocene sediment sequences have been excluded from this study because of ongoing investigations in this region.

We describe the sections/localities according to their location from west to east (Fig. 3a to z). A complete overview of all investigated sections/localities, fossil samples, the respective codes, coordinates, and letter zones are given in Table 3. Some assemblage lists (e.g. TGR-C/1+2) are composite. They comprise fossils of two individual samples from subsequent, time-equivalent sediment layers of the same section. All these data are published here for the first time. For some published data of the figured sections, we give references in the figure captions.

**Table 3:** Overview of the figured sections/fossil sites, fossil samples, the respective codes, coordinates, and letter zones

	Locality	Code section/locality	Code sample	Coordinates	Letter zone
Fig. 4	Luuny Yas	LUS	LUS-027	N 45°32′05.5″	D
				E 100°56′49.9″	
Fig. 4	Luuny Yas	LUS	LUS-028	N 45°32′06.4″	D
				E 100°56′54.5″	
Fig. 4	Luuny Yas	LUS	LUS-029	N 45°32′20.9	D
				E 100°00′51.3″	
Fig. 5	Luugar Khudag	LOG-A	LOG-A/1	N 45°32′19.6″	D
				E 101°00′51.3″	
Fig. 6	Abzag Ovo	ABO-A	ABO-A/3	N 45°34′25.4″	C
				E 101°03′49.7″	
Fig. 7	Toglorhoi	TGW-A	TGW-A/1-2		C
Fig. 7	Toglorhoi	TGW-A	TGW-A/3-4		C1
Fig. 7	Toglorhoi	TGW-A	TGW-A/5	N 45°22′37.6″	C1
				E 101°05′49.2″	
Fig. 8	Khongil	HL-A	HL-A/1-2	N 45°27′40.4″	A
				E 101°09′18.5″	
Fig. 9	Huch Teeg	RHN-A	RHN-A/12	N 45°29′29.9″	D
				E 101°12′17.1″	
Fig. 9	Huch Teeg	RHN-A	RHN-020	N 45°29′29.8″	D
				E 101°12′18.3″	
Fig. 9	Huch Teeg	RHN-A	RHN-021; RHN-A/11	N 45°29′30.6″	C1-D
				E 101°12′19.1″	
Fig. 9	Huch Teeg	RHN-A	RHN-019; RHN-A/10	N 45°29′30.5″	C1
				E 101°12′20.2″	
Fig. 9	Huch Teeg	RHN-A	RHN-A/7; RHN-A/8-9	N 45°29′36.0″	C1
				E 101°12′22.2″	
Fig. 9	Huch Teeg	RHN-A	RHN-023	N 45°29′33.6″	C1
				E 101°12′30.0″	
Fig. 10	Hotuliin Teeg	HTE	HTE-057	N 45°28′54.2″	C1
Fig. 11d				E 101°12′26.2″	
Fig. 11c	Hotuliin Teeg	HTE	HTE-008; HTE-003; HTE-009; HTE-014-018	N 45°29′07.4″	D
				E 101°11′58.9″	
	Hotuliin Teeg	HTE	HTE-007	N 45°29′08.2″	D
				E 101°11′49.3″	
Fig. 11b	Hotuliin Teeg	HTE	HTE-005; HTE-12/6; HTE-12/8	N 45°29′09.7″	D
				E 101°11′49.0″	D
Fig. 11b	Hotuliin Teeg	HTE	HTE-012; HTE-12/7	N 45°29′11.9″	D
				E 101°11′49.3″	
Fig. 11f	Hotuliin Teeg	HTSE	HTSE-009	N 45°28′49.2″	C1
				E 101°11′55.0″	
Fig. 11f	Hotuliin Teeg	HTSE	HTSE-013	N 45°28′49.9″	C1
				E 101°11′57.2″	
Fig. 11e	Hotuliin Teeg	HTS	HTS-056/1+2	N 45°28′53.2″	C1-D
				E 101°11′34.9″	
Fig. 11e	Hotuliin Teeg	HTS	HTS-056/3	N 45°28′54.5″	C1-D
				E 101°11′36.3″	
Fig. 11a	Unkheltseg	UNCH-A	UNCH-A/3B+4B	N 45°27′40.1″	B
Fig. 12a–d				E 101°12′04.4″	
Fig. 11a	Unkheltseg	UNCH-A	UNCH-A/3+4	N 45°27′40.1″	D
Fig. 12a–d				E 101°12′04.4″	
Fig. 13	Taatsiin Gol (south)	TGR-C	TGR-C/1	N 45°23′10.9″	C
				E 101°14′34.9″	
Fig. 13	Taatsiin Gol (south)	TGR-C′	TGR-C′/1	N 45°23′12.3″	C
				E 101°14′35.4″	
	Taatsiin Gol (right)	TGR-ZO	TGR-ZO/1+2	N 45°24′13.5″	?B
				E 101°15′53.0″	
Figs. 14 and 15	Taatsiin Gol (right)	TGR-B′	TGR-B/1	N 45°24′47.3″	B
				E 101°15′23.2″	
Fig. 14	Taatsiin Gol (right)	TGR-AB	TGR-AB (basis)	N 45°25′08.8″	Eocene
				E 101°15′39.2″	
Figs. 14 and 15	Taatsiin Gol (right)	TGR-AB	TGR-AB/21	N 45°24′41.1″	B
				E 101°15′24.7″	
Fig. 14	Taatsiin Gol (right)	TGR-AB	TGR-AB/22		B
Fig. 14	Taatsiin Gol (right)	TGR-A	TGR-A/13+14	N 45°25′12.5″	A
				E 101°15′44.3″	
Fig. 16	Taatsiin Gol (left)	TGL-A	TGL-A/1+2	N 45°26′57.4″	A
				E 101°16′20.9″	
Fig. 17	Taatsiin Gol (left)	TGL-A′	TGL-A/11		B
Figs. 18–19	Unzing Churum	TAR-A	TAR-A/2	N 45°31′14.4″	C
				E 101°18′19.2″	
Fig. 20	Del	DEL-B	DEL-B/7+8	N 45°27′10.2″	B
				E 101°22′22.3″	
Fig. 20	Del	DEL-B	DEL-B/12		C1
Figs. 21 and 22	Tatal Gol				
Fig. 22a	Tatal Gol	TAT	TAT-051/2	N 45°18′08.2″	C1
				E 101°37′09.3″	
Fig. 22a	Tatal Gol	TAT	TAT-051/1		C1
Fig. 22a	Tatal Gol	TAT	TAT-054	N 45°18′07.6″	B
				E 101°37′09.7″	
Fig. 22a	Tatal Gol	TAT	TAT-052/2	N 45°18′09.4″	C1-D
				E 101°37′14.5″	
Fig. 22a	Tatal Gol	TAT	TAT-052/1		C1
Fig. 22a	Tatal Gol	TAT-E	TAT-E/32	N 45°18′12.6″	C1-D
				E 101°37′15.7″	
Fig. 21a	Tatal Gol	TAT-E	TAT-E/27		C1
Fig. 21a	Tatal Gol	TAT-E	TAT-E/22		C1
Fig. 22b	Tatal Gol	TAT-E	TAT-044	N 45°18′00.5″	C1
				E 101°37′20.6″	
Fig. 22b	Tatal Gol	TAT-E	TAT-043	N 45°17′59.8″	C1
				E 101°37′17.1″	
Fig. 22b	Tatal Gol	TAT-E	TAT-055	N 45°17′59.0″	C
				E 101°37′16.6″	
Fig. 22b	Tatal Gol	TAT-E	TAT-E/3	N 45°14′58.2″	B
				E 101°37′16.6″	
	Tatal Gol	TAT	TAT-038	N 45°17′56.0″	B
				E 101°37′10.9″	
	Tatal Gol	TAT	TAT-037	N 45°17′54.1″	A
				E 101°37′11.7″	
Fig. 22c	Tatal Gol	TAT-D	TAT-D/1	N 45°17′52.2″	A
				E 101°37′18.5″	
Fig. 23	Tatal Gol	TAT-C	TAT-C/1-3	N 45°18′19.5″	A
				E 101°38′00.0″	
Fig. 23	Tatal Gol	TAT-C	TAT-C/6-7		B
Fig. 24	Hsanda Gol	SHG-C	SHG-C/1-2	N 45°15′49.9″	A
				E 101°43′04.9″	
Fig. 25	Hsanda Gol	SHG-A	SHG-A/6-20		B
Fig. 25	Hsanda Gol	SHG-AB	SHG-AB/15-20		B
Fig. 25	Hsanda Gol	SHG-AB	SHG-top		C1
Fig. 25	Hsanda Gol	SHG-D	SHG-D/12	N 45°16′11.8″	Sandstone
				E 101°45′55.9″	
Fig. 25	Hsanda Gol	SHG-D	SHG-D/12-26		B
	Loh	LOH-C	LOH-C/1		C1
Fig. 26	Loh	LOH-B	LOH-B/3	N 45°17′04.9″	C1
				E 101°47′22.7″	
Fig. 27	Talyn Churum	GRAB	GRAB-II	N 45°16′50.4″	A
				E 101°57′28.4″	
Fig. 28	Ikh Argalatyn Nuruu	IKH-A	IKH-A/1	N 45°17′48.4″	B
			IKH-A/2-4	E 102°04′57.2″	
Fig. 28	Ikh Argalatyn Nuruu	IKH-A	IKH-A/5	N 45°17′49.1″	C1
				E 102°05′00.7″	
Fig. 29	Ikh Argalatyn Nuruu	IKH-B	IKH-B/2	N 45°17′32.6″	B
				E 102°05′34.2″	
Fig. 29	Ikh Argalatyn Nuruu	IKH-B	IKH-B/5		C1

### **Locality Luuny Yas**

#### Samples: LUS-027, LUS-028, LUS-029 (=LUS-078)

**Fig. 4:**
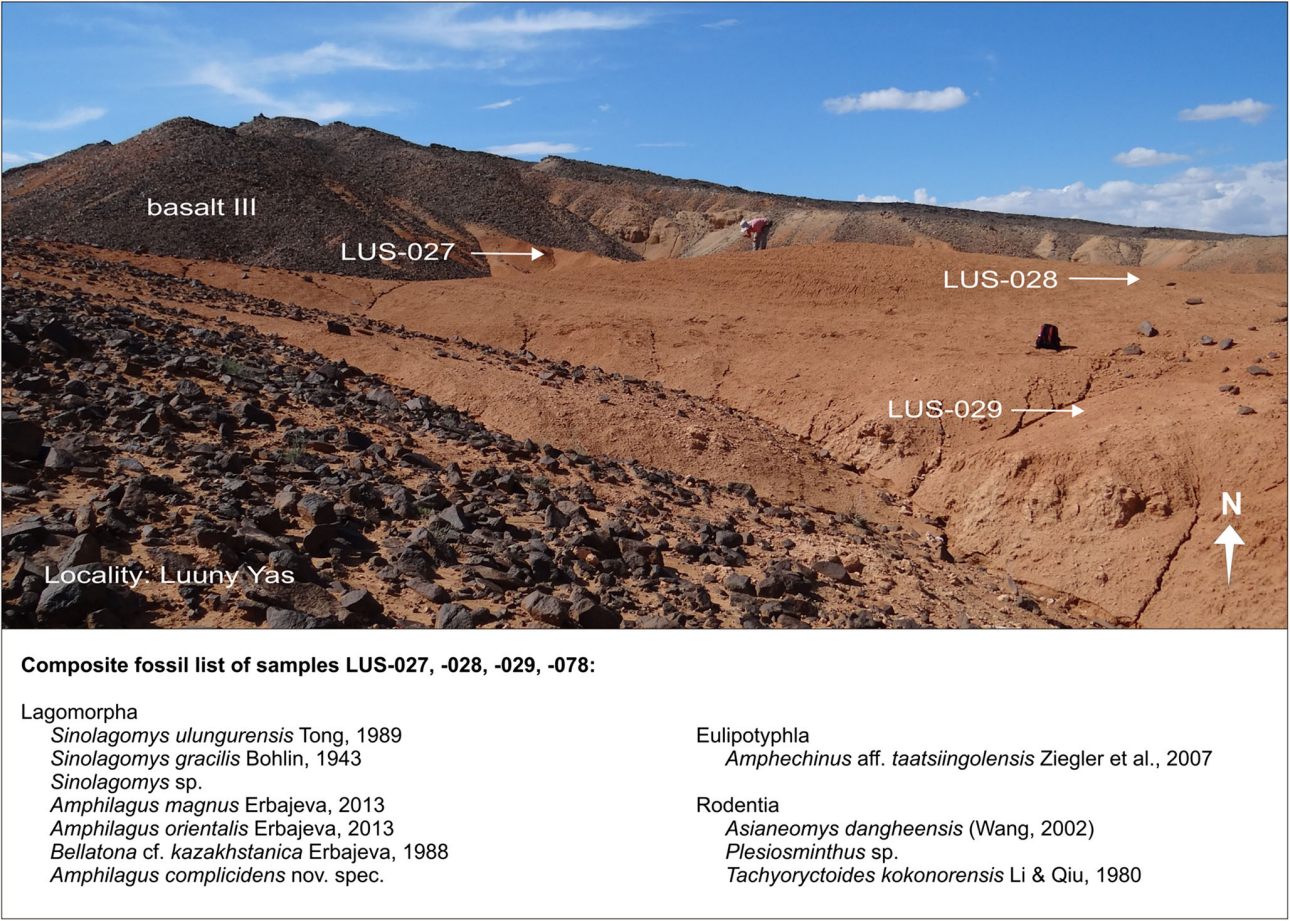
The locality name Luuny Yas means “dragon bone”. *Arrows* mark the fossil points LUS-027, LUS-028, and LUS-029 (= LUS-078)

Luuny Yas is the westernmost fossil point of the study area (Fig. 3a), first recognised during geological mapping in 1997. Later, in the field seasons 2006, 2011, and 2012, fossils were collected from the surface at three locations (LUS-027, LUS-028, and LUS-029; Fig. 4). So far, no geological section has been studied in detail. In Luuny Yas, the red-brown sandy silts of the Loh Fm. are topped by basalt III. At LUS-029, fossil concentrations are visible on top of a caliche layer. From this site, a test sample (sample LUS-078/∼500 kg) was investigated. The lower Miocene and letter zone D are indicated by the small mammals (composite fossil list below).

### **Locality Luugar Khudag**

#### Sample: LOG-A/1

**Fig. 5:**
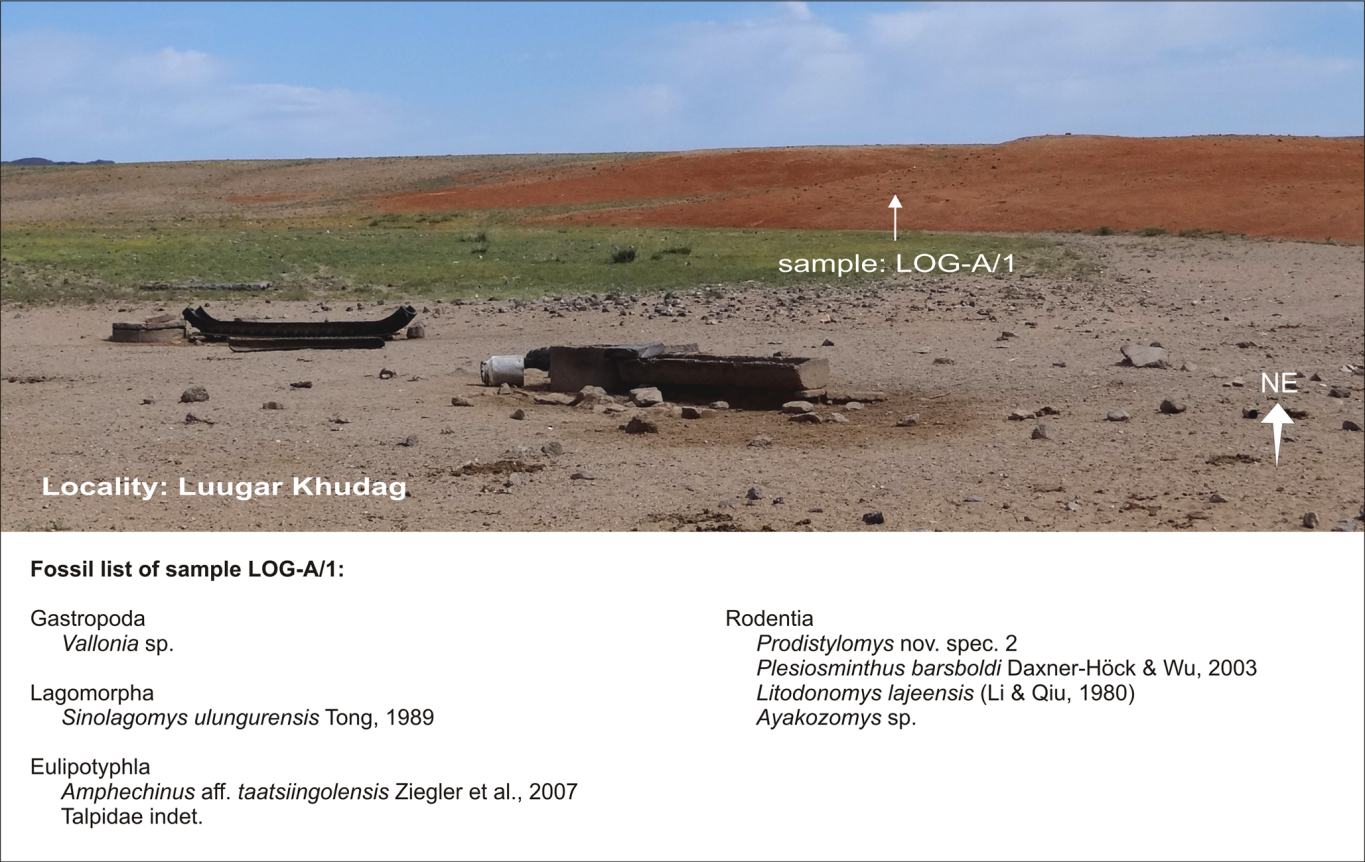
Sample point LOG-A/1 from the locality Luugar Khudag, Valley of Lakes

Luugar Khudag is located in the northwestern part of the study area (Fig. 3b). The palaeontological sample LOG-A/1 (∼500 kg brick-red sandy silt of the Loh Fm.) was taken close to a well in the dry river bed (Fig. 5). The lower Miocene is indicated by characteristic fossils of letter zone D.

### **Locality Abzag Ovo**

#### Section: ABO-A; samples: ABO-A/3,-083

**Fig. 6:**
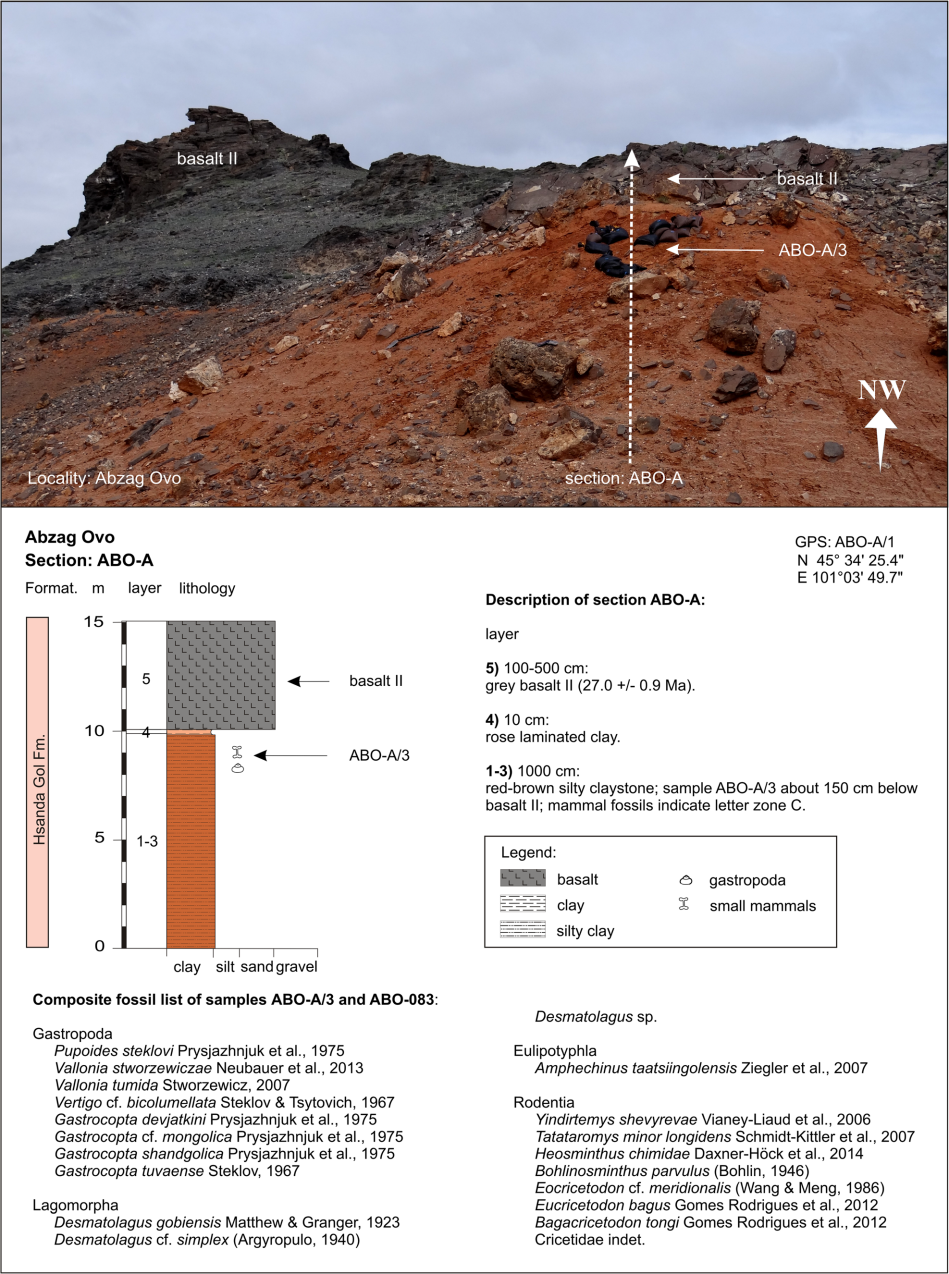
In Abzag Ovo, the samples ABO-A/3 and ABO-083 were taken from red silty claystone 1–2 m below the late Oligocene basalt II

Abzag Ovo and the section ABO-A are located ∼30 km northwest of the Taatsiin Gol (Fig. 3c). At Abzag Ovo, the red silty claystone of the Hsanda Gol Fm. is up to 10 m thick. It is topped by a 1–5-m-thick basalt II, which was dated at 27.0 ± 0.9 Ma (^40^Ar/^39^Ar age). The palaeontological samples ABO-A/3 (∼500 kg sampled 1997) and ABO-083 (∼500 kg sampled 1997 and 2011) were taken 1–2 m below basalt II (Fig. 6). The two samples yield identical fossils. Basalt II and the small mammal assemblage indicate a late Oligocene age and letter zone C. Abzag Ovo is one of the rare assemblages yielding land gastropods.

### **Locality Toglorhoi**

#### Section: TGW-A; samples: TGW-A/1-5

**Fig. 7:**
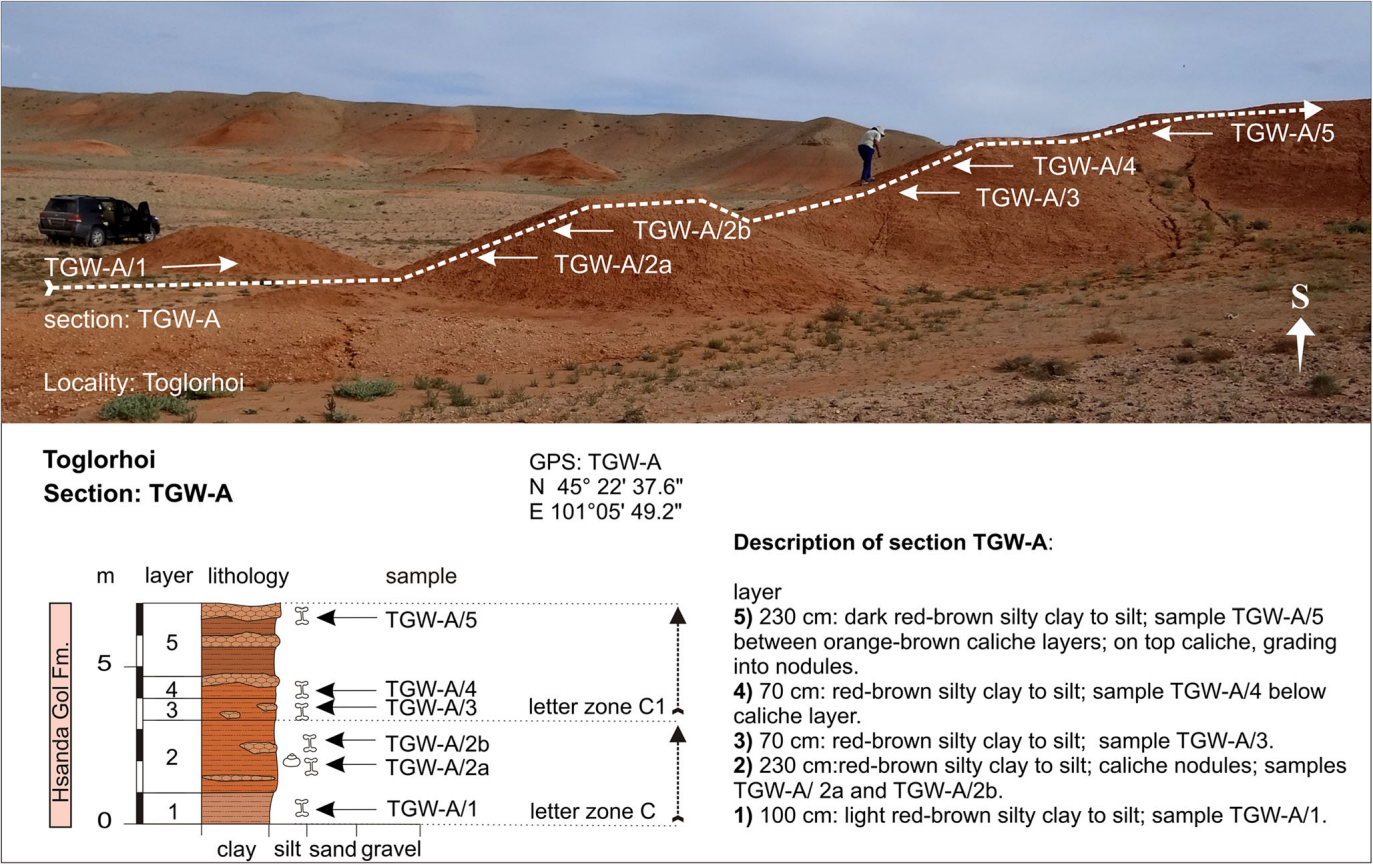
Section TGW-A from the locality Toglorhoi is located in the Khunug Valley, west of the Taatsiin Gol region

The locality Toglorhoi is located in the Khunug Valley, west of the Taatsiin Gol region (Fig. 3d). The section comprises ∼7 m of red-brown sediments of the Hsanda Gol Fm. (Fig. 7). Fossil concentrations are mostly bound to caliche layers and caliche nodules. The colour of the silty clay grades from red-brown (TGW-A/1) to dark red-brown in its higher part (TGW-A/5). Bulk samples of several tons were investigated from all horizons with visible fossil content (TGW-A/1-5). Samples TGW-A/1, TGW-A/2a, and TGW-A/2b yield index fossils of letter zone C. The prevailing fossils of samples TGW-A/3, TGW-A/4, and TGW-A/5 from the higher part of the section are *Tsaganomys* and the large ctenodactylid *Yindirtemys deflexus*; the latter is an excellent marker of letter zone C1 (Table 4). The entire sequence is of late Oligocene age.

**Table 4:** Fossil list from the locality Toglorhoi (section—TGW-A, samples—TGW-A/1, TGW-A/2a+2b, TGW-A/3+4, TGW-A/5) [the age of the assemblages is late Oligocene (letter zones C and C1)]

Toglorhoi	TGW-A/1	TGW-A/2a+b	TGW-A/3+4	TGW-A/5
Letter zone	C	C	C1	C1
Gastropoda				
*Vallonia* sp.		x		
Lagomorpha				
*Ordolagus* cf. *teilhardi* (Burke, 1941)		x		
*Desmatolagus gobiensis* Matthew and Granger, 1923		x	x	
*Desmatolagus* cf. *simplex* (Argyropulo, 1940)		x	x	
*Desmatolagus* cf. *chinensis* Erbajeva and Sen, 1998		x		
*Desmatolagus* cf. *orlovi* (Gureev, 1960)		x	x	x
*Desmatolagus* sp.	x	x		x
*Bohlinotona* cf. *pusilla* (Teilhard de Chardin, 1926)	x	x		
*Sinolagomys badamae* nov. spec. Erbajeva et al. (2017, this issue.)			x	
*Sinolagomys kansuensis* Bohlin, 1937				x
*Sinolagomys major* Bohlin, 1937				x
*Sinolagomys* sp.				x
Eulipotyphla				
*Zaraalestes minutus* (Matthew and Granger, 1924a)				x
*Palaeoscaptor acridens* Matthew and Granger, 1924a		x		
*Palaeoscaptor gigas* (Lopatin, 2002)		x	x	x
*Palaeoscaptor tenuis* Ziegler et al., 2007		x		
*Amphechinus taatsiingolensis* Ziegler et al., 2007	x	x		
*Amphechinus minutissimus* Ziegler et al., 2007				x
*Amphechinus major* Ziegler et al., 2007				x
Erinaceidae indet.		x		
Talpidae indet.		x		x
Crocidosoricidae indet.		x		
Rodentia				
*Ninamys arboraptus* (Shevyreva, 1966)	x			
*Proansomys badamae* sp. nov. Maridet et al., 2017﻿, this issue		x		x
*Asianeomys bolligeri* (Lopatin, 2000)		x		
*Tataromys sigmodon* Matthew and Granger, 1923		x		
*Tatataromys minor longidens* Schmidt-Kittler et al., 2007		x	x	
*Tataromys plicidens* Matthew and Granger, 1923			x	
*Yindirtemys deflexus* (Teilhard de Chardin, 1926)			x	x
*Cyclomylus intermedius* Wang, 2001		x		
Tsaganomyidae indet.	x	x		
*Coelodontomys asiaticus* Wang, 2001	x			
*Tsaganomys altaicus* Matthew and Granger, 1923	x	x	x	x
*Allosminthus minutus* (Daxner-Höck, 2001)	x	x		
*Heosminthus* sp.		x		
*Bohlinosminthus parvulus* (Bohlin, 1946)	x	x		x
*Parasminthus* cf. *tangingoli* Bohlin, 1946		x		
*Eocricetodon meridionalis* (Wang and Meng, 1986)		x		
*Eucricetodon bagus* Gomes Rodrigues et al., 2012		x		
*Eucricetodon jilantaiensis* Gomes Rodrigues at al., 2012	x	x		
Cicetidae indet.		x		
*Bagacricetodon tongi* Gomes Rodrigues et al., 2012		x		
*Aralocricetodon schokensis* Bendukidze, 1993		x		
*Argyromys cicigei* nov. spec. López-Guerrero et al. (in prep)		x		
*Tachyoryctoides bayarmae* Daxner-Höck et al., 2015			x	
*Tachyoryctoides radnai* Daxner-Höck et al., 2015			x	
Leptictida				
Didymoconidae indet.			x	
Carnivora				
*Asiavorator altidens* Spassov and Lange-Badré, 1995				x
Ruminantia				
*Paragelocus* aff. *scotti* Schlosser, 1902	x			
Bovidae gen. 1		x		
Ruminantia indet.	x			

### **Locality Khongil**

#### Samples: HL-A/1 and HL-A/2

**Fig. 8:**
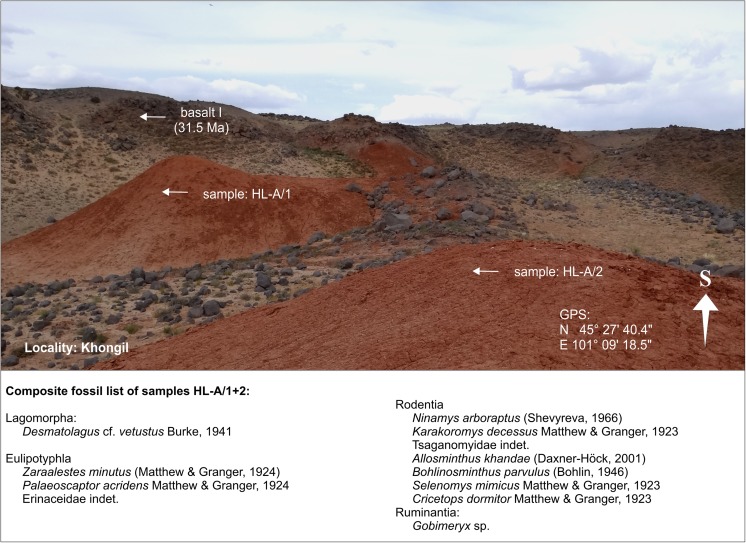
Khongil is located at the NW corner of the plateau at the orographic right side of Taatsiin Gol. *Arrows* mark two sample points HL-A/1 and HL-A/2 below the early Oligocene basalt I

Khongil is located at the NW corner of the Taatsiin plateau at the orographic right side of Taatsiin Gol (Fig. 3e). There, several metres of brick-red clay of the Hsanda Gol Fm. are exposed immediately below basalt I. The mammal fauna stems from two test samples HL-A/1 and HL-A/2 (for location, see Fig. 8). The early Oligocene age is indicated by basalt I and by respective fossils.

### **Locality Huch Teeg**

#### Section: RHN-A; samples: RHN-A/6-12, RHN-019-023

**Fig. 9:**
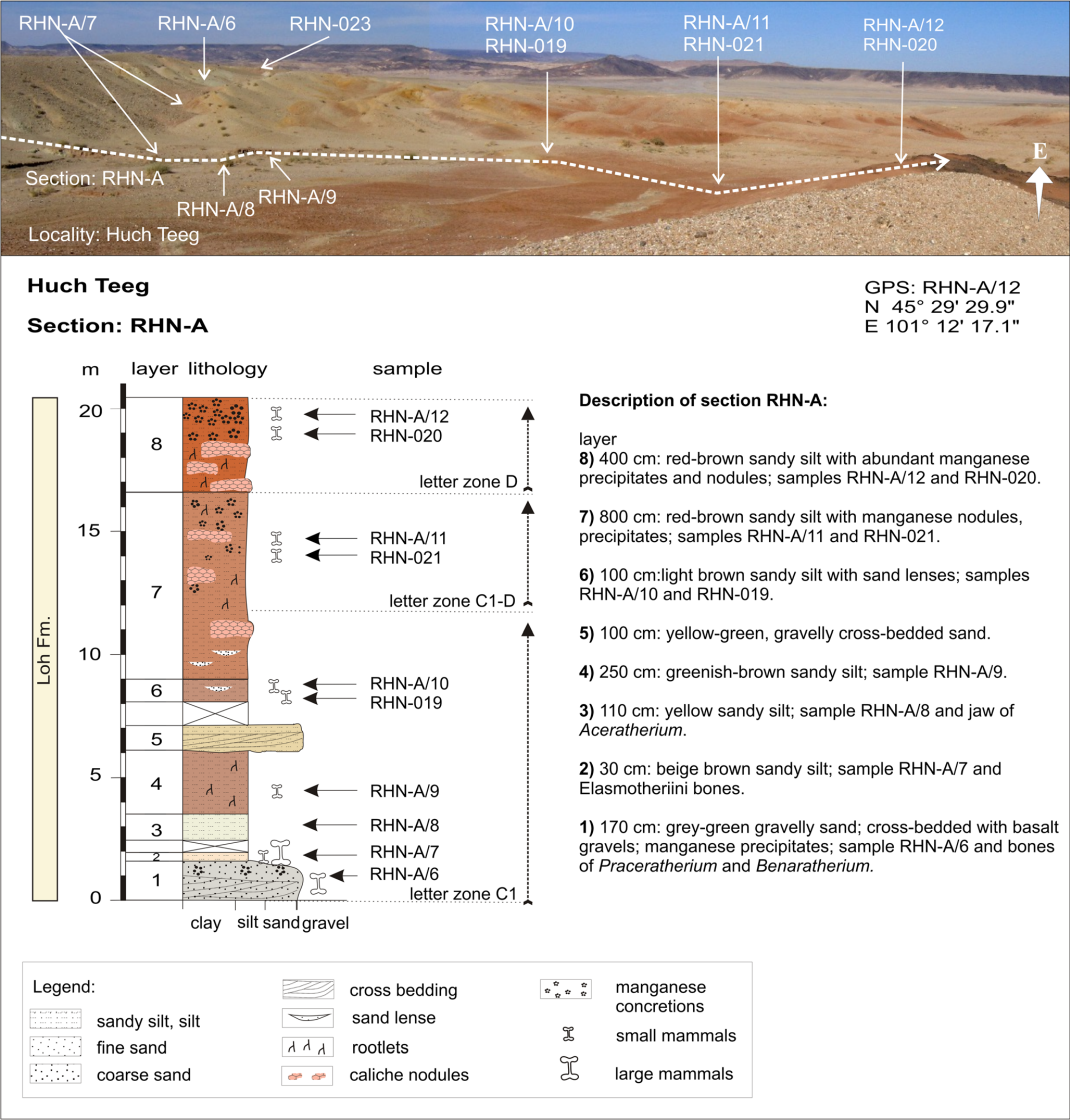
Huch Teeg is located at the orographic right side of Taatsiin Gol, north of the western Taatsiin plateau. Description of the section RHN-A modified from Schmid (1999; Abb. A5) and Daxner-Höck et al. (2013, Fig. 20.2)

Huch Teeg is located at the orographic right side of Taatsiin Gol, north of the western Taatsiin plateau (Fig. 3f). The direction of section RHN-A is N → S (Fig. 9). There, sediments of the Tsagan Ovo and Loh Fms. are exposed; the Hsanda Gol Fm. is missing. The present study does not consider the Tsagan Ovo Fm. from the northernmost part of the section. The fossil-bearing strata of the Loh Fm. (RHN-A/6-10) dip toward south. The southernmost part of the section (samples—RHN-A/11-12) is horizontally bedded and built up of light rose-brown to red-brown sandy silt.

The tilted northern part of the section (samples RHN-A/6-10, RHN-019, and RHN-023 of the Loh Fm.) yields fossils of letter zone C1, indicating the late Oligocene age. The horizontally bedded southern part (samples RHN-A/11 and RHN-021-22) starts with fossils of letter zone C1-D and ends with fossils of letter zone D (samples RHN-A/12 and RHN-020), indicating an early Miocene age (Table 5). There, concentrations of manganese precipitates and nodule are evident.

**Table 5:** Fossil list from Huch Teeg (section—RHN-A, samples—RHN-A/6-12, RHN-019-022) [the age of the assemblages is late Oligocene (letter zones C1 and C1-D) to early Miocene (letter zone D)]

Huch Teeg	RHN-A/6	RHN-A/7	RHN-023	RHN-A/8	RHN-A/9	RHN-A/10, RHN-019	RHN-A/11, RHN-021+022	RHN-A/12, RHN-020
Letter zone	C1	C1	C1	C1	C1	C1	C1-D	D
Lagomorpha								
*Desmatolagus gobiensis* Matthew and Granger, 1923		x						
*Desmatolagus robustus* Matthew & Granger, 1923					x			
*Desmatolagus* cf. *chinensis* Erbajeva and Sen, 1998		x						
*Bohlinotona* cf. *pusilla* (Teilhard de Chardin, 1926)					x			
*Sinolagomys kansuensis* Bohlin, 1937			x		x	x	x	x
*Sinolagomys major* Bohlin, 1937			x			x	x	
*Sinolagomys ulungurensis* Tong, 1989						x		x
*Sinolagomys* sp.		x			x	x	x	
*Amphilagus magnus* Erbajeva, 2013							x	x
*Eulipotyphla*								
*Palaeoscaptor* cf. *rectus* Matthew and Granger, 1924a		x	x					
*Palaeoscaptor tenuis* Ziegler et al., 2007								x
*Amphechinus taatsiingolensis* Ziegler et al., 2007						x		x
*Amphechinus minutissimus* Ziegler et al., 2007		x			x	x		
*Amphechinus major* Ziegler et al., 2007		x	x		x	x		
*Amphechinus* aff. *taatsiingolensis* Ziegler et al., 2007								x
*Tavoonyia altaica* Ziegler et al., 2007					x			
Heterosoricinae indet.		x						
Rodentia								
*Proansomys badamae* sp. nov. Maridet et al. (2017, this issue)		x						
*Ansomys* sp. 1								x
*Yindirtemys deflexus* (Teilhard de Chardin, 1926)		x	x					
*Prodistylomys taatsiini* nov. spec.Oliver et al. (in prep)								x
*Tsaganomys altaicus* Matthew and Granger, 1923		x						
*Heosminthus borrae* Daxner-Höck et al., 2014								x
*Bohlinosminthus parvulus* (Bohlin, 1946)		x	x					
*Parasminthus debruijni* Lopatin, 1999					x			
*Plesiosminthus asiaticus* Daxner-Höck and Wu, 2003		x	x					
*Plesiosminthus promyarion* Schaub, 1930					x	x		
*Plesiosminthus olzi* Daxner-Höck et al., 2014								x
*Plesiosminthus barsboldi* Daxner-Höck and Wu, 2003								x
*Litodonomys lajeensis* (Li & Qiu, 1980)								x
*Heterosminthus firmus* Zazhigin and Lopatin, 2000		x			x	x		x
*Heterosminthus* cf. *lanzhouensis* Wang and Qiu, 2000			x				x	
*Primus* sp.								x
*Tachyoryctoides* sp.							x	
Leptictida								
*Didymoconus berkey* Matthew and Granger, 1924b		x						
Didymoconidae indet.			x					
Perissodactyla								
*Paraceratherium* sp.	x							
cf. *Benaratherium* sp.	x							
*Aceratherium* (*Alicornops*) cf. *pauliacense* (Richard, 1937)				x				
Elasmotheriini indet.		x						
Rhinocerotidae indet.								x
Ruminantia								
Ruminantia indet.					x	x		

### **Locality Hotuliin Teeg**

#### Sections HTE; samples: HTE-003-018 (Fig. 11b, c), HTE-057 (Figs. 10 and 11d); HTSE-009, HTSE-013 (Fig. 11f); HTS-056/1-3 (Fig. 11e)

**Fig. 10:**
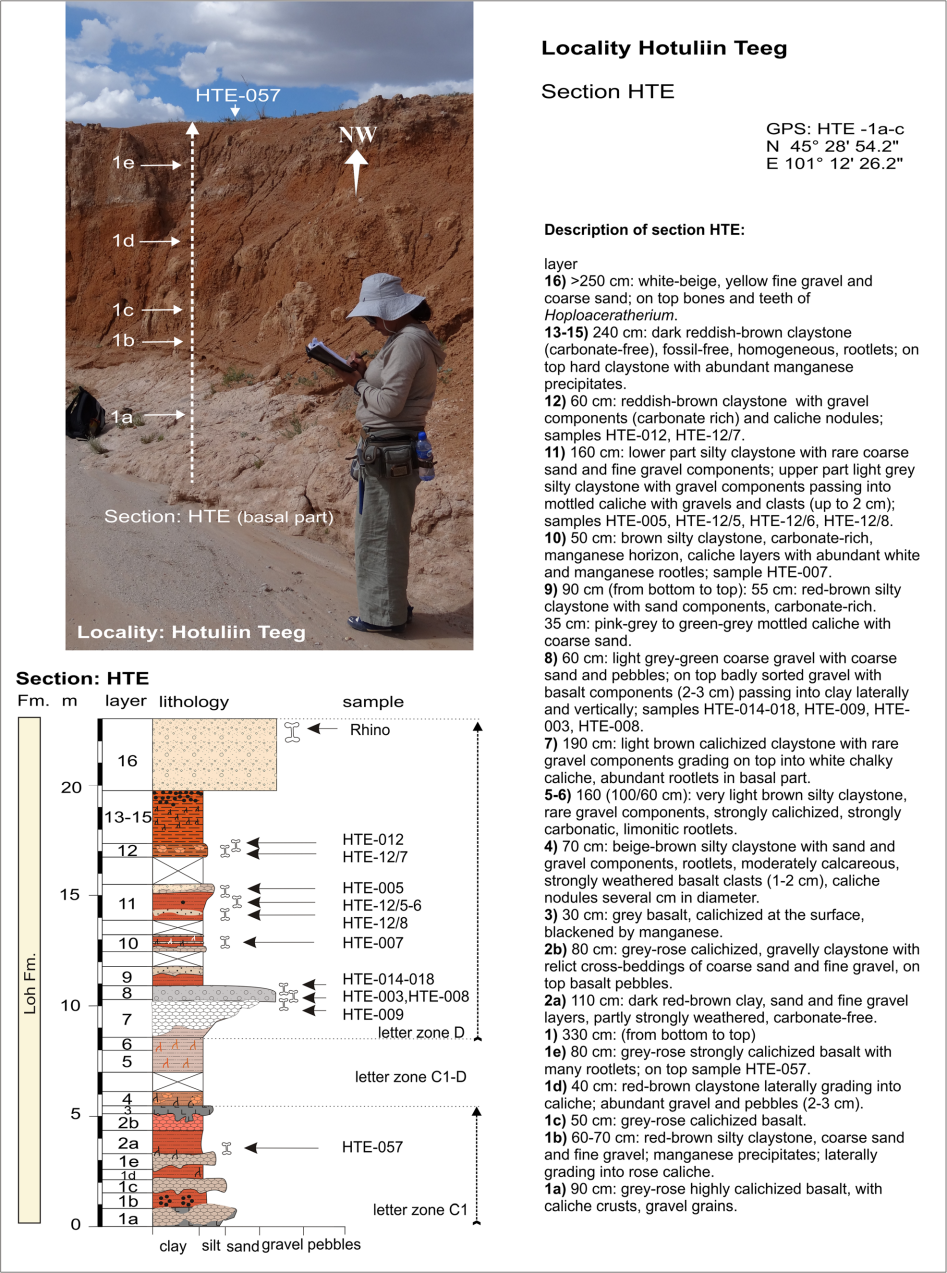
The Hotuliin Teeg section is located west of the Taatsiin Gol, in a wide NW-SE striking valley north of the main Taatsiin plateau (western plateau). The picture shows the lowermost, the late Oligocene part of the section

**Fig. 11:**
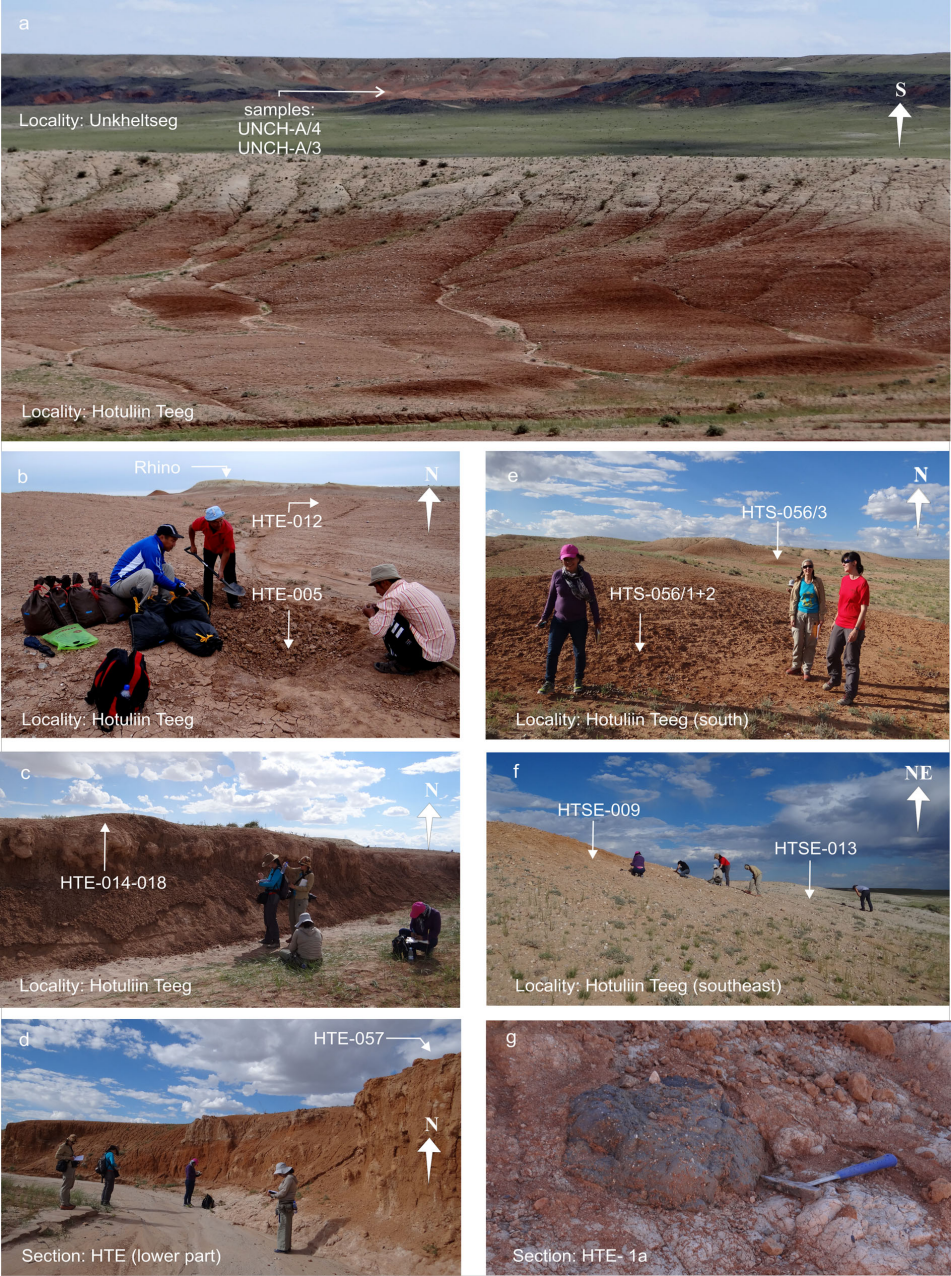
Localities Hotuliin Teeg and Unkheltseg north of the western Taatsiin plateau. **a** View in southern direction from Hotuliin Teeg to Unkheltseg at the north rim of the plateau. **b** Sample place HTE-005 (= HTE-12/5) at the upper part of the HTE-section. **c** Sample point HTE-014-018 in the middle part of the HTE-section. **d** Lower part of the HTE-section, showing red silty claystone alternating with calichized basalt, on top sample HTE-057. **e** HTS-056/1+2 and HTS-056/3. **f** HTSE-009 and HTSE-013. **g** Calichized basalt from the lowermost layer of the HTE section

The Hotuliin Teeg section (HTE) and additional fossil points (HTSE and HTS) are located north of the western Taatsiin plateau (Fig. 3g). The area is flat and comprises no more than 23 m of sediment. The section HTE (Figs. 10 and 11a) was studied along of a dry creek. In the lower part, several layers of strongly weathered basalt alternate with silty-sandy claystone. On top of this first sequence (Fig. 11d, g), the late Oligocene is indicated by fossils of letter zone C1.

Upsection, alternating beds of calichized basalt and silty clay continue. The colour changes into light brown. The claystone ultimately grades into thick white chalky caliche, which is topped by badly sorted coarse sand and gravels (Fig. 11c). The boundary horizon between the caliche and gravely sand shows significant fossil concentrations (samples HTE-014-018 from the south bank of the dry creek, Figs. 10 and 11c). Upsection, similar fossil traps were found between sand-silt layers/lenses and caliche beds. The fossils indicate letter zone D and the lower Miocene. The top layer of the HTE-section is built up by 2–3 m of beige sand and gravel, which yield fossil bones of the rhinos cf. *Hoploaceratherium gobiense* and cf. *Caementodon* sp. (Fig. 11b; Table 6).

**Table 6:** Fossils from Hotuliin Teeg (section—HTE, samples—HTE-003-012) [the age of the assemblages is early Miocene (letter zone D)]

Hotuliin Teeg (section HTE)	HTE-009	HTE-008+003	HTE-014+018	HTE-005+007	HTE*	HTE-012
Letter zone	D	D	D	D	D	D
Lagomorpha						
*Desmatolagus gobiensis* Matthew & Granger, 1923					x	
*Desmatolagus* sp.				x		x
*Sinolagomys kansuensis* Bohlin, 1937	x	x	x	x	x	
*Sinolagomys major* Bohlin, 1937	x	x	x		x	
*Sinolagomys ulungurensis* Tong, 1989	x	x	x	x	x	x
*Sinolagomys gracilis* Bohlin, 1942						x
*Amphilagus magnus* Erbajeva, 2013	x	x	x	x	x	x
*Amphilagus orientalis* Erbajeva, 2013					x	x
*Bellatona* cf. *kazakhstanica* Erbajeva, 1988						x
Eulipotyphla						
*Palaeoscaptor acridens* Matthew & Granger, 1924a		x	x			x
*Palaeoscaptor* cf. *rectus* Matthew & Granger, 1924a	x	x	x			
*Palaeoscaptor tenuis* Ziegler et al., 2007		x				
*Amphechinus taatsiingolensis* Ziegler et al., 2007		x				
*Amphechinus minutissimus* Ziegler et al., 2007	x					
*Amphechinus* aff. *taatsiingolensis* Ziegler et al., 2007		x	x	x		x
*Exallerix* sp.		x			x	
Heterosoricinae indet.						x
Crocidosoricinae indet.		x				x
Talpidae indet.				x	x	
Rodentia						
*Plesiosciurus* aff. *sinensis* Qiu & Liu, 1986		x				
*Kherem shandgoliensis* Minjin, 2004				x	x	
*Eutamias* sp.		x				
*Asianeomys dangheensis* (Wang, 2002)				x		x
*Yindirtemys suni* Li & Qiu, 1980	x	x	x		x	
*Prodistylomys* sp.			x			
*Prodistylomys mongoliensis* nov. spec. Oliver et al. (, in prep)		x				x
*Heosminthus borrae* Daxner-Höck et al., 2014			x	x		
*Bohlinosminthus parvulus* (Bohlin, 1946)		x	x			
*Plesiosminthus* sp.						x
*Plesiosminthus olzi* Daxner-Höck et al., 2014		x	x	x		
*Litodonomys huangheensis* Wang & Qiu, 2000		x		x		
*Litodonomys lajeensis* (Li & Qiu, 1980)		x	x	x	x	x
*Heterosminthus firmus* Zazhigin & Lopatin, 2000	x	x	x			
*Heterosminthus* aff. *nanus* Zazhigin & Lopatin, 2000			x	x		
*Tachyoryctoides kokonorensis* Li & Qiu, 1980	x		x	x	x	x
*Tachyoryctoides engesseri* Wang & Qiu, 2012	x	x	x		x	
*Ayakozomys* sp.					x	
Carnivora						
Carnivora indet.		x	x		x	
Perissodactyla						
cf. *Hoploaceratherium gobiense* (Beliajeva, 1960)					x	x
cf. *Caementodon* sp.					x	
Rhinocerotidae indet.		x				
Ruminantia						
Ruminantia indet.		x			x	

Five samples from a neighbouring dry creek yield fossils of letter zone C1 and letter zone C1-D. These are the samples HTSE-009 and HTSE-013 (Fig. 11f) south-east, and the samples HTS-056/1-3 (Fig. 11e) south of the Hotuliin Teeg creek. Sample HTSE-009 consists of red clay above a white-green caliche layer. On top, basalt pebbles are exposed. Sample HTSE-013 was collected between rose and white caliche layers. Both samples yield fossils of letter zone C1 (Table 7), indicating the late Oligocene. Following the dry river westward leads to the fossil points HTS-056/1+2 and HTS-056/3, which yield fossils of letter zone C1-D and indicate the uppermost Oligocene.

**Table 7:** Fossils from Hotuliin Teeg (samples—HTE-057, HTSE-009, HTSE-013, HTS-056/1-3) [the age of the assemblages is late Oligocene (letter zones C1 and C1-D)]

Hotuliin Teeg (HTE-057, HTS, HTSE)	HTE-057	HTSE-009+013	HTS-056/1-3
Letter zone	C1	C1	C1-D
Gastropoda			
*Pupoides steklovi* Prysjazhnjuk et al., 1975			x
*Vallonia stworzewiczae* Neubauer et al., 2013			x
*Vallonia tumida* Stworzewicz, 2007			x
*Gastrocopta devjatkini* Prysjazhnjuk et al., 1975			x
*Gastrocopta tuvaense* Steklov, 1967			x
Lagomorpha			
*Desmatolagus* sp.		x	
*Sinolagomys kansuensis* Bohlin, 1937		x	x
*Sinolagomys major* Bohlin, 1937		x	
*Sinolagomys* sp.	x		x
*Sinolagomys ulungurensis* Tong, 1989		x	x
*Amphilagus magnus* Erbajeva, 2013		x	x
Eulipotyphla			
*Palaeoscaptor acridens* Matthew & Granger, 1924a	x		
*Palaeoscaptor* cf. *rectus* Matthew & Granger, 1924a			x
*Palaeoscaptor tenuis* Ziegler et al., 2007		x	x
Erinaceidae indet.	x		x
*Amphechinus taatsiingolensis* Ziegler et al., 2007		x	x
*Amphechinus minutissimus* Ziegler et al., 2007	x		x
*Amphechinus major* Ziegler et al., 2007	x	x	x
*Taatsiinia hoeckorum* Ziegler et al., 2007			x
Rodentia			
*Tataromys sigmodon* Matthew & Granger, 1923		x	
*Yindirtemys deflexus* (Teilhard de Chardin, 1926)	x	x	
*Yindirtemys suni* Li & Qiu, 1980			x
*Tsaganomys altaicus* Matthew & Granger, 1923		x	x
*Bohlinosminthus parvulus* (Bohlin, 1946)	x		x
*Plesiosminthus promyarion* Schaub, 1930			x
*Litodonomys lajeensis* (Li & Qiu, 1980)			x
*Heterosminthus firmus* Zazhigin & Lopatin, 2000			x
*Heterosminthus* cf. *lanzhouensis* Wang & Qiu, 2000			x
*Tachyoryctoides obrutschewi* Bohlin, 1937	x		
*Tachyoryctoides tatalgolicus* Dashzeveg, 1971		x	
Creodonta			
Hyaenodontidae indet.		x	
Leptictida			
Didymoconidae indet.		x	
Perissodactyla			
Rhinocerotidae indet.			x
Ruminantia			
Ruminantia indet.		x	

### **Locality Unkheltseg**

#### Samples: UNCH-A/3 and UNCH-A/4

**Fig. 12:**
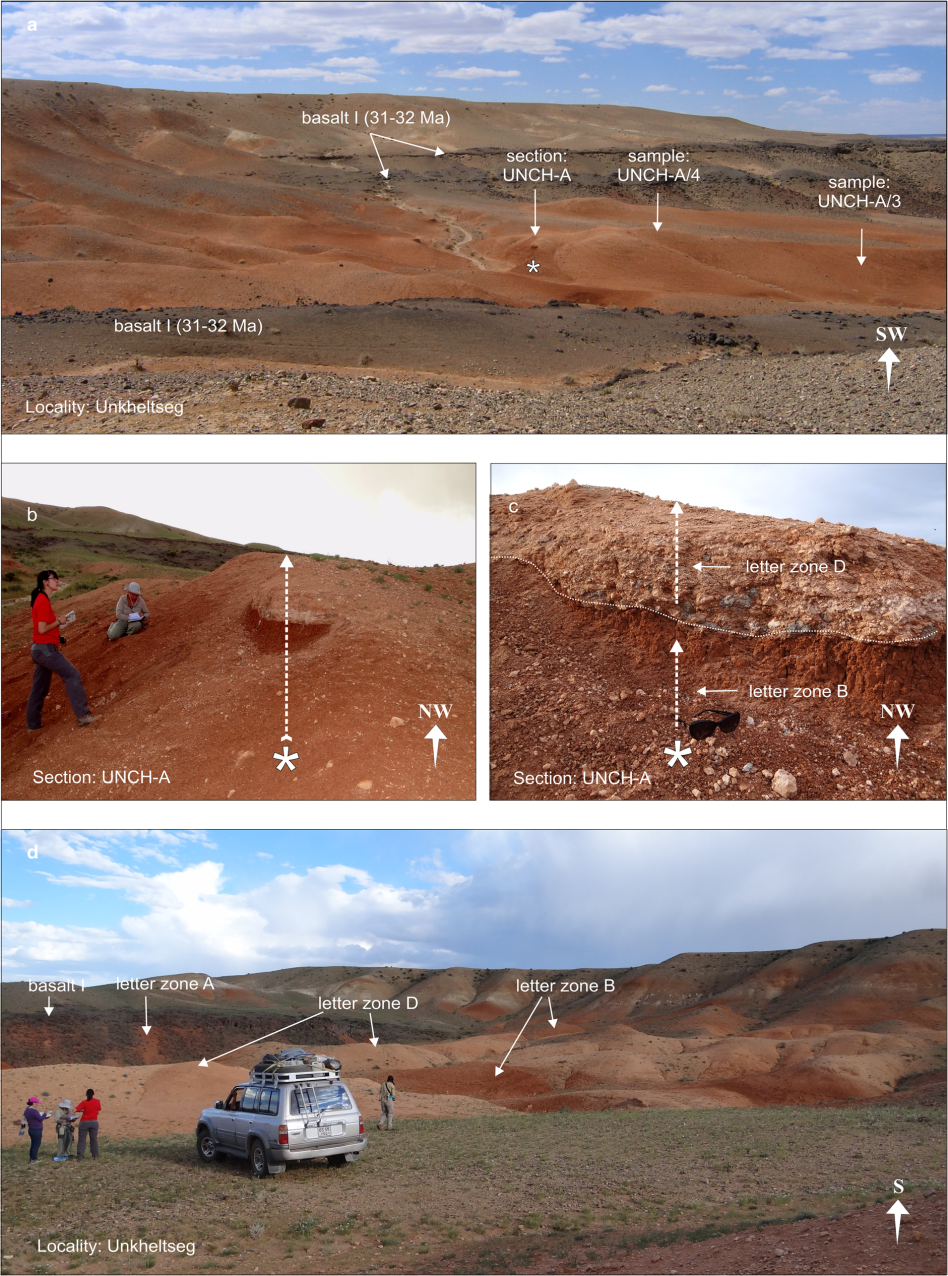
Locality Unkheltseg showing the sample points UNCH-A/3 and UNCH-A/4. **a** View from Unkheltseg toward southwest. Localization of section UNCH-A and samples UNCH-A/3 and UNCH-A/4. **b**, **c** Section UNCH-A: The contact zone between the Hsanda Gol Fm. with fossils of letter zone B and the Loh Fm. with fossils of letter zone D is marked. **d** View toward the north rim of the Taatsiin Plateau. The brick-red colour indicates the Hsanda Gol Fm. with fossils of letter zones A and B; the rose silt indicates the Loh Fm. with fossils of letter zone D

Unkheltseg is located at the northern rim of the Taatsiin plateau, west of the Taatsiin Gol (Fig. 3h). In this area, basalt I is interrupted, and the brick-red clay of the Hsanda Gol Fm. is immediately overlain by a thin layer of rose silty sand and gravels of the Loh Fm. mixed with abundant caliche nodules and reworked basalt (section UNCH-A; Fig. 12b, c). Here, the Loh Fm. yields fossils of letter zone D; the Hsanda Gol clay yields fossils of letter zone B. When we investigated the first bulk samples UNCH-A/3 and UNCH-A/4, years ago, the formation boundary of section UNCH-A was not visible; thus, fossils of letter zone B and D were mixed in both samples. Later, the fossils could easily be separated into two parts, one of letter zone B and the second of letter zone D (Table 8).

**Table 8:** Fossils from the locality Unkheltseg (samples—UNCH-A/3B+4B and UNCH-A/3+4) [the ages of the assemblages are early Oligocene (letter zone B) and early Miocene (letter zone D)]

Unkheltseg (UNCH-A/3B+4B)	Unkheltseg (UNCH-A/3+4)
Letter zone B	Letter zone D
Gastropoda	Lagomorpha
?*Strobilops* sp.	*Sinolagomys major* Bohlin, 1937
Lagomorpha	*Sinolagomys ulungurensis* Tong, 1989
*Desmatolagus gobiensis* Matthew & Granger, 1923	*Sinolagomys* sp.
*Desmatolagus* cf. *simplex* (Argyropulo, 1940)	*Amphilagus magnus* Erbajeva, 2013
*Desmatolagus* sp.	*Amphilagus orientalis* Erbajeva, 2013
Eulipotyphla	*Amphilagus plicadentis* Erbajeva, 2013
*Zaraalestes minutus* (Matthew & Granger, 1924a)	*Bellatona* cf. *kazakhstanica* Erbajeva, 1988
*Palaeoscaptor acridens* Matthew & Granger, 1924a	*Bellatona yanghuensis* Zhou, 1988
*Palaeoscaptor tenuis* Ziegler et al., 2007	*Alloptox* cf. *minor* Li, 1978
*Gobisorex kingae* Sulimski, 1970	Eulipotyphla
Crocidosoricinae indet.	*Palaeoscaptor acridens* Matthew & Granger, 1924a
Rodentia	*Amphechinus* aff. *taatsiingolensis* Ziegler et al., 2007
*Ninamys kazimierzi* Vianey-Liaud et al., 2013	*Exallerix* sp.
*Ninamys arboraptus* (Shevyreva, 1966)	Crocidosoricinae indet.
*Huangomys frequens* Schmidt-Kittler et al., 2007	Rodentia
*Ardynomys* sp.	*Plesiosciurus* aff. *sinensis* Qiu & Liu, 1986
*Anomoemys lohiculus* (Matthew & Granger, 1923)	*Kherem shandgoliensis* Minjin, 2004
Tsaganomyidae indet.	Pteromyini indet.
*Coelodontomys asiaticus* Wang, 2001	*Eutamias* sp.
*Tsaganomys altaicus* Matthew & Granger, 1923	*Asianeomys dangheensis* (Wang, 2002)
*Heosminthus chimidae* Daxner-Höck et al., 2014	*Yindirtemys suni* Li & Qiu, 1980
*Heosminthus* sp.	*Prodistylomys* nov. spec. Oliver et al. (in prep)
*Heosminthus borrae* Daxner-Höck et al., 2014	*Plesiosminthus barsboldi* Daxner-Höck & Wu, 2003
*Onjosminthus baindi* Daxner-Höck et al., 2014	*Litodonomys huangheensis* Wang & Qiu, 2000
*Shamosminthus sodovis* Daxner-Höck, 2001	*Litodonomys lajeensis* (Li & Qiu, 1980)
*Cricetops dormitor* Matthew & Granger, 1923	*Heterosminthus firmus* Zazhigin & Lopatin, 2000
*Witenia* sp.	*Heterosminthus* aff. *nanus* Zazhigin & Lopatin, 2000
*Eocricetodon meridionalis* (Wang & Meng, 1986)	*Tachyoryctoides kokonorensis* Li & Qiu, 1980
*Eucricetodon asiaticus* Matthew & Granger, 1923	*Democricetodon sui* Maridet et al. 2011
*Eucricetodon bagus* Gomes Rodrigues et al., 2012	Perissodactyla
Carnivora	cf. *Hoploaceratherium gobiense* (Beliajeva, 1960)
*Shandgolictis elegans* Hunt, 1998	cf. *Caementodon* sp.
*Nimravus mongoliensis* (Gromova, 1959)	Ruminantia
Leptictida	Ruminantia indet.
*Didymoconus berkey* Matthew & Granger, 1924b	
Ruminantia	
*Pseudomeryx* sp.	
*Paragelocus* aff. *scotti* Schlosser, 1902	

### **Locality Taatsiin Gol (south of the western plateau)**

#### Sections: TGR-C and TGR-C′; samples: TGR-C/1, TGR-C/2, TGR-C/5+6, TGR-C/7 (Fig. 3i, Fig. 13)

The lower part of the sections consists of red-brown claystone alternating with red-rose caliche layers (layers—TGR-C/1-10). It is overlain by 55 cm of dark brown claystone, a thin layer of orange-pink caliche, and red claystone (sediment layers—TGR-C/11-13). The dark brown claystone and orange-pink caliche (TGR-C/11) mark the boundary between letter zones C and C1. The samples TGR-C/1+2 are very fossil-rich, which indicate letter zone C (Table 9). Upsection, olive-green claystone layers alternate with white chalky caliche and grade into red-brown caliche (layers—TGR-C/14-19 with poor fossil content).

**Fig. 13:**
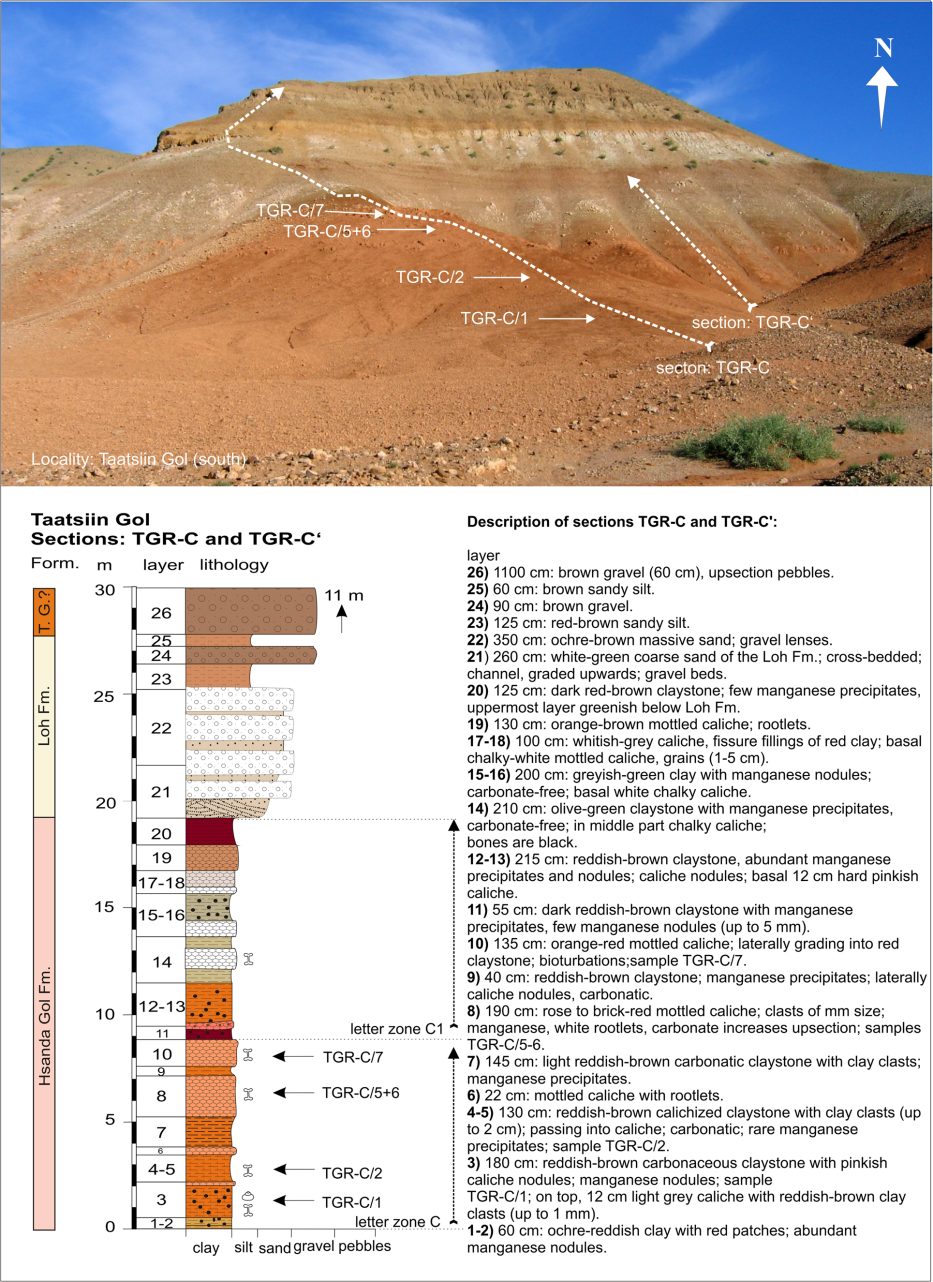
The sections TGR-C and TGR-C′ are located at the southeast rim of the Taatsiin plateau (western plateau) and are exposed toward south. The lower part shows the Hsanda Gol Fm., the upper part is built up by units of the Loh and Tuyn Gol Fms. and ?Pleistocene gravels

**Table 9:** Fossils from the locality Tatsiin Gol south (composite samples—TGR-C/1+2 and TGR-C/5-7) [the age of the assemblages is late Oligocene (letter zone C)]

Taatsiin Gol (section TGR-C)
TGR-C/1+2
Gastropoda
*Vallonia* cf. *lepida* (Reuss, 1849)
*Vallonia* sp.
Lagomorpha
*Desmatolagus gobiensis* Matthew & Granger, 1923
*Desmatolagus* cf. *simplex* (Argyropulo, 1940)
*Desmatolagus* cf. *chinensis* Erbajeva & Sen, 1998
*Desmatolagus* cf. *orlovi* (Gureev, 1960)
*Desmatolagus* sp.
*Bohlinotona* cf. *pusilla* (Teilhard de Chardin, 1926)
*Sinolagomys* sp.
Eulipotyphla
*Palaeoscaptor acridens* Matthew & Granger, 1924a
*Palaeoscaptor gigas* (Lopatin, 2002)
*Palaeoscaptor tenuis* Ziegler et al., 2007
*Amphechinus taatsiingolensis* Ziegler et al., 2007
*Exallerix pustulatus* Ziegler et al., 2007
*Gobisorex kingae* Sulimski, 1970
Crocidosoricinae indet.
Rodentia
*Proansomys badamae* sp. nov. Maridet et al. (this vol.)
Ansomyinae indet.
*Asianeomys bolligeri* (Lopatin, 2000)
*Tataromys sigmodon* Matthew & Granger, 1923
*Tatataromys minor* longidens Schmidt-Kittler et al., 2007
*Cyclomylus biforatus* Wang, 2001
*Coelodontomys asiaticus* Wang, 2001
*Tsaganomys altaicus* Matthew & Granger, 1923
*Heosminthus chimidae* Daxner-Höck et al., 2014
*Bohlinosminthus parvulus* (Bohlin, 1946)
*Parasminthus* cf. *tangingoli* Bohlin, 1946
*Parasminthus debruijni* Lopatin, 1999
*Litodonomys huangheensis* Wang & Qiu, 2000
*Tachyoryctoides radnai* Daxner-Höck et al., 2015
*Tachyoryctoides bayarmae* Daxner-Höck et al., 2015
*Eucricetodon bagus* Gomes Rodrigues et al., 2012
*Eucricetodon jilantaniensis* Gomes Rodrigues et al., 2012
*Bagacricetodon tongi* Gomes Rodrigues et al., *2012*
*Aralocricetodon schokensis* Bendukidze, 1993
Carnivora
*Amphicynodon* sp.
*Shandgolictis elegans* Hunt, 1998
cf. *Asiavorator* sp.
*Palaeogale* sp.
Leptictida
*Didymoconus colgatei* Matthew & Granger, 1924b
Ruminantia
*Dremotherium* cf. *guthi* Jehenne, 1987
Ruminantia indet.
TGR-C/5-7
Gastropoda
*Vallonia* cf. *lepida* (Reuss, 1849)
Lagomorpha
*Desmatolagus* cf.*chinensis* Erbajeva & Sen, 1998
Eulipotyphla
*Amphechinus taatsiingolensis* Ziegler et al., 2007
Rodentia
*Proansomys badamae* sp. nov. Maridet et al. (2017, this issue)
*Cyclomylus intermedius* Wang, 2001
*Heosminthus borrae* Daxner-Höck et al., 2014
*Bohlinosminthus parvulus* (Bohlin, 1946)
*Eocricetodon* cf. *meridionalis* (Wang & Meng, 1986)
*Tachyoryctoides radnai* Daxner-Höck et al., 2015

The boundary between the Hsanda Gol and Loh Fms. is marked by a second dark red-brown to chocolate-brown clay (sediment layer—TGR-C/20). The uppermost part of the section is dominated by light-coloured sand and gravel layers of the Loh Fm.; on top, gravels of the Tuyn Gol Fm. and/or Pleistocene gravels (TGR-C/21-26) are exposed.

According to magnetostratigraphic investigations (Sun and Windley 2015), the red clay-caliche sequences (layers—TGR-C/1-13) correspond with the palaeomagnetic polarity Chrons C9n–C8n.1n, with an age range of 27.4–25.2 Ma. The whitish clay and caliche sequence up to the chocolate brown clay (layers—TGR-C/14-20) below the sand-gravel sequence of the Loh Fm. correspond with Chrons C7Ar–C7n.2n (age 25.2–24.2 Ma). Our section TGR-C was described as section B by Sun and Windley (2015). The correlation of Mongolian letter zones and magnetostratigraphic data is discussed below. The data from section TGR-C confirm the hitherto estimated age range of ∼28–25.6 Ma of letter zone C (Daxner-Höck et al. 2015).

### **Locality Taatsiin Gol (right side of the river Taatsiin; western plateau)**

#### Sections: TGR-B, TGR-B′, TGR-AB, TGR-A; samples: TGR-B/1, TGR-AB/22, TGR-AB/21, TGR-A/13+14, TGR-ZO/1, and TGR-ZO/2

**Fig. 14:**
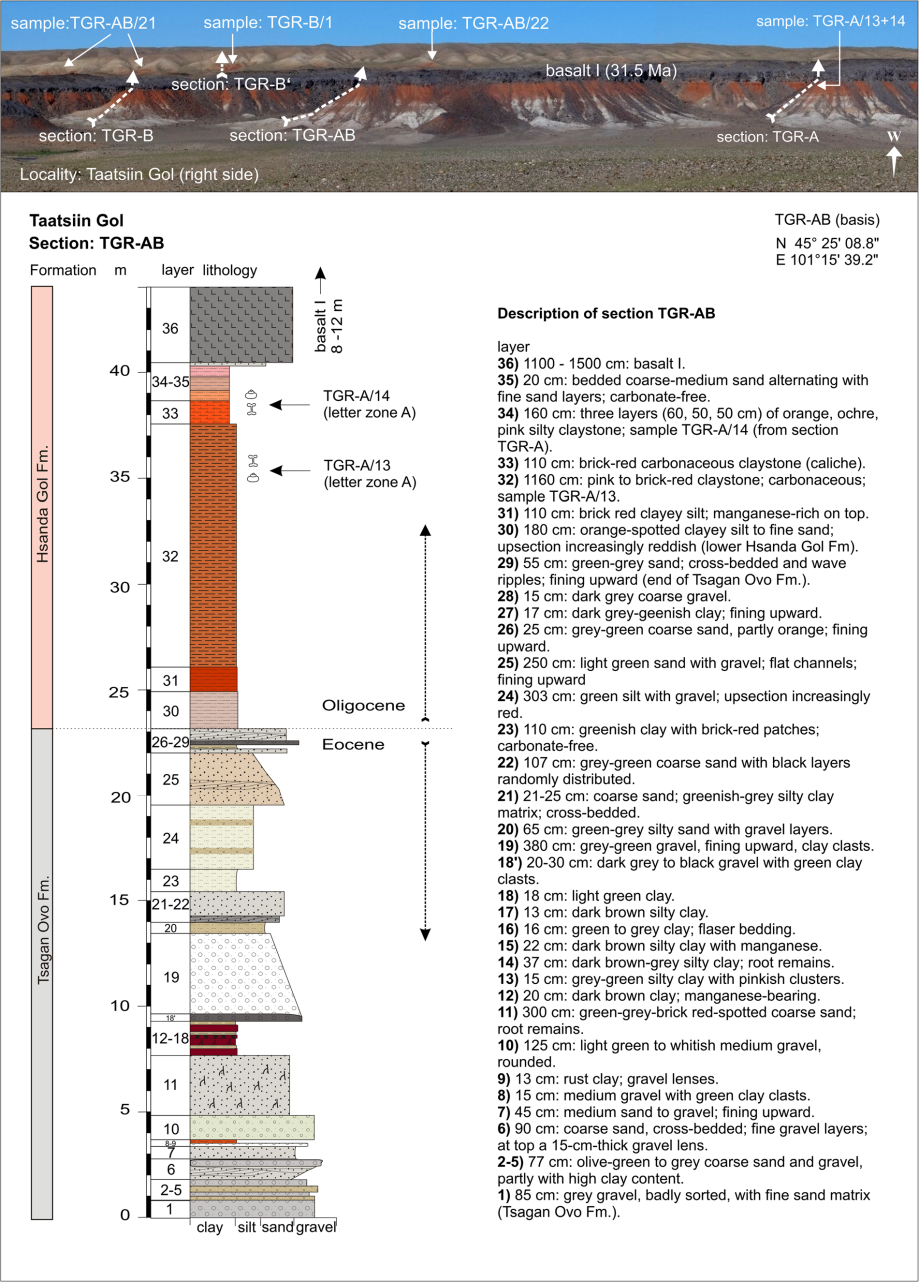
The eastern rim of Taatsiin plateau exposes the sections TGR-B+B′, TGR-AB, TGR-A, and basalt I (∼31.5 Ma) along of the orographic right side of Taatsiin Gol. From *bottom* to *top*, the sections display strata of the Tsagan Ovo, Hsanda Gol, Loh, and Tuyn Gol Fms

**Fig. 15:**
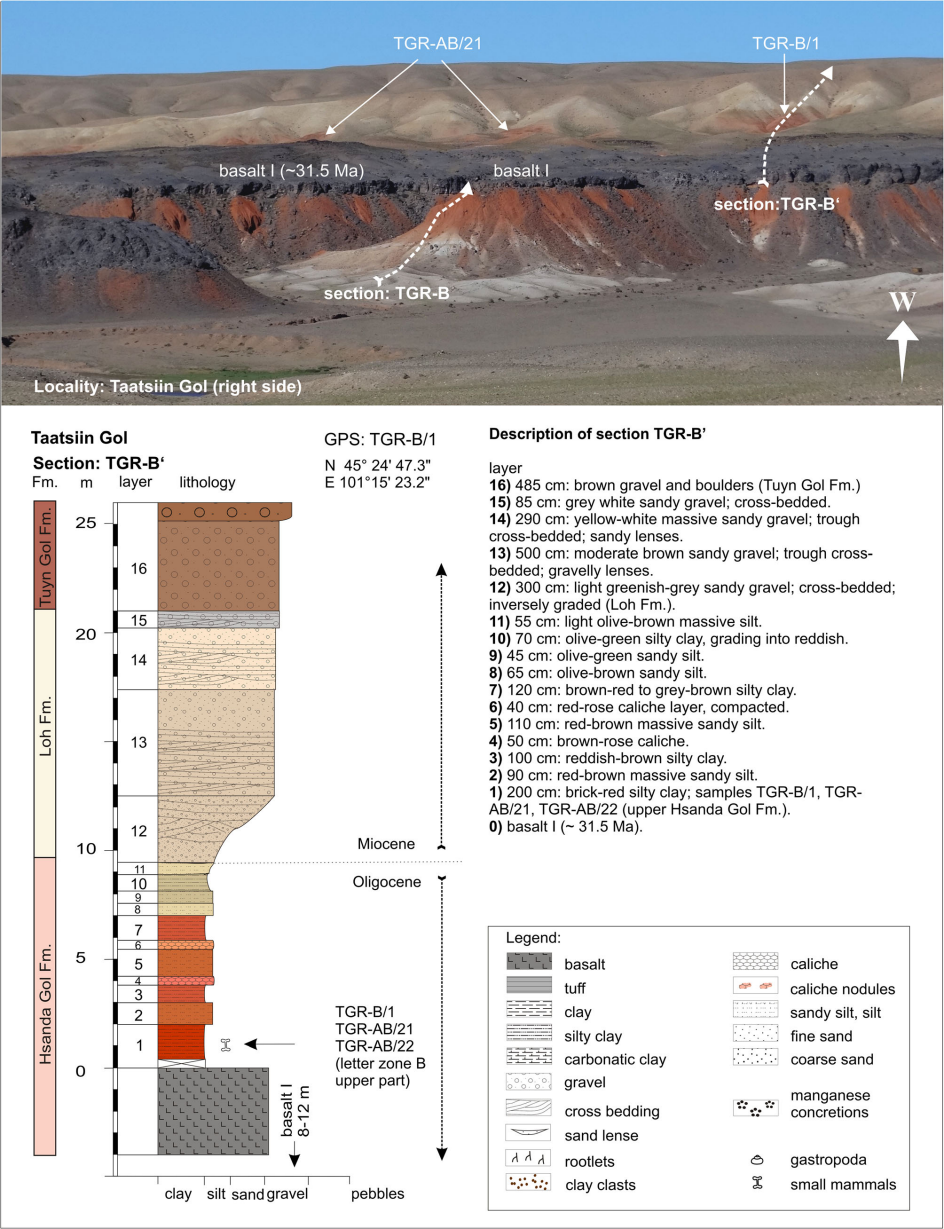
The section TGR-B and B′ is shown in detail. It displays the light-coloured sediments of the Tsagan Ovo Fm., the lower red beds of the Hsanda Gol Fm., which are overlain by basalt I. Above basalt I, the upper Hsanda Gol Fm. is locally visible as red exposures (TGR-AB/21, TGR-B/1). On *top*, sequences of the Loh and Tuyn Gol Fms. in light brownish colours. Description of section TGR-B modified from Schmid (1999, Abb. A1)

Along of the east rim of the Taatsiin plateau (orographic right side of the river Taatsiin), the sections TGR-A, TGR-B, and TGR-AB are exposed (Fig. 3k–m). There, four lithological units are visible: the Tsagan Ovo, Hsanda Gol, Loh, and Tuyn Gol Fms. In its lower part, the sections cover fluvio-lacustrine deposits of the Tsagan Ovo Fm. Upsection, and the brick-red clay of Hsanda Gol Fm. is topped by basalt I of early Oligocene age (^40^Ar/^39^Ar age, ∼31.5 Ma). The fossil beds (TGR-A/13+14), located immediately below basalt I, comprise key fossils of letter zone A. Above basalt I, 7 m of upper Hsanda Gol beds follow. The samples TGR-B/1, TGR-AB/21, and TGR-AB/22 from above basalt I yield fossils of letter zone B. Upsection, light-coloured sand and gravels of the Loh Fm. follow; on top, brown gavels and boulders of the Tuyn Gol Fm. are exposed (Figs. 14 and 15 and Höck et al. 1999; Fig. 6a).

Samples TGR-ZO/1 and TGR-ZO/2 were taken from red beds between two individual lava flows of basalt I at the east rim of the Taatsiin plateau (Fig. 3j). The mammal assemblages and basalt I indicate an early Oligocene age (Table 10).

**Table 10:** Fossils from Taatsiin Gol right side (sections—TGR-A and TGR-B, samples—TGR-A/13+14, TGR-ZO/1+2, TGR-B/1, TGR-AB/21, TGR-AB/22) [the age of the assemblages is early Oligocene (letter zones A and B)]

Taatsiin Gol (right side)	TGR-A/13+14	TGR-ZO/1+2	TGR-B/1	TGR-AB/21	TGR-AB/22
Biozone	A	B	B	B	B
Gastropoda					
*Pupoides steklovi* Prysjazhnjuk et al., 1975	x				
*Vallonia* cf. *lepida* (Reuss, 1849)	x				
*Vallonia stworzewiczae* Neubauer et al., 2013	x				
*Vallonia tumida* Stworzewicz, 2007	x				
*Gastrocopta devjatkini* Prysjazhnjuk et al., 1975	x				
*Gastrocopta* cf. *mongolica* Prysjazhnjuk et al., 1975	x				
*Gastrocopta shandgolica* Prysjazhnjuk, 1975	x				
Reptilia					
*Tinosaurus* sp.	x				
*Lacerta* sp. 1	x	x			
*Lacerta* sp. 2 + 3		x			
Scincomorpha indet.	x				
Squamata indet.		x			
Mammalia					
Lagomorpha					
*Desmatolagus youngi* (Gureev, 1960)					x
*Desmatolagus gobiensis* Matthew & Granger, 1923	x		x	x	x
*Desmatolagus robustus* Matthew & Granger, 1923				x	
*Desmatolagus* cf. *chinensis* Erbajeva & Sen, 1998				x	
*Desmatolagus* cf. *orlovi* (Gureev, 1960)			x	x	
*Desmatolagus* sp.	x				
Marsupialia					
*Asiadelphis zaissanensis* Gabunia et al., 1990			x	x	x
Eulipotyphla					
*Zaraalestes minutus* (Matthew & Granger, 1924a)	x		x	x	x
*Zaraalestes* sp.				x	
*Palaeoscaptor acridens* Matthew & Granger, 1924a	x		x	x	x
*Palaeoscaptor tenuis* Ziegler et al., 2007	x			x	x
*Gobisorex kingae* Sulimski, 1970	x		x		x
*Taatsiinia hoeckorum* Ziegler et al., 2007			x		
Crocidosoricidae indet.				x	x
Heterosoricinae indet.				x	
Talpidae indet.				x	
Rodentia					
*Promeniscomys* cf. *sinensis* Wang 1987			x		
*Ninamys kazimierzi* Vianey-Liaud et al., 2013	x		x	x	
*Ninamys arboraptus* (Shevyreva, 1966)			x	x	
*Eomys* aff. *orientalis* Wang & Emry, 1991			x	x	x
*Eomys* sp.				x	
*Karakoromys decessus* Matthew & Granger, 1923	x				
*Huangomys frequens* Schmidt-Kittler et al. 2007			x	x	x
*Yindirtemys shevyrevae* Vianey-Liaud et al., 2006			x	x	x
*Tataromys sigmodon* Matthew & Granger, 1923					x
*Anomoemys lohiculus* (Matthew & Granger, 1923)	x				
Tsaganomyidae indet.			x		
*Cyclomylus lohensis* Matthew & Granger, 1923					x
*Cyclomylus intermedius* Wang, 2001					x
*Coelodontomys asiaticus* Wang, 2001			x		
*Tsaganomys altaicus* Matthew & Granger, 1923			x	x	x
*Allosminthus khandae* (Daxner-Höck, 2001)	x				
*Allosminthus minutus* (Daxner-Höck, 2001)			x	x	
*Heosminthus chimidae* Daxner-Höck et al., 2014	x		x	x	x
*Heosminthus* sp.	x		x		
*Heosminthus borrae* Daxner-Höck et al., 2014			x	x	x
*Onjosminthus baindi* Daxner-Höck et al., 2014			x	x	x
*Shamosminthus sodovis* Daxner-Höck, 2001	x		x	x	x
*Ulaancricetodon badamae* Daxner-Höck, 2000			x	x	x
*Selenomys mimicus* Matthew & Granger, 1923	x				
*Cricetops dormitor* Matthew & Granger, 1923	x		x	x	x
*Eocricetodon meridionalis* (Wang & Meng, 1986)	x	x	x		x
*Eucricetodon caducus* (Shevyreva, 1967)	x	x	x	x	x
*Eucricetodon asiaticus* Matthew & Granger, 1923	x		x	x	x
*Eucricetodon occasionalis* Lopatin, 1996					x
*Eucricetodon jilantaiensis* Gomes Rodrigues et al., 2012				x	
*Paracricetodon* sp.	x				
Carnivora					
*Amphicticeps shackelfordi* Matthew & Granger, 1924b			x		
*Palaeogale* sp.				x	x
Carnivora indet.				x	
Leptictida					
cf. *Ergilictis* sp.					
Ruminantia					
*Praetragulus gobiae* (Matthew & Granger, 1925b)			x		
*Miomeryx* sp.				x	x
*Gobimeryx* sp.					x
*Pseudogelocus mongolicus* Vislobokova & Daxner-Höck, 2002					x
*Pseudomeryx* sp.			x		x
*Eumeryx* sp.			x		

Magnetostratigraphic investigations of the Tsagan Ovo Fm. and Hsanda Gol Fm., including basalt I, have been performed along a comparable section, which was named section A by Sun and Windley (2015). The strata above basalt I, containing the upper Hsanda Gol beds and the Loh Fm., were not considered in the magnetostratigraphic investigations.

From bottom to top, the sequences of the Tsagan Ovo Fm. correspond with Chrons C15r–C13r (>35–34 Ma/late Eocene). Thus, the boundary between the Tsagan Ovo Fm. and Hsanda Gol Fms. corresponds with the Eocene-Oligocene boundary. The lower Hsanda Gol strata and basalt I correspond with the palaeomagnetic polarity Chrons C13r–C12r, with an age range of ∼34–31.2 Ma (Kraatz and Geisler 2010; Sun and Windley 2015), which is an early Oligocene age. These data agree with the ^40^Ar/^39^Ar ages measured from several samples of basalt I in the Taatsiin Gol region (Tables 1 and 2).

### **Locality Taatsiin Gol (left side of the river; eastern plateau)**

#### Sections: TGL-A+A′; samples: TGL-A/1+2, TGL-A/11

**Fig. 16:**
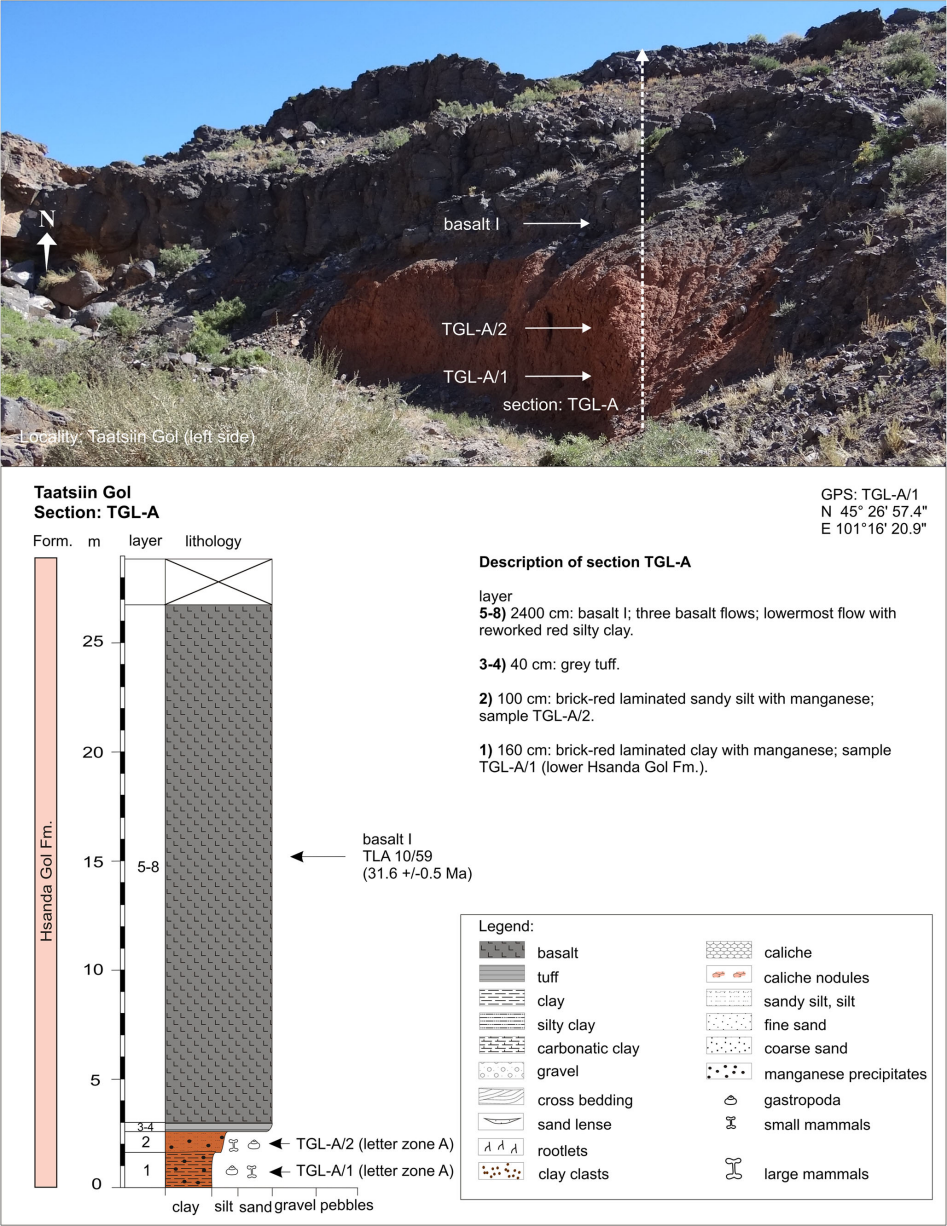
Section TGL-A is located at the orographic left side of Taatsiin Gol (eastern plateau). The lower part of section TGL-A comprises the lower Hsanda Gol beds and basalt I (31.6 ± 0.5 Ma). Description of section TGL-A after Daxner-Höck et al. (1997; Fig. 2)

**Fig. 17:**
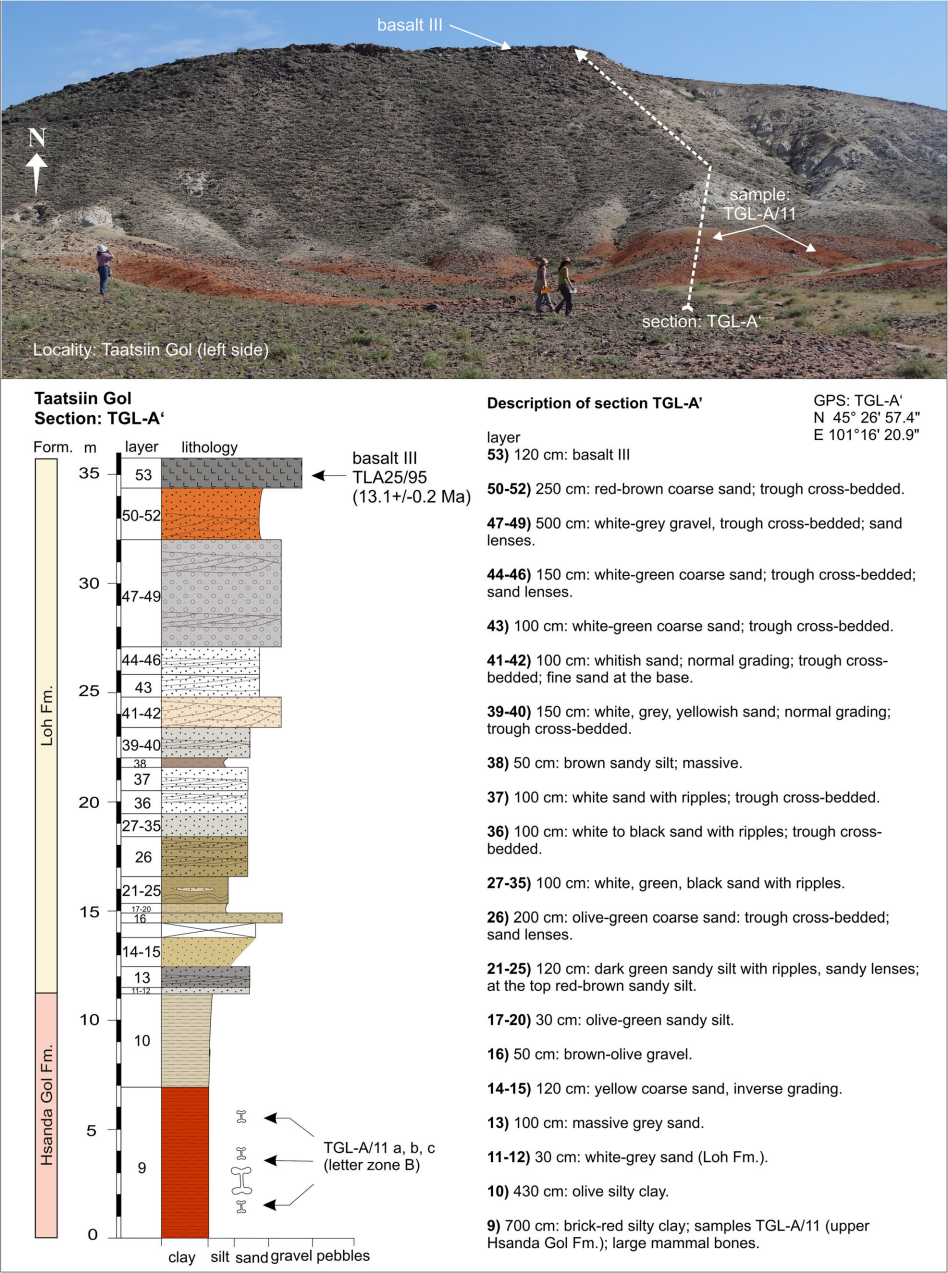
Above the Oligocene basalt I, the upper Hsanda Gol beds (sample TGL-A/11) are located below light-coloured sand and gravel beds of the Loh Fm. On *top*, the middle Miocene basalt III (13.1 ± 0.2 Ma). Description of section TGL-A after Daxner-Höck et al. (1997; Fig. 2)

Section TGL-A from the orographic left side of Taatsiin Gol (Fig. 3n) comprises the lower Hsanda Gol beds with fossils of letter zone A (samples TGL-A/1+2) and basalt I (31.6 Ma; Fig. 16). Above basalt I, section TGL-A′ displays the upper Hsanda Gol beds with fossils of letter zone B (sample TGL-A/11; Fig. 17) and a 25-m-thick sequence of the Loh Fm. The middle Miocene basalt III (13.1 Ma) forms the top of the plateau. Samples below basalt I (TGL-A/1+2) yield small mammal fossils and land gastropods (Table 11). The early Oligocene age and letter zone A are indicated by basalt I and the included fossils.

**Table 11:** Fossils from Taatsiin Gol left side (section—TGL-A, samples—TGL-A/1+2, TGL-A/11) [the age of the assemblages is early Oligocene (letter zones A and B)]

Taatsiin Gol (left side)	TGL-A/1+2	TGL-A/11
Letter zone	A	B
Gastropoda		
*Pupoides steklovi* Prysjazhnjuk et al., 1975		
*Vallonia stworzewiczae* Neubauer et al., 2013		
*Vallonia tumida* Stworzewicz, 2007		
Reptilia		
*Lacerta* 1.	x	x
Squamata indet.	x	
Mammalia		
Lagomorpha		
*Desmatolagus gobiensis* Matthew & Granger, 1923	x	x
*Desmatolagus* cf. *vetustus* Burke, 1941	x	
*Desmatolagus* sp.		x
Eulipotyphla		
*Zaraalestes minutus* (Matthew & Granger, 1924a)	x	x
*Palaeoscaptor acridens* Matthew & Granger, 1924a	x	x
*Palaeoscaptor tenuis* Ziegler et al., 2007	x	x
*Mongolopala tathue* Ziegler et al., 2007	x	
Rodentia		
*Ninamys kazimierzi* Vianey-Liaud et al., 2013	x	x
*Ninamys arboraptus* (Shevyreva, 1966		x
*Karakoromys decessus* Matthew & Granger, 1923	x	
*Ardynomys* sp.	x	
*Anomoemys lohiculus* (Matthew & Granger, 1923)		x
*Tsaganomyidae* indet.	x	
*Cyclomylus lohensis* Matthew & Granger, 1923		x
*Cyclomylus intermedius* Wang, 2001		x
*Tsaganomys altaicus* Matthew & Granger, 1923		x
*Heosminthus chimidae* Daxner-Höck et al., 2014	x	x
*Shamosminthus sodovis* Daxner-Höck, 2001	x	x
*Selenomys mimicus* Matthew & Granger, 1923		x
*Cricetops dormitor* Matthew & Granger, 1923		x
*Ulaancricetodon badamae* Daxner-Höck, 2000	x	x
*Eucricetodon caducus* (Shevyreva, 1967)	x	x
*Eucricetodon asiaticus* Matthew & Granger, 1923	x	x
Creodonta		
Hyaenodontidae indet.		x
Carnivora		
*Asiavorator altidens* Spassov & Lange-Badré, 1995		x
Ruminantia		
*Pseudogelocus mongolicus* Vislobokova & Daxner-Höck, 2002		x
Ruminantia indet.		x

### **Locality Unzing Churum**

#### Section: TAR-A; sample: TAR-A/2

**Fig. 18:**
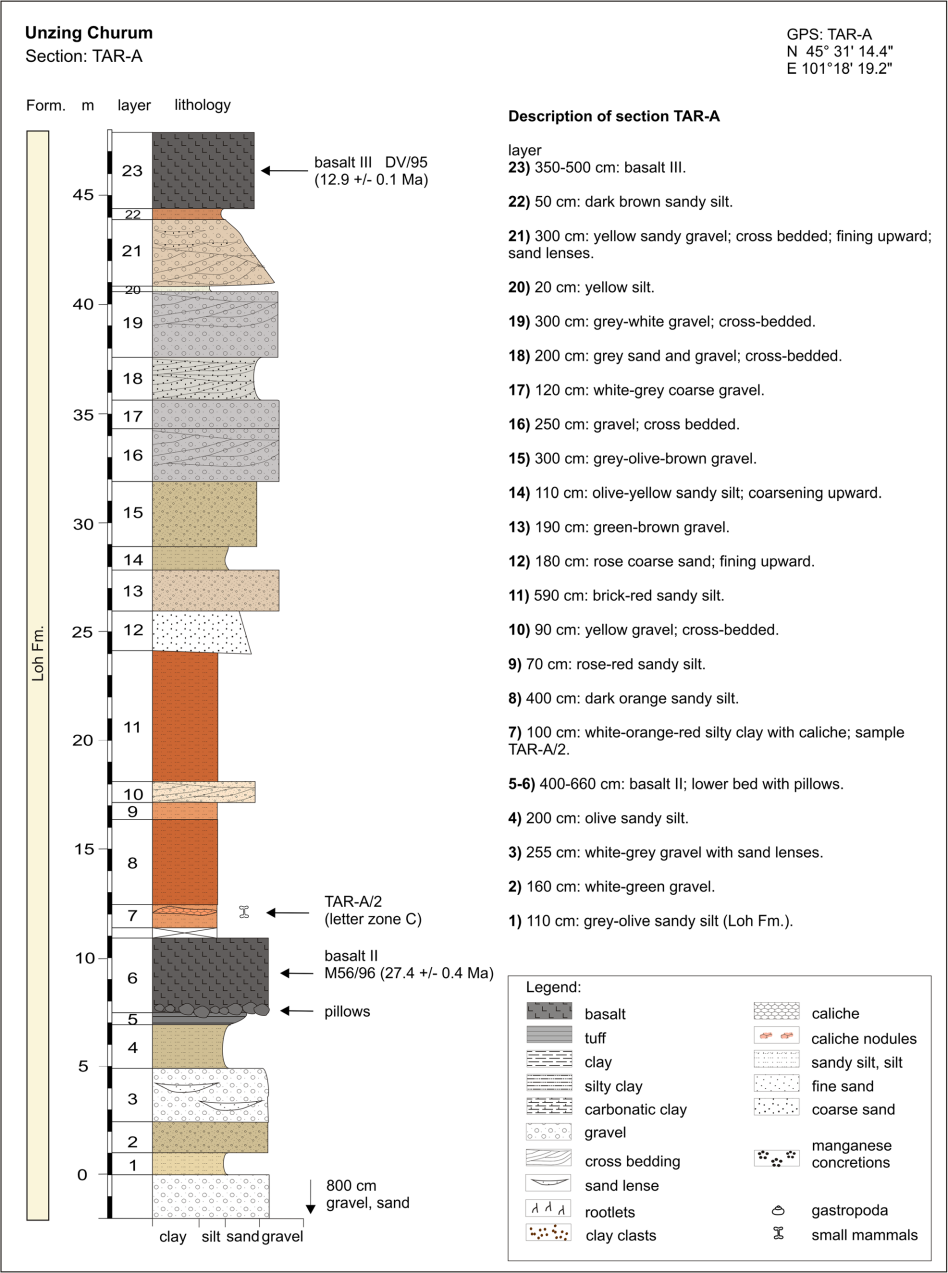
Section TAR-A is located north-east of Taatsiin Gol. The section comprises sequences of the Loh Fm. including two basalt layers, basalt II and basalt III. Description of section TAR-A modified from Schmid (1999; Abb. A4)

**Fig. 19:**
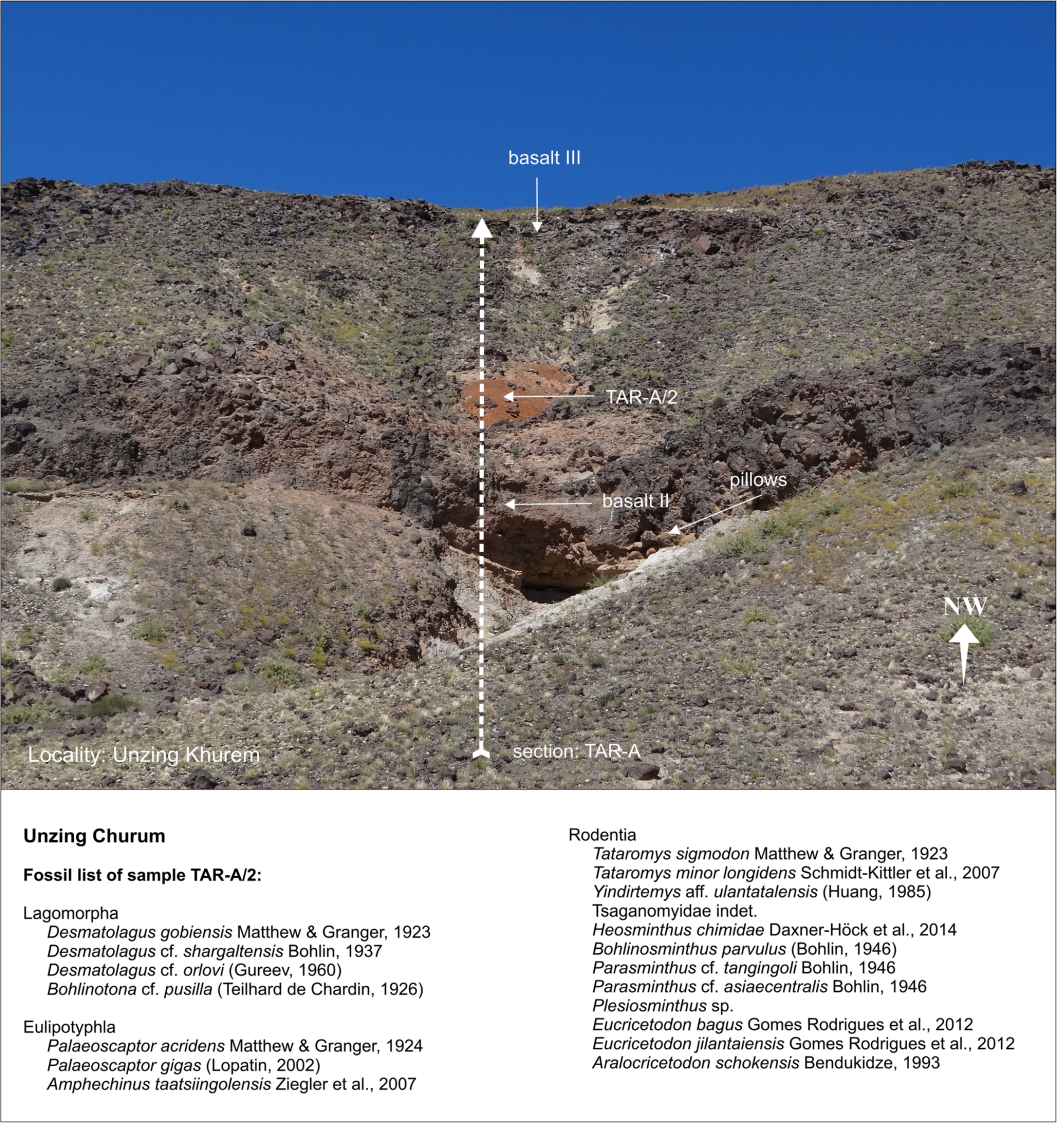
Section TAR-A from the locality Unzing Churum. Sample TAR-A/2 on top of basalt II yields fossils of letter zone C

Unzing Churum is located north-east of Taatsiin Gol (Fig. 3o). The lower part of section TAR-A consists of light-coloured fluvial sand and gravel deposits, which are overlain by basalt II. The ^40^Ar/^39^Ar age of basalt II is 27.4 ± 0.4 Ma (Höck et al. 1999 and Tables 1 and 2). Above basalt II, 8 m of brick-red sandy silt follow. Sample TAR-A/2 from the white-orange-red silty clay yields fossils of letter zone C (Figs. 18 and 19). Upsection, fluvial deposits follow. These include sand and silt layers and partly cross-bedded gravels. The section is topped by the middle Miocene basalt III, dated at 12.9 ± 0.1 Ma.

For palaeoenvironmental considerations, it is worth to note that pillow structures were formed locally where basalt II flowed into a shallow pond or lake (Höck et al. 1999; Fig. 10b).

### **Locality Del**

#### Section: Del-B; samples: DEL-B/2, DEL-B/7+8, DEL-B/12

**Fig. 20:**
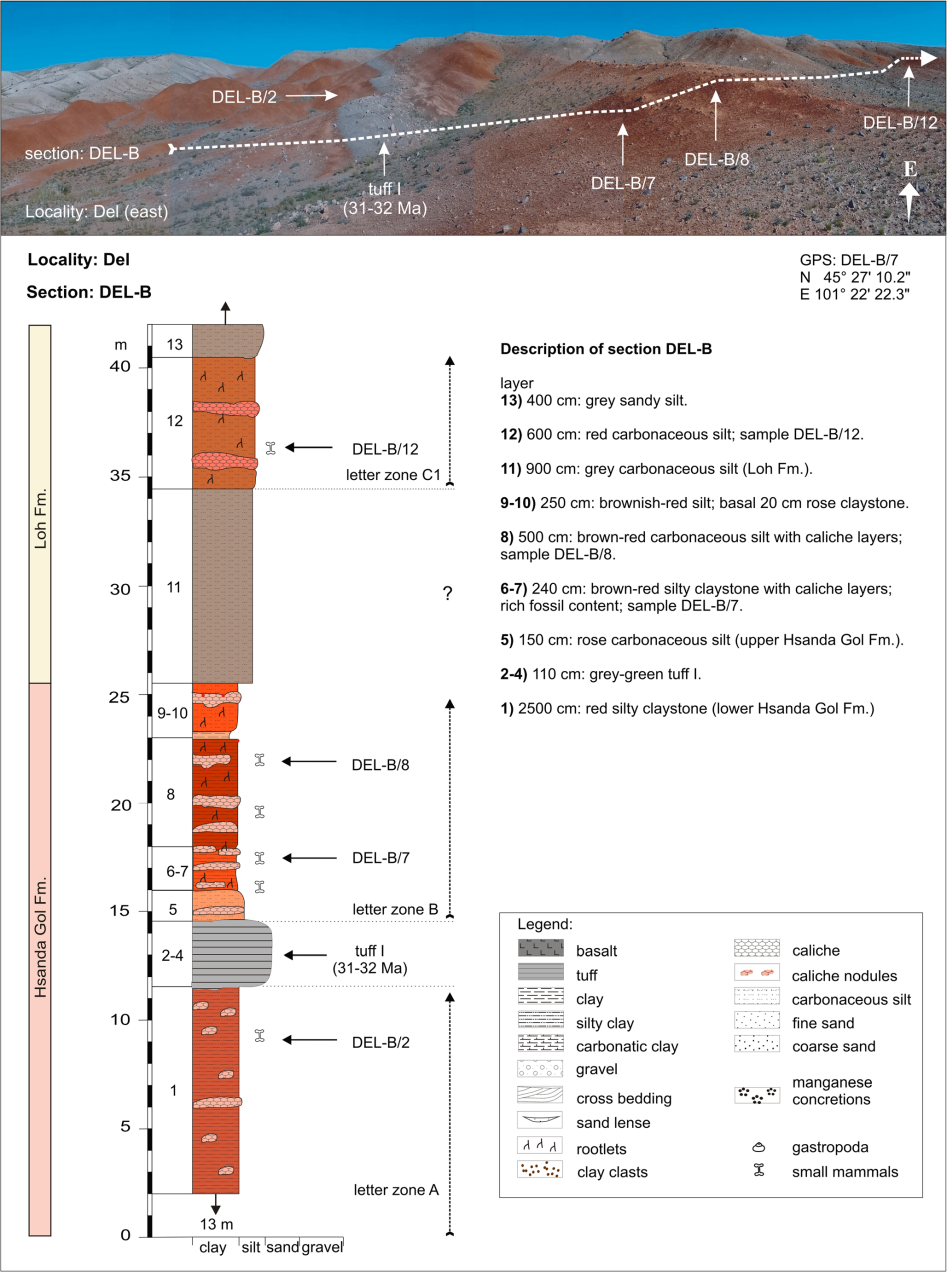
The Del section is located at the Del fault, close to the boundary between Mesozoic and Paleogene strata in the northern part of the Taatsiin Tsagaan Nuur basin. From the *left* to the *right* side of the picture, the Tsagan Ovo Fm. (in whitish-grey colours), the lower Hsanda Gol beds (red-brown), tuff I (gey), and the upper Hsanda Gol beds (grey-brown to red) are visible

The locality Del is located in the northern part of the Taatsiin Tsaagan Nuur basin (Fig. 3p). The direction of section DEL-B is N → S. From north to south, strata of the Tsagan Ovo, Hsanda Gol, and Loh Fms. are affected by the Del fault and are tilted towards south. The Hsanda Gol beds are divided by a tuff layer several metres in thickness (tuff I; Fig. 20). The lower Hsanda Gol beds yield very rare fossils of letter zone A (sample DEL-B/2). Above tuff I, several caliche layers are imbedded in the upper Hsanda Gol beds. The abundant fossils from these caliche layers (samples DEL-B/7 and DEL-B/8) indicate letter zone B and the early Oligocene. The following grey-brown silt of the Loh Fm. lacks fossils. Upsection, sample DEL-B/12 from a red silt layer yields fossils of letter zone C1 (*Yindirtemys deflexus*), pointing to the late Oligocene (Table 12).

**Table 12:** Fossils from the locality Del (section—DEL-B, samples—DEL-B/7+8 and DEL-B/12) [the age of assemblages DEL-B/7+8 is early Oligocene (letter zone B) and of assemblage DEL-B/12 is late Oligocene (letter zone C1)]

DEL-B/7+8	DEL-B/12
Lagomorpha	Lagomorpha
*Ordolagus* cf. *teilhardi* (Burke, 1941	*Ordolagus* cf. *teilhardi* (Burke, 1941)
*Desmatolagus youngi* (Gureev, 1960)	*Desmatolagus* cf. *simplex* (Argyropulo, 1940)
*Desmatolagus gobiensis* Matthew & Granger, 1923	*Desmatolagus* cf. *chinensis* Erbajeva & Sen, 1998
*Desmatolagus robustus* Matthew & Granger, 1923	*Bohlinotona* cf. *pusilla* (Teilhard de Chardin, 1926)
*Desmatolagus* cf. *simplex* (Argyropulo, 1940)	*Sinolagomys kansuensis* Bohlin, 1937
*Desmatolagus* cf. *orlovi* (Gureev, 1960)	Eulipotyphla
*Desmatolagus* sp.	*Palaeoscaptor acridens* Matthew & Granger, 1924a
Eulipotyphla	*Palaeoscaptor* cf. *rectus* Matthew & Granger, 1924a
*Zaraalestes minutus* (Matthew & Granger, 1924a)	*Amphechinus minutissimus* Ziegler et al., 2007
*Zaraalestes* sp.	*Amphechinus major* Ziegler et al., 2007
*Palaeoscaptor acridens* Matthew & Granger, 1924a	Crocidosoricinae indet.
*Gobisorex kingae* Sulimski, 1970	Rodentia
Rodentia	*Asianeomys dangheensis* (Wang, 2002)
*Ninamys kazimierzi* Vianey-Liaud et al., 2013	*Tatataromys minor* longidens Schmidt-Kittler et al., 2007
*Anomoemys lohiculus* (Matthew & Granger, 1923)	*Tataromys plicidens* Matthew & Granger, 1923
*Cyclomylus lohensis* Matthew & Granger, 1923	*Yindirtemys deflexus* (Teilhard de Chardin, 1926)
*Cyclomylus intermedius* Wang, 2001	*Tsaganomys altaicus* Matthew & Granger, 1923
Tsaganomyidae indet.	*Bohlinosminthus parvulus* (Bohlin, 1946)
*Tsaganomys altaicus* Matthew & Granger, 1923	*Parasminthus* cf. *tangingoli* Bohlin, 1946
*Heosminthus chimidae* Daxner-Höck et al., 2014	*Parasminthus debruijni* Lopatin, 1999
*Heosminthus* sp.	*Parasminthus* cf. *asiaecentralis* Bohlin, 1946
*Shamosminthus sodovis* Daxner-Höck, 2001	*Eucricetodon bagus* Gomes Rodrigues et al., 2012
*Ulaancricetodon badamae* Daxner-Höck, 2000	*Bagacricetodon tongi* Gomes Rodrigues et al., 2012
*Cricetops dormitor* Matthew & Granger, 1923	*Aralocricetodon schokensis* Bendukidze, 1993
*Eocricetodon meridionalis* (Wang & Meng, 1986)	Creodonta
*Eucricetodon caducus* (Shevyreva, 1967)	Hyaenodontidae indet.
Ruminantia	Carnivora
Ruminantia indet.	*Amphicticeps shackelfordi* Matthew & Granger, 1924b
	Ruminantia
	Bovidae gen.2
	*Palaeohypsodontus* sp.

### **Locality Tatal Gol**

**Fig. 21:**
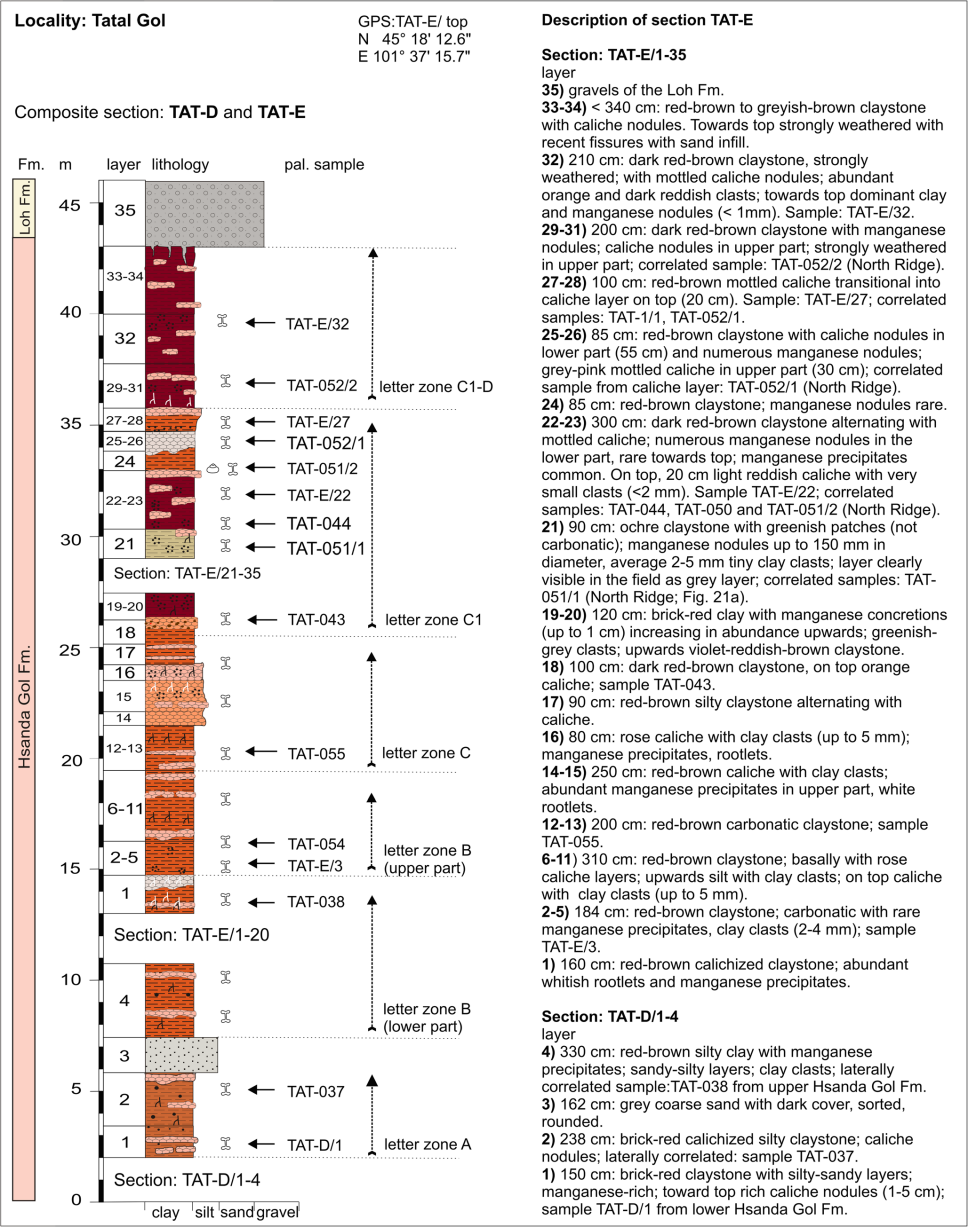
The Tatal Gol section at the west side of the Tatal creek is composed of three parts, the lower part TAT-D/1-4, the middle part TAT-E/1-20, and the upper part TAT-E/21-35 at the “North Ridge.” From south to north, the section comprises the complete sequence of Oligocene sediments

**Fig. 22:**
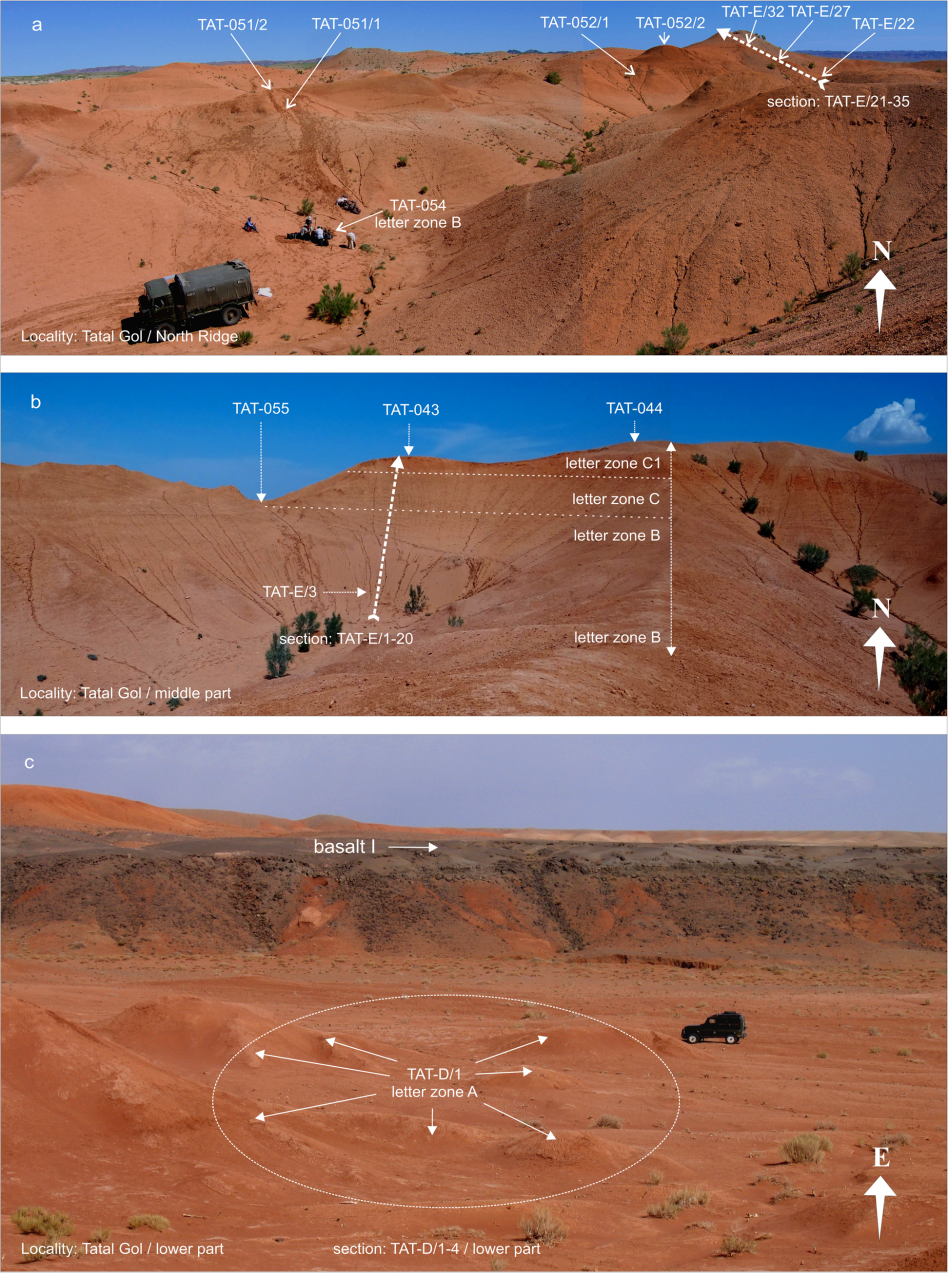
Locality Tatal Gol. **a** Tatal Gol North Ridge shows sample points TAT-051/1-2, TAT-052/1-2, TAT-054, and the upper part of section TAT-E/21-35. **b** Tatal section Gol middle part shows section TAT-E/1-20 and fossil points TAT-055, TAT-043, TAT-044, and TAT-E/3. **c** Tatal Gol lower part showing the flat area from where several bulk samples TAT-D/1 were screen washed

**Fig. 23:**
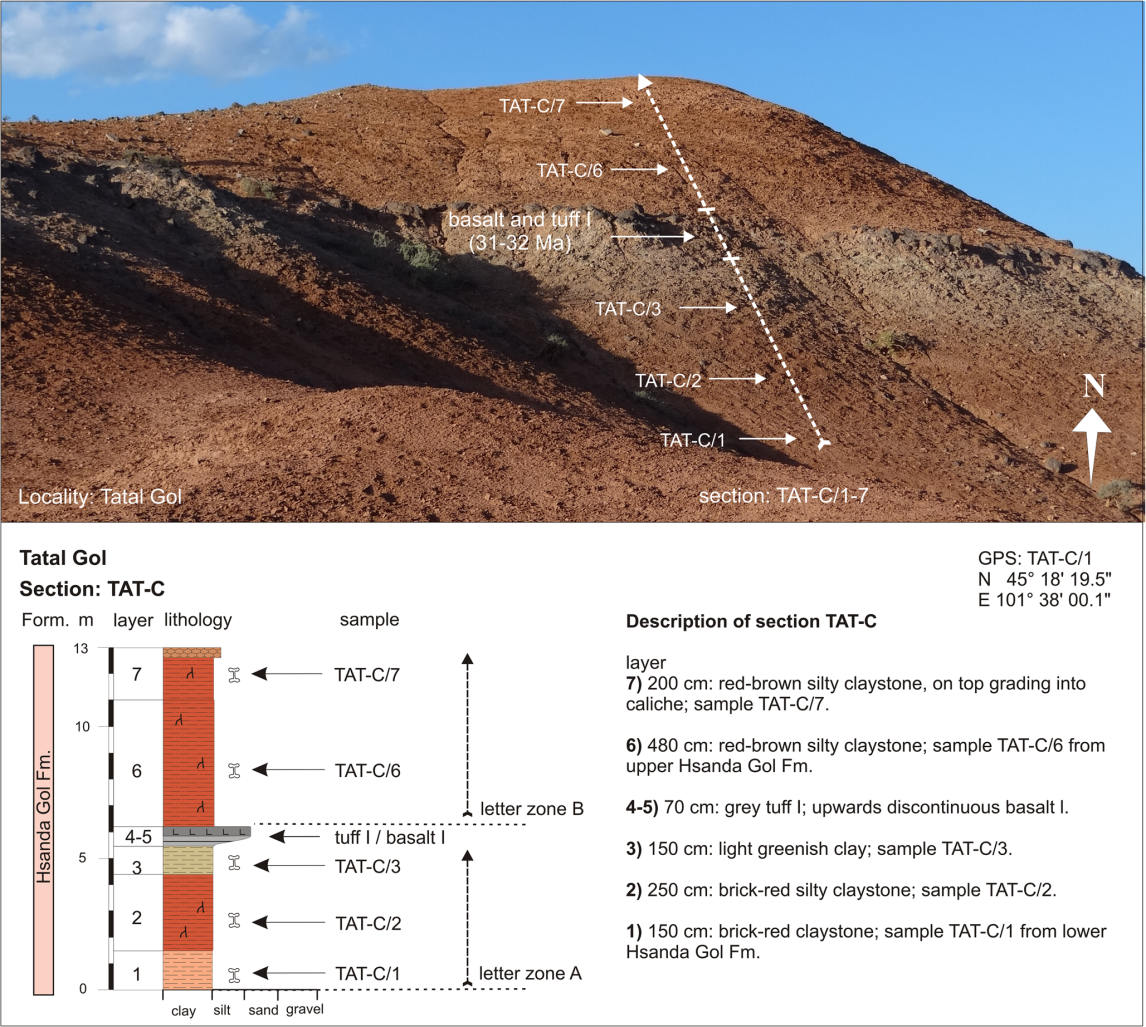
Section TAT-C is located east side of the Tatal creek in the north-eastern part of the locality

For localization, see Fig. 3q, r. In Tatal Gol, two sections were studied, the composite section TAT-D+E (Figs. 21 and 22, Tables 13 and 14) and section TAT-C (Fig. 23, Table 16).

Section TAT-D+E (Fig. 21) is located west of the dry creek, called Tatal Gol. The section is composed of three parts: the lower part TAT-D/1-4 (Fig. 22c), the middle part TAT-E/1-20 (Fig. 22b), and the upper part at the “North Ridge” is TAT-E/21-32 (Fig. 22a). In section TAT-C (Fig. 23, Table 16), which is located east of the Tatal creek, the Hsanda Gol Fm. displays the lower Hsanda Gol beds, tuff and basalt I, and the upper Hsanda Gol beds.

#### Section TAT-D+E; samples: TAT-D/1, TAT-E/3, TAT-E/22, TAT-E/27, TAT-E/32; TAT-037, TAT-038-, TAT-054, TAT-055, TAT-043, TAT-044, TAT-051/1-2, TAT-052/1-2

In this section, the Hsanda Gol Fm. comprises fossils of letter zones A, B, C, C1, and C1-D, showing that the Hsanda Gol Fm. ranges from the early Oligocene to the Oligocene/Miocene transition. Although basalt I is missing in the western part of Tatal Gol, the lower and upper Hsanda Gol beds are easily recognisable by the included fossils.

The lower (= southern) part, section TAT-D, is composed of brick-red silty claystone, which yields abundant caliche concretions and a mammal assemblage that is very rich in fossils of letter zone A (sample TAT-D/1; Table 13). A layer of coarse grey sand follows, which is topped by the brick-red clay of the Hsanda Gol Fm., yielding fossils of letter zone B. In the middle part (TAT-E/1-11), the upper Hsanda Gol Fm. comprises fossils of letter zone B (sample TAT-E/3; Table 14). Upsection, fossils of letter zone C (sample TAT-055; Table 14) were recovered from a reddish carbonatic claystone below a 3-m-thick red-rose caliche. On top, the colour of caliche changes to orange-red with dark brown clay clast inclusions. The fossils from the orange caliche and the overlying brick-red to dark-brown clay indicate letter zone C1. These fossil assemblages of letter zone C1 are characteristic of the higher part of the section (samples TAT-043 to TAT-E/27; Table 14) and of sample points laterally of the main section. Finally, fossils of letter zone C1-D were found in the dark brown clay layers close to the top of the section at the North Ridge (samples TAT-052/2 and TAT-E/32) (Figs. 21 and 22, Table 14), which are easily recognisable by the included fossils.

**Table 13:** Fossils from the locality Tatal Gol (section—TAT-D, sample—TAT-D/1) [the age of assemblage is early Oligocene (letter zone A)]

Tatal Gol: TAT-D/1
Lagomorpha
*Ordolagus* cf. *teilhardi* (Burke, 1941)
*Desmatolagus* cf. *vetustus* Burke, 1941
*Desmatolagus youngi* (Gureev, 1960)
*Desmatolagus gobiensis* Matthew & Granger, 1923
*Desmatolagus* cf. *chinensis* Erbajeva & Sen, 1998
*Desmatolagus* cf. *orlovi* (Gureev, 1960)
*Desmatolagus* sp.
Marsupialia
*Asiadelphis tjutkovae* Emry et al., 1995
Eulipotyphla
*Zaraalestes minutus* (Matthew & Granger, 1924a)
*Palaeoscaptor acridens* Matthew & Granger, 1924a
*Palaeoscaptor* cf. *rectus* Matthew & Granger, 1924a
*Palaeoscaptor tenuis* Ziegler et al., 2007
Erinaceidae indet.
*Gobisorex kingae* Sulimski, 1970
cf. *Asiapternodus mackennai* Lopatin, 2003
*Mongolopala tathue* Ziegler et al., 2007
Talpidae indet.
Rodentia
*Ninamys kazimierzi* Vianey-Liaud et al., 2013
*Prosciurus* ? *mongoliensis* Wang & Dashzeveg, 2005
*Prosciurus* ? nov. spec.
*Promeniscomys* cf. *sinensis* Wang, 1987a
*Karakoromys decessus* Matthew & Granger, 1923
*Ardynomys* sp.
*Cyclomylus lohensis* Matthew & Granger, 1923
*Cyclomylus biforatus* Wang, 2001
*Cyclomylus intermedius* Wang, 2001
*Coelodontomys asiaticus* Wang, 2001
*Tsaganomys altaicus* Matthew & Granger, 1923
*Allosminthus khandae* (Daxner-Höck, 2001)
*Heosminthus chimidae* Daxner-Höck et al., 2014
*Heosminthus* sp.
*Shamosminthus sodovis* Daxner-Höck, 2001
*Ulaancricetodon badamae* Daxner-Höck, 2000
*Selenomys mimicus* Matthew & Granger, 1923
*Cricetops dormitor* Matthew & Granger, 1923
*Cricetops minor* Wang, 1987b
*Eocricetodon meridionalis* (Wang & Meng, 1986)
*Eucrucetodon caducus* (Shevyreva, 1967)
*Eucricetodon asiaticus* Matthew & Granger, 1923
Creodonta
*Hyaenodon* cf. *incertus* Dashzeveg, 1985
Carnivora
*Amphicynodon teilhardi* (Matthew & Granger, 1924b)
aff. *Amphicynodon* sp.
*Amphicticeps shackelfordi* Matthew & Granger, 1924b
Leptictida
*Didymoconus colgatei* Matthew & Granger, 1924b
Artiodactyla
*Lophiomeryx angarae* Matthew & Granger, 1925b
*Lophiomeryx* sp.
*Praetragulus gobiae* (Matthew & Granger, 1925b)
*Miomeryx* sp.
*Gobimeryx dubius* Trofimov, 1957
*Pseudomeryx gobiensis* Trofimov, 1957
*Pseudomeryx* sp.
*Pseudogelocus mongolicus* Vislobokova & Daxner-Höck, 2002
Ruminantia indet

**Table 14:** Fossils from the locality Tatal Gol (section—TAT-E/1-32 and samples—Fig. 21a, b) [the ages of the assemblages are early Oligocene (letter zones A and B) to late Oligocene (letter zones C, C1, and C1-D)]

Tatal Gol Section: TAT-E	TAT-037	TAT-E/3	TAT-055	TAT-043	TAT-044	TAT-051/2	TAT-E/22	TAT-E/27	TAT-E/32
Letter zone	A	B	C	C1	C1	C1	C1	C1	C1-D
Gastropoda									
*Vallonia* sp.									
Lagomorpha									
*Desmatolagus gobiensis* Matthew & Granger, 1923			x		x				
*Desmatolagus* cf. *simplex* (Argyropulo, 1940	x		x		x	x			
*Desmatolagus* cf. *chinensis* Erbajeva & Sen, 1998	x								
*Desmatolagus* sp.	x	x		x	x			x	
*Bohlinotona* cf. *pusilla* (Teilhard de Chardin, 1926)			x						
*Sinolagomys kansuensis* Bohlin, 1937				x	x	x	x	x	x
*Sinolagomys major* Bohlin, 1937					x	x	x	x	x
*Sinolagomys ulungurensis* Tong, 1989									x
Eulipotyphla									
*Zaraalestes minutus* (Matthew & Granger, 1924a)	x	x	x			x			
*Palaeoscaptor acridens* Matthew & Granger, 1924a	x	x	x	x					
*Palaeoscaptor* cf. *rectus* Matthew & Granger, 1924a			x	x		x			
*Palaeoscaptor tenuis* Ziegler et al., 2007		x	x						
*Palaeoscaptor gigas* (Lopatin, 2002)						x		x	
*Amphechinus taatsiingolensis* Ziegler et al., 2007			x	x		x			x
*Amphechinus minutissimus* Ziegler et al., 2007				x		x	x	x	
*Amphechinus major* Ziegler et al., 2007				x		x	x	x	x
Erinaceidae indet.	x	x	x			x			
*Gobisorex kingae* Sulimski, 1970	x		x						
Rodentia									
*Promeniscomys sinensis* Wang 1987		x							
*Asianeomys dangheensis* (Wang, 2002)									x
*Karakoromys decessus* Matthew & Granger, 1923									
*Yindirtemys shevyrevae* Vianey-Liaud et al., 2006	x								
*Tataromys sigmodon* Matthew & Granger, 1923			x						
*Tatataromys minor longidens* Schmidt-Kittler et al., 2007			x						
*Tataromys plicidens* Matthew & Granger, 1923			x						
*Yindirtemys deflexus* (Teilhard de Chardin, 1926)				x	x	x	x	x	
*Yindirtemys birgeri* Bendukidze, 1993						x			
*Yindirtemys suni* Li & Qiu, 1980									x
*Ardynomys* sp.	x								
*Cyclomylus intermedius* Wang, 2001	x								
*Tsaganomys altaicus* Matthew & Granger, 1923			x	x	x	x			
*Allosminthus minutus* (Daxner-Höck, 2001)			x						
*Heosminthus chimidae* Daxner-Höck et al., 2014	x	x	x	x			x		
*Heosminthus borrae* Daxner-Höck et al., 2014			x					x	x
*Heosminthus* sp.			x	x					
*Onjosminthus baindi* Daxner-Höck et al., 2014	x								
*Shamosminthus sodovis* Daxner-Höck, 2001		x	x						
*Shamosminthus tongi* Huang, 1992			x						
*Bohlinosminthus parvulus* (Bohlin, 1946)				x		x	x	x	x
*Parasminthus* cf. *tangingoli* Bohlin, 1946				x					
*Parasminthus debruijni* Lopatin, 1999								x	
*Litodonomys huangheensis* Wang & Qiu, 2000									x
*Litodonomys lajeensis* (Li & Qiu, 1980)					x	x	x	x	x
*Litodonomys lajeensis* (Li & Qiu, 1980)							x	x	x
*Selenomys mimicus* Matthew & Granger, 1923	x								
*Cricetops dormitor* Matthew & Granger, 1923	x								
*Eocricetodon meridionalis* (Wang & Meng, 1986)	x							x	
*Eucricetodon caducus* (Shevyreva, 1967)	x	x							
*Eucricetodon asiaticus* Mathew & Granger, 1923		x							
*Eucricetodon bagus* Gomes Rodrigues et al., 2012		x						x	
*Tachyoryctoides radnai* Daxner-Höck et al., 2015			x						
*Tachyoryctoides obrutschewi* Bohlin, 1937						x			
*Tachyoryctoides tatalgolicus* Dashzeveg, 1971				x			x		
*Tachyoryctoides* sp.				x	x				*x*

#### Section: TAT-C; samples: TAT-C/1-3, TAT-C/6-7

From bottom to top, the section displays lower Hsanda Gol beds below basalt and tuff I (samples—TAT-C/1-3) and upper Hsanda Gol beds (samples—TAT-C/6-7) above basalt I (Fig. 23; Table 15).

**Table 15:** Fossil list from section TAT-C in Tatal Gol (the samples TAT-C/1-3 below basalt I yield fossils of letter zone A, the fossils from samples TAT-C/6-7 above basalt I indicate letter zone B)

Tatal Gol Section: TAT-C	TAT-C/1-3	TAT-C/6-7
Letter zone	A	B
Lagomorpha		
*Desmatolagus youngi* (Gureev, 1960)	x	
*Desmatolagus gobiensis* Matthew & Granger, 1923	x	x
Eulipotyphla		
*Zaraalestes minutus* (Matthew & Granger, 1924a)	x	x
*Palaeoscaptor acridens* Matthew & Granger, 1924a	x	x
*Palaeoscaptor tenuis* Ziegler et al., 2007	x	x
Erinaceidae indet.	x	
Crocidosoricinae indet.		x
Rodentia		
*Ninamys kazimierzi* Vianey-Liaud et al., 2013	x	x
*Ninamys arboraptus* (Shevyreva, 1966)	x	
*Eomys* cf. *orientalis* Wang & Emry, 1991	x	
*Huangomys frequens* Schmidt-Kittler et al., 2007	x	
*Cyclomylus lohensis* Matthew & Granger, 1923		x
*Cyclomylus intermedius* Wang, 2001	x	
*Coelodontomys asiaticus* Wang, 2001	x	
*Tsaganomys altaicus* Matthew & Granger, 1923	x	x
*Allosminthus minutus* (Daxner-Höck, 2001)		x
*Heosminthus chimidae* Daxner-Höck et al., 2014	x	x
*Heosminthus borrae* Daxner-Höck et al., 2014		x
*Shamosminthus sodovis* Daxner-Höck, 2001	x	
*Bohlinosminthus parvulus* (Bohlin, 1946)	x	
*Ulaancricetodon badamae* Daxner-Höck, 2000	x	x
*Eocricetodon meridionalis* (Wang & Meng, 1986)	x	
*Eucricetodon caducus* (Shevyreva, 1967)	x	x
*Eucricetodon asiaticus* Matthew & Granger, 1923	x	x
Cricetidae s.l. indet.	x	x
Creodonta		
*Hyaenodon pervagus* Matthew & Granger, 1924b	x	
Carnivora		
cf. *Asiavorator* sp.		x
Ruminantia		
*Eumeryx culminis* Matthew & Granger, 1924a	x	
Ruminantia indet.	x	x

### **Locality Hsanda Gol**

From the Hsanda Gol area, we investigated three sections: SHG-C (Fig. 3s), SHG-A, and SHG-D (Figs. 3t and u).

**Fig. 24:**
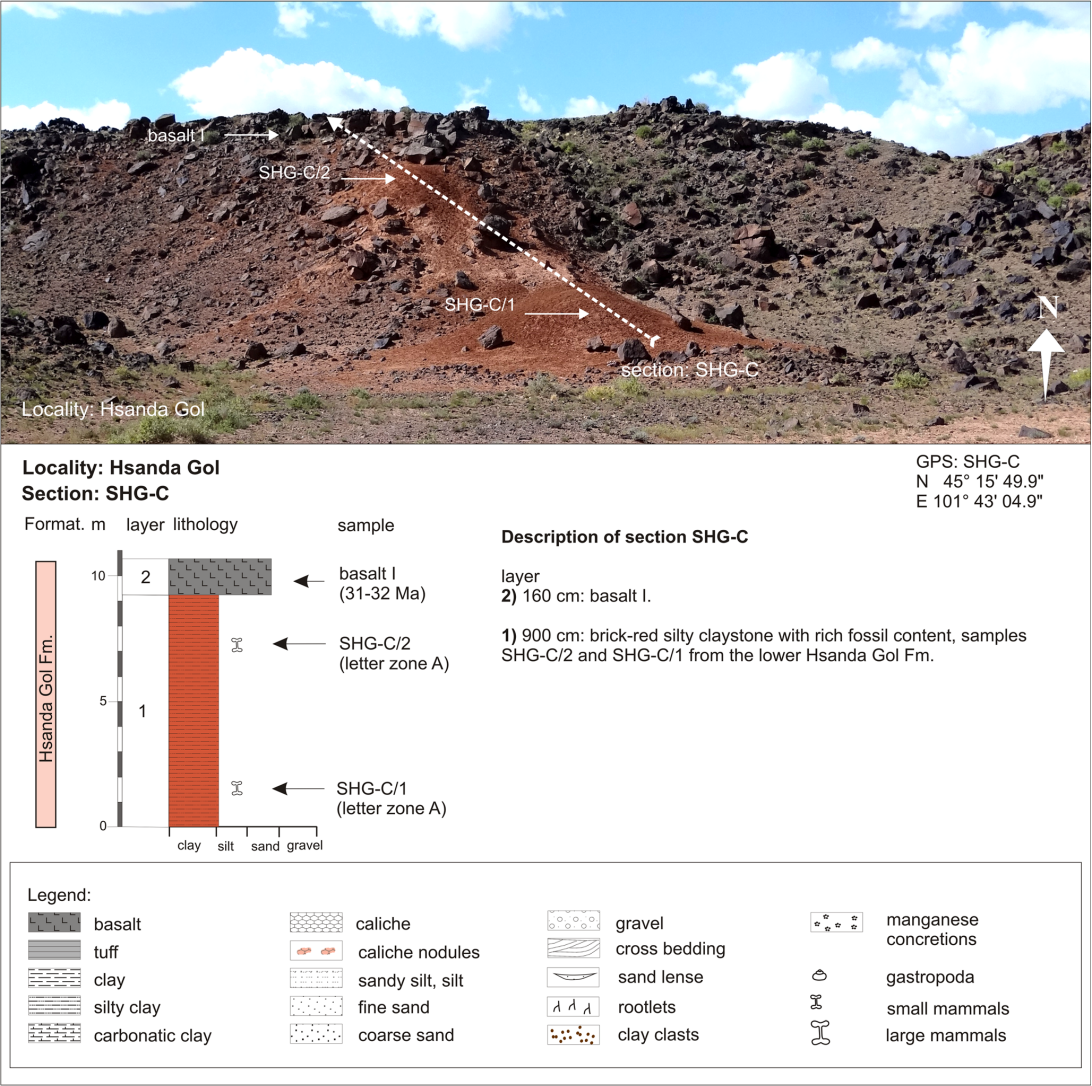
The Hsanda Gol section SHG-C is located below basalt I of a widespread basalt flow between the Tatal Gol and Hsanda Gol localities

A basalt plateau of ∼50 km^2^ extension is exposed between the Tatal Gol and Hsand Gol regions, and section SHG-C (Fig. 24) is located in its south-eastern corner. East of the basalt plateau, a SW → NE striking ridge consists of sequences of the Hsanda Gol Fm. The sections SHG-A and SHG-D are located at the southern part of this ridge. Following the ridge in NW direction, the Hsanda Gol Fm. is topped by strata of the Loh Fm. A small dry creek, the “Hsanda Gol,” east of the ridge is giving name to the entire region and to the Hsanda Gol Fm.

#### Section: SHG-C; samples: SHG-C/1-2

Below basalt I (31–32 Ma), 10 m of red-brown silty claystone yields fossils of letter zone A (samples—SHG-C/1 and SHG-C/2). The early Oligocene age is indicated by basalt I and by the fossils.

#### Sections: SHG-A and SHG-D; samples: SHG-A/1, SHG-A/9-10, SHG-A/12, SHG-A/15, SHG-A/17-20; SHG-AB/12, SHG-AB/15-20, SHG-AB/top

**Fig. 25:**
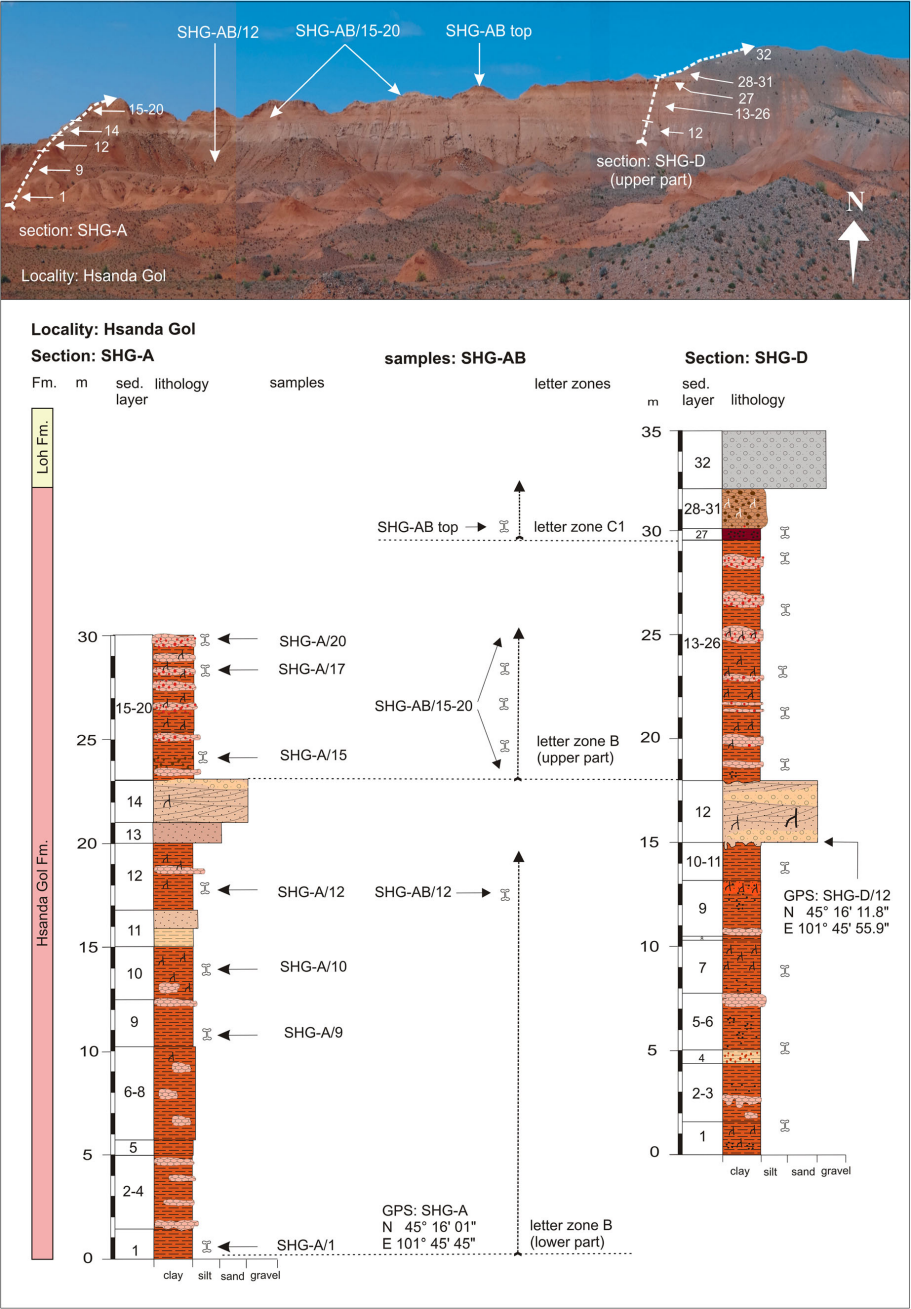
Sections SHG-A and SHG-D are located at the south-western part of the S → NE striking ridge connecting the Hsanda Gol and Loh localities. The southern part of the ridge is dominated by sediments of the Hsanda Gol Fm

The Hsanda Gol sections SHG-A, SHG-D, and the SHG-AB samples have no contact to any basalt; however, letter zone B is indicated by the rich fossil content (Fig. 25; Tables 15 and 16 and Table 17). Here, the upper Hsanda Gol beds are composed of 35–40 m red-brown claystone alternating with caliche. This sequence is divided by 3 m of sandstone and gravels (layers—SHG-A/13+14 and SHG-D/12). A significant orange caliche layer (SHG-D/28-31) above dark brown clay-stone (SHG-D/27) terminates the lower Oligocene strata. On top of this sequence, fossils of letter zone C1 indicate the upper Oligocene.

**Table 16:** Description of sections SHG-A and SHG-D from the Hsanda Gol region

**Description of section SHG**-**A**
Layer
**(16**–**20**) 450 cm: light red-brown claystone alternates with white-rose caliche layers and mottled caliche; caliche with red clay clasts, rootlets. Samples SHG-A/17, SHG-A/18, SHG-A/20
**(15**) 240 cm: light red-brown claystone with brown clasts, rich fossil content; sample SHG-A/15
**(14**) 200 cm: light grey-ochre fine sand and medium gravel with silt layers, well bedded, cross-bedded
**(13**) 100 cm: beige to orange silt and sandy beds overlying the red claystone. Red-brown claystone with rich fossil content; sample SHG-A/12
**(11**) 180 cm: orange-brown claystone with silt lenses; upper part silty sand
**(10**) 320 cm: red-brown clay; sample SHG-A/10
**(9**) 230 cm: red-brown clay; sample SHG-A/9
**(6**–**8**) 450 cm: red-brown calichized claystone with interbedded soft clay layers
**(5**) 70 cm: red-brown silty claystone
**(2**–**4**) 350 cm: red-brown claystone alternates with caliche and soft clay layers; poor fossil content
**(1**) 150 cm: red-brown silty clay, sample SHG-A/1 from the upper Hsanda Gol Fm.
**Description of section SHG**-**D**
Layer
**(32**) → gravels of the Loh Fm.
**(28**–**31**) 200 cm: orange caliche with dark brown clay clasts (5–50 mm) alternating with reddish-brown, silty, calichized claystone. Basal layer with high carbonate content; few rootlets
**(27**) 50 cm: dark red-brown silty claystone with rare gravel components, small bones, concretions, manganese-rich caliche layers, and knolls
**(13**–**26**) 1150 cm: light red-brown claystone alternates with white-rose caliche layers and mottled caliche; caliche with red clay clasts; rich fossil content. Basal part reddish-ochre silty clay with clasts, rootlets, low manganese content
**(12**) 300 cm: light grey-ochre fine sand and medium gravel with silt layers; cross bedding; ripple-bedded; uppermost part fine sand with clay layers, upper surface irregular
**(10**–**11**) 180 cm: brick-red claystone; moderate manganese content; fine gravel bound to bioturbation; clay more weathered towards overlying sandy beds; ochre clay in basal part; fossil rich
**(9**) 280 cm: light brown silty clay with limonitic patches; 60 cm above base changing to dark red clay; high manganese content; in upper parts fine sandy contents; caliche at the base
**(8**) 25 cm: homogeneous, brick-red claystone
**(7**) 250 cm: light red-brown claystone with sand lenses; abundant rootlets; manganese bound to rootlets, limonitic colouration; carbonate-free; lateral concretions
**(5**–**6**) 185 cm: red-brown claystone with manganese; caliche in its middle part; fossil rich
**(4**) 70 cm: ochre-brown clay with fine gravel clasts and sand lenses; on top grading into caliche
**(2**–**3**) 280 cm: red-brown silty claystone with manganese precipitates; upwards becoming browner; upwards caliche lenses and nodules
**(1**) 155 cm: red-brown claystone of the lower Hsanda Gol Fm.; claystone with manganese precipitates; carbonate free

**Table 17:** Fossils from the Hsanda Gol locality (sections SHG-C, SHG-A, and SHG-AB-samples yield fossils of the letter zones A, B, and C1)

Hsanda Gol	SHG-C/1+2	SHG-A/1-9	SHG-A/15-20	SHG-AB/12+13	SHG-AB/17-20	SHG-AB/top.
Letter zone	A	B	B	B	B	C1
Amphibia						
Anura indet.	x					
Reptilia						
*Tinosaurus* sp.		x				
Lacertidae indet.						
*Calamagras* sp.						
Melanosaurini indet.		x				
Mammalia						
Marsupialia						
*Asiadelphis zaissanensis* Gabunia et al., 1990			x			
Lagomorpha						
*Ordolagus* cf. *teilhardi* (Burke, 1941)		x			x	
*Desmatolagus youngi* (Gureev, 1960)	x			x		
*Desmatolagus gobiensis* Matthew & Granger, 1923	x	x	x	x	x	
*Desmatolagus* cf. *simplex* (Argyropulo, 1940)				x		
*Desmatolagus* cf. *chinensis* Erbajeva & Sen, 1998					x	
*Desmatolagus* cf. *orlovi* (Gureev, 1960)	x				x	
*Desmatolagus* sp.	x		x			
*Sinolagomys kansuensis* Bohlin, 1937					x	
Eulipotyphla						
*Zaraalestes minutus* (Matthew & Granger, 1924a)	x	x	x	x		
*Palaeoscaptor acridens* Matthew & Granger, 1924a	x		x	x	x	
*Palaeoscaptor tenuis* Ziegler et al., 2007			x		x	
*Gobisorex kingae* Sulimski, 1970	x		x		x	
*Taatsiinia hoeckorum* Ziegler et al., 2007			x		x	
Crocidosoricinae indet.			x			
Heterosoricinae indet					x	
Mongolopala tathue Ziegler et al., 2007	x					
Talpidae indet.	x					
Rodentia						
*Ninamys arboraptus* (Shevyreva, 1966)	x					
*Eomys* aff. *orientalis* Wang & Emry, 1991			x		x	
*Karakoromys decessus* Matthew & Granger, 1923	x	x				
*Yindirtemys shevyrevae* Vianey-Liaud et al., 2006			x		x	
*Tataromys sigmodon* Matthew & Granger, 1923			x			
*Tataromys plicidens* Matthew & Granger, 1923						x
*Ardynomys* sp.	x					
*Cyclomylus intermedius* Wang, 2001	x	x	x		x	
*Cyclomylus lohensis* Matthew & Granger, 1923		x	x		x	
*Coelodontomys asiaticus* Wang, 2001		x	x		x	
Tsaganomyidae indet.	x	x	x		x	
*Tsaganomys altaicus* Matthew & Granger, 1923			x	x	x	
*Allosminthus khandae* (Daxner-Höck, 2001)	x					
*Allosminthus minutus* (Daxner-Höck, 2001)		x	x		x	
*Heosminthus chimidae* Daxner-Höck et al., 2014	x	x	x		x	
*Heosminthus* sp.			x		x	
*Onjosminthus baindi* Daxner-Höck et al., 2014	x		x		x	
*Shamosminthus* sp.					x	
*Bohlinosminthus parvulus* (Bohlin, 1946)					x	
*Ulaancricetodon badamae* Daxner-Höck, 2000			x	x		
*Selenomys mimicus* Matthew & Granger, 1923		x				
*Cricetops dormitor* Matthew & Granger, 1923	x	x				
*Eocricetodon meridionalis* (Wang & Meng, 1986)	x				x	
*Eucricetodon caducus* (Shevyreva, 1967)	x	x	x			
*Eucricetodon asiaticus* Matthew & Granger, 1923	x	x	x		x	
*Tachyoryctoides obrutschewi* Bohlin, 1937						x
Creodonta						
*Hyaenodon* cf. *mongoliensis* (Dashzeveg, 1964)	x					
*Hyaenodon pervagus* Matthew & Granger, 1924b				x	x	
*Hyaenodon eminus* Matthew & Granger, 1925a					x	
Hyaenodontidae indet.				x	x	
Carnivora						
*Amphicynodon teilhardi* (Matthew & Granger, 1924b)					x	
*Amphicynodon* sp.					x	
*Shandgolictis elegans* Hunt, 1998				x	x	
*Nimravus mongoliensis* (Gromova, 1959)				x	x	
*Palaeogale* sp.				x		
Carnivora indet.				x		
Leptictida						
*Didymoconus colgatei* Matthew & Granger, 1924b				x		
Artiodactyla						
*Gobimeryx* sp.					x	
*Eumeryx* sp.				x	x	

### **Locality Loh**

#### Sections: LOH-C and LOH-B; samples: LOH-C/1, LOH-B/3

**Fig. 26:**
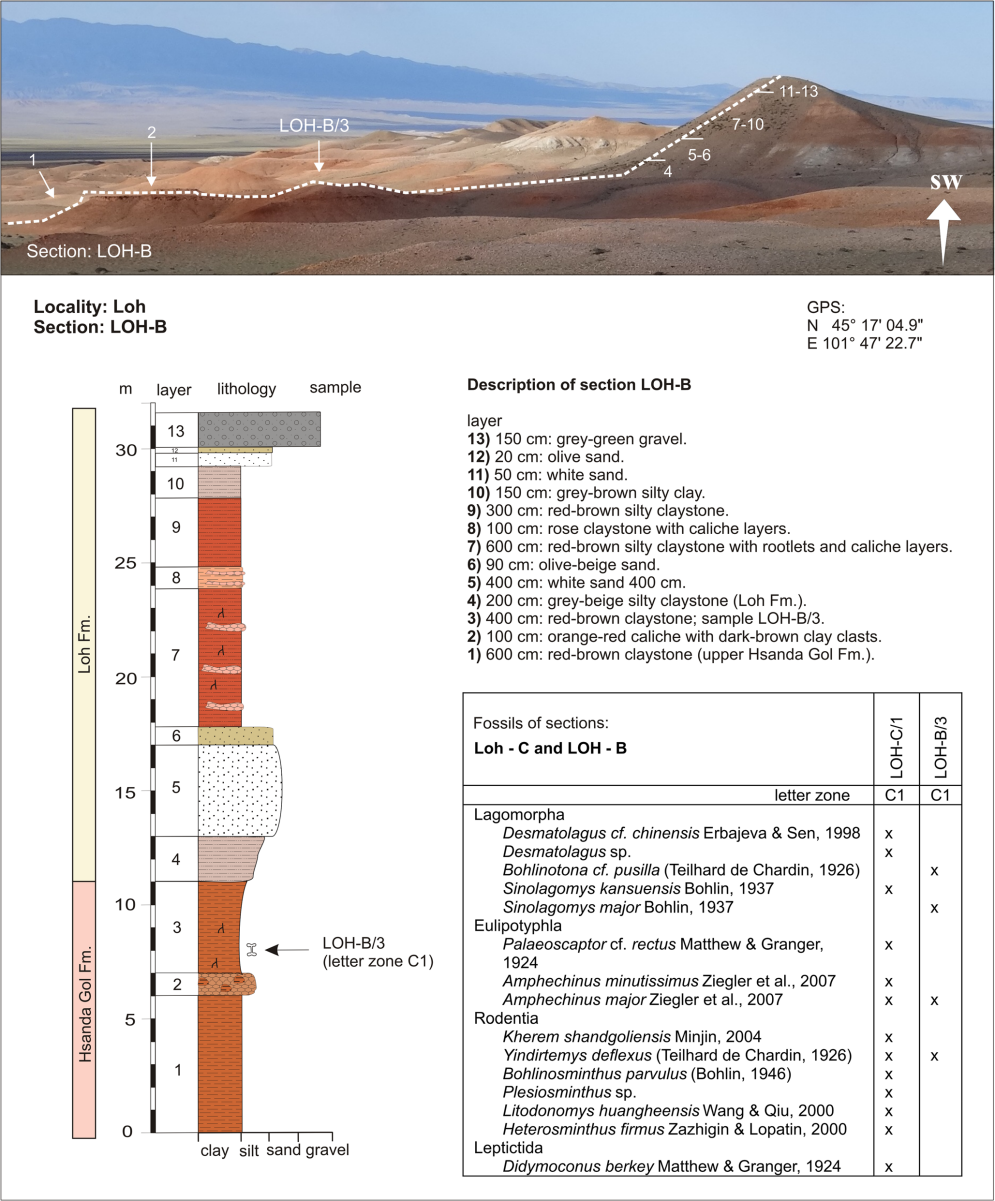
Section LOH-B and fossil list of sections LOH-B and LOH-C

*The sections LOH*-*C and LOH*-*B* (Fig. 3v and w) are located in the middle and north-eastern part of the SW → NE striking ridge, between the Hsanda Gol and Loh regions. From bottom to top, the sections display strata of the Hsanda Gol and Loh Fms. (Fig. 26). Above the orange caliche layer, characteristic fossils of letter zone C1 were found (LOH-C/1 and LOH-B/3). Upsection, light-coloured sand of the Loh Fm. alternates with red silty clay.

### **Locality Talyn Churum**

#### Sample: GRAB-II

**Fig. 27:**
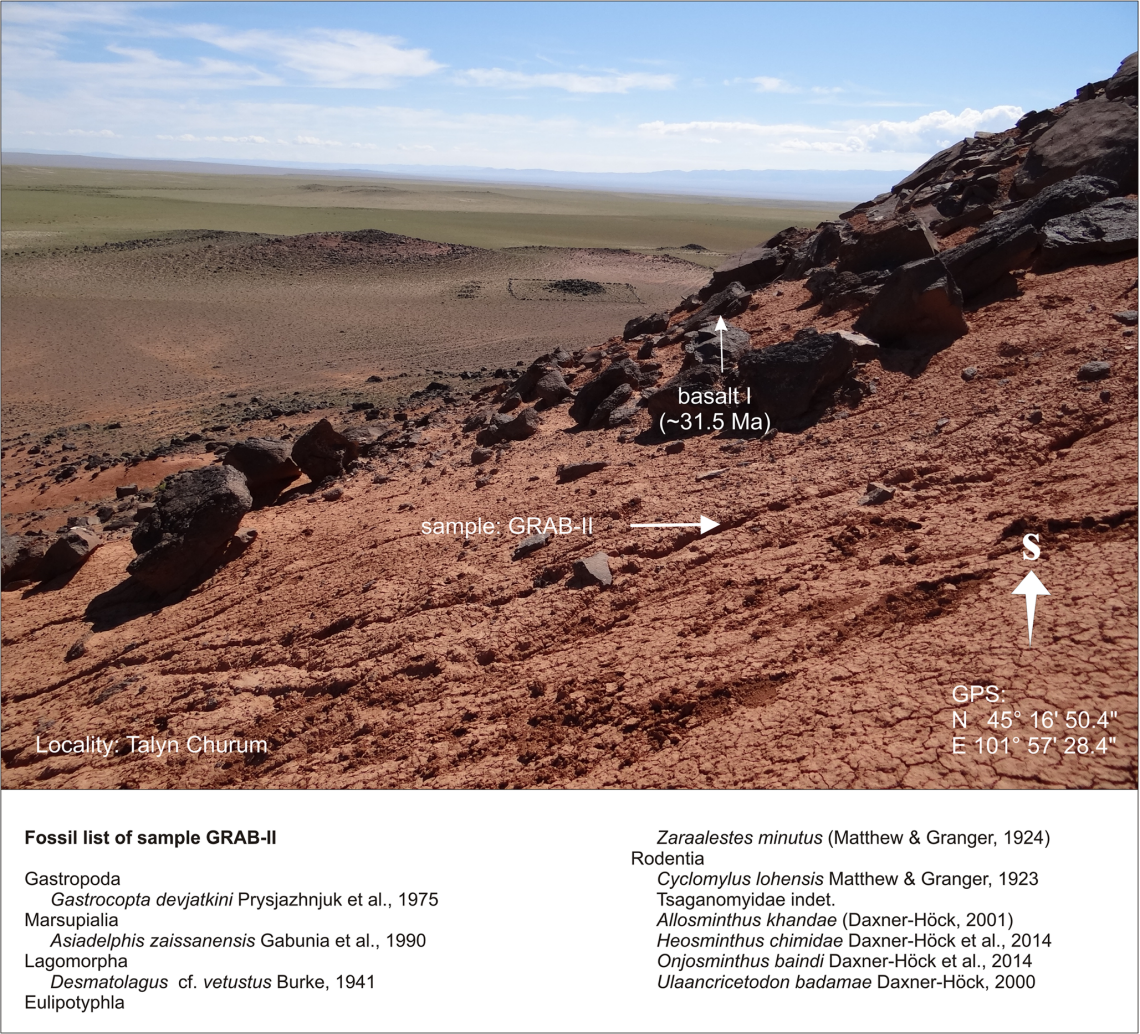
Talyn Churum is located in the eastern part of the studied area, southeast of the volcano Dzun Hsir. The sample GRAB-II was excavated below basalt I. Graves of the Bronze Age are visible in the background

Talyn Churum is one of the eastern fossil points (Fig. 3x). The short section displays about 10 m of red-brown silty claystone of the Hsanda Gol Fm. below basalt I (31–32 Ma) (Fig. 27). The early Oligocene age of the included fauna (letter zone A) is indicated by basalt I.

### **Locality Ikh Argalatyn Nuruu**

#### Sections: IKH-A and IKH-B; samples: IKH-A/1-5, IKH-B/2, IKH-B/5

**Fig. 28:**
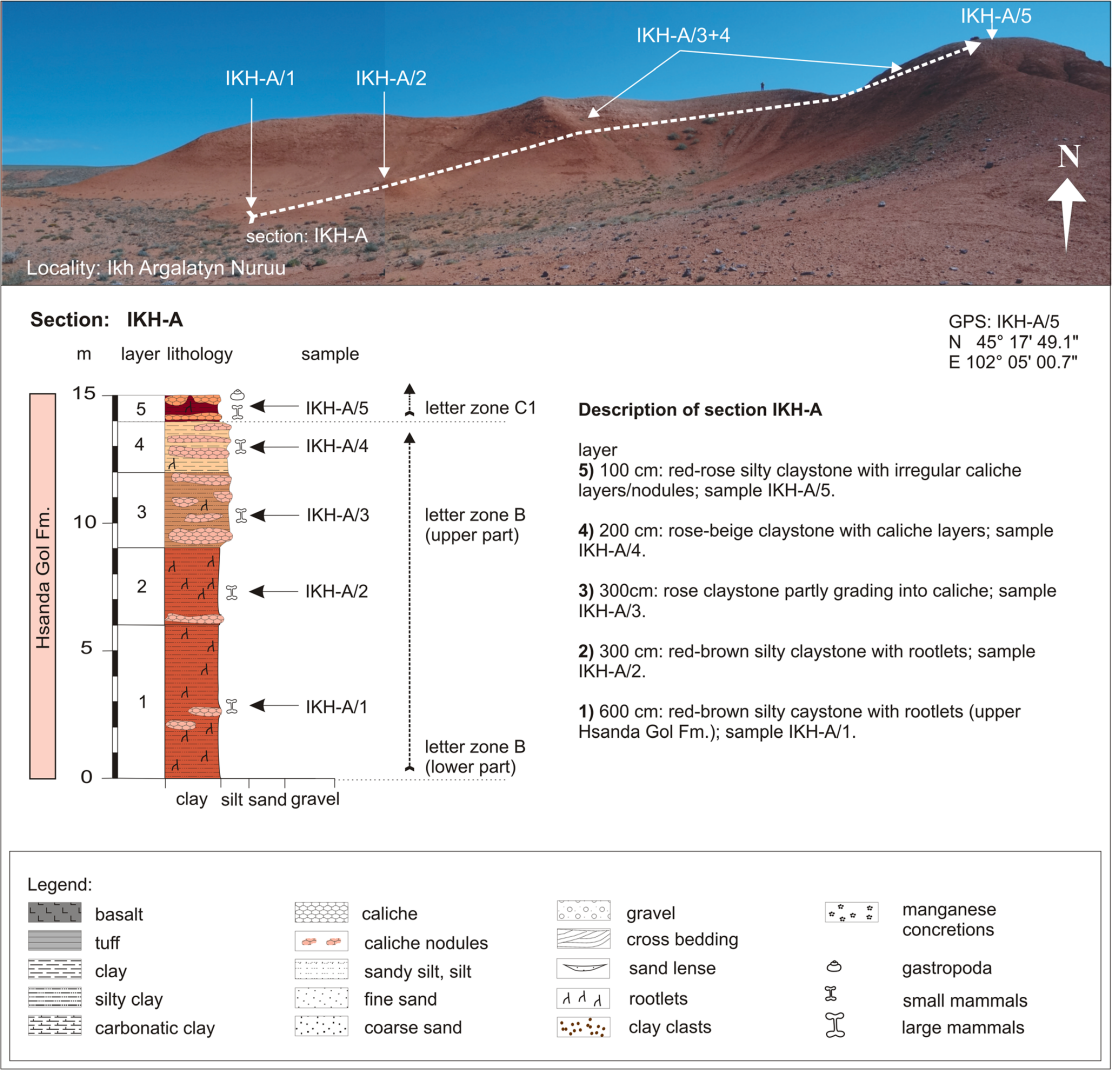
Ikh Argalatyn Nuruu is located in the eastern part of the studied area. Section IKH-A comprises sequences of the upper Hsanda Gol Fm. ranging from the early to the late Oligocene

**Fig. 29:**
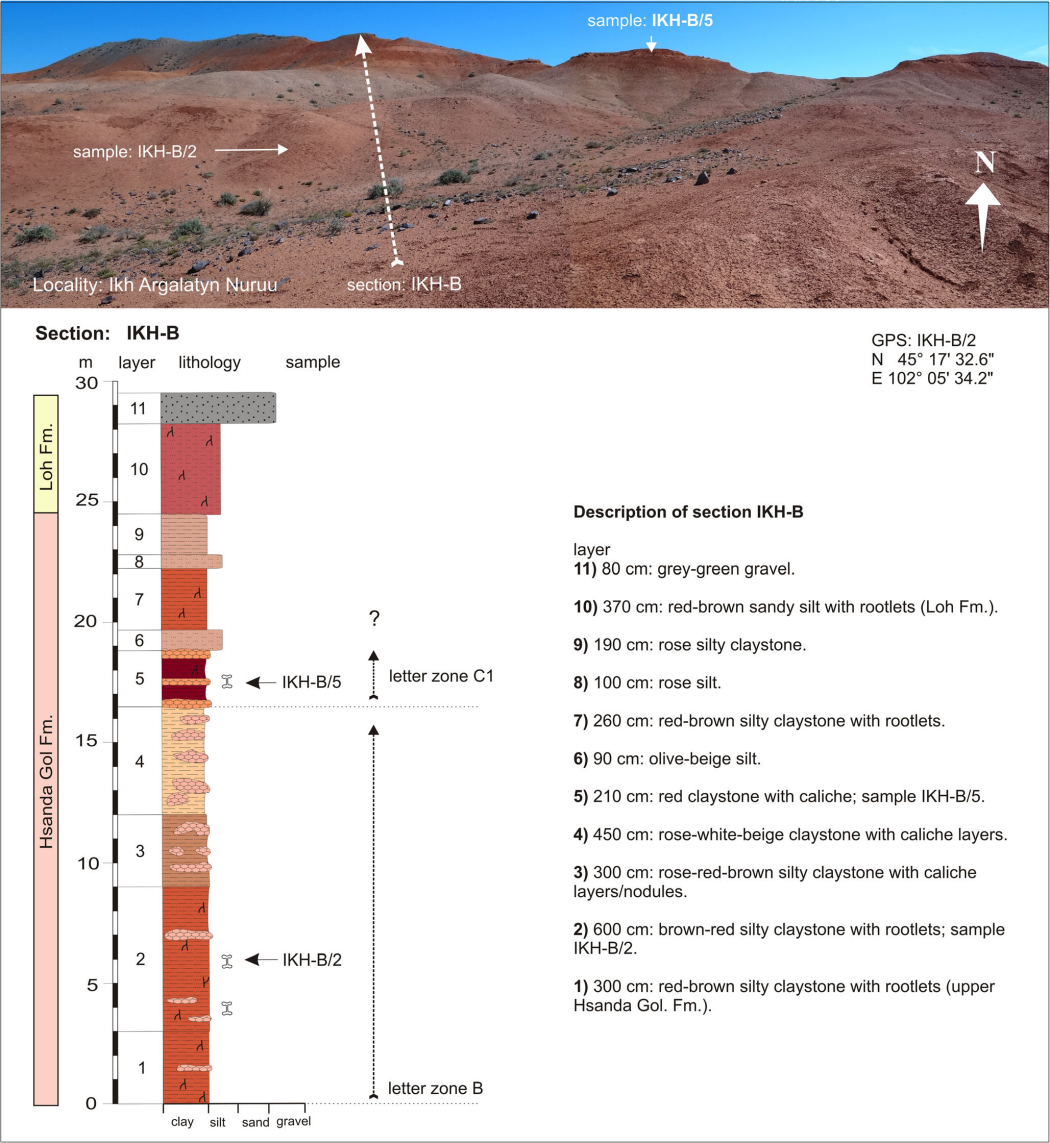
Section IKH-B is located in the easternmost part of the study area. The section comprises sequences of the Hsanda Gol and Loh Fms

From Ikh Argalatyn Nuruu, two sections were investigated, section IKH-A (Fig. 3y) and section IKH-B (Fig. 3z). The two sections are located in the easternmost part of the study area. Section IKH-A exposes red silty clay layers alternating with caliche of the upper Hsanda Gol Fm. Samples IKH-A/1-4 yield fossils of letter zone B. The top layer of orange caliche (yielding *Y. deflexus*) marks the lower boundary of letter zone C1 (Fig. 28,29; Table 18).

**Table 18:** Fossils from the locality Ikh Argalatyn Nuruu [fossils of section IKH-A (samples IKH-A/1-4) indicate the letter zone B (early Oligocene), fossils from samples IKH-A/5 and IKH-B/5 indicate letter zone C1 (late Oligocene)]

Ikh Argalatyn Nuruu	IKH-A/1-4	IKH-A/5	IKH-B/5
Letter zone	B	C1	C1
Gastropoda			
*Vallonia* sp.		x	
Reptilia			
Acrodonta indet.	x		
*Lacerta* sp. 1	x		
Mammalia			
Lagomorpha			
*Ordolagus* cf. *teilhardi* (Burke, 1941)	x		
*Desmatolagus youngi* (Gureev, 1960)	x		
*Desmatolagus gobiensis* Matthew & Granger, 1923	x		
*Desmatolagus robustus* Matthew & Granger, 1923	x		
*Desmatolagus* cf. *chinensis* Erbajeva & Sen, 1998	x		x
*Desmatolagus* sp.	x		
*Sinolagomys major* Bohlin, 1937	x		
*Sinolagomys* sp.		x	x
Eulipotyphla			
*Zaraalestes minutus* (Matthew & Granger, 1924a)	x		
*Palaeoscaptor acridens* Matthew & Granger, 1924a	x		
*Palaeoscaptor* cf. *rectus* Matthew & Granger, 1924a		x	
*Amphechinus major* Ziegler et al., 2007		x	x
*Gobisorex kingae* Sulimski, 1970	x	x	x
Rodentia			
*Promeniscomys* cf. *sinensis* Wang 1987	x		
*Ninamys kazimierzi* Vianey-Liaud et al., 2013	x		
*Ninamys arboraptus* (Shevyreva, 1966)	x		
*Yindirtemys deflexus* (Teilhard de Chardin, 1926)		x	x
*Ardynomys* sp.	x		
*Cyclomylus intermedius* Wang, 2001	x		
*Cyclomylus lohensis* Matthew & Granger, 1923	x		
*Coelodontomys asiaticus* Wang, 2001	x		
Tsaganomyidae indet.	x		
*Tsaganomys altaicus* Matthew & Granger, 1923	x	x	x
*Allosminthus minutus* (Daxner-Höck, 2001)	x		
*Heosminthus chimidae* Daxner-Höck et al., 2014	x		
*Heosminthus* sp.	x		
*Onjosminthus baindi* Daxner-Höck et al., 2014	x		
*Shamosminthus sodovis* Daxner-Höck, 2001	x		
*Bohlinosminthus parvulus* (Bohlin, 1946)	x		x
*Ulaancricetodon badamae* Daxner-Höck, 2000	x		
*Cricetops dormitor* Matthew & Granger, 1923	**x**		
*Eocricetodon* cf. *meridionalis* (Wang & Meng, 1986)	x		
*Eucricetodon caducus* (Shevyreva, 1967)	x		
*Eucricetodon asiaticus* Matthew & Granger, 1923	x		
*Eucricetodon* cf. *occasionalis* Lopatin, 1996	x		
*Eucricetodon bagus* Gomes Rodrigues et al., 2012	x		x
*Eucricetodon* sp.	x	x	
Cricetidae indet.	x		x
*Aralocricetodon* cf. *schokensis* Bendukidze, 1993		x	
*Tachyoryctoides obrutschewi* Bohlin, 1937		x	
Creodonta			
*Hyaenodon* cf. *incertus* Dashzeveg, 1985	x		
*Hyaenodon pervagus* Matthew & Granger, 1924b	x		
cf. *Hyaenodon gigas* Dashzeveg, 1985	x		
Carnivora			
*Amphicynodon teilhardi* (Matthew & Granger, 1924b)	x		
aff. *Amphicynodon* sp.	x		
*Nimravus mongoliensis* (Gromova, 1959)	x		
*Palaeogale sectoria* sp.	x		
Ruminantia			
*Pseudogelocus mongolicus* Vislobokova & Daxner-Höck, 2002	x		
*Prodremotherium* sp.	x		
*Eumeryx* sp.	x		
*Amphitragulus* sp.			x
? *Gobiocerus* sp.			x

## Correlation of geological sections from the Valley of Lakes

**Fig. 30:**
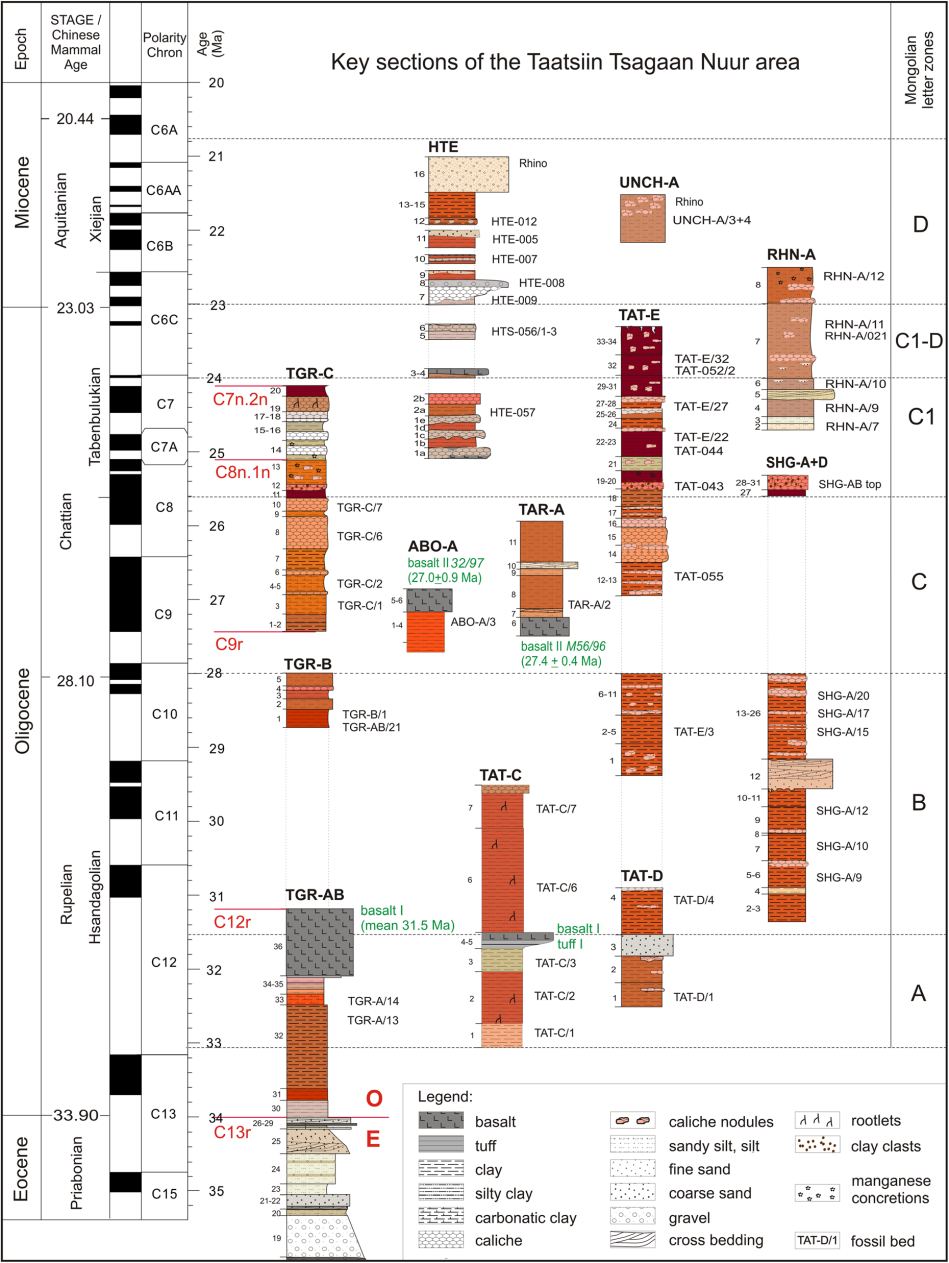
Chronostratigraphic and geochronologic correlation of key sections from the Valley of Lakes. The stratigraphic chart includes the Geologic Time Scale and the GPTS (Gradstein et al. 2012; Ogg et al. 2004); the Chinese Mammal Ages Hsandagolian, Tabenbulukian, and Xiejian (Meng and McKenna 1998; Meng et al. 2008); ^40^Ar/^39^Ar ages of basalt I and II (Table 1a and b) and Höck et al. (1999; Fig. 18); magnetostratigraphic data (Kratz and Geisler 2010; Sun and Windley 2015); key sections from the Taatsiin Gol and Taatsiin Tsagaan Nuur region; and the Mongolian letter zones A, B, C, C1, C1-D, and D. Left side of the sections, the sediment layers are numbered. The position of palaeontological samples/fossil layers is shown right side of the sections (e.g. TGR-A/13)

Today, the combination of biostratigraphic and lithologic data from the Taatsiin Gol and Taatsiin Tsagaan Nuur regions, the ^40^Ar/^39^Ar ages of basalts (Tables 1 and 2), and magnetostratigraphic data (Sun and Windley 2015) allows correlation of sections and fossil horizons with the Geomagnetic Polarity Time Scale (GPTS) (Gradstein et al. 2012). This provides a composite age chronology for the entire sequence as demonstrated for selected key sections (Fig. 30).

### Late Priabonian to early Rupelian (Hsandagolian/letter zone A)

As outlined above, the lower part of section TGR-AB (Figs. 14 and 30) comprises fluvio-lacustrine deposits of the Tsagan Ovo Fm. followed by brick-red clay of the Hsanda Gol Fm. (lower Hsanda Gol beds), which is topped by basalt I (^40^Ar/^39^Ar age ∼31.5 Ma). The fossils of samples TGR-A/13+14 below basalt I (Table 11) evidence letter zone A and the early Hsandagolian Mammal age, respectively. These data allow correlation of magnetostratigraphic measurements along of the TGR section with the GPTS, showing that the lower Hsanda Gol beds and basalt I are to be correlated with Chrons C12r–C13r (section A in Sun and Windley 2015; Fig. 3) and the early Rupelian, respectively. The age range of the lower Hsanda Gol beds is ∼34–31.5 Ma. The age range of the Tsagan Ovo sequence is >35 to ∼34 Ma (late Piabonian). The Eocene and Oligocene boundary (EOB; Figs. 14 and 30) is located between the Tsagan Ovo and the Hsanda Gol Fms. at ∼34 Ma (Kraatz and Geisler 2010; Sun and Windley 2015).

**Fig. 31:**
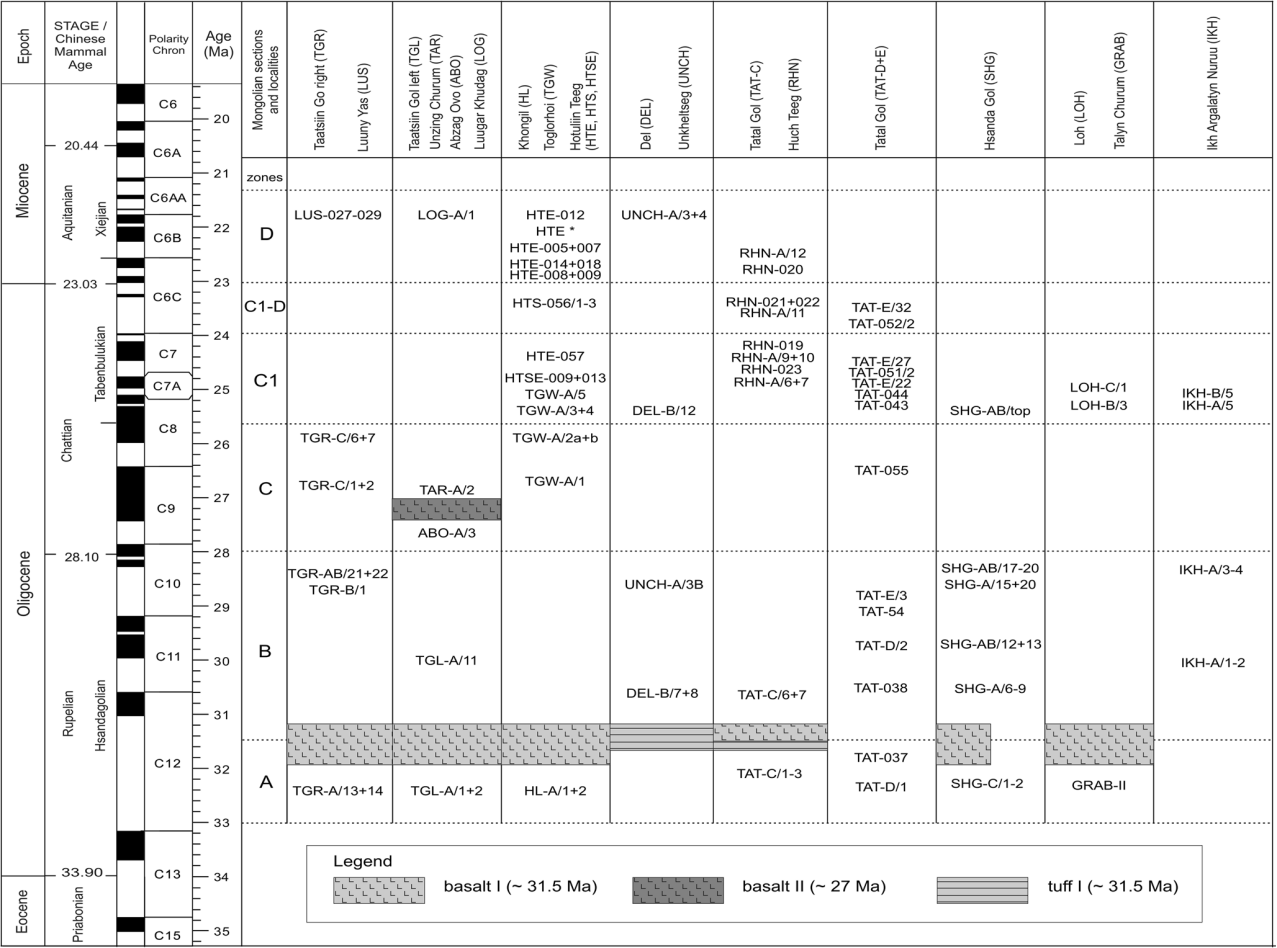
Chronostratigraphic correlation and calculation of geochronologic ages of mammal assemblages from the Valley of Lakes in Mongolia. The correlation chart includes the Geologic Time Scale and the Geomagnetic Polarity Time Scale (GPTS) (Gradstein et al 2012; Ogg et al. 2004); the Chinese Mammal Ages Hsandagolian, Tabenbulukian, and Xiejian; ^40^Ar/^39^Ar ages of basalt I and II (Höck et al 1999); the Mongolian letter zones A, B, C, C1, C1-D, and D; geological sections and fossil localities from the Taatsiin Gol and Taatsiin Tsagaan Nuur region (Valley of Lakes); and the respective mammal assemblages (acronyms)

Sediment sequences of the early Rupelian (below basalt I or tuff I) are evidenced in the regions Taatsiin Gol (sections TGR-A, TGR-AB, TGR-B, HL-A, TGL-A), Del (section DEL-B), Tatal Gol (sections TAT-D and TAT-C), Hsanda Gol (section SHG-C), and Talyn Churum (GRAB-II) (see Figs. 30 and 31).

### Late Rupelian (Hsandagolian/letter zone B)

From the upper Hsanda Gol beds with fossils of letter zone B, no magnetostratigraphic data are available. The lower boundary is basalt or tuff I (∼31.5 Ma); the upper boundary is built by Hsanda Gol sequences, which include fossils of letter zone C, and which are located below basalt II (∼27 Ma).

This lower part of upper Hsanda Gol beds is not only characterised by abundant fossils of letter zone B but also by increased number and thickness of caliche layers, alternating with brick-red clay/silty clay. In the Hsanda Gol region, the sequence is interrupted by a 2–3-m-thick sandstone layer (Fig. 25).

Sediment sequences of the late Rupelian are evidenced in the regions: Taatsiin Gol (sections TGR-AB, TGR-B, TGL-A), Unkheltseg (section UNCH-A), Del (section DEL-B), Tatal Gol (sections TAT-D, TAT-E, and TAT-C), Hsanda Gol (section SHG-A, SHG-AB, SHG-D), and Ikh Argalatyn Nuruu (sections IKH-A and IKH-B) (see Figs. 30 and 31).

### Early Chattian (Hsandagolian/letter zone C) to late Chattian (Tabenbulukian/letter zones C1 and C1-D)

In the Chattian, some sections consist of sediments of the Hsanda Gol Fm. (sections TGR-C, TGW-A, TAT-E) and others of the Loh Fm. (sections TAR-A, RHN-A). Thus, both formations occur in Chattian strata. Three sections are of special importance for correlation. The sections ABO-A (Fig. 6) and TAR-A (Figs. 18 and 19) provide biostratigraphic data and radiometric ages of basalt II. Magnetostratigraphic measurements of section TGR-C (Fig. 13) allow correlation with the GPTS. In section ABO-A, fossils of letter zone C (sample ABO-A/3) were recovered below basalt II (27.0 ± 0.9 Ma); in section TAR-A, fossils of letter zone C (sample TAR-A/2) occur above basalt II (27.4 ± 0.4 Ma) (Höck et al. 1999; Daxner-Höck et al. 2010). These geochronologic data are in agreement with section TGR-C. There, the upper Hsanda Gol beds contain rich mammal assemblages of letter zone C, and fossils of letter zone C1 were sporadically found from the uppermost part of the Hsanda Gol Fm. Magnetostratigraphic measurements from section TGR-C allow correlation of the Hsanda Gol beds with Chrons C9n–C7n.2n (total range 27.4–24.2 Ma). The boundary between the reddish-brown and olive-green claystone (TGR-C/13/14) was correlated with Chron C8n.1n at 25.2 Ma (Sun and Windley 2015; Fig. 3); it is 3 m above the dark-brown claystone (TGR-C/11) marking the boundary between letter zones C and C1 at 25.6 Ma. Thus, in section TGR-C, letter zone C ranges from 27.4 to 25.6 Ma, and the range of letter zone C1 is 25.6 to 24.2 Ma (Fig. 30).

In the locality Tatal Gol, a composite section (section TAT-D+E) displays the sequence ranging from the early Rupelian to the late Chattian. The sequence evidences the early Rupelian (sample TAT-D/1 with fossils of letter zone A), followed by the late Rupelian (sample TAT-E/3 with fossils of letter zone B), the early Chattian (sample TAT-055 with fossils of letter zone C), and the late Chattian/Tabenbulukian (samples—TAT-043, TAT-044, TAT-E/22, TAT-E/27, TAT-052/1 with fossils of letter zone C1); finally, the sequence is topped by dark-brown clay at the North Ridge (samples TAT-E/32 and TAT-052/2 with fossils of letter zone C1-D; Figs. 21 and 22; Figs. 30 and 31).

In the Taatsiin Gol and Taatsiin Tsagaan Nuur region, characteristic Tabenbulukian fossils cannot be found earlier than 25.6 Ma (Chron C8n.2n). These fossils, *Yindirtemys deflexus*, *Sinolagomys kansuensis*, *Bohlinosminthus parvulus*, and *Amphechinus major*, mark the beginning of letter zone C1. Consequently, we follow Meng and McKenna (1998) and (Meng et al. 2008) and draw the Hsandagolian/Tabenbulukian boundary at 25.6 Ma (Figs. 30 and 31). We do not agree with Kraatz and Geisler (2010, Fig. 3) to shift the lower boundary of the Tabenbulukian Mammal age down to Chron C11r at 30.6 Ma. This opinion of Kraatz and Geisler (2010) contradicts our fossil data (elaborated above and illustrated in Figs. 32, 33, 34, 35, 36, 37, 38, 39, 40, 41, 42, 43, 44, 45, 46, 47, 48, 49, 50, 51, 52, 53, 54, 55, 56, 57, 58, 59, 60, 61, and 62), and also contradicts the radiometric ages of basalt II (Höck et all 1999; and Tables 1 and 2) and the magnetostratigraphic correlation of section TGR-C (Sun and Windley 2015).

**Fig. 32:**
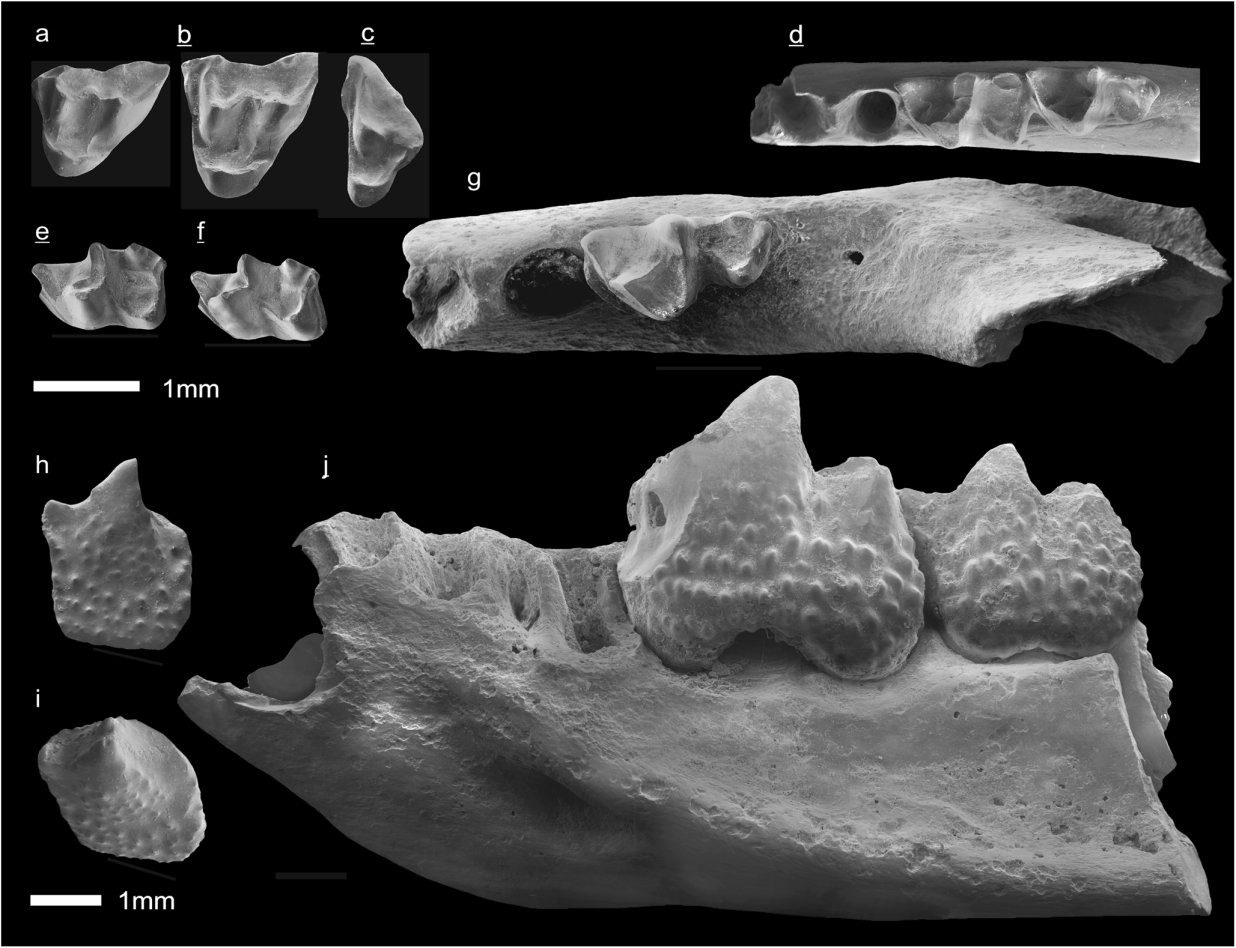
Family Didelphidae ***Asiadelphis zaissanensis*** **Gabunia**, **Shevyreva and Gabunia**, **1990** from Taatsiin Gol (TGR-B/1, TGR-AB/21, TGR-AB/22) and Hsanda Gol (SHG-A/15+20), Valley of Lakes, Mongolia. Early Oligocene, letter zone B. Ziegler et al. (2007). **a** Left D3 (NHMW 2006/0115/0001), TGR-AB/21. **b** Right M1 (NHMW 2006/0116/0002), TGR-AB/22. **c** Right M4 (NHMW 2006/0117/0001), TGR-B/1. **d** Right m3-4 (NHMW 2006/0116/0001), TGR-AB/22. **e** Right m1 (NHMW 2006/0118/0001), SHG-A/15+20. **f** Right m2/3 (NHMW 2006/0116/0002), TGR-AB/22 ***Asiadelphis tjutkovae*** **Emry**, **Lucas**, **Szalay and Tleuberdina**, **1995** from Tatal Gol (TAT-D/1 = Hü1), Valley of Lakes, Mongolia. Early Oligocene, letter zone A. Ziegler et al. (2007). **g** Left mand. m4 (NHMW 2006/0119/0001) Family Erinaceidae ***Exallerix pustulatus*** **Ziegler**, **Dahlmann and Storch**, **2007** from Taatsiin Gol (TGR-C/1), Valley of Lakes, Mongolia. Late Oligocene, letter zone C. All figured specimens are the holotype (H) and paratypes. Ziegler et al. (2007). **h** Left p4, labial view (NHMW 2006/0192/0002). **i** Left p4, occlusal view (NHMW 2006/0192/0002). **j** right mand. m1-2 (NHMW 2006/0192/0001), **H**

**Fig. 33:**
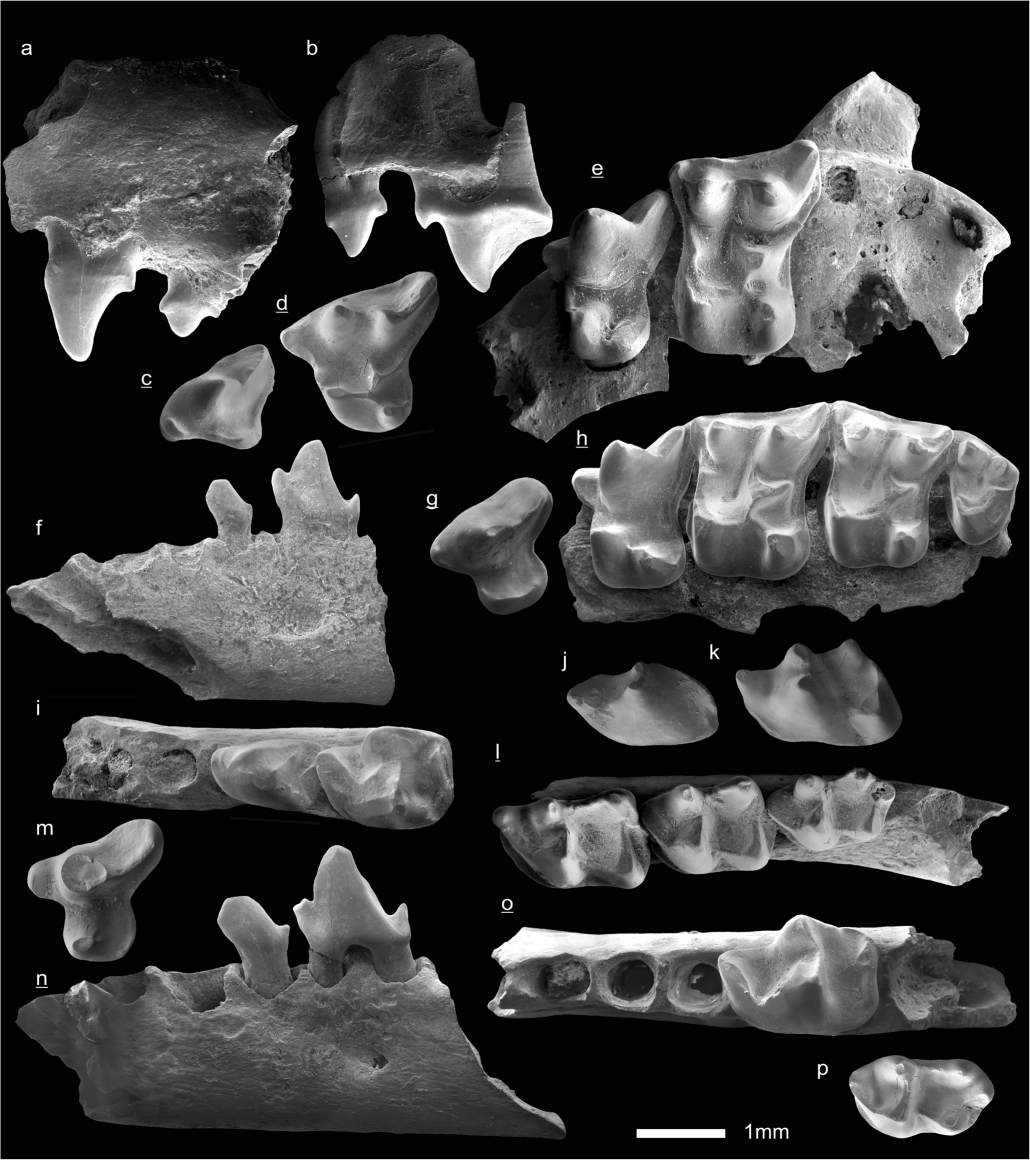
Family Erinaceidae. ***Zaraalestes minutus*** (**Matthew and Granger**, **1924**) from Tatal Gol (TAT-D/1; letter zone A) and Taatsiin Gol (TGR-B/1, TGR-AB/22; letter zone B), Valley of Lakes, Mongolia. Early Oligocene. Ziegler et al. (2007). **a** Left max. C2-P2 (NHMW 2006/0174/0006), TGR-AB/22. **b** Left max. P2-3 (NHMW 2006/0174/0002), TGR-AB/22. **c** Right D3 (NHMW 2006/0174/0003), TGR-AB/22. **d** Right D4 (NHMW 2006/0175/0001), TGR-B/1. **e** Right max. P4-M1(NHMW 2006/0174/0007), TGR-AB/22. **f** Left mand. p 2-3 (NHMW 2006/0121/0001), TAT-D/1. **g** Right P3 (NHMW 2006/0174/0005), TGR-AB/22. **h** Right max. P4-M3 (NHMW 2006/0175/0002), TGR-B/1. **i** Left mand. p3-4 (NHMW 2006/0121/0002), TAT-D/1. **j** Left d3 (NHMW 2006/0174/0001), TGR-AB/22. **k** Left d4 (NHMW 2006/0175/0001), TGR-AB/22. **l** Right mand. m1-3 (NHMW 2006/0121/0003), TAT-D/1 ***Zaraalestes*** **sp**. from Taatsiin Gol (TGR-AB/21) and Del (DEL-B/7) Valley of Lakes, Mongolia. Early Oligocene, letter zone B. Ziegler et al. (2007). **m** Left P3 (NHMW 2006/0190/0002), TGR-AB/21. **n** Right mand. p2-3 (NHMW 2006/0190/0002), TGR-AB/21. **o** Right mand. p4 (NHMW 2006/0191/0001), DEL-B/7. **p** Left m3 3 (NHMW 2006/0190/0003), TGR-AB/21

**Fig. 34:**
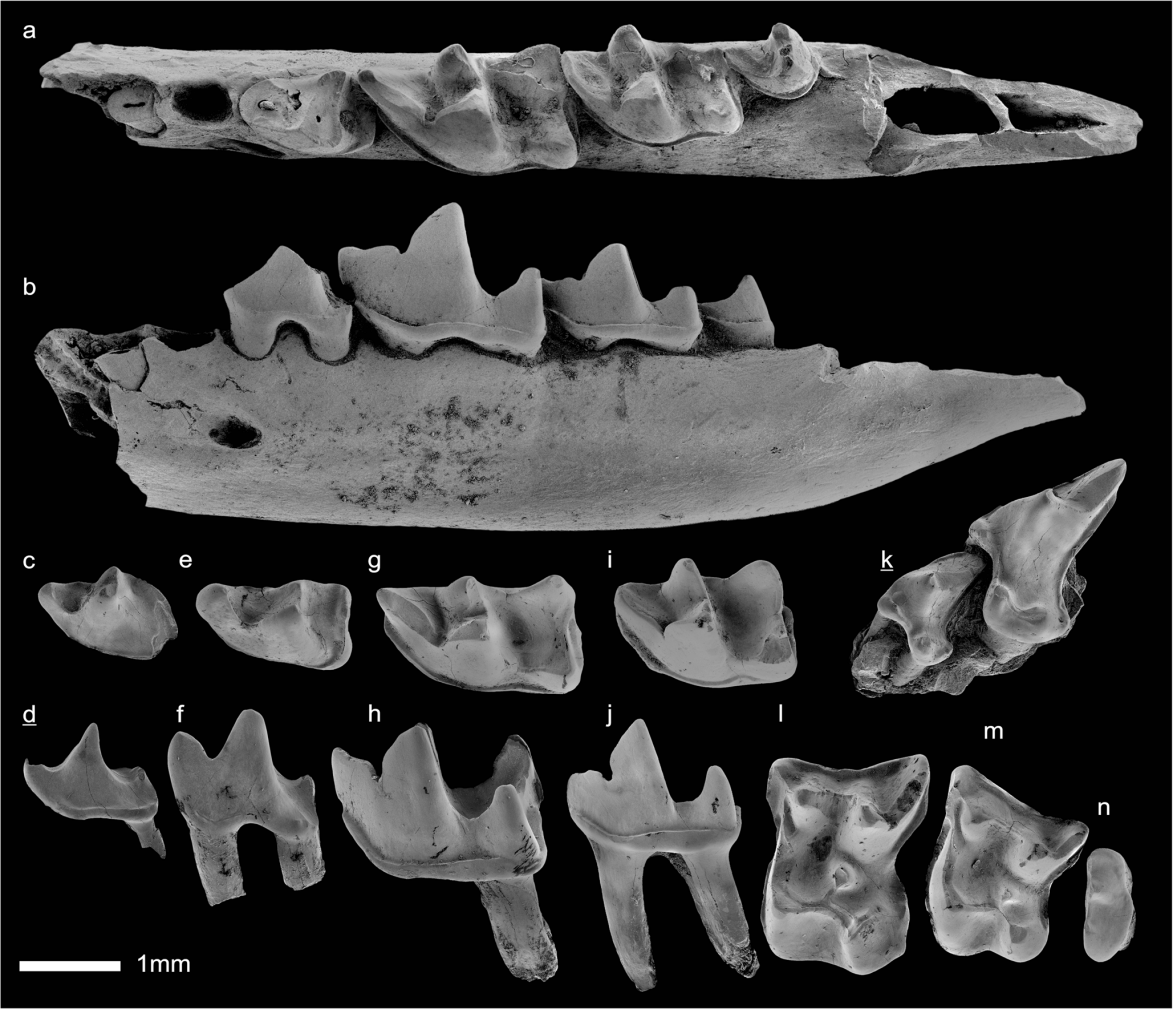
Family Erinaceidae. ***Amphechinus taatsiingolensis*** **Ziegler**, **Dahlmann and Storch**, **2007** from Toglorhoi / Khunug Valley (TGW-A/2), Valley of Lakes, Mongolia. Late Oligocene, Biozone C. All figured specimens are the holotype (H) and paratypes. Ziegler et al. (2007). **a** Left mand. p4-m3, occlusal view (NHMW 2005/0152/0001), **H. b** Left mand. p4-m3, labial view (NHMW 2005/0152/0001), **H. c** Left d4, occlusal view (NHMW 2005/0152/0002). **d** Right d4, labial view (NHMW 2005/0152/0003). **e** Left p4, occlusal view (NHMW 2005/0152/0005). **f** Left p4, labial view (NHMW 2005/0152/0004). **g** Left m1, occlusal view (NHMW 2005/0152/0006). **h** Left m1, labial view (NHMW 2005/0152/0007). **i** Left m2, occlusal view (NHMW 2005/0152/0008). **j** Left m2, labial view (NHMW 2005/0152/0009). **k** Right max. P4-M1 (NHMW 2005/0152/0013). **l** Left M1(NHMW 2005/0152/0014). **m** Left M2 (NHMW 2005/0152/0016). **n** Left M3 (NHMW 2005/0152/0017)

**Fig. 35:**
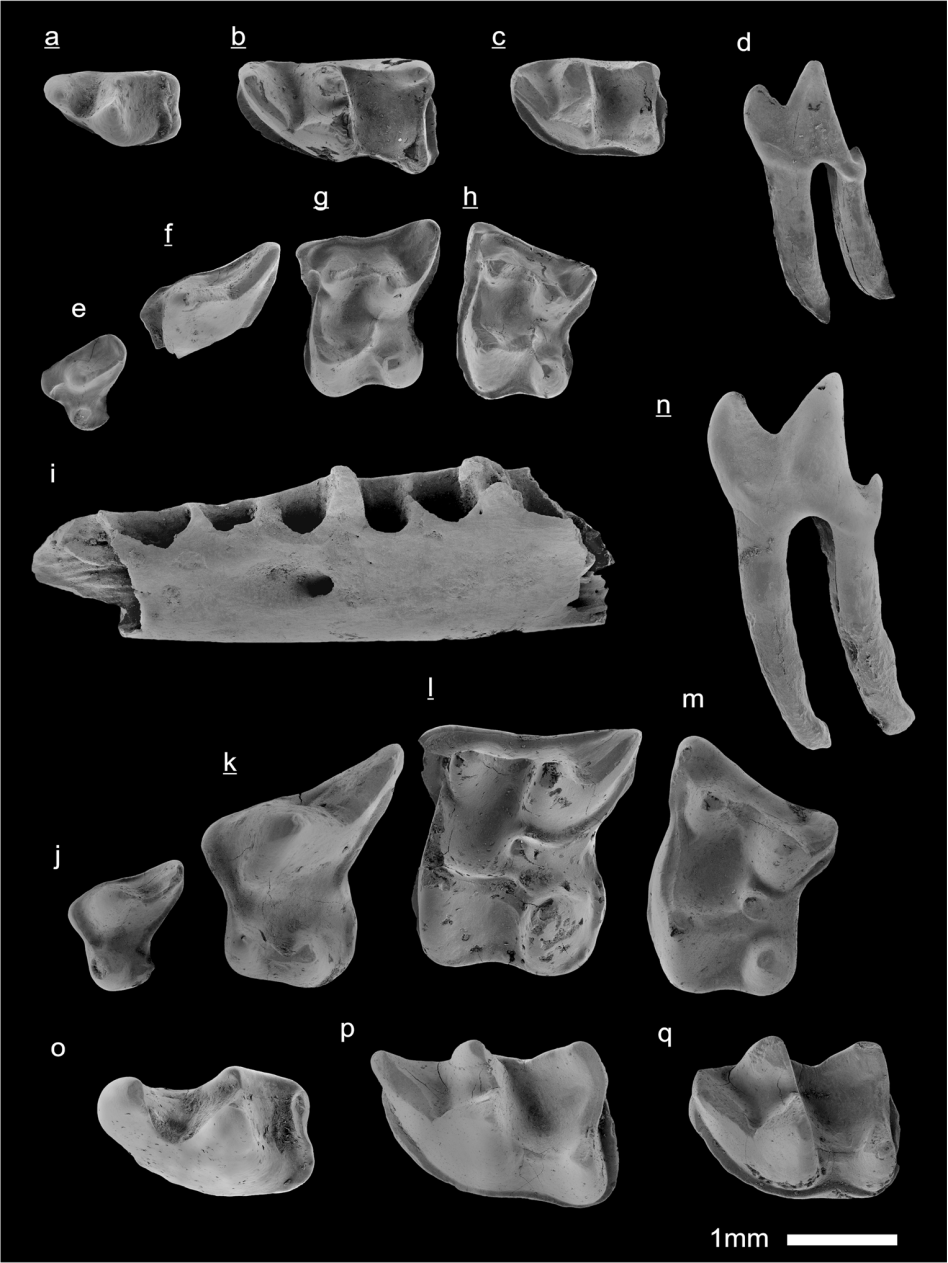
Family Erinaceidae. ***Amphechinus minutissimus*** **Ziegler**, **Dahlmann and Storch**, **2007** from Del (DEL-B/12), Valley of Lakes, Mongolia. Late Oligocene, letter zone C1. All figured specimens are the holotype (H) and paratypes. Ziegler et al. (2007). **a** Right p4 (NHMW 2005/0196/0002). **b** Right m1 (NHMW 2005/0196/0003). **c** Right m2 (NHMW 2005/0199/0003). **d** Left p4, labial view (NHMW 2005/0196/0001). **e** Left P3 (NHMW 2005/0196/0004). **f** Right P4-fragm. (NHMW 2005/0199/0005). **g** Right M1 (NHMW 2005/0199/0001), **H. h** Left M2 (NHMW 2005/0196/0005). **i** Left mand. (NHMW 2005/0199/0002). ***Amphechinus major*** **Ziegler**, **Dahlmann and Storch**, **2007** from Del (DEL-B/12), Valley of Lakes, Mongolia. Late Oligocene, Letter zone C1. All figured specimens are paratypes. Ziegler et al. (2007). **j** Left P3 (NHMW 2005/0198/0008). **k** Right P4 (NHMW 2005/0198/0003). **l** Right M1 (NHMW 2005/0183/0001). **m** Left M2 (NHMW 2005/0198/0010). **n** Right p4, labial view (NHMW 2005/0198/0004). **o** Left p4, occlusal view (NHMW 2005/0198/0002). **p** Left m1 (NHMW 2005/0198/0005). **q** Left m2 (NHMW 2005/0198/0006)

**Fig. 36:**
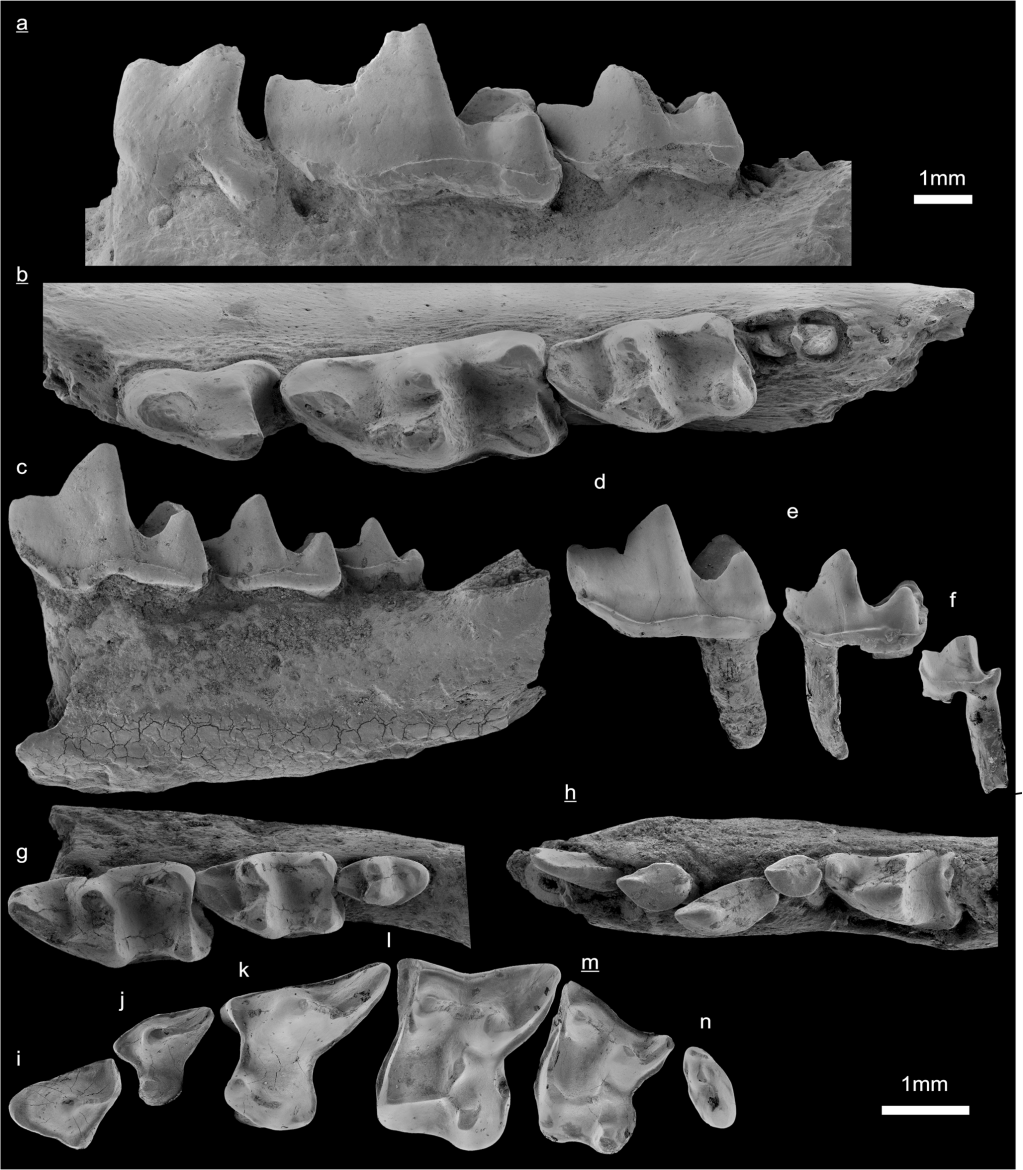
Family Erinaceidae. ***Palaeoscaptor gigas*** (**Lopatin**, **2002**) from Taatsiin Gol (TGR-C/1+2) Valley of Lakes, Mongolia. Late Oligocene, letter zone C. Ziegler et al. (2007). **a** Right mand. p4-m2, labial view (NHMW 2005/0128/0001). **b** Right mand. p4-m2, occlusal view (NHMW 2005/0128/0001) ***Palaeoscaptor tenuis*** **Ziegler**, **Dahlmann and Storch**, **2007** from Tatal Gol (TAT-D/1; letter zone A), Hsanda Gol (SHG-AB/17-20; letter zone B), and Unkheltseg (UNCH-A/3B; letter zone B), Valley of Lakes, Mongolia. Early Oligocene. H = holotype. Ziegler et al. (2007). **c** Left mand. m1-3, labial view (NHMW 2005/0103/0001), TAT-D/1, **H. d** Left m1 (NHMW 2005/0114/0003), SHG-AB/17-20. **e** Left m2 (NHMW 2005/0114/0004), SHG-AB/17-20. **f** Right m3 (NHMW 2005/0209/0001), UNCH-A/3. **g** Left mand. m1-3, occlusal view (NHMW 2005/0103/0001), TAT-D/1, **H**. **h** Right mand. i3, c, p2, i2, p4 (NHMW 2005/0103/0002), TAT-D/1. **i** Left D2 (NHMW 2005/0114/0006), SHG-AB/17-20. **j** Left P3 (NHMW 2005/0114/0007), SHG-AB/17-20. **k** Left P4 (NHMW 2005/0114/0008), SHG-AB/17-20. **l** Left M1 (NHMW 2005/0114/0009), SHG-AB/17-20. **m** Right M2 (NHMW 2005/0114/0010), SHG-AB/17-20. **n** Left M3 (NHMW 2005/0114/0012), SHG-AB/17-20

**Fig. 37:**
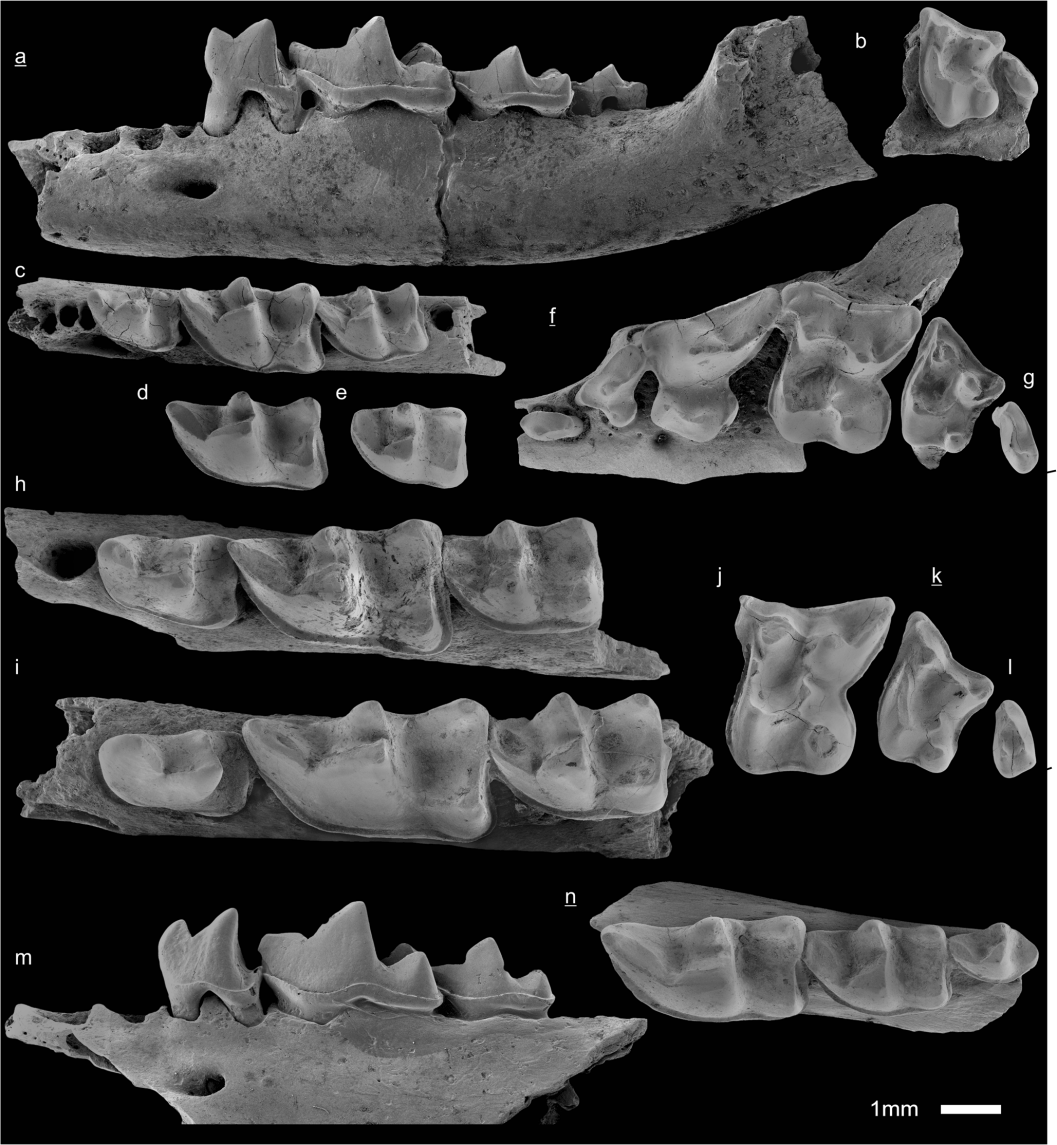
Family Erinaceidae. ***Palaeoscaptor acridens*** (**Matthew and Granger**, **1924**) from Tatal Gol (TAT-D/1; letter zone A), Khongil (HL-A/1+2; letter zone A) and Taatsiin Gol (TGR-AB/21, TGR-AB/22; letter zone B), Valley of Lakes, Mongolia. Early Oligocene. Ziegler et al. (2007). **a** Right mand. p4-m3 (NHMW 2005/0104/0001), TAT-D/1. **b** Left max. M2-3 (NHMW 2005/0133/0006), TGR-AB/22. **c** Left mand. p4-m2 (NHMW 2005/0094/0001), HL-A/1+2. **d** Left m1 (NHMW 2005/0133/0002), TGR-AB/22. **e** Left m2 (NHMW 2005/0133/0003), TGR-AB/22. **f** Right max. P2-M2 (NHMW 2005/0133/0005), TGR-AB/22. **g** Left M3 (NHMW 2005/ 0136/0002), TGR-AB/21. ***Palaeoscaptor*** **cf**. ***rectus*** **Matthew and Granger**, **1924** from Hsanda Gol (SHG-AB/17-20; Biozone B), Ikh Argalatyn Nuruu (IKH-B/5; letter zone C1), Tatal Gol (TAT-C/7; letter zone B), Taatsiin Gol (TGR-C/1+2; letter zone C), and Toglorhoi (TGW-A/2a and TGW-A/2b; Biozone C), Valley of Lakes, Mongolia. Early Oligocene and late Oligocene. Ziegler et al. (2007). **h** Left mand. p4-m2 (NHMW 2005/0115/0001), SHG-AB/17-20. **i** Left mand. p4-m2 (NHMW 2005/0195/0001), IKH-B/5. **j** Left M1 (NHMW 2005/0123/0001), TAT-C/7. **k** Right M2 (NHMW 2005/0160/0001), TGW-A/2b. **l** Left M3 (NHMW 2005/0123/0001), TAT-C/7. **m** Left mand. p4-m2, labial view (NHMW 2005/0154/0001), TGW-A/2a. **n** Right mand. m1-3 (NHMW 2005/0129/0001), TGR-C/1+2

**Fig. 38:**
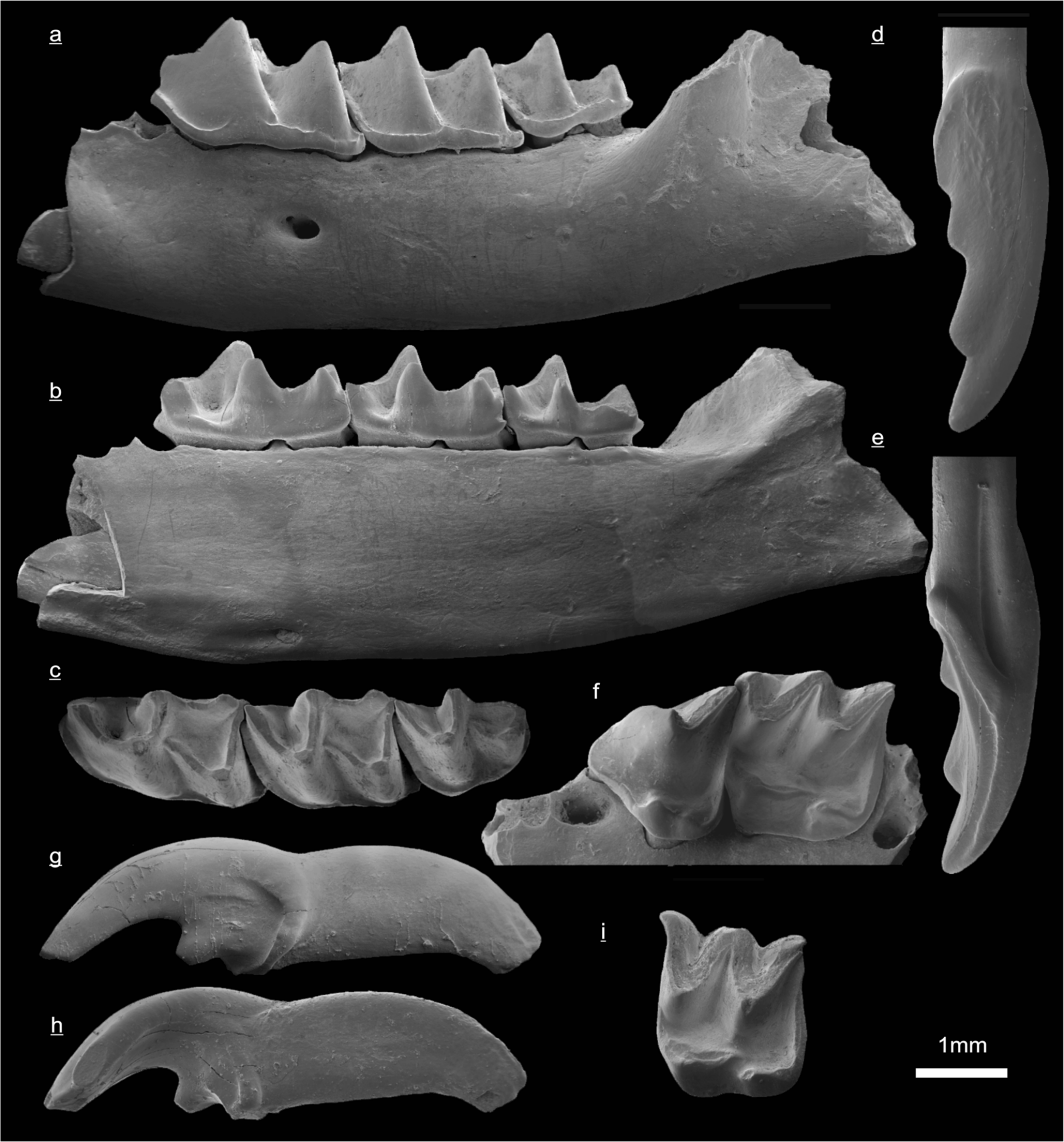
Family Soricidae. ***Gobisorex kingae*** **Sulimski**, **1970** from Taatsiin Gol (TGR-B/1, TGR-AB/22), Ikh Argalatyn Nuruu (IKH-A/2), Hsanda Gol (SHG-AB/17-20), and Unkheltseg (UNCH-A/3B), Valley of Lakes, Mongolia. Early Oligocene, letter zone B. Ziegler et al. (2007). **a** Right mand. m1-2, labial view (NHMW 2006/0027/0001), TGR-B/1. **b** Right mand. m1-2, lingual view (NHMW 2006/0027/0001), TGR-B/1. **c** Right mand. m1-2, occlusal view (NHMW 2006/0027/0001), TGR-B/1. **d** Right inc. inf., labial view (NHMW 2006/0023/0001), IKH-A/2. **e** Right inc. inf., lingual view (NHMW 2006/0023/0001), IKH-A/2. **f** Left max. P4-M1 (NHMW 2006/0025/0001), SHG-AB/17-20. **g** Right Inc. sup., labial view (NHMW 2006/0030/0001), UNCH-A/3B. **h** Right Inc. sup., lingual view (NHMW 2006/0030/0001), UNCH-A/3B. **i** Right M2 (NHMW 2006/0026/0001), TGR-AB/22

**Fig. 39:**
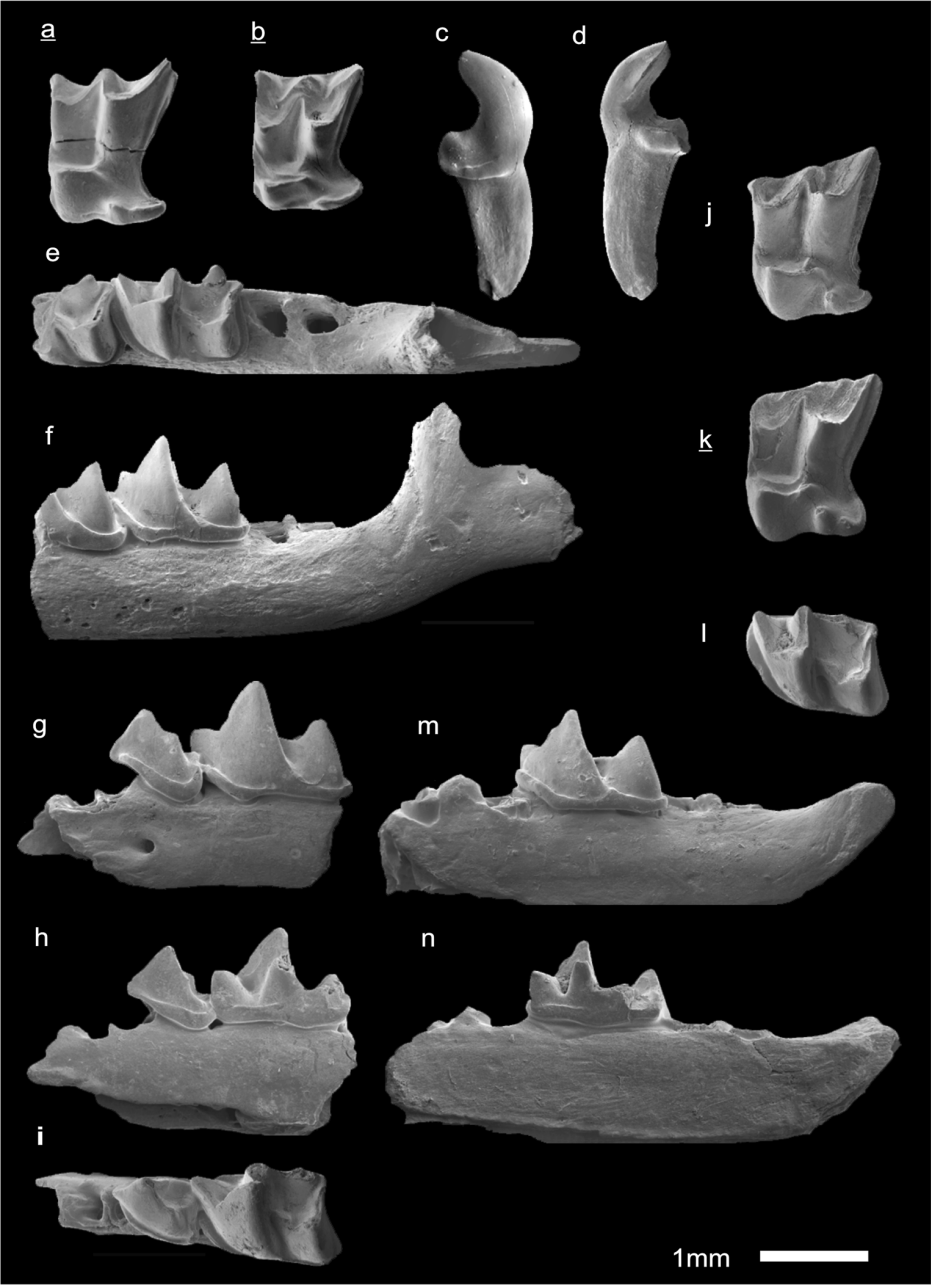
Family Soricidae. ***Taatsiinia hoeckorum*** **Ziegler**, **Dahlmann and Storch**, **2007** from Taatsiin Gol (TGR-B/1), Valley of Lakes, Mongolia. Early Oligocene, letter zone B. All figured specimens are the holotype (H) and paratypes. Ziegler et al. (2007). **a** Right M1 (NHMW 2006 0036/0001), **H**. **b** Right M2 (NHMW 2006/0036/0003). **c** Left I sup, labial view (NHMW 2006/0036/0002). **d** Left I sup, lingual view (NHMW 2006/0036/0002). **e** Left mand. m1-2, occlusal view (NHMW 2006/0036/0004). **f** Left mand. m1-2, labial view (NHMW 2006/0036/0004). ***Tavoonyia altaica*** **Ziegler**, **Dahlmann and Storch**, **2007** from Huch Teeg (RHN-A/9), Valley of Lakes, Mongolia. Late Oligocene, letter zone C1. All figured specimens are the holotype (H) or paratypes. Ziegler et al. (2007). **g** Left mand. p4-m1 labial view (NHMW 2006/0037/0003). **h** Left mand. p4-m1 lingual view (NHMW 2006/0037/0003). **i** Left mand. p4-m1 occlusal view (NHMW 2006/0037/0003). **j** Left M1 (NHMW 2006/0037/0001), **H**. **k** Right M2 (NHMW 2006/0037/0002). **l** Left m2 of mand. (NHMW 2006/0037/0004). **m** Left mand. m2, labial view (NHMW 2006/0037/0004). **n** Left mand. m2, lingual view (NHMW 2006/0037/0004)

**Fig. 40:**
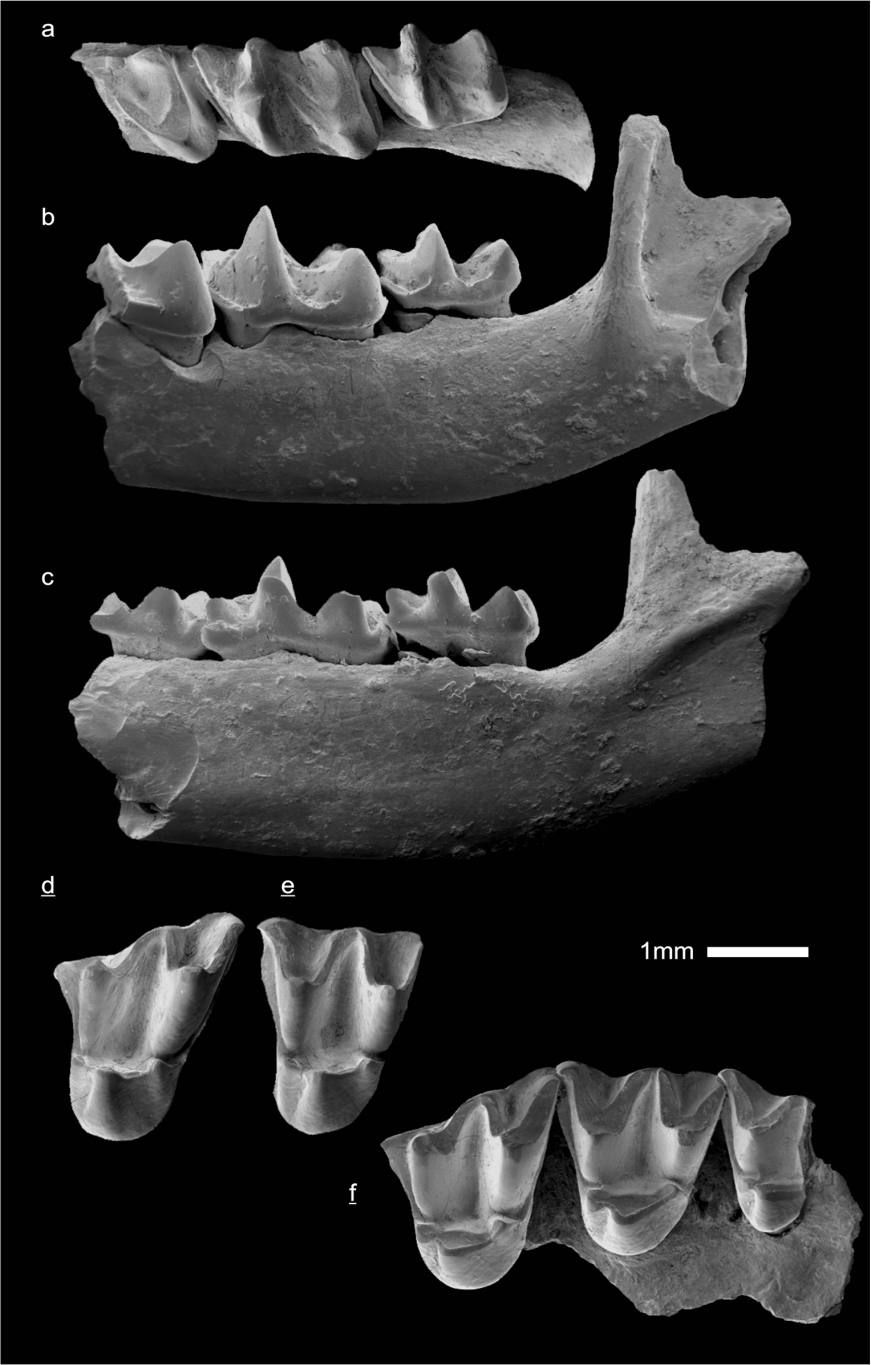
Family Talpidae. ***Mongolopala tathue*** **Ziegler**, **Dahlmann and Storch**, **2007** from Tatal Gol (TAT-D/1) and Hsanda Gol (SHG-C/1), Valley of Lakes, Mongolia. Early Oligocene, letter zone A. Holotype (H), paratypes (P). Ziegler et al. (2007). **a**–**c** Left mand. m1-3 (NHMW 2006/0055/0002), TAT-D/1. **a** Occlusal view, **b** labial view, **c** lingual view. **d** Right M1 (NHMW 2006/0056/0001), SHG-C/1, P. **e** Right M2 (NHMW 2006/0056/0002), SHG-C/1, P. **f** Right Max M1-3 (NHMW 2006/ 0055/0001), TAT-D/1, **H**

**Fig. 41:**
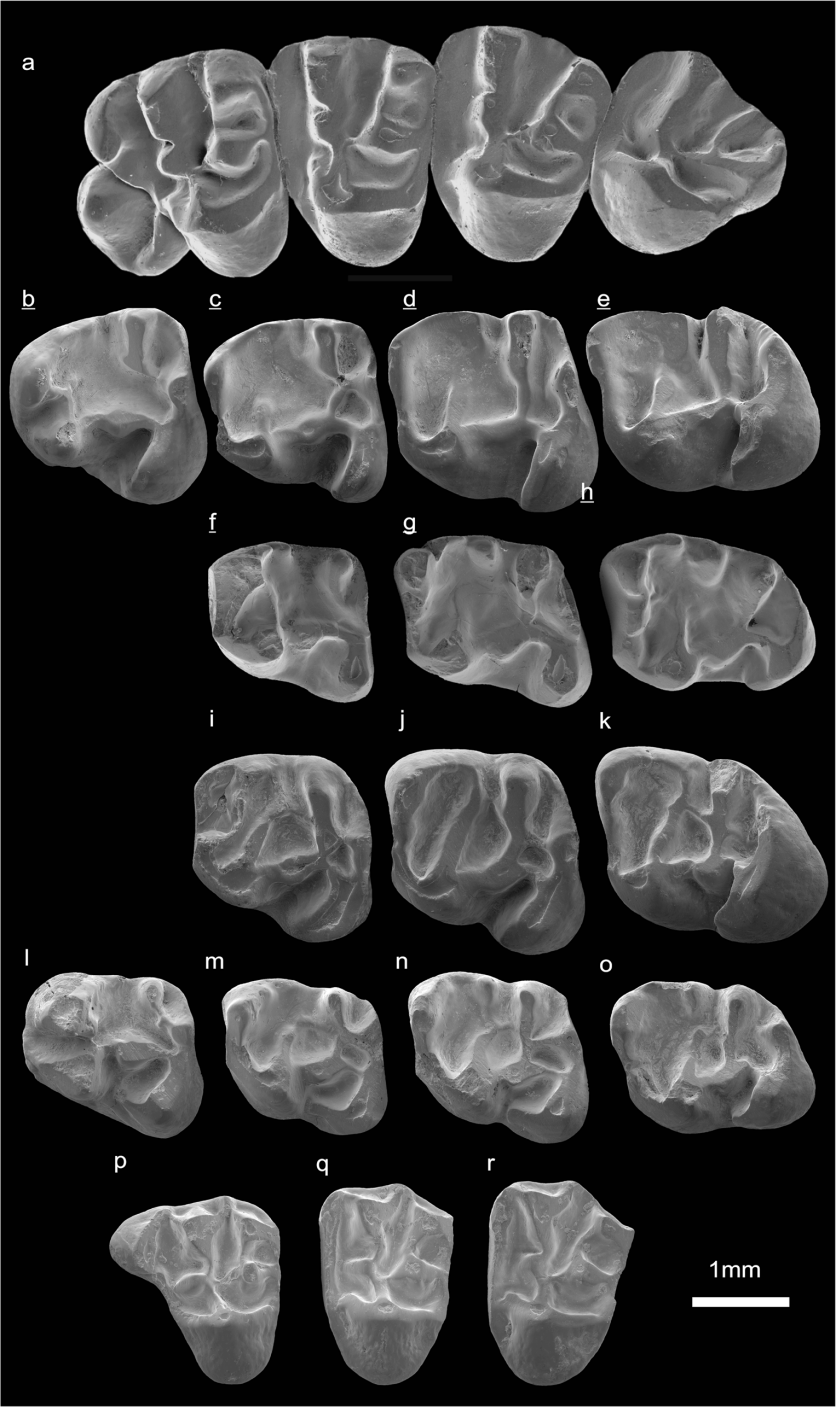
Family Aplodontidae. ***Ninamys kazimierzi*** **Vianey**-**Liaud**, **Gomes Rodrigues and Marivaux**, **2013** from Taatsiin Gol (TGR-B/1) and Ikh Argalatyn Nuruu (IKH-A/1+2), Valley of Lakes, Mongolia. Early Oligocene, letter zone B. Maridet et al. (2017, this issue). **a** Left maxilla P4-P3-M3 (NHMW 2009/0137/0001), TGR-B/1 **b**–**e** Right mand. p4-m3 (NHMW 20115/0358/0001), IKH-A/1+2. **b** Right p4, **c** right m1, **d** right m2, **e** right m3. ***Ninamys arboraptus*** (**Shevyreva**, **1966**) from Taatsiin Gol (TGR-B/1), Valley of Lakes, Mongolia. Early Oligocene, letter zone B. Maridet et al. (2017, this issue).**f**–**h** Right m1-3 (NHMW 2009/0138/0001), TGR-B/1. **f** Right m1, **g** right m2, **h** right m3. ***Prosciurus*** ? ***mongoliensis*** **Wang and Dashzeveg**, **2005** from Tatal Gol (TAT-D/1), Valley of Lakes, Mongolia. Early Oligocene, letter zone A. Maridet et al. (2017, this issue). **i**–**k** Left mand. m1-3 (NHMW 2015/0350/0001). **i** Left m1, **j** left m2, **k** left m3. ***Promeniscomys*** **cf**. ***sinensis*** (**Wang**, **1987**) from Taatsiin Gol (TGR-AB/21, letter zone B) and Tatal Gol (TAT-D/1, letter zone A), Valley of Lakes, Mongolia. Early Oligocene. Maridet et al. (2017, this issue). **l**–**o** Left mand. p4-m3 (NHMW 2015/0366/0001), TGR-AB/21, letter zone B. **l** Left p4, **m** left m1, **n** left m2, **o** left m3. **p**–**r** Left max. P4-M2 (NHMW 2015/0351/0001), TAT-D/1, letter zone A. **p** Left P4, **q** left M1, **r** left M2

**Fig. 42:**
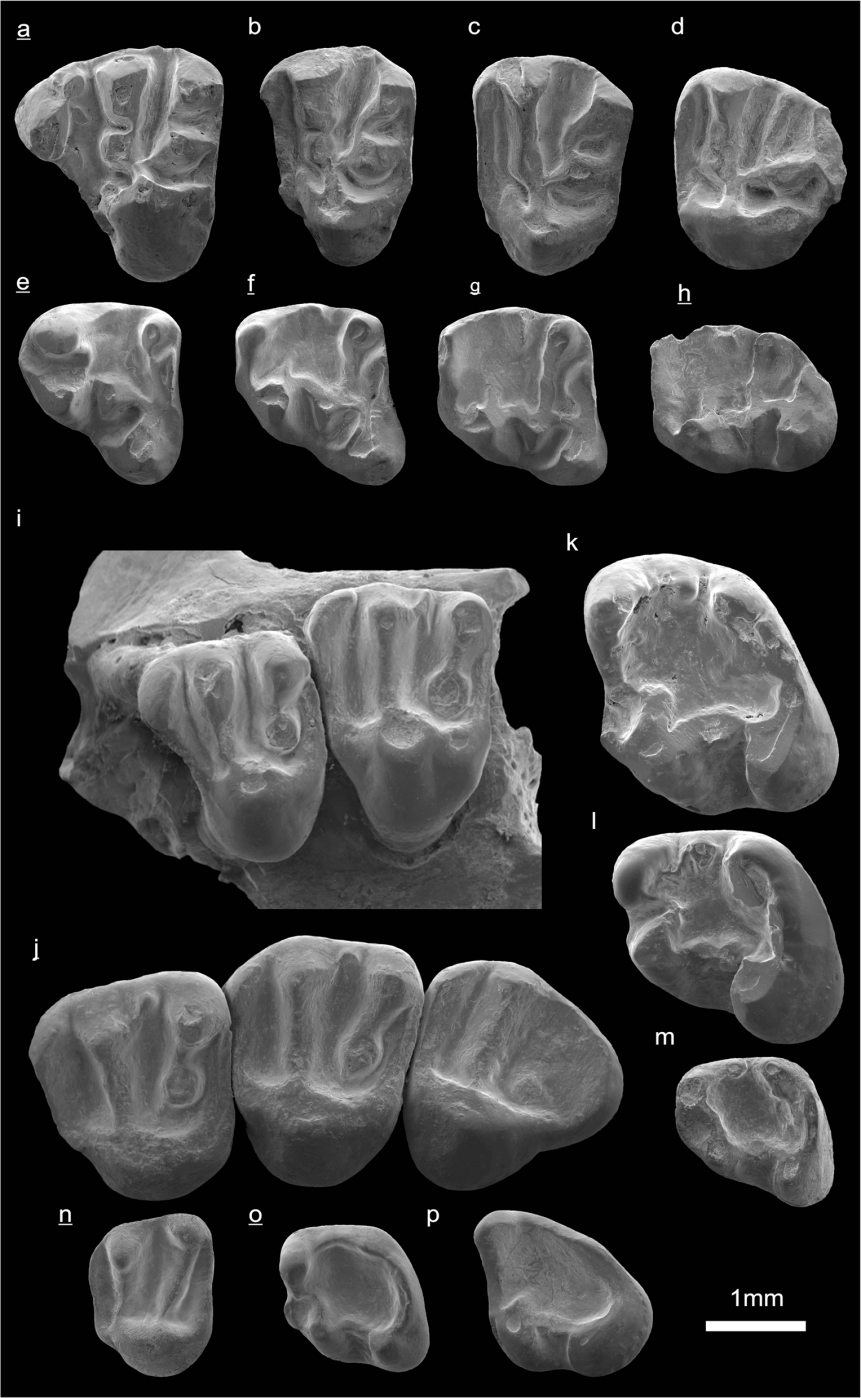
Family Aplodontidae. ***Proansomys badamae*** **sp. nov**.**Maridet, Daxner-Höck, López-Guerrero and Göhlich, 2017** from Taatsiin Gol (TGR-C/1, TGR-C/7), Valley of Lakes, Mongolia. Late Oligocene, letter zone C. Maridet et al. (2017, this issue). **a** Right P4 (NHMW 2015/0381/0001), TGR-C/7. **b** Left M1/2 (NHMW 2015/0381/0002), TGR-C/7. **c** Left M1/2 (NHMW 2015/0381/0003), TGR-C/7. **d** Left M3 (NHMW 2015/0381/0006), TGR-C/7. **e**–**h** Right mand. p4-m3 (NHMW 2015/0374/0002), TGR-C/1. **e** Right p4, **f** right m1, **g** right m2, **h** right m3. Family Sciuridae. ***Kherem shandgoliensis*** **Minjin**, **2004** from Hotuliin Teeg (HTE*), Valley of Lakes, Mongolia. Early Miocene, letter zone D. Maridet et al. (2014a). **i** Left max. P4-M1 (NHMW 2013/0407/0001). **j** Right max. M1-3 (NHMW 2013/0407/0002). **Pteromyini indet**. from Unkheltseg (UNCH-A/4), Valley of Lakes, Mongolia. Early Miocene, letter zone D. Maridet et al. (2014a). **k** Left m1/2 (NHMW 2013/0412/0003). **l** Left p4 (NHMW 2013/0412/0002). **m** Left d4 (NHMW 2013/0412/0001). ***Plesiosciurus*** **aff**. ***sinensis*** **Qiu and Liu**, **1986** from Ulan Tolgoi (UTO-A/5), Valley of Lakes, Mongolia. ?Middle Miocene, letter zone D1/2. Further occurrences are Toglorhoi (TGW-A/2a, Late Oligocene), Hotuliin Teeg (HTE*), and Unkheltseg (UNCH-A/4), early Miocene. Maridet et al. (2014a). **n** Right M1/2 (NHMW 2013/0400/0001), UTO-A/5. **o** Right m1/2 (NHMW 2013/0400/0004), UTO-A/5. **p** Left m3 (NHMW 2013/0400/0005), UTO-A/5

**Fig. 43:**
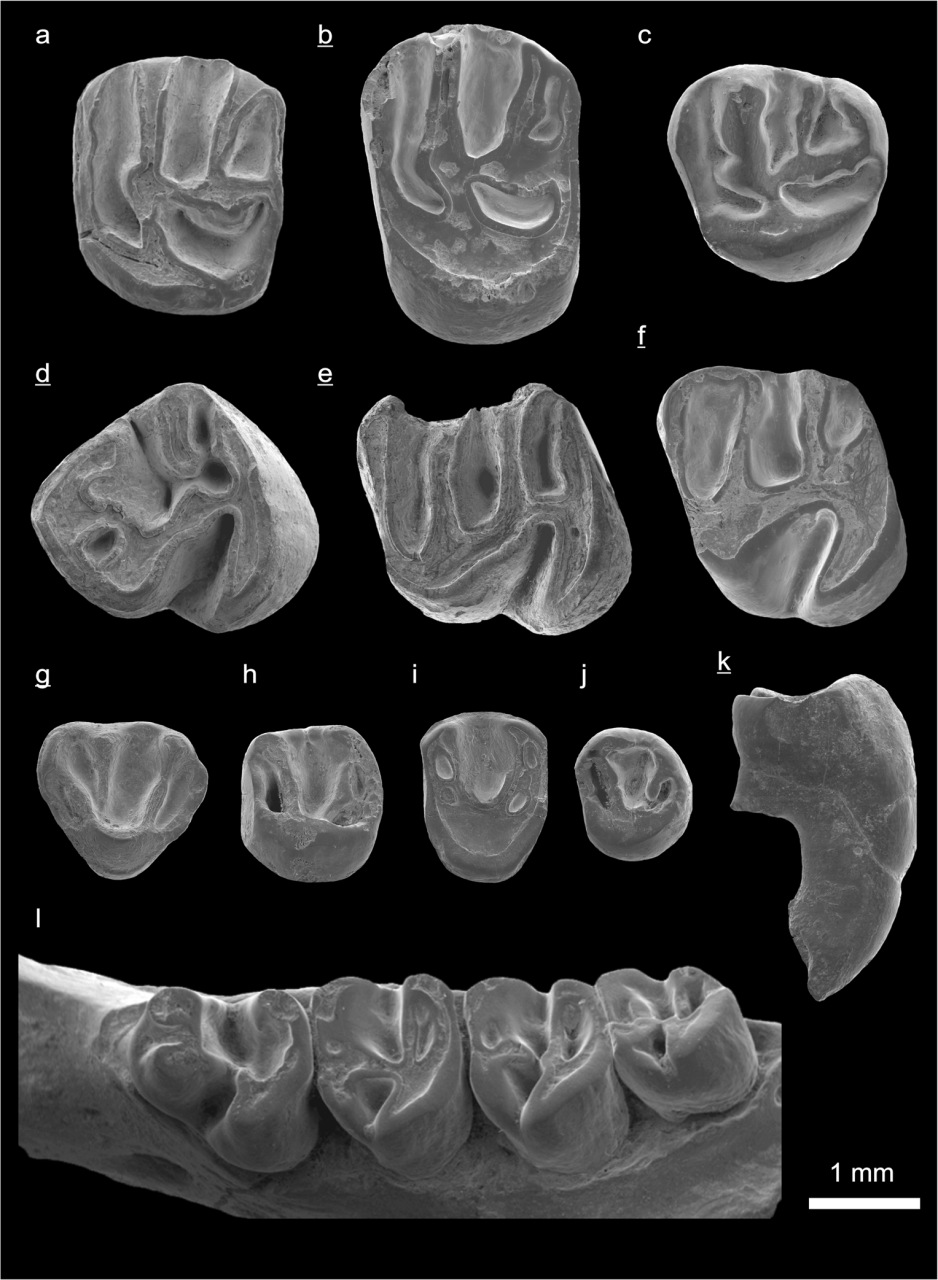
Fam. Cylindrodontidae. ***Anomoemys lohiculus*** (**Matthew and Granger**, **1923**) from Del (DEL-B/7, letter zone B) and Taatsiin Gol (TGR-A/ 13, letter zone A and TGL-A/11, letter zone B), Valley of Lakes, Mongolia. Early Oligocene. Daxner-Höck et al. (2010). **a** Left M1 (NHMW 2009/0140/0002), Del-B/7. **b** Right M2 (NHMW 2016/0015/0001), TGL-A/11. **c** Left M3 (NHMW 2016/0014/0001), TGR-A/13. **d** Right p4 (NHMW 2016/0015/0003), TGL-A/11. **e** Right m1/2 (NHMW 2016/0015/0004), TGL-A/11. **f** Right m2 (NHMW 2009/0140/0003), DEL-B/7. ***Ardynomys*** **sp**. from Tatal Gol (TAT-037 and TAT-D/1), Valley of Lakes, Mongolia. Early Oligocene, letter zone A. Daxner-Höck et al. (2010). **g** Right D4 (NHMW 2016/0017/0002), TAT-037. **h** Left M1/2 (NHMW 2016/0130/0003), TAT-D/1. **i** Left M1/2 (NHMW 2016/0130/0006), TAT-D/1. **j** Left M3 (NHMW 2016/0130/0004), TAT-D/1. **k** Right M1/2, distal view (NHMW 2016/0130/0007), TAT-D/1. **l** Left mand. p4-m3 (NHMW 2016/0017/0001), TAT-037

**Fig. 44:**
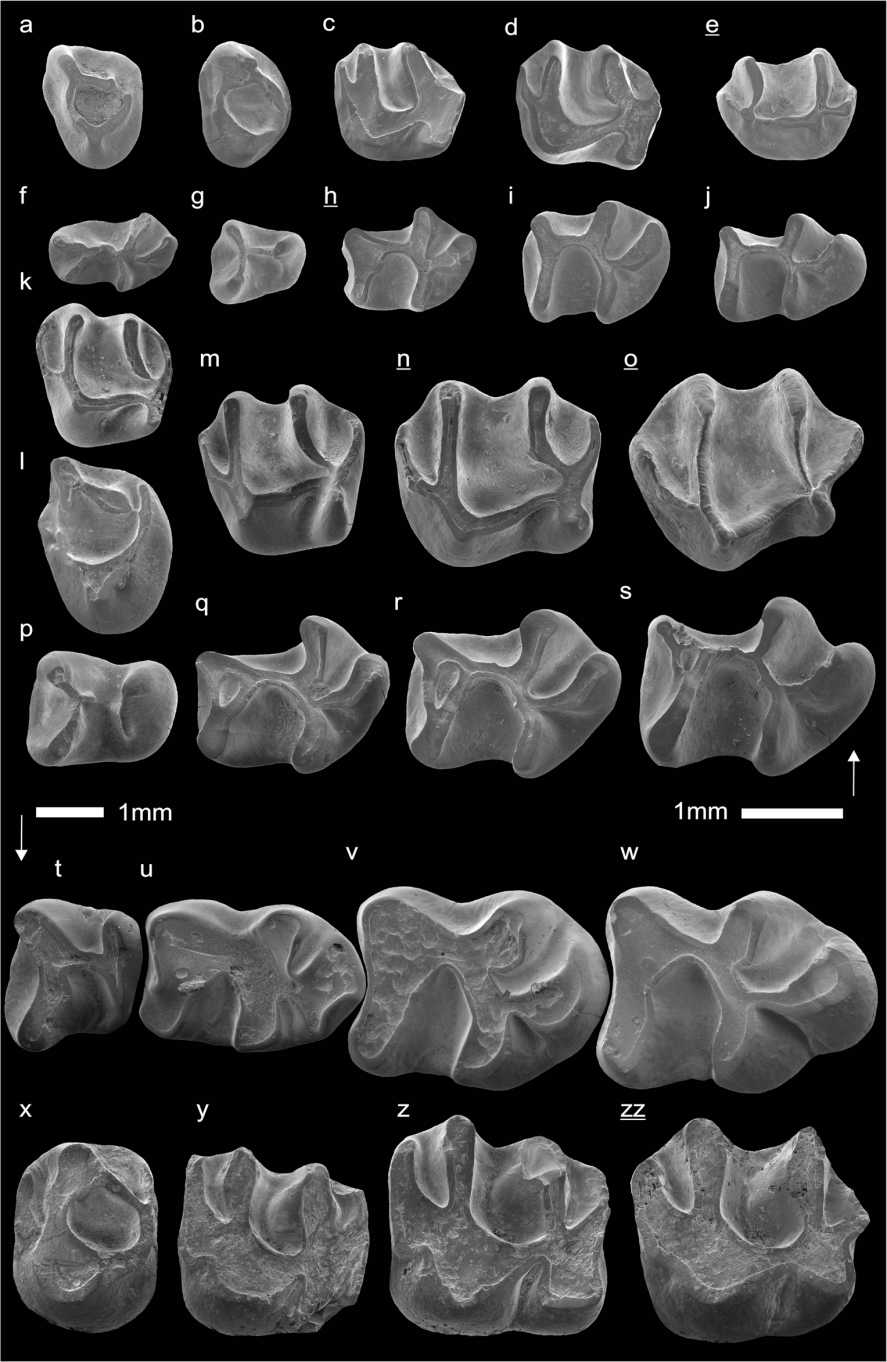
Family Ctenodactylidae. ***Tataromys minor longidens*** **Schmidt**-**Kittler**, **Vianey**-**Liaud and Marivaux**, **2007** from Taatsiin Gol (TGW-A/2b). Late Oligocene, letter zone C. Schmidt-Kittler et al. (2007). **a** Left P4 (NHMW 2006/0100/0013). **b** Left P4 (NHMW 2006/0100/0011). **c** Left M1 (NHMW 2006/0100/0015). **d** Left M2 (NHMW 2006/0100/0020). **e** Right M3 (NHMW 2006/0100/0009). **f** Left d4 (NHMW 2006/0100/0030). **g** Left p4 (NHMW 2006/0100/0031). **h** Right m1 (NHMW 2006/0100/0033). **i** Left m2 (NHMW 2006/0100/0037). **j** Left m3 (NHMW 2006/0100/0038). ***Tataromys sigmodon*** **Matthew and Granger**, **1923** from Toglorhoi (TGW-A/2a, TGW-A/2b), Valley of Lakes, Mongolia. Late Oligocene, letter zone C. Schmidt-Kittler et al. (2007). **k** Left D4 (NHMW 2006/0105/0003)TGW-A/2a. **l** Left P4 (NHMW 2006/0106/0009)TGW-A/2b. **m** Left M1 (NHMW 2006/0105/0005)TGW-A/2a. **n** Right M2 (NHMW 2006/0106/0004)TGW-A/2b. **o** Right M3 (NHMW 2006/0106/0001)TGW-A/2b. **p** Left p4 (NHMW 2006/0105/0011)TGW-A/2a. **q** Left m1 (NHMW 2006/0105/0017)TGW-A/2a. **r** Left m2/3 (NHMW 2006/0105/0018)TGW-A/2a. **s** Left m3 (NHMW 2006/0105/0020)TGW-A/2aw. ***d***. **t**–**w** Left mand. p4-m3 (NHMW 2012/0037/0001), TAT-055. **t** Left p4, **u** left m1, **v** left m2, **w** left m3. **x**–**zz** Maxilla left P4-M2 and right M1-M3 (NHMW 2012/0024/0001), SHG-AB/top. **x** Left P4, **y** left M1, **z** left M2, **zz** right M3

**Fig. 45:**
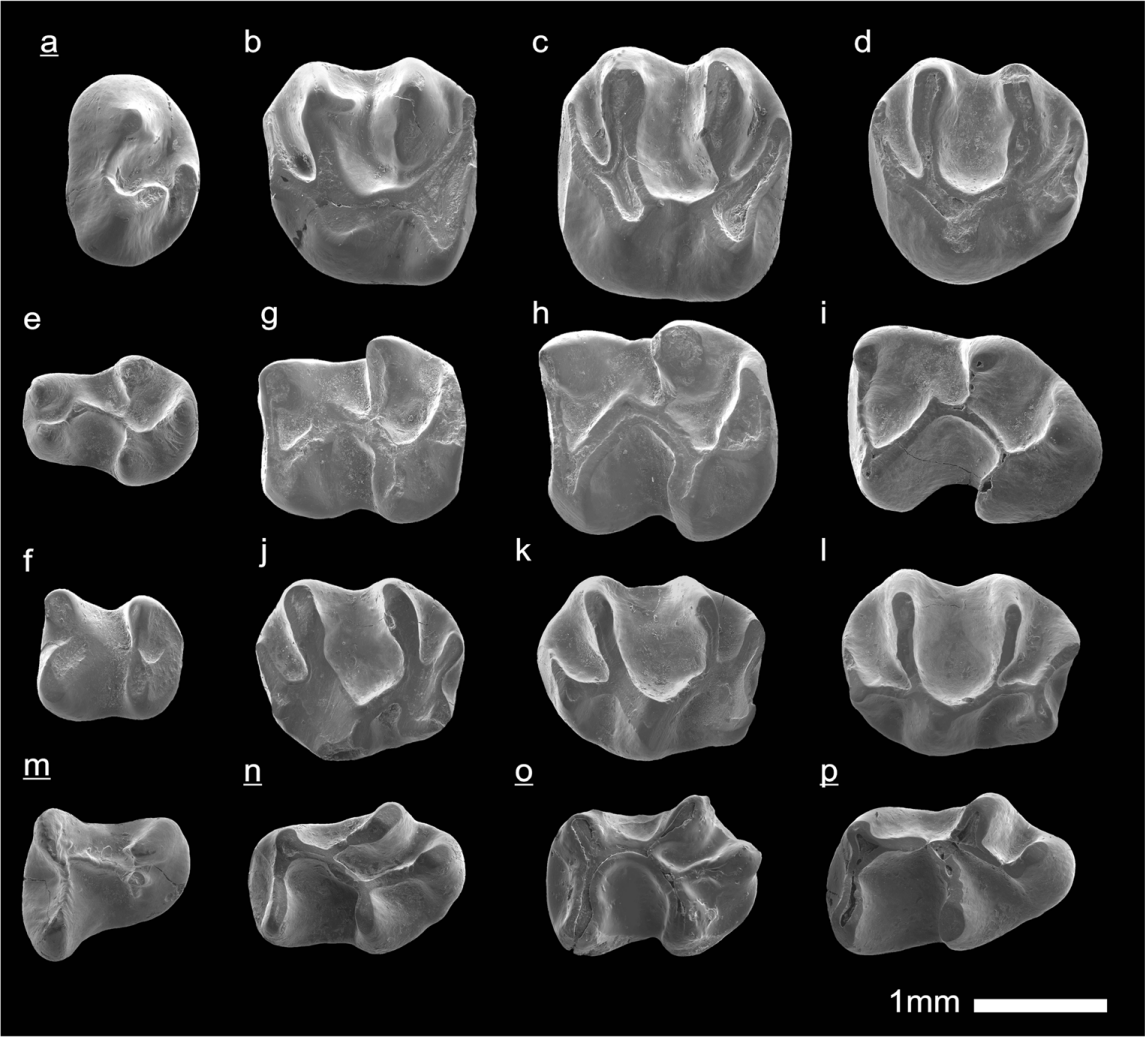
Fam. Ctenodactylidae. ***Karakoromys decessus*** **Matthew and Granger**, **1923** from Hsanda Gol (SHG-A/9, SHG-A*), letter zone B and Khongil (HL-A/1), letter zone A, Valley of Lakes, Mongolia. Early Oligocene. Schmidt-Kittler et al. (2007), Oliver et al. (2017, this issue) **a** Right P4 (NHMW 2012/0021/0002), SHG-A/9. **b** Left M1 (NHMW 2012/0021/0005), SHG-A/9. **c** Left M2 (NHMW 2012/0021/0004), SHG-A/9. **d** Left M3 (NHMW 2012/0059/0012), HL-A/1. **e** Left d4 (NHMW 2012/0059/0013), HL-A/1. **f** Left p4 (NHMW 2012/0022/0003), SHG*. **g** Left m1 (NHMW 2012/0022/0004), SHG*. **h** Left m2 (NHMW 2012/0022/0006), SHG*. **i** Left m3 (NHMW 2012/0059/0014), HL-A/1. ***Huangomys frequens*** **Schmidt**-**Kittler**, **Vianey**-**Liaud and Marivaux**, **2007** from Taatsiin Gol (TGR-AB/22), Valley of Lakes, Mongolia. Early Oligocene, letter zone B. Schmidt-Kittler et al. (2007). **j** Left M1 (NHMW 2006/0075/0016). **k** Left M2 (NHMW 2006/0075/0018). **l** Left M3 (NHMW 2006/0075/0023). **m** Right p4 (NHMW 2006/0075/0029). **n** Right m1 (NHMW 2006/0075/0033). **o** Right m2 (NHMW 2006/0075/0050). **p** Right m2/3 (NHMW 2006/0075/0034)

**Fig. 46:**
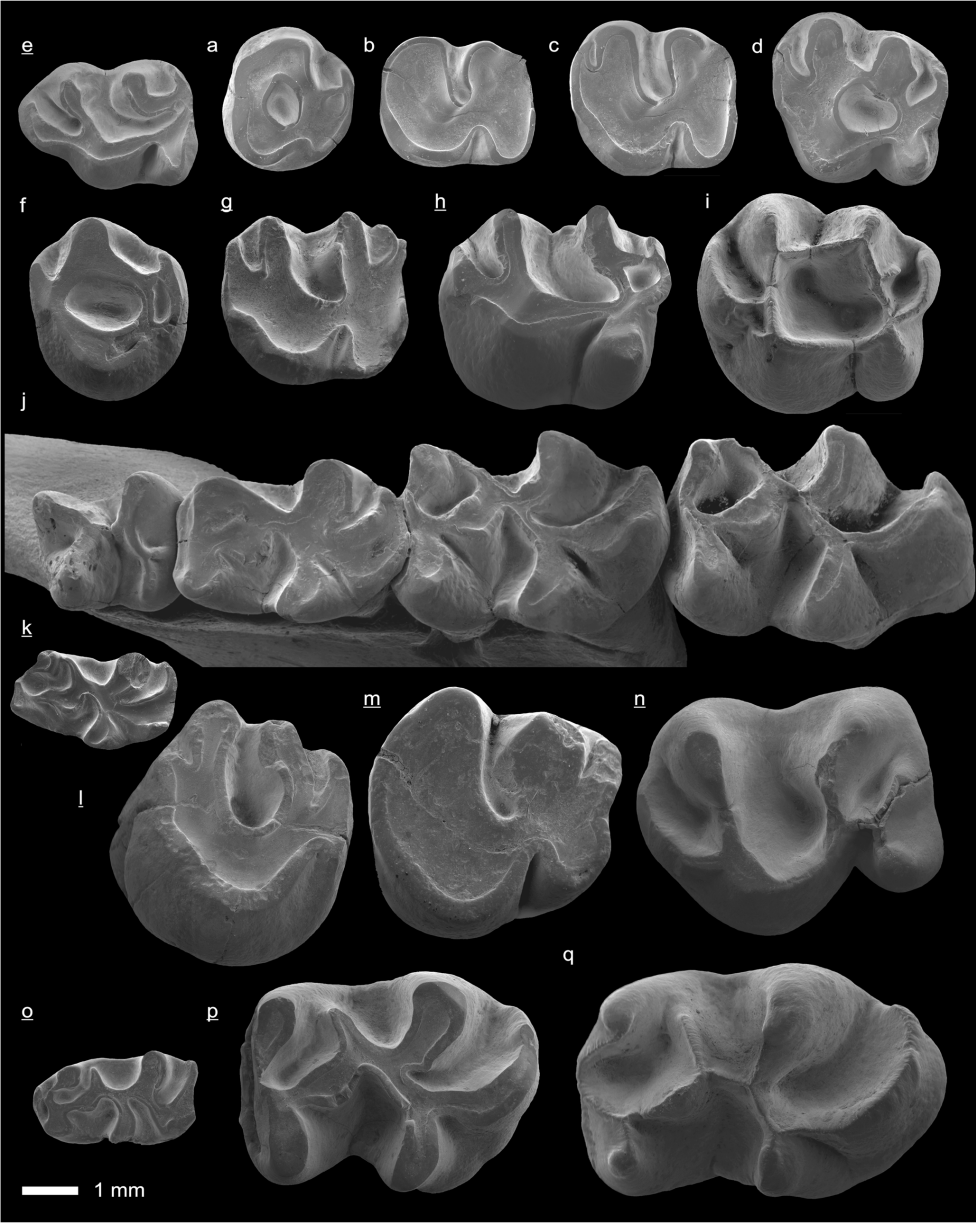
Fam. Ctenodactylidae. ***Yindirtemys birgeri*** **Bendukidze**, **1993** from Tatal Gol (TAT-051/2), Valley of Lakes, Mongolia. Late Oligocene, letter zone C1. Oliver and Daxner-Höck (2016). **a**–**d** Left maxilla right and left P4-M3 (NHMW 2012/0060/0001). **a** Left P4, **b** left M2, **c** left M2, **d** left M3. ***Yindirtemys deflexus*** (**Teilhard de Chardin**, **1926**) from Huch Teeg (RHN-A/7) and Del (DEL-B/12), Valley of Lakes, Mongolia. Late Oligocene, letter zone C1. Schmidt-Kittler et al. (2007), Oliver and Daxner-Höck (2016). **e** Right D4 (NHMW 2006/0090/0015), RHN-A/7. **f** Left P4 (NHMW 2006/0090/0039), RHN-A/7. **g** Left M1 (NHMW 2006/0089/0033), DEL-B/12. **h** Right M2 (NHMW 2006/0090/0053), RHN-A/7. **i** Left M3 (NHMW 2006/0090/0010), RHN-A/7. **j** Left mand. p4-m3 (NHMW 2006/0090/0001), RHN-A/7. **k** Right d4 (NHMW 2006/0090/0021), RHN-A/7 ***Yindirtemys suni*** (**Li and Qiu**, **1980**) from Hotuliin Teeg (HTS-056/3, letter zone C1-D; HTE-014-018, HTE-008, and HTE-009, letter zone D) and Unkheltseg (UNCH-A/4, letter zone D), Valley of Lakes, Mongolia. Late Oligocene and early Miocene. Oliver and Daxner-Höck (2016). **l** Right P4 (NHMW 2012/0031/0012), HTE-014-018. **m** Right M2 (NHMW 2012/0047/0001), HTS-056/3. **n** Right M3 (NHMW 2012/0031/0003), HTE-014-018. **o** Left d4 (NHMW 2006/0088/0001), UNCH-A/4. **p** Right m2 (NHMW 2012/0032/0005), HTE-008. **q** Left m3 (NHMW 2012/0033/0009), HTE-009

**Fig. 47:**
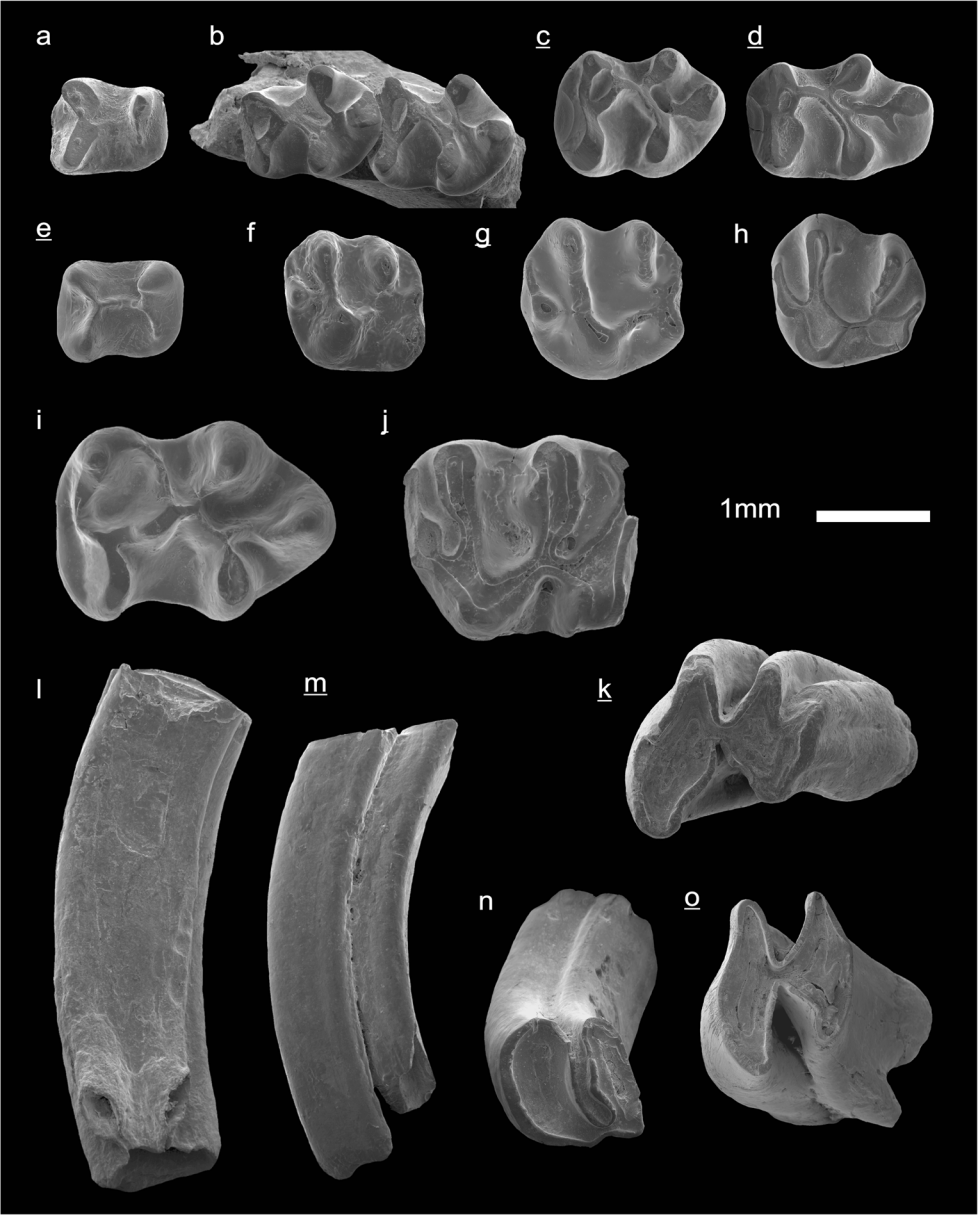
Fam. Ctenodactylidae. ***Yindirtemys shevyrevae*** **Vianey**-**Liaud**, **Schmidt**-**Kittler and Marivaux**, **2006** from Hsanda Gol (SHG-AB/17-18, SHG-AB/17-20) and Taatsiin Gol (TGR-AB/22, TGR-AB/21), Valley of Lakes, Mongolia. Early Oligocene, letter zone B. Schmidt-Kittler et al. (2007). **a** Left p4 (NHMW 2006/0094/0002), SHG-AB/17-20. **b** Left m1-2 (NHMW 2006/0094/0001), SHG-AB/17-20. **c** Right m2 (NHMW 2006/0095/0001), SHG-AB/17-18. **d** Right m3 (NHMW 2006/0097/0001), TGR-AB/22. **e** Right p4 (NHMW 2006/0096/0007), TGR-AB/21. **f** Left M1 (NHMW 2006/0096/0001), TGR-AB/21. **g** Right M2 (NHMW 2006/0096/0012), TGR-AB/21. **h** Left M3 (NHMW 2006/0096/0003), TGR-AB/21. ***Yindirtemys*** **aff**. ***ulantatalensis*** (**Huang**, **1985**) from Unzing Khurem (TAR-A/2), Valley of Lakes, Mongolia. Late Oligocene, letter zone C. Schmidt-Kittler et al. (2007). **i** Left m3 (NHMW 2006/0091/0001), TAR-A/2. **j** Right M2 (NHMW 2006/0091/0002), TAR-A/2. ***Prodistylomys mongoliensis*** **nov. spec**. from Hotuliin Teeg (HTE-012), Valley of Lakes, Mongolia. Early Miocene, letter zone D. Oliver et al. (in prep.). **k** Right m2, occlusal view (NHMW 2012/0051/0001), HTE-012 ***Prodistylomys taatsiini*** **nov. spec**. from Huch Teeg (RHN-A/12), Luugar Khudag (LOG-A/1) and Unkheltseg (UNCH-A/3), Valley of Lakes, Mongolia. Early Miocene, letter zone D. Oliver et al. (2016). **l** Left M1/2, distal view (NHMW 2012/0050/0004), RHN-A/12. **m** Right M1/2, lingual view (NHMW 2012/0048/0002), LOG-A/1. **n** Left M1/2, occlusal-labial view (NHMW 2012/0049/0005), UNCH-A/3. **o** Right m2, occlusal view (NHMW 2012/0048/0001), LOG-A/1

**Fig. 48:**
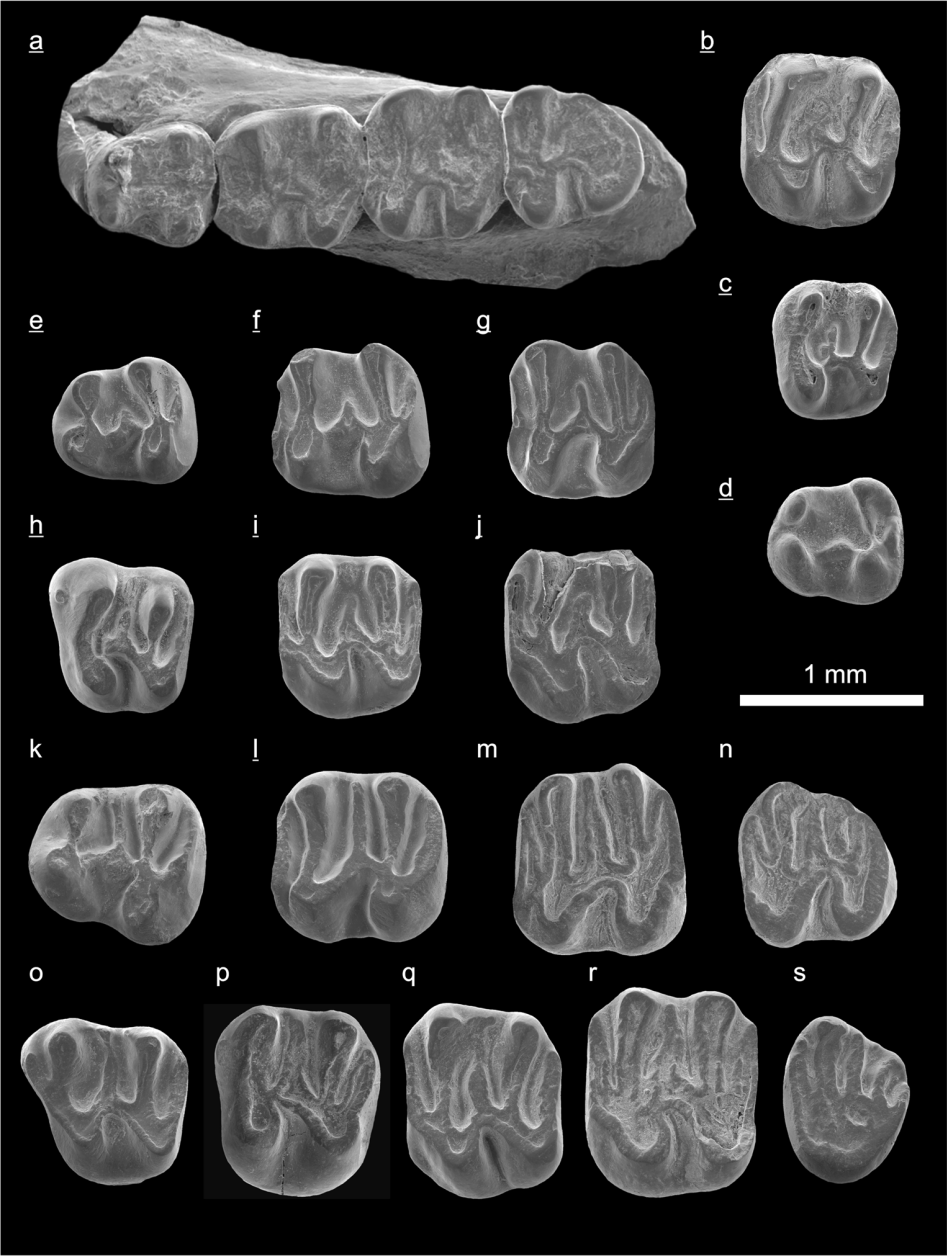
Fam. Eomyidae. ***Eomys*** **aff**. ***orientalis*** **Wang and Emry**, **1991** from Taatsiin Gol (TGR-AB/21) and Hsanda Gol (SHG-A/17-18), Valley of Lakes, Mongolia. Early Oligocene, letter zone B. Maridet et al. (2015). **a** Right mand. p4-m3 (NHMW 2013/0059/0001), TGR-AB/21. **b** Right M1/2 (NHMW 2013/0055/0004), SHG-A/17-18. **c** Right P4 (NHMW 2013/0055/0002), SHG-A/17-18 ***E***. **cf**. ***orientalis*** **Wang and Emry**, **1991** from Tatal Gol (TAT-C/1), Valley of Lakes, Mongolia. Early Oligocene, letter zone A. Maridet et al. (2015). **d** Right p4 (NHMW 2013/0054/0001) **cf**. ***Asianeomys bolligeri*** (**Lopatin**, **2000**) from Toglorhoi (TGW-A/2b), Valley of Lakes, Mongolia. Late Oligocene, letter zone C. Maridet et al. (2015). **e** Right p4 (NHMW 2013/0064/0005). **f** Right m1/2(NHMW 2013/0064/0006). **g** Right m1/2(NHMW 2013/0064/0007). **h** Right P4 (NHMW 2013/0064/0001). **i** Right M1/2 (NHMW 2013/0064/0003). **j** Right M1/2 (NHMW 2013/0064/0004) ***Asianeomys dangheensis*** (**Wang**, **2002**) from Unkheltseg (UNCH-A/3) and Hotuliin Teeg (HTE-12/5), Valley of Lakes, Mongolia. Early Miocene, letter zone D. Maridet et al. (2015). **k** Left p4 (NHMW 2013/0073/0007), UNCH-A/3. **l** Right m1/2 (NHMW 2013/0073/0008), UNCH-A/3. **m** Left m1/2 (NHMW 2013/0073/0009), UNCH-A/3. **n** Left m3 (NHMW 2013/0073/0010), UNCH-A/3. **o** Left D4 (NHMW 2013/0073/0001), UNCH-A/3. **p** Left P4 (NHMW 2013/0073/0003), UNCH-A/3. **q** Left M1/2 (NHMW 2013/0073/0004), UNCH-A/3. **r** Left M1/2 (NHMW 2013/0073/0005), UNCH-A/3. **s** Left M3 (NHMW 2013/0070/0017), HTE-12/5 (= HTE-005)

**Fig. 49:**
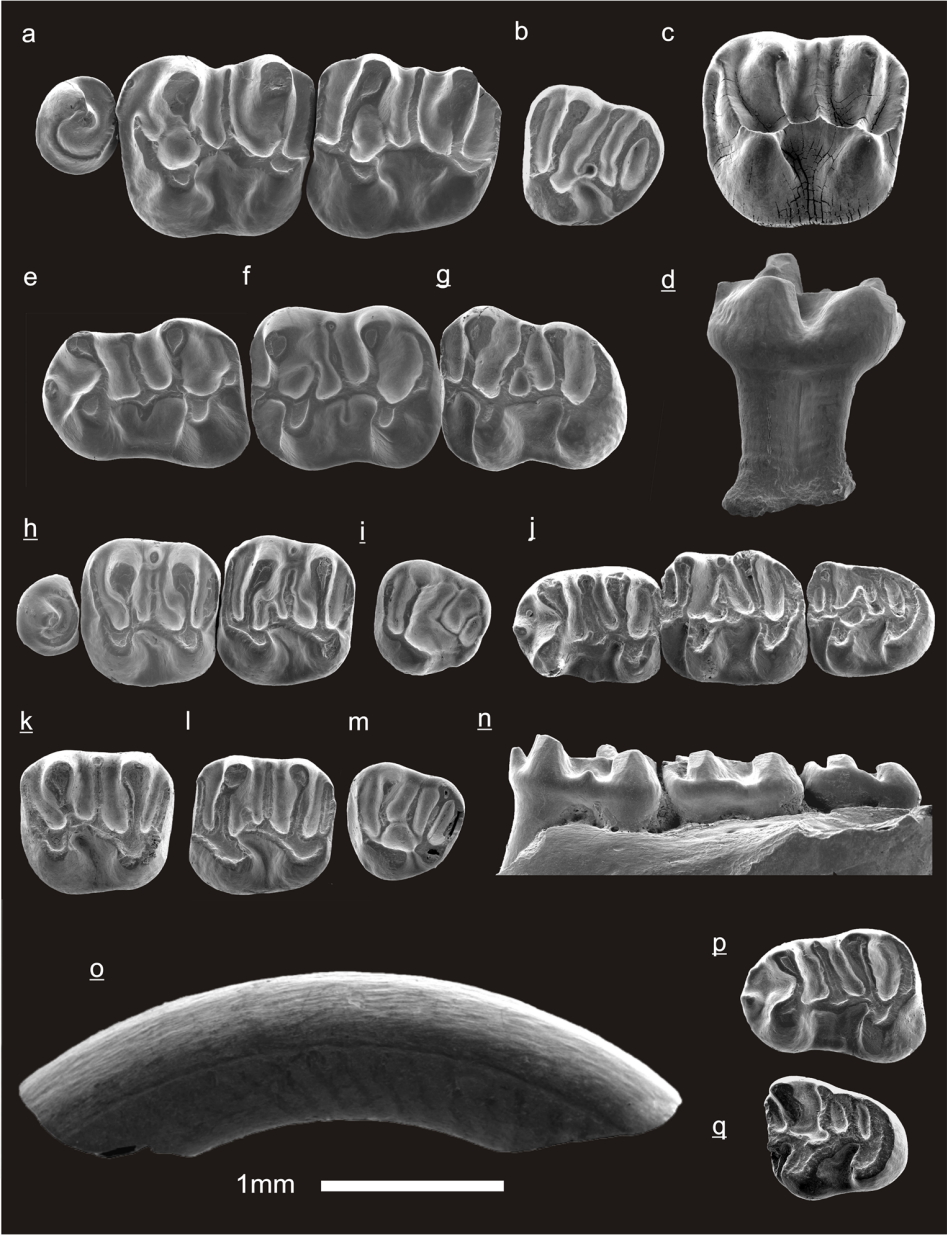
Fam. Dipodidae. ***Heosminthus chimidae*** **Daxner**-**Höck**, **Badamgarav and Maridet**, **2014** from Taatsiin Gol (TGR-B/1), Valley of Lakes, Mongolia. Early Oligocene, letter zone B. All figured specimens are the holotype (H) and paratypes. Daxner-Höck et al. (2014). **a** Left P4-M2 (NHMW 2013/0128/0001), **H. b** Left M3 (NHMW 2013/0128/0006). **c** Left M1 (NHMW 2013/0128/0007). **d** Right M1, lingual (NHMW 2013/0128/0003). **e** Left m1 (NHMW 2013/0128/0010). **f** Left m2 (NHMW 2013/0128/0014). **g** Right m3 (NHMW 2013/0128/0015) ***Heosminthus borrae*** **Daxner**-**Höck**, **Badamgarav and Maridet**, **2014** from Unkheltseg (UNCH-A/3B; letter zone B) and Huch Teeg (RHN-A/12; letter zone D), Valley of Lakes, Mongolia. Early Oligocene (UNCH-A/3B) and early Miocene (RHN-A/12). Holotype (H). Daxner-Höck et al. (2014). **h** Right P4-M2 (NHMW 2013/0117/0001), UNCH-A/3B, **H**. **i** Right M3 (NHMW 2013/0117/0005), UNCH-A/3B. **j** Right m1-3 (NHMW 2013/0127/0004), RHN-A/12. **k** Right M1 (NHMW 2013/0127/0001), RHN-A/12. **l** Left M2 (NHMW 2013/0127/0002), RHN-A/12. **m** Left M3 (NHMW 2013/0117/0006), UNCH-A/3B. **n** Right m1-3 (NHMW 2013/0117/0007), UNCH-A/3B. **o** Right Inc. sup. (NHMW 2013/0127/0005), RHN-A/12. **p** Right m1 (NHMW 2013/0117/0008), UNCH-A/3B. **q** Right m3 (NHMW 2013/0117/0013), UNCH-A/3B

**Fig. 50:**
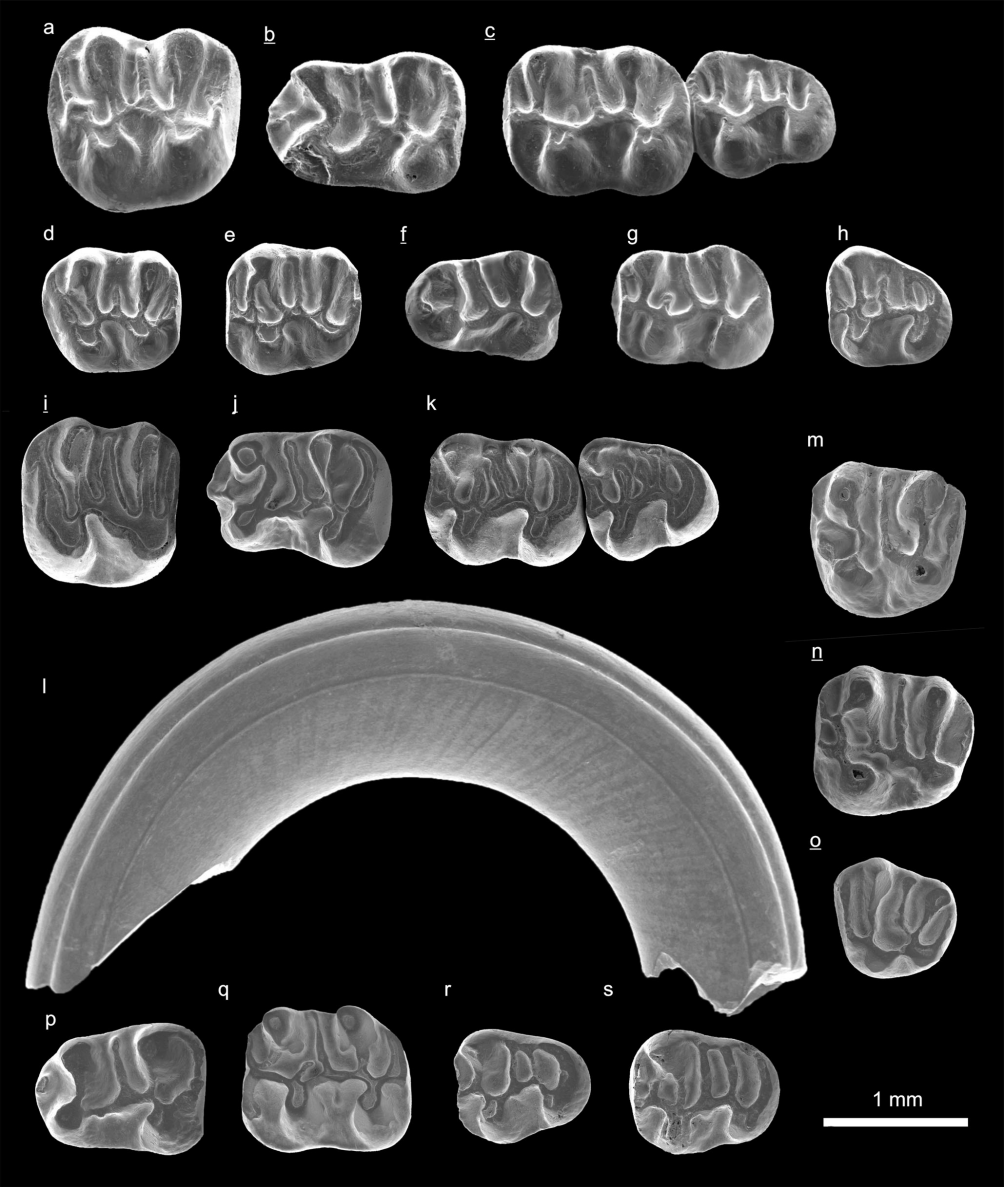
Fam. Dipodidae. ***Plesiosminthus asiaticus*** **Daxner**-**Höck and Wu**, **2003** from Huch Teeg (RHN-A/7), Valley of Lakes, Mongolia. Late Oligocene, letter zone C1. Daxner-Höck and Wu (2003), Daxner-Höck et al. (2014). **a** Left M1 (NHMW 2001/0064/0001/2). **b** Right m1 (NHMW 2001/0064/0001/7). **c** Right m2-3 (NHMW 2001/0064/0001/9) ***Plesiosminthus promyarion*** **Schaub**, **1930** from Huch Teeg (RHN-A/9) and Hotuliin Teeg (HTS-056/2), Valley of Lakes, Mongolia. Late Oligocene letter zone C1-D. Daxner-Höck and Wu (2003), Daxner-Höck et al. (2014). **d** Left M1 (NHMW 2013/0175/0001), HTS-056/2. **e** Left M2 (NHMW 2013/0175/0002), HTS-056/2. **f** Right m1 (NHMW 2001/0065/0001/7), RHN-A/9. **g** Left m2 (NHMW 2001/0065/0001/8), RHN-A/9. **h** Left m3 (NHMW 2013/0175/0004), HTS-056/2. ***Plesiosminthus barsboldi*** **Daxner**-**Höck and Wu**, **2003** from Unkheltseg (UNCH-A/3), Valley of Lakes, Mongolia. Early Miocene, letter zone D. All figured specimens are paratypes. Daxner-Höck and Wu (2003), Daxner-Höck et al. (2014). **i** Right M1 (NHMW 2001/0066/0002/7). **j** Right m1 (NHMW 2001/0066/0002/13). **k** Left m2-3 (NHMW 2001/0066/0002/19). ***Plesiosminthus olzi*** **Daxner**-**Höck**, **Badamgarav and Maridet**, **2014** from Hotuliin Teeg (HTE-005 and HTE-008), Valley of Lakes, Mongolia. Early Miocene, letter zone D. Holotype (H). Daxner-Höck et al. (2014). **l** Left Inc. sup. (NHMW 2013/0176/0009), HTE-008. **m** Left M1 (NHMW 2013/0176/0001), HTE-008, **H**. **n** Right M2 (NHMW 2013/0176/0005), HTE-008. **o** Right M3 (NHMW 2013/0176/0003), HTE-008. **p** Left m1 (NHMW 2013/0176/0006), HTE-008. **q** Left m2 (NHMW 2013/0177/0001), HTE-005. **r** Left m3 (NHMW 2013/0176/0008), HTE-008. **s** Left m3 (NHMW 2013/0176/0007), HTE-008

**Fig. 51:**
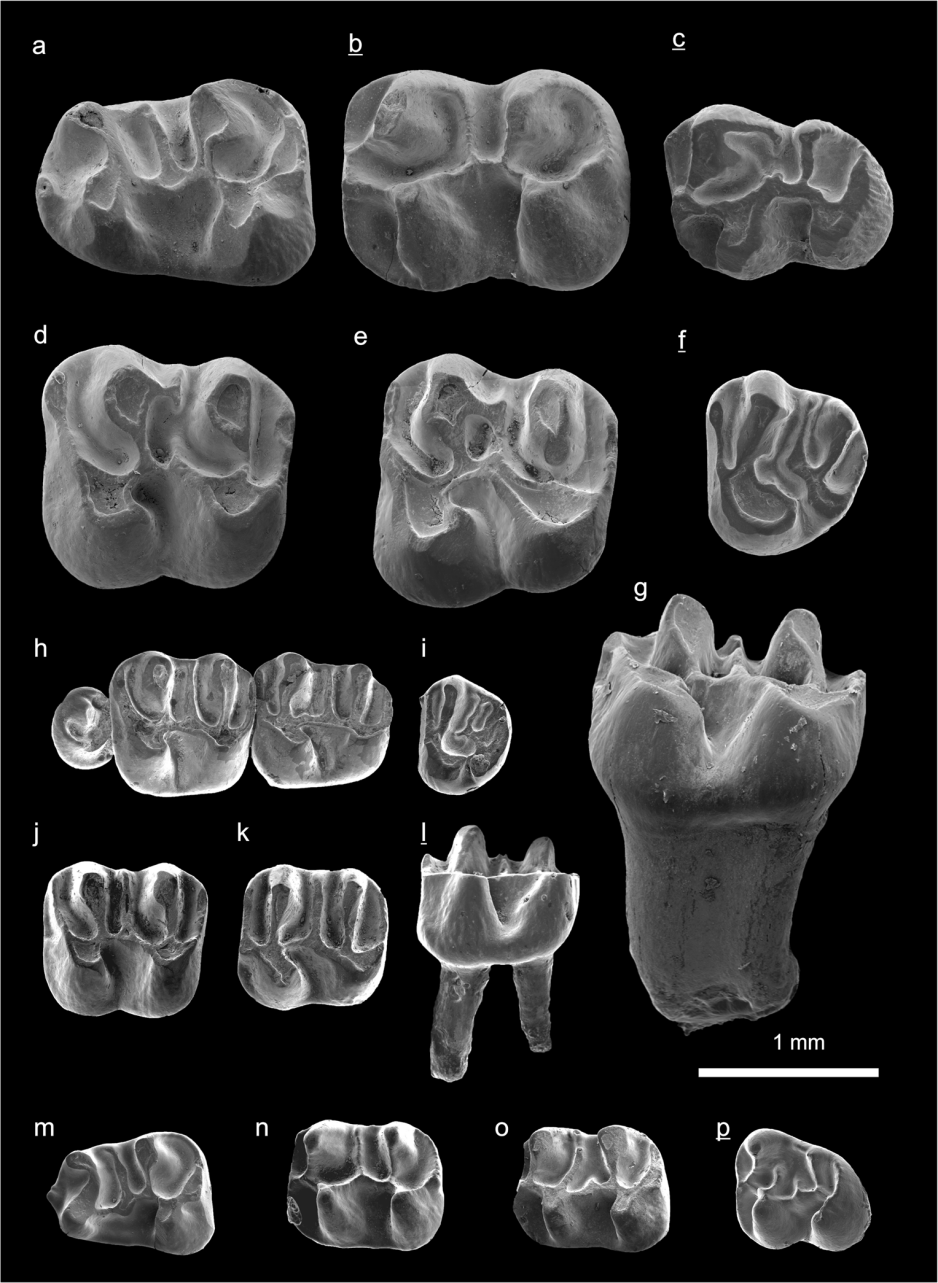
Fam. Dipodidae. ***Onjosminthus baindi*** **Daxner**-**Höck**, **Badamgarav and Maridet**, **2014** from Taatsiin Gol (TGR-A/13+14, TGL-A/2), Tatal Gol (TAT-D/1), and Hsanda Gol (SHG-C/1), Valley of Lakes. Early Oligocene, letter zone A. Paratype (P). Daxner-Höck et al. (2014). **a** Left m1 (NHMW 2013/0180/0003), TGR-A/13. **b** Right m2 (NHMW 2013/0181/0004), TGL-A/2. **c** Right m3 (NHMW 2013/0179/0005), P, TAT-D/1. **d** Left M1 (NHMW 2013/0180/0002), TGR-A/14. **e** Left M2 (NHMW 2013/0181/0001), TGL-A/2. **f** Right M3 (NHMW 2013/0179/0002), P, TAT-D/1, **P**. **g** Left M1, lingual view (NHMW 2013/0183/0001), SHG-C/1. ***Bohlinosminthus parvulus*** **Lopatin**, **1999** from Toglorhoi (TGW-A/2a+b; letter zone C), Hsanda Gol (SHG-AB/17-20; letter zone B), Taatsiin Gol (TGR-C/1; letter zone C), and Tatal Gol (TAT-C/1; letter zone A), Valley of Lakes. Early to late Oligocene. Daxner-Höck et al. (2014). **h** Left P4-M2 (NHMW 2013/0211/0001), TGW-A/2b. **i** Left M3 (NHMW 2013/0205/0002), SHG-AB/17-20. **j** Left M1 (NHMW 2013/0210/0001), TGW-A/2a. **k** Left M2 (NHMW 2013/0210/0002), TGW-A/2a. **l** Right M2, lingual view (NHMW 2013/0203/0002), TAT-C/1. **m** Left m1 (NHMW 2013/0210/0005), TGW-A/2a. **n** Left m2 (NHMW 2013/0205/0005), SHG-AB/17-20. **o** Left m2 (NHMW 2013/0206/0003), TGR-C/1. **p** Right m3 (NHMW 2013/0211/0004), TGW-A/2b

**Fig. 52:**
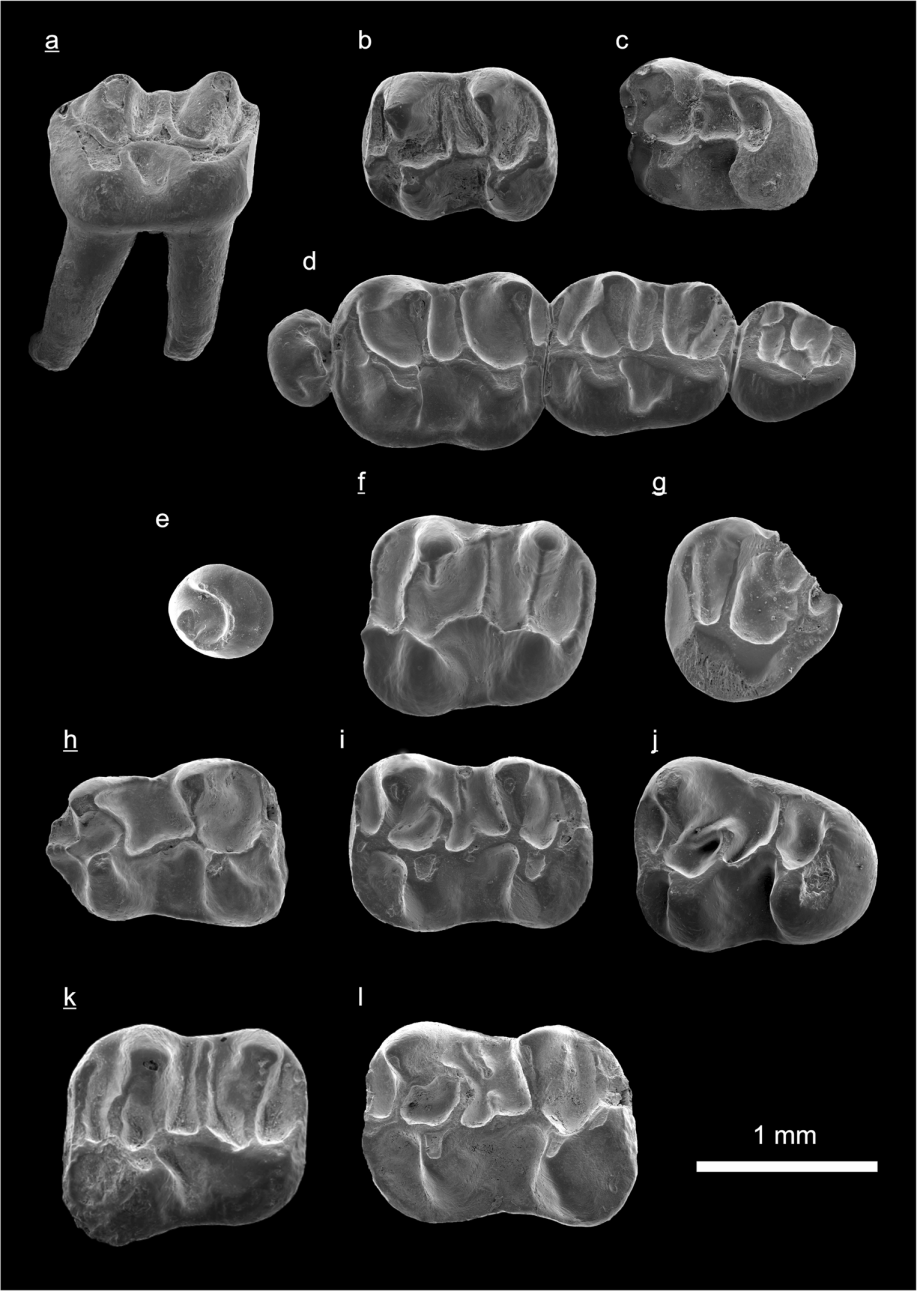
Fam. Dipodidae. ***Parasminthus debruijni*** **Lopatin**, **1999** from Taatsiin Gol (TGR-C/1; letter zone C), Del (DEL-B/12; letter zone C1), and Tatal Gol (TAT-E/27; letter zone C1), Valley of Lakes. Late Oligocene. Daxner-Höck et al. (2014). **a** Right M1, lingual view (NHMW 2013/0199/0001),TGR-C/1. **b** Left m2 (NHMW 2013/0200/0003),DEL-B/12. **c** Left m3 (NHMW 2013/0199/0002),TGR-C/1. **d** Left max. P4-M3 (NHMW 2013/0198/0001),TAT-E/27. ***Parasminthus*** **cf**. ***tangingoli*** **Bohlin**, **1946** from Taatsiin Gol (TGR-C/1; letter zone C) and Del (DEL-B/12; letter zone C1), Valley of Lakes. Late Oligocene. Daxner-Höck et al. (2014). **e** Left P4 (NHMW 2013/0192/0001),TGR-C/1. **f** Right M2 (NHMW 2013/0192/0002),TGR-C/1. **g** Right M3 (NHMW 2013/0196/0002), DEL-B/12. **h** Right m1 (NHMW 2013/0192/0003),TGR-C/1. **i** Left m2 (NHMW 2013/0192/0005),TGR-C/1. **j** Right m3 (NHMW 2013/0192/0006),TGR-C/6. ***Parasminthus*** **cf**. ***asiaecentralis*** **Bohlin**, **1946** from Unzing Khurem (TAR-A/2; letter zone C) and Del (DEL-B/12; letter zone C1), Valley of Lakes. Late Oligocene. Daxner-Höck et al. (2014). **k** Right M2 (NHMW 2013/0263/0001),TAR-A/2. **l** Left m2 (NHMW 2013/0191/0002), DEL-B/12

**Fig. 53:**
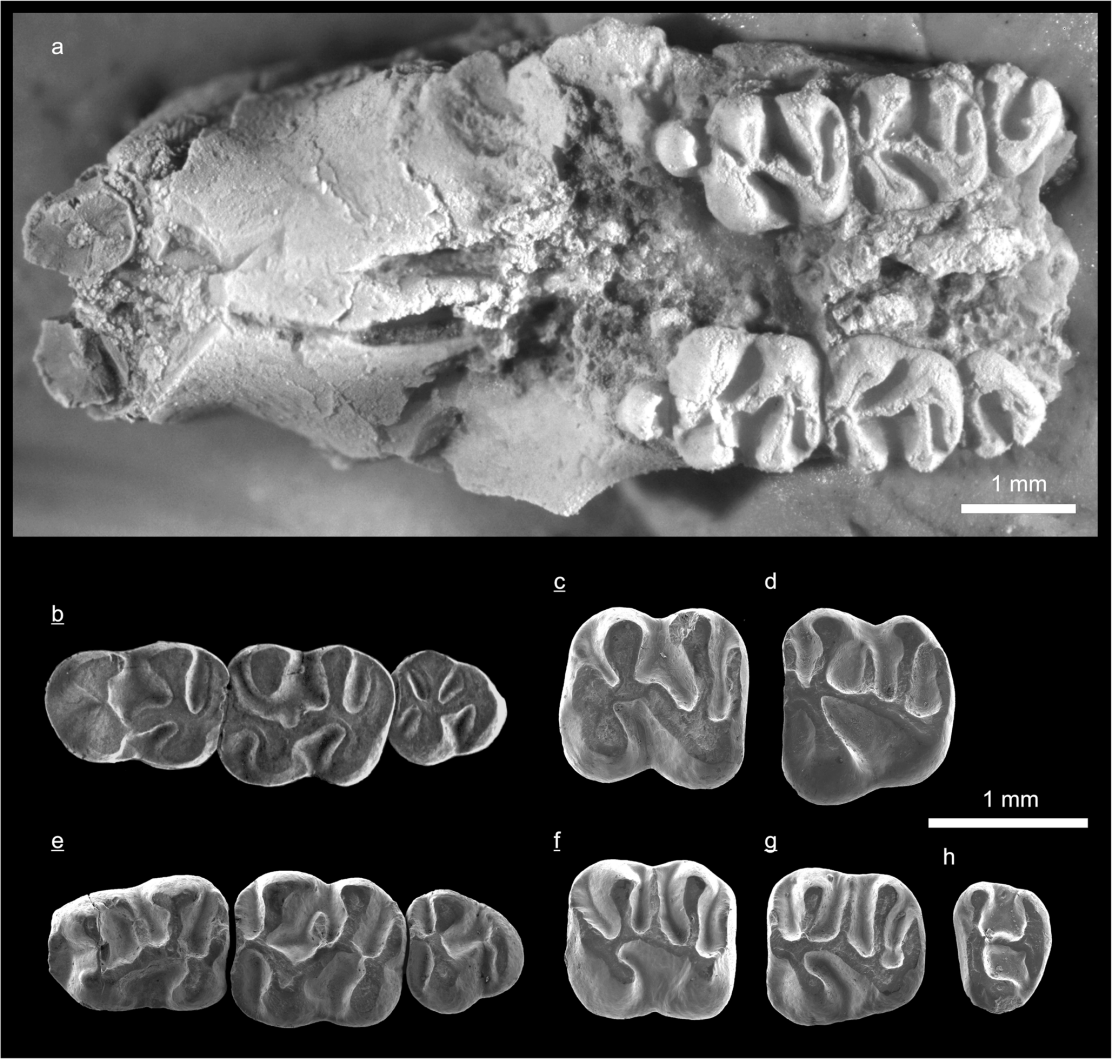
Fam. Dipodidae. ***Litodonomys huangheensis*** **Wang and Qiu**, **2000** from Unkheltseg (UNCH-A/3), Valley of Lakes. Early Miocene, letter zone D. Daxner-Höck et al.( 2014). **a** Skull with right and left P4-M3 (NHMW 2013/0232/0001). **b** Right mand. m1-3 (NHMW 2013/0232/0005). **c** Right M1 (NHMW 2013/0232/0007). **d** Left M2 (NHMW 2013/0232/0008). ***Litodonomys jajeensis*** (**Li and Qiu**, **1980**) from Hotuliin Teeg (HTE-007, HTE-012) and Unkheltseg (UNCH-A/3), Valley of Lakes. Early Miocene, letter Biozone D. Daxner-Höck et al. (2014). **e** Right mand. m1-3 (NHMW 2013/0246/0001), HTE-007. **f** Right M1 (NHMW 2013/0248/0001), HTE-012. **g** Right M2 (NHMW 2013/0242/0015), UNCH-A/3. **h** Left M3 (NHMW 2013/0242/0017), UNCH-A/3

**Fig. 54:**
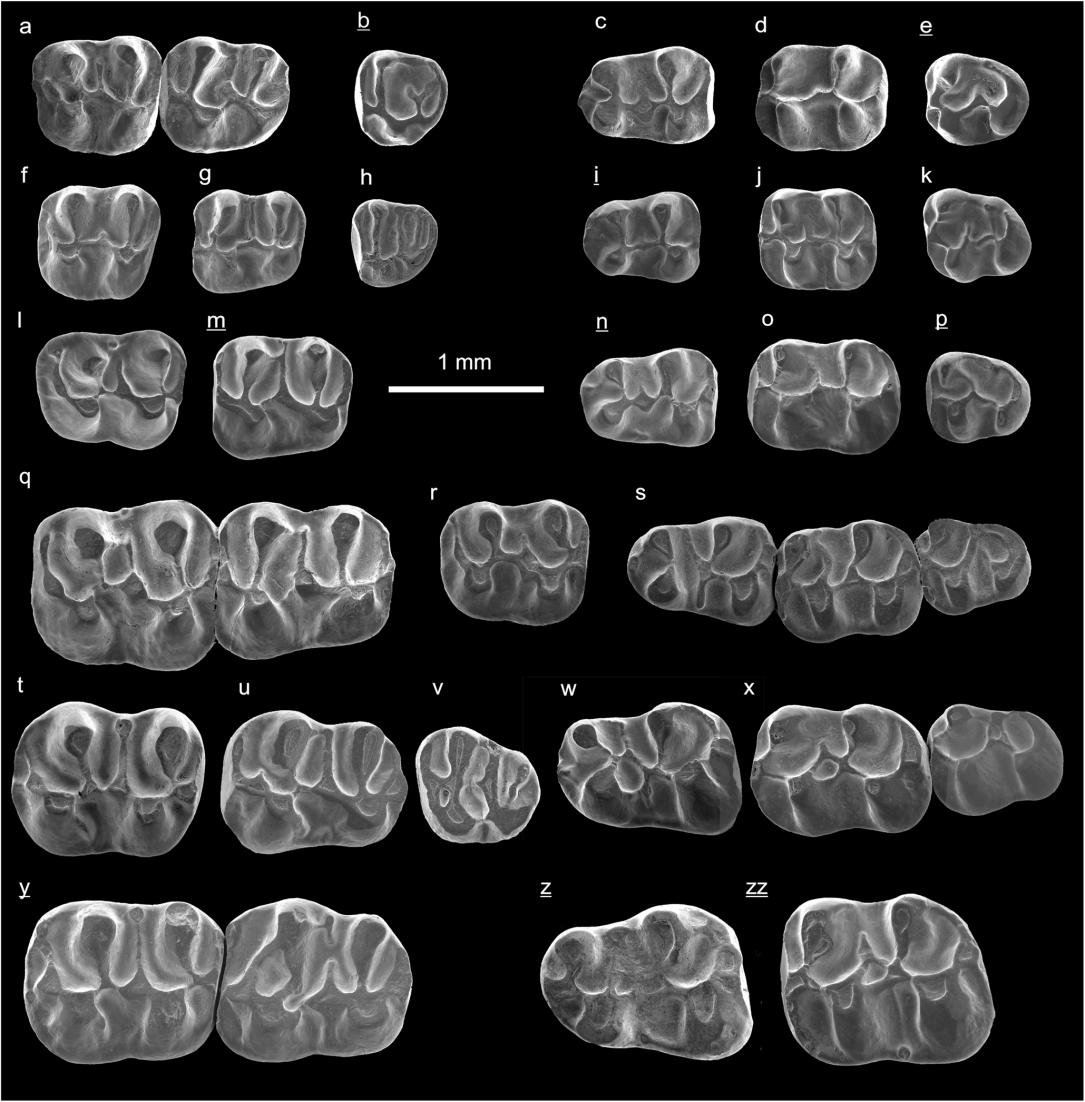
Fam. Dipodidae. ***Allosmintus khandae*** (**Daxner**-**Höck**, **2001**) from Talyn Churum (GRAB-II), Tatal Gol (TAT-D/1), and Taatsiin Gol (TGR-A/13), Valley of Lakes, Mongolia. Early Oligocene, letter zone A. Paratype (P). Daxner-Höck (2001), Daxner-Höck et al. (2014). **a** Left M1-2 (NHMW 2001/0032/0005/1), GRAB-II. **b** Right M3 (NHMW 2001/0032/0001/21), TAT-D/1. **c** Left m1 (NHMW 2001/0032/0002/3), TGR-A/13. **d** Left m2 (NHMW 2001/0032/0002/4), TGR-A/13. **e** Right m3 (NHMW 2001/0032/0001/9), TAT-D/1, **P**. ***Allosminthus minutus*** (**Daxner**-**Höck**, **2001**) from Hsanda Gol (SHG-A/9, SHG-AB/17-20), Tatal Gol (TAT-C/6+7), Taatsiin Gol (TGR-B/1), and Ikh Argalatyn Nuruu (IKH-A/3-4), Valley of Lakes, Mongolia. Early Oligocene, letter zone B. Paratypes (P). Daxner-Höck (2001), Daxner-Höck et al. (2014). **f** Left M1 (NHMW 2001/0033/0005/9), SHG-AB/17-20. **g** Left M2 (NHMW 2001/0033/0003/2), SHG-A/9, **P. h** Left M3 (NHMW 2001/0033/0008/3), TGR-B/1. **i** Right m1 (NHMW 2001/0033/0003/5), SHG-A/9, **P. j** Left m2 (NHMW 2001/0033/0006/3), IKH-A/3-4. **k** Left m3 (NHMW 2001/0033/0011/4), TAT-C/6+7. ***Shamosminthus sodovis*** **Daxner**-**Höck**, **2001** from Taatsiin Gol (TGR-B/1 and TGL-A/11), Valley of Lakes, Mongolia. Early Oligocene, letter zone B. Daxner-Höck (2001), Daxner-Höck et al. (2014). **l** Left M1 (NHMW 2001/0034/0003/3), TGR-B-/1. **m** Right M2 (NHMW 2001/0034/0003/1), TGR-B-/1. **n** Right m1 (NHMW 2001/0034/0003/13), TGR-B-/1. **o** Left m2 (NHMW 2001/0034/0006/2), TGL-A/11. **p** Right m3 (NHMW 2001/0034/0003/16), TGR-B-/1. ***Shamosminthus tongi*** **Huang**, **1992** from Tatal Gol (TAT-055), Valley of Lakes, Mongolia. Late Oligocene, letter zone C. Daxner-Höck et al. (2014). **q** Left M1-2 (NHMW 2013/0251/0001), TAT-055. ***Heterosminthus*** **aff**. ***nanaus*** **Zazhigin and Lopatin**, **2000** from Hotuliin Teeg (HTE-005 and HTE-014-018), Valley of Lakes, Mongolia. Early Miocene, letter zone D. Daxner-Höck et al. (2014). **r** Left M1 (NHMW 2013/0262/0001), HTE-005. **s** Right m1-3 (NHMW 2013/0261/0001), HTE-014-018. ***Heterosminthus firmus*** **Zazhigin and Lopatin**, **2000** from Unkheltseg (UNCH-A/3), Valley of Lakes, Mongolia. Early Miocene, letter zone D. Daxner-Höck (2001), Daxner-Höck et al. (2014). **t** Left M1 (NHMW 2001/0036/0001/2). **u** Left M2 (NHMW 2001/0036/0001/22). **v** Left M3 (NHMW 2001/0036/0001/36). **w** Left m1 (NHMW 2001/0036/0001/53). **x** Left m2-3 (NHMW 2001/0036/0001/84). ***Heterosminthus*** **cf**. ***lanzhouensis*** **Wang and Qiu**, **2000** from Huch Teeg (RHN-021) and Hotuliin Teeg (HTS-056/1+2), Valley of Lakes, Mongolia. Late Oligocene, letter zone C1-D. Daxner-Höck et al. (2014). **y** Right M1-2 (NHMW 2013/0259/0001), RHN-021. **z** Right m1 (NHMW 2013/0260/0002), HTS-056/1+2. **zz** Right m2 (NHMW 2013/0260/0003), HTS-056/1+2

**Fig. 55:**
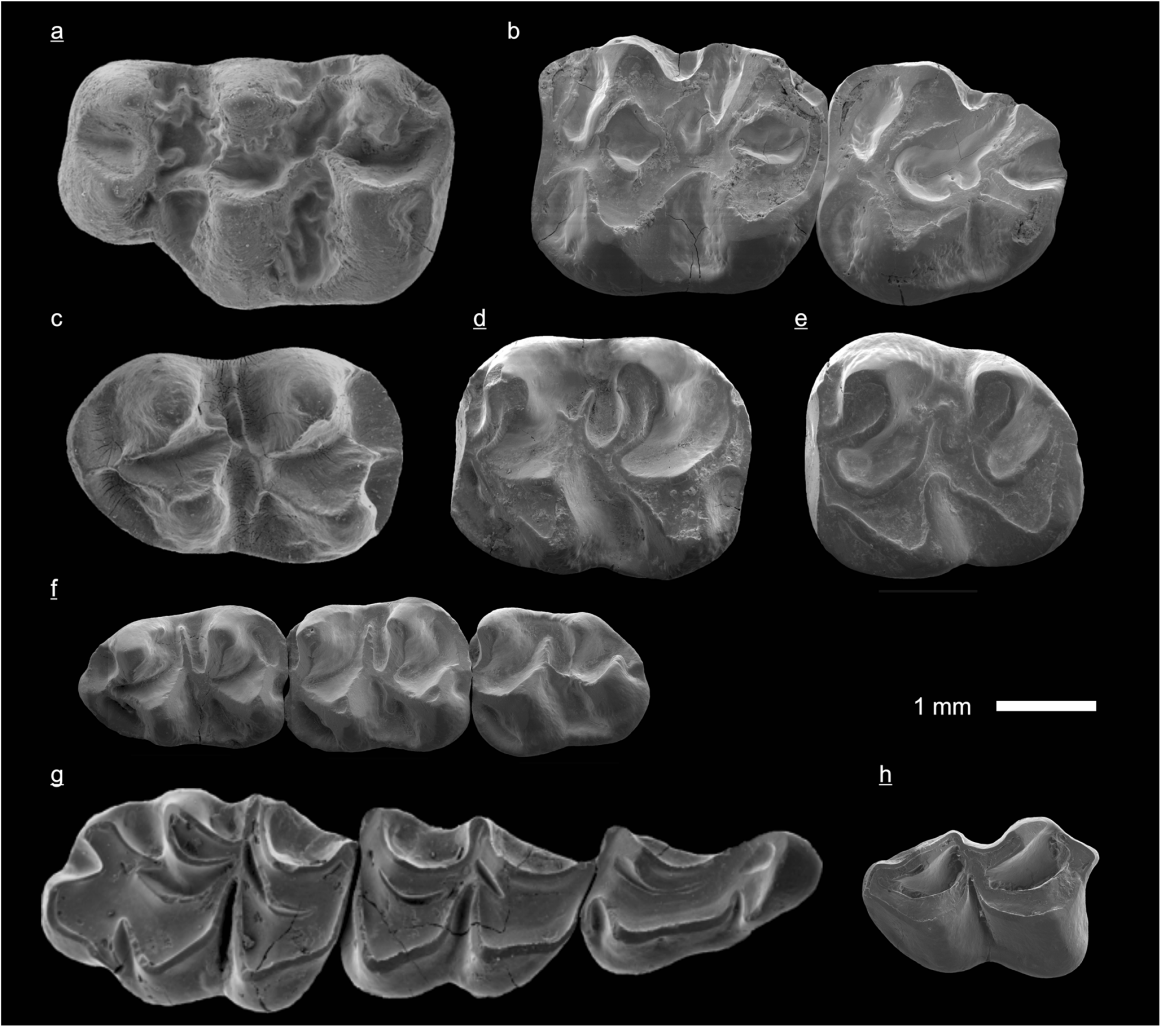
Fam. Cricetidae s.l. ***Cricetops dormitor*** **Matthew and Granger**, **1923** from Taatsiin Gol (TGR-AB/22) and Hsanda Gol (SHG* and SHG-AB/12), Valley of Lakes, Mongolia. Early Oligocene, letter zone B. Daxner-Höck et al. (2010), Maridet et al. (2014b). **a** Right M1(NHMW 2009/0139/0001), TGR-AB/22. **b** Left M2-3 (NHMW 2014/0218/0055), SHG*. **c** Left m1 (NHMW 2009/0139/0002), TGR-AB/22. **d** Right m2 (NHMW 2016/0020/0002), SHG-AB/12. **e** Right m3 (NHMW 2016/0020/0001), SHG-AB/12. ***Cricetops minor*** **Wang**, **1987b** from Tatal Gol (TAT-D/1), Valley of Lakes, Mongolia. Early Oligocene, letter zone A. **f** Right mand m1-3 (NHMW 2014/0225/0002). ***Selenomys mimicus*** **Matthew and Granger**, **1923** from Tatal Gol (TAT-C/2) and Taatsiin Gol (TGL-A/2), Valley of Lakes, Mongolia. Early Oligocene, letter zone B. Daxner-Höck et al. (2010), Maridet et al. (2014b). **g** Right max M1-3 (NHMW 2009/0133/0001), TGL-A/2. **h** Right m2 (NHMW 2016/0021/0002), TAT-C/2

**Fig. 56:**
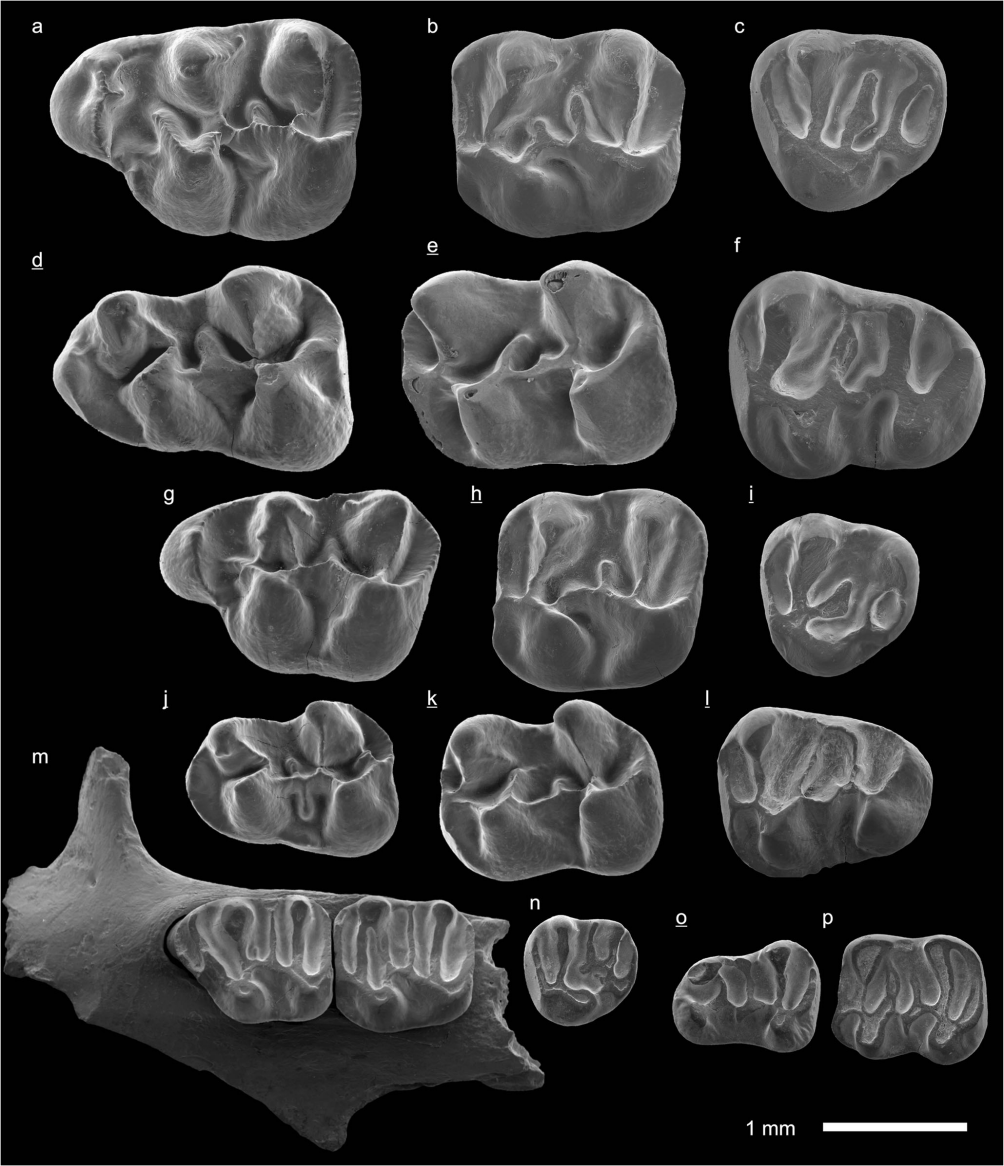
Fam. Cricetidae s.l. ***Eucricetodon asiaticus*** (**Matthew and Granger**, **1923**) from Tatal Gol (TAT-C/3 and TAT-D/1; letter zone A), Hsanda Gol (SHG-A/20; letter zone B), Taatsiin Gol (TGR-B/1; letter zone B), and Ikh Argalatyn Nuruu (IKH-A/2; Biozone B), Valley of Lakes, Mongolia. Early Oligocene. Daxner-Höck et al. (2010), Maridet et al. (2014b), López-Guerrero et al. (2017a, this issue). **a** Left M1 (NHMW 2015/0249/0001), TAT-C/3. **b** Left M2 (NHMW 2015/0252/0001), TAT-D/1. **c** Left M3 (NHMW 2015/0243/0003), SHG-A/20. **d** Right m1 (NHMW 2009/0135/0002), TGR-B/1. **e** Right m2 (NHMW 2009/0135/0004), TGR-B/1. **f** Left m3 (NHMW 2015/0240/0010), IKH-A/2. ***Eucricetodon caducus*** (**Shevyreva**, **1967**) from Tatal Gol (TAT-D/1; letter zone A), Hsanda Gol (SHG-C/1; letter zone A), Taatsiin Gol (TGR-AB/22; letter zone B), and Ikh Argalatyn Nuruu (IKH-A/1; letter zone B), Valley of Lakes, Mongolia. Early Oligocene. Daxner-Höck et al. (2010), Maridet et al. (2014b), López-Guerrero et al. (2017a, this issue). **g** Left M1 (NHMW 2009/0132/0001), SHG-C/1. **h** Left M2 (NHMW 2009/0276/0001), IKH-A/1. **i** Right M3 (NHMW 2009/0287/0013), TAT-D/1. **j** Right m1 (NHMW 2009/0132/0002), SHG-C/1. **k** Right m2 (NHMW 2009/0132/0004), SHG-C/1. **l** Right m3 (NHMW 2009/0294/0003), TGR-AB/22. ***Ulaancricetodon badamae*** **Daxner**-**Höck**, **2000** from Taatsiin Gol (TGL-A/11) and Del (DEL-B/7), Valley of Lakes, Mongolia. Early Oligocene, letter zone B. Daxner-Höck (2000). **m** Left max. M1-2 (NHMW 1999/0083/0033/1), TGL-A/11. **n** Left M3 (NHMW 2016/0018/0001), DEL-B/7. **o** Right m1 (NHMW 2016/0018/0006), DEL-B/7. **p** Left m2 (NHMW 2016/0018/0005), DEL-B/7

**Fig. 57:**
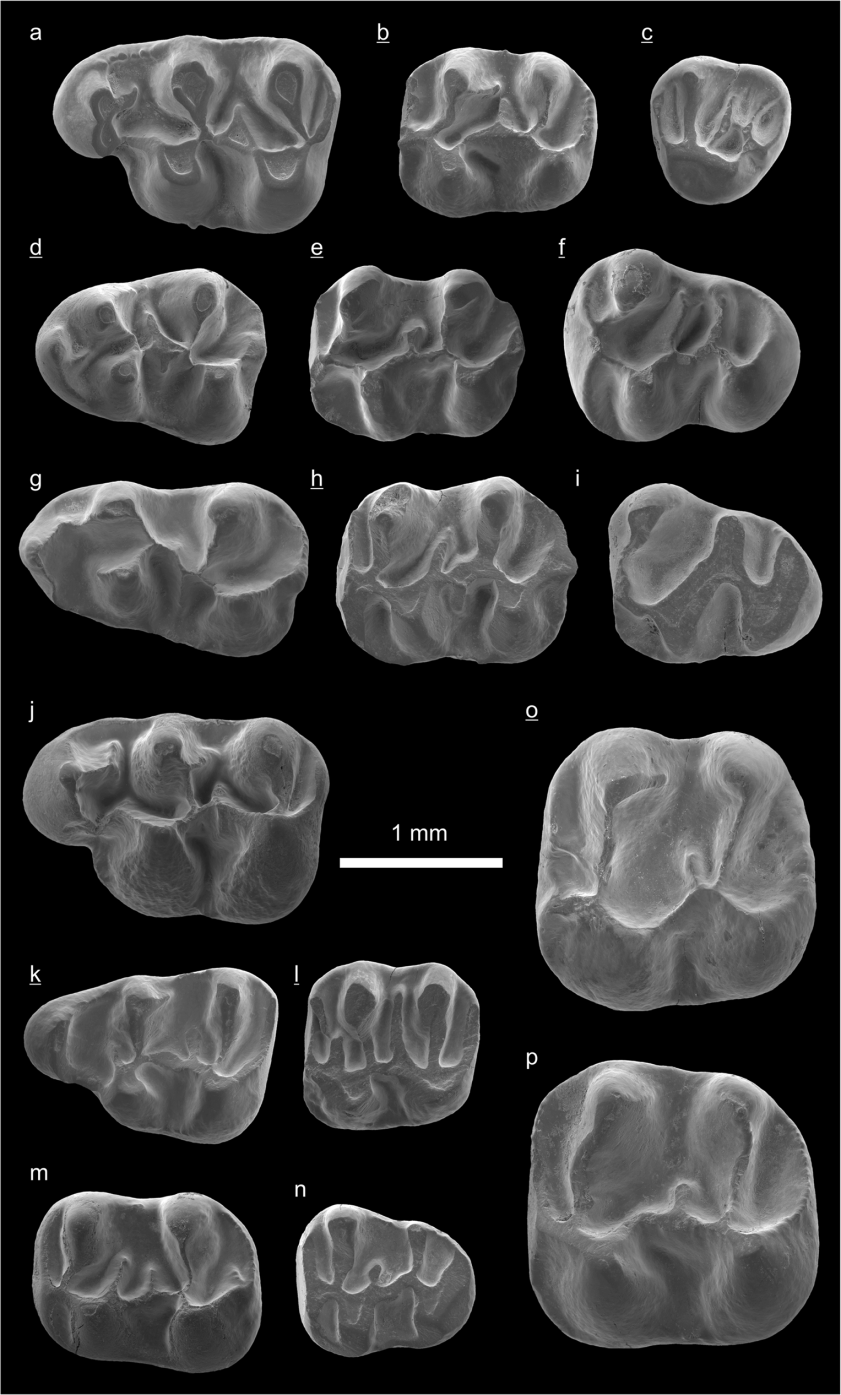
Fam. Cricetidae s.l. ***Eucricetodon bagus*** **Gomes Rodrigues**, **Marivaux and Vianey**-**Liaud**, **2012** from Toglorhoi (TGW-A/2a and TGW-A/2b) and Taatsiin Gol (TGR-C/2), Valley of Lakes, Mongolia. Late Oligocene, letter zone C. Daxner-Höck et al. (2010), Maridet et al. (2014b), López-Guerrero et al. (2017a, 2017b, this issue). **a** Left M1 (NHMW 2015/0272/0005), TGW-A/2a. **b** Right M2 (NHMW 2015/0271/0008), TGR-C/2. **c** Right M3 (NHMW 2015/0273/0021), TGW-A/2b. **d** Right m1 (NHMW 2015/0272/0038), TGW-A/2a. **e** Right m2 (NHMW 2015/0272/0043), TGW-A/2a. **f** Right m3 (NHMW 2015/0273/0035), TGW-A/2b. ***Eucricetodon jilantaiensis*** **Gomes Rodrigues**, **Marivaux and Vianey**-**Liaud**, **2012** from Toglorhoi (TGW-A/2a and TGW-A/2b), Valley of Lakes, Mongolia. Late Oligocene, letter zone C. Maridet et al. (2014b), López-Guerrero et al. (2017a, this issue). **g** Left m1 (NHMW 2015/0340/0011) TGW-A/2a. **h** Right m2 (NHMW 2015/0340/0017) TGW-A/2a. **i** Left m3 (NHMW 2015/0336/0018) TGW-A/2b. **j** Left M1 (NHMW 2015/0336/0001) TGW-A/2b. ***Eucricetodon cf. occasionalis*** **Lopatin**, **1996** from Taatsiin Gol (TGR-AB/22) and Ikh Argalatyn Nuruu (IKH-A/2), Valley of Lakes, Mongolia. Early Oligocene, letter zone B. López-Guerrero et al. (2017a, this issue). **k** Right M1(NHMW 2015/0335/0001), TGR-AB/22. **l** Right M2 (NHMW 2015/0334/0002), IKH-A/2. **m** Left m2 (NHMW 2015/0334/0005), IKH-A/2. **n** Left m3 (NHMW 2015/0335/0002), TGR-AB/22. ***Paracricetodon*** **sp**. from Taatsiin Gol (TGR-A/14), Valley of Lakes, Mongolia. Early Oligocene, letter zone A. Maridet et al. (2014b), López-Guerrero et al. (2017b, this issue). **o** Right M2 (NHMW 2015/0533/0001). ***Witenia*** **sp**. from Unkheltseg (UNCH-A/3B), Valley of Lakes, Mongolia. Early Oligocene, letter zone B. López-Guerrero et al. (2017b, this issue). **p** Left M2 (NHMW 2015/0537/0001)

**Fig. 58:**
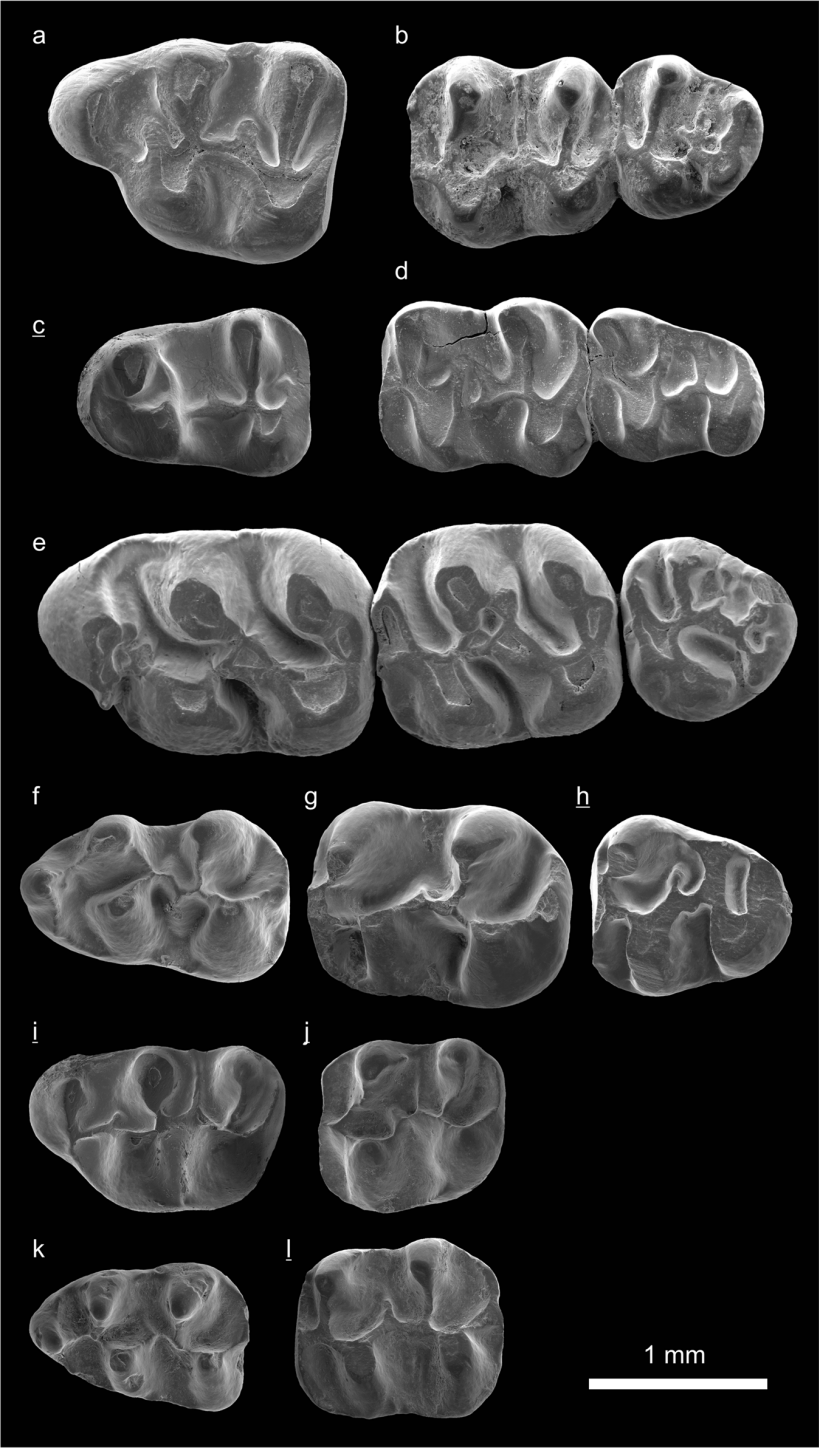
Fam. Cricetidae s.l. ***Eocricetodon meridionalis*** (**Wang and Meng**, **1986**) from Unkheltseg (UNCH-A/3B; letter zone B), Del (DEL-B/7; letter zone B), Taatsiin Gol (TGR-B/1; letter zone B) (**a**–**c**), and ***E. cf. meridionalis***/***E. meridionalis*** (**Wang and Meng**, **1986**) from Toglorhoi (TGW-A/2a; letter zone C) (**c**), Valley of Lakes, Mongolia. Oligocene, letter zones B and C. Maridet et al. (2014b), López-Guerrero et al. (2017b, this issue). **a** Left M1 (NHMW 2015/0311/0001), UNCH-A/3B. **b** Left max. M2-3 (NHMW 2015/0300/0001), DEL-B/7. **c** Right m1 ( NHMW 2015/0307/0001),TGR-B/1. **d** Left mand. m2-3 (NHMW 2015/0310/0002), TGW-A/2a. ***Bagacricetodon tongi*** **Gomes Rodrigues**, **Marivaux and Vianey**-**Liaud**, **2012** from Toglorhoi (TGW-A/2b), Valley of Lakes, Mongolia. Late Oligocene, letter zone C. Maridet et al. (2014b), López-Guerrero et al. (2017b, this issue). **e** Left max. M1-3 (NHMW 2015/0318/0004). **f** Left m1 (NHMW 2015/0318/0009). **g** Left m2 (NHMW 2015/0318/0017). **h** Right m3 (NHMW 2015/0318/0024). ***Democricetodon sui*** **Maridet**, **Wu**, **Je**, **Bi**, **Ni and Meng**, **2011** from Unkheltseg (UNCH-A/3), Valley of Lakes, Mongolia. Early Miocene, letter zone D. Höck et al. (1999), Maridet et al. (2014b). **i** Right M1 (NHMW 2013/0432/0006). **j** Right M2 (NHMW 2013/0432/0003). **k** Left m1 (NHMW 2013/0432/0004). **l** Right m2 (NHMW 2013/0432/0005)

**Fig. 59:**
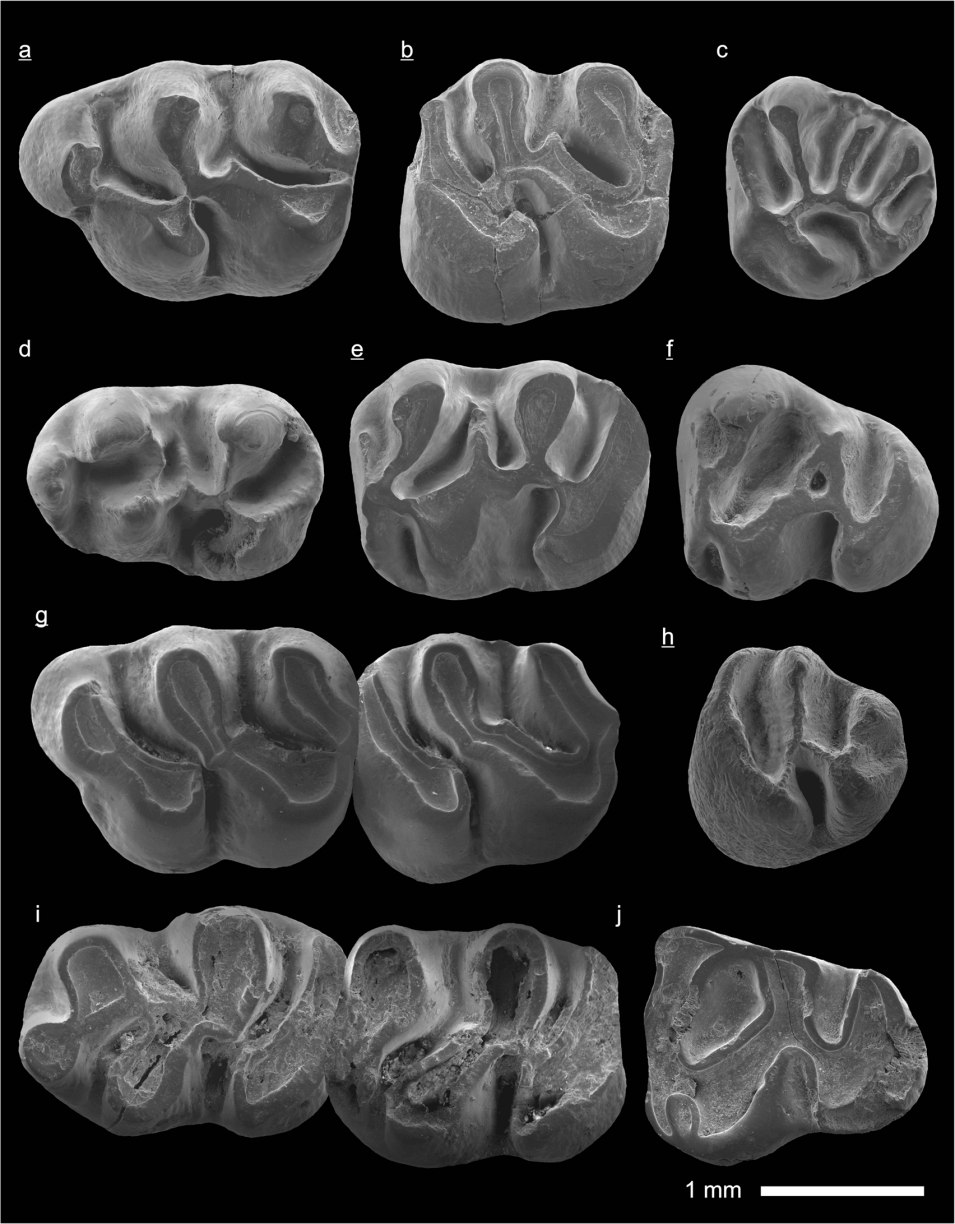
Fam. Cricetidae s.l. ***Aralocricetodon shokensis*** **Bendukidze**, **1993** from Taatsiin Gol (TGR-C/1, TGR-C/2; letter zone C), Toglorhoi (TGW-A/2a; letter zone C), Unzing Churum (TAR-A/2; letter zone C), and Del (DEL-B/12; letter zone C1), Valley of Lakes, Mongolia. Late Oligocene. Daxner-Höck et al. (2010), Maridet et al. (2014b), López-Guerrero et al. (2017b, this issue). **a** Right M1 (NHMW 2009/0142/0005), TGR-C/1. **b** Right M2 (NHMW 2015/0321/0004), TAR-A/2. **c** Left M3 (NHMW 2015/0323/0001), TGW-A/2a. **d** Left m1 (NHMW 2015/0323/0002), TGW-A/2a. **e** Right m2 (NHMW 2015/0322/0009), TGR-C/2. **f** Right m3 (NHMW 2015/0325/0002), DEL-B/12. ***Argyromys cicigei*** **nov. spec**. from Toglorhoi (TGW-A/2a), Valley of Lakes, Mongolia. Late Oligocene, letter zone C. López-Guerrero et al. (2016). **g** Right max. M1-2 (NHMW 2015/0312/0001). **h** Right M3 (NHMW 2015/0312/0006). **i** Left mand m1-2 (NHMW 2015/0312/0007). **j** Left m3 (NHMW 2015/0312/0012)

**Fig. 60:**
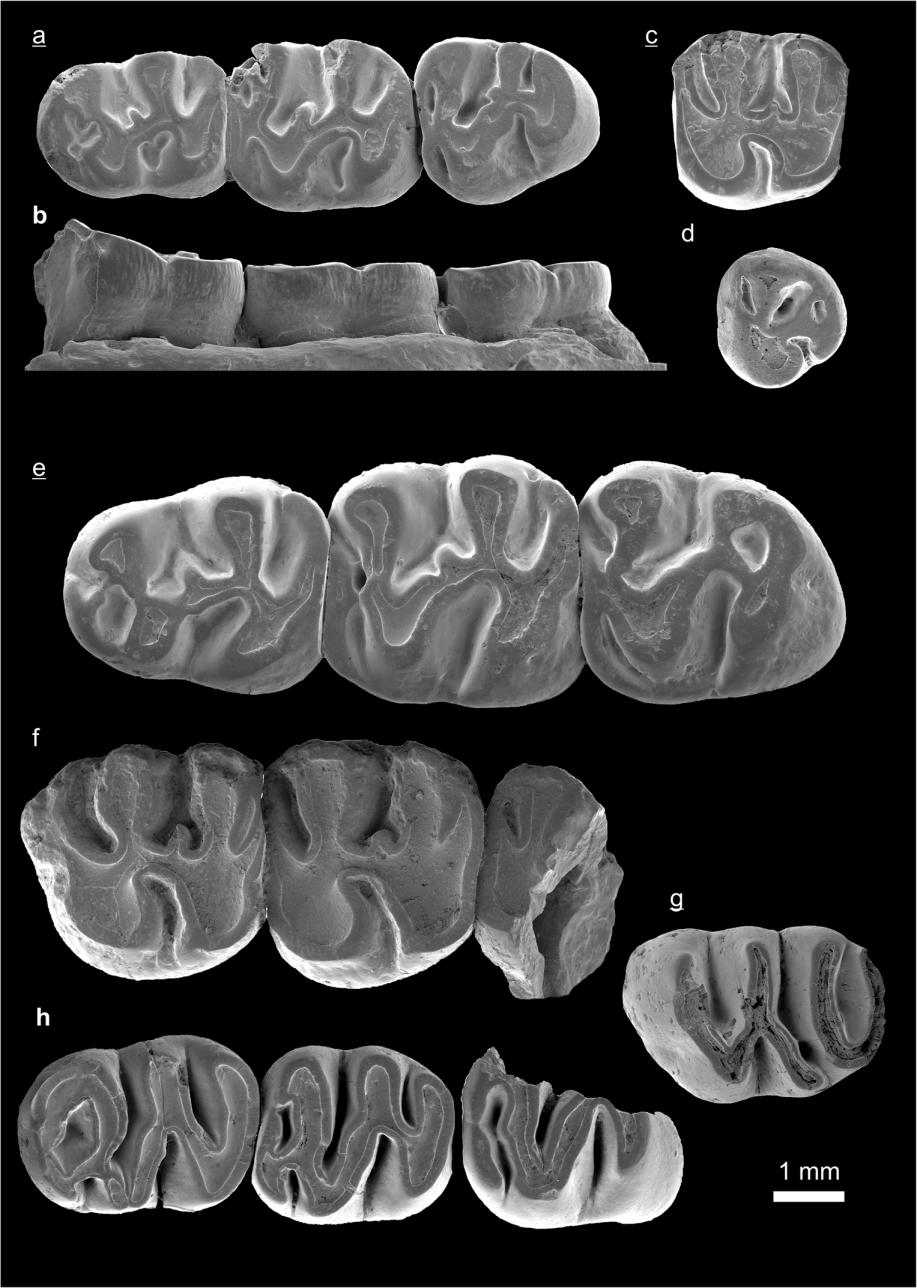
Fam. Tachyoryctoididae. ***Tachyoryctoids bayarmae*** **Daxner**-**Höck**, **Badamgarav and Maridet**, **2015** from Taatsiin Gol (TGR-C/1+2; letter zone C) and Toglorhoi (TGW-A/3+4; letter zone C1), Valley of Lakes, Mongolia. Late Oligocene. Daxner-Höck et al. (2015). **a** Right mand. m1-3 (NHMW 2012/0063/0002), TGW-A/3+4, P. **b** Left mand. m1-3, labial view (NHMW 2012/0062/0001), TGR-C/1. **c** Right M2 (NHMW 2012/0063/0004), TGW-A/3+4, P. **d** Left M3 (NHMW 2012/0063/0006), TGW-A/3+4, P. ***Tachyoryctoides radnai*** **Daxner**-**Höck**, **Badamgarav and Maridet**, **2015** Taatsiin Gol (TGR-C/1-2), Valley of Lakes, Mongolia. Late Oligocene, letter zone C. Daxner-Höck et al. (2015). **e** Right m1-3 (NHMW 2014/0445/0001), **H. f** Left max. M1-3 (NHMW 2014/0445/0008), P. ***Ayakozomys*** **sp**. from Luugar Khudag (LOG-A/1) and Hotuliin Teeg (HTE*), Valley of Lakes, Mongolia. Early Miocene, letter zone D. Daxner-Höck et al. (2015). **g** Right M1 (NHMW 2012/0066/0001), LOG-A/1. **h** Left mand. m1-3 (NHMW 2012/0065/0001), HTE*

**Fig. 61:**
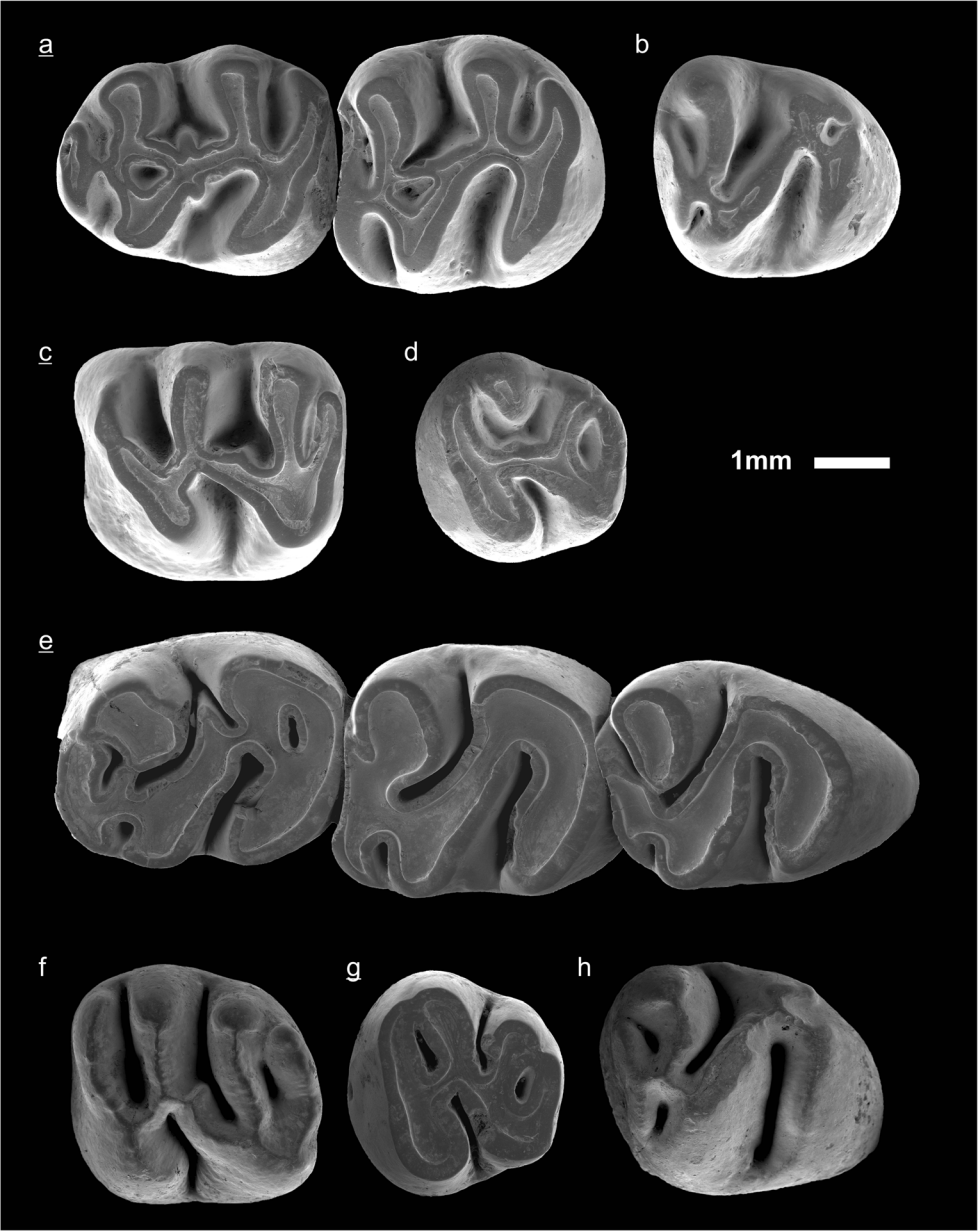
Fam. Tachyoryctoididae. ***Tachyoryctoides obrutschewi*** **Bohlin**, **1937** from Tatal Gol (TAT-051/2), Ikh Argalatyn Nuruu (IKH-B/5), and Hotuliin Teeg (HTE-057), Valley of Lakes, Mongolia. Late Oligocene, letter zone C1. Daxner-Höck et al. (2015). **a** Right m1-2 (NHMW 2013/0450/0001), TAT-051/2. **b** Left m3 from jaw with m2-3 (NHMW 2013/0449/0001), HTE-057. **c** Right M1 (NHMW 2013/0451/0001), IKH-B/5. **d** Left M3 (NHMW 2013/0449/0003), HTE-057. ***Tachyoryctoides tatalgolicus*** **Dashzeveg**, **1971** from Tatal Gol (TAT-043 and TAT-E/22), Valley of Lakes, Mongolia. Late Oligocene, letter zone C1. Daxner-Höck et al. (2015). **e** Right m1-3 (NHMW 2013/0453/0001), TAT-043. **f** Left M2 (NHMW 2013/0453/0004), TAT-043. **g** Right M3 (NHMW 2013/0454/0001), TAT-E/22. **h** Left m3 (NHMW 2013/0453/0003), TAT-043

**Fig. 62:**
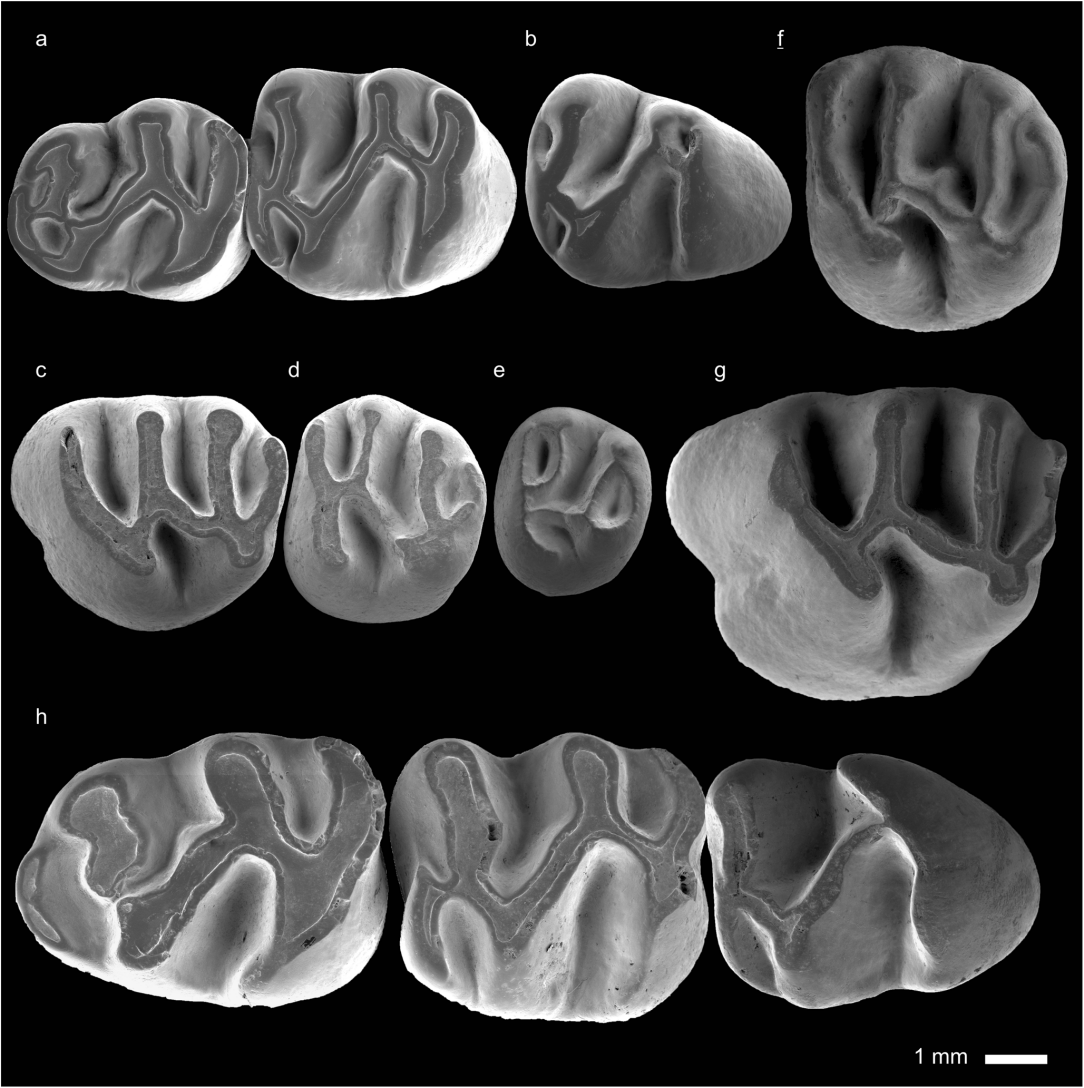
Fam. Tachyoryctoididae. ***Tachyoryctoides kokonorensis*** **Li and Qiu**, **1980** from Hotuliin Teeg (HTE* and HTE-012), Valley of Lakes, Mongolia. Early Miocene, letter zone D. Daxner-Höck et al. (2015). **a** Left m1-2 (NHMW 2013/0456/0001), HTE*. **b** Left m3 (NHMW 2013/0456/0002), HTE*. **c** Left M1 (NHMW 2013/0457/0002), HTE-012. **d** Left M2 (NHMW 2013/0457/0003), HTE-012. **e** Left M3 (NHMW 2013/0457/0004), HTE-012. ***Tachyoryctoides engesseri*** **Wang and Qiu**, **2012** from Hotuliin Teeg (HTE* and HTE-008), Valley of Lakes, Mongolia. Early Miocene, letter zone D. Daxner-Höck et al. (2015). **f** Right M2 (NHMW 2012/0068/0003), HTE*. **g** Left M1 (NHMW 2012/0068/0002), HTE*. **h** Left m1-3 (NHMW 2013/0463/0001), HTE-008

Sediment sequences of the early Chattian are evidenced in the regions Taatsiin Gol (section TGR-C), Toglorhoi (section TGW-A), Abzag Ovo (section ABO-A), Unzing Churum (section TAR-A), and Tatal Gol (section TAT-E).

Sediments of the late Chattian/Tabenbulukian are evidenced in the regions Hotuliin Teeg (section HTE and localities HTSE and HTS), Huch Teeg (section RHN-A), Toglorhoi (section TGW-A), Del (section DEL-B), Tatal Gol (section TAT-E), Hsanda Gol (section SHG-AB), Loh (sections LOH-B, LOH-C), and Ikh Argalatyn Nuruu (sections IKH-A, IKH-B).

### Auitanian (Xiejian/letter zone D)

In the lower part of section HTE (Figs. 10 and 11), strata of the Loh Fm. contain fossils of letter zone C1. The main part of this section is built up by silt and silty claystone and caliche of the Loh Fm. Here, the fossil concentrations are mostly bound to sandy, gravely layers/lenses filling the relief of massive caliche (e.g. fossil layer HTE-007), or to thin layers of caliche nodules (fossil layers HTE-8). The fossils indicate letter zone D and allow correlation with the Xiejian mammal age and the lowermost Miocene.

Sediments of the Aquitanian/Xiejian are evidenced in the regions Hotuliin Teeg (section HTE), Unkheltseg (section UNCH-A), Huch Teeg (section RHN-A), Luuny Yas (locality LUS), and Luugar Khudag (section LOG-A).

## Chronostratigraphic correlation of Mongolian letter zones and calculation of their age ranges

The initial characterisation of Mongolian letter zones was based on preliminarily determined rodents. It included integrated rodent lists, the first/last records, the most abundant/characteristic taxa, the lithostratigraphic position, and the relation to one of the basalt events (Daxner-Höck et al. 1997; Höck et al. 1999). Later, new taxonomic and field data enabled several updates of the original informal biozones (Daxner-Höck and Badamgarav 2007: 14, Tables 3 and 4; Daxner-Höck et al. 2010: 352, Tables 2, 3, 4, 5, and 6; Daxner-Höck et al. 2014: 204–205; Daxner-Höck et al. 2015: 188–190). After finalising the taxonomy of almost all mammal groups, the huge dataset allowed to formalize the informal letter zones as biozones according to the International Stratigraphic Guide. Consequently, the letter zones A, B, C, C1, C1-D, and D, covering the Oligocene and lowermost part of the Miocene, were defined as Taxon Range Zones and Abundance Zones (Harzhauser et al. 2017, this issue). Moreover, the biostratigraphic, lithostratigraphic, radiometric, and magnetostratigraphic data from the study area enable correlations with the GPTS and help estimate the time ranges of the Mongolian biozones. All species and the respective stratigraphic ranges are listed in Table 19.

**Table 19:** The fossil list comprises all identified fossils from the Taatsiin Gol and Taatsiin Tsagaan Nuur region and their stratigraphic ranges (letter zones A–D)

Vertebrata and Gastropoda of the Valley of Lakes/Oligocene early Miocene						
Mongolian letter zones	A	B	C	C1	C1-D	D
**Marsupialia** (Ziegler et al. 2007)						
*Asiadelphis tjutkovae* Emry, Lucas, Szalay & Tleuberdina, 1995	x					
*Asiadelphis zaissanensis* Gabunia, Shevyreva & Gabunia, 1990	x	x				
**Lagomorpha** (Erbajeva 2007, 2013, 2017, this issue; Erbajeva & Daxner-Höck 2014)						
**Leporidae**						
*Ordolagus* cf. *teilhardi* (Burke, 1941)	x	x	x	x		
**Palaeolagidae**						
*Desmatolagus* cf. *vetustus* Burke, 1941	x					
*Desmatolagus youngi* (Gureev, 1960)	x	x				
*Desmatolagus gobiensis* Matthew & Granger, 1923	x	x	x	x		x
*Desmatolagus robustus* Matthew & Granger, 1923		x		x		
*Desmatolagus* cf. *simplex* (Argyropulo, 1940)	x	x	x	x		
*Desmatolagus* cf. *shargaltensis* Bohlin, 1937			x			
*Desmatolagus* cf. *chinensis* Erbajeva & Sen, 1998	x	x	x	x		
*Desmatolagus* cf. *orlovi* (Gureev, 1960)	x	x	x	x		
*Desmatolagus* sp.	x	x	x	x		x
*Bohlinotona pusilla* (Teilhard de Chardin, 1926)			x	x		
*Amphilagus magnus* Erbajeva, 2013				x	x	x
*Amphilagus orientalis* Erbajeva, 2013						x
*Amphilagus plicadentis* Erbajeva, 2013						x
*Amphilagus complicidens* nov. spec. (identified by Erbajeva)						x
**Ochotonidae**						
*Bohlinotona* cf. *pusilla* (Teilhard de Chardin, 1926)			x	x		
*Sinolagomys kansuensis* Bohlin, 1937		x		x	x	x
*Sinolagomys major* Bohlin, 1937				x	x	x
*Sinolagomys ulungurensis* Tong, 1989				x	x	x
*Sinolagomys* sp.	x		x	x	x	x
*Sinolagomys badamae* nov. spec. Erbajeva, Bayarmaa, Daxner-Höck & Flynn (2017, this issue)				x		
*Sinolagomys gracilis* Bohlin, 1942						x
*Bellatona kazakhstanica* Erbajeva, 1988						x
*Bellatona yanghuensis* Zhou, 1988						x
*Alloptox* cf. *minor* Li, 1978						x
**Eulipotyphla** (Ziegler et al. 2007)						
**Erinaceidae**						
*Zaraalestes minutus* (Matthew & Granger, 1924a)	x	x	x	x		
*Zaraalestes* sp.		x				
*Palaeoscaptor acridens* Matthew & Granger, 1924a	x	x	x	x	x	x
*Palaeoscaptor* cf. *rectus* Matthew & Granger, 1924a	x	x	x	x	x	x
*Palaeoscaptor* aff. *rectus* Matthew & Granger, 1924a				x		
*Palaeoscaptor gigas* (Lopatin, 2002)			x	x		
*Palaeoscaptor tenuis* Ziegler, Dahlmann & Storch, 2007	x	x	x	x	x	x
Erinaceidae indet.	x	x	x	x	x	x
*Amphechinus taatsiingolensis* Ziegler, Dahlmann & Storch, 2007			x	x	x	x
*Amphechinus* aff. *taatsiingolensis* Ziegler, Dahlmann & Storch, 2007						x
*Amphechinus minutissimus* Ziegler, Dahlmann & Storch, 2007				x	x	x
*Amphechinus major* Ziegler, Dahlmann & Storch, 2007				x	x	
*Exallerix pustulatus* Ziegler, Dahlmann & Storch, 2007			x			
*Exallerix* sp.						x
**Soricidae**						
*Gobisorex kingae* Sulimski, 1970	x	x	x	x		
Heterosoricinae indet. sp. 1–3		x		x		x
*Taatsiinia hoeckorum* Ziegler, Dahlmann & Storch, 2007		x			x	
*Tavoonyia altaica* Ziegler, Dahlmann & Storch, 2007				x		
Crocidosoricinae indet. sp. 1–11		x	x	x		x
**Talpidae**						
cf. *Asiapternodus mackennai* Lopatin, 2003	x					
*Mongolopala tathue* Ziegler, Dahlmann & Storch, 2007	x			x		
Talpidae indet. sp. 1–9	x	x	x	x		x
**Rodentia**						
**Aplodontidae** (Maridet et al. 2017, this issue)						
*Promeniscomys* cf. *sinensis* Wang, 1987a	x	x				
*Prosciurus* ? *mongoliensis* Wang & Dashzeveg, 2005	x					
*Prosciurus* ? nov. spec.	x					
*Ninamys kazimierzi* Vianey-Liaud, Gomes Rodrigues & Marivaux, 2013	x	x				
*Ninamys arboraptus* (Shevyreva, 1966)	x	x	x			
*Proansomys badamae* sp. nov. Maridet, Daxner-Höck, López-Guerrero, Oliver (2017, this issue)			x	x		
Ansomyinae indet.			x			
*Ansomys* sp. 1						x
**Sciuridae** (Maridet et al. 2014)						
*Plesiosciurus* aff. *sinensis* Qiu & Liu, 1986			x			x
*Kherem shandgoliensis* Minjin, 2004				x		x
Pteromyini indet.						x
? *Eutamias* sp.						x
**Eomyidae** (Maridet et al. 2015)						
*Eomys* cf. *orientalis* Wang & Emry, 1991	x					
*Eomys* aff. *orientalis* Wang & Emry, 1991		x				
*Eomys* sp.		x				
*Asianeomys* cf. *bolligeri* (Lopatin, 2000)			x			
*Asianeomys dangheensis* (Wang, 2002)				x	x	x
**Ctenodactylidae** (Schmidt-Kittler et al. 2007, Oliver et al. 2017, this issue)						
*Karakoromys decessus* Matthew & Granger, 1923	x	x				
*Huangomys frequens* Schmidt-Kittler, Vianey-Liaud & Marivaux, 2007	x	x				
*Yindirtemys shevyrevae* Vianey-Liaud, Schmidt-Kittler & Marivaux, 2006	x	x	x			
*Tataromys sigmodon* Matthew & Granger, 1923		x	x			
*Tatataromys minor longidens* Schmidt-Kittler, Vianey-Liaud & Marivaux, 2007			x	x		
*Tataromys plicidens* Matthew & Granger, 1923			x	x		
*Yindirtemys* aff. *ulantatalensis* (Huang, 1985)			x			
*Yindirtemys deflexus* (Teilhard de Chardin, 1926)				x	x	
*Yindirtemys birgeri* Bendukidze, 1993				x		
*Yindirtemys suni* Li & Qiu, 1980					x	x
*Prodistylomys taatsiini* nov. sp. Oliver, López-Guerrero & Daxner-Höck (in prep)						x
*Prodistylomys mongoliensis* nov. sp. Oliver, López-Guerrero & Daxner-Höck (in prep)						x
*Prodistylomys* sp.						x
**Cylindrodontidae** (Daxner-Höck et al. 2010)						
*Ardynomys* sp.	x	x				
*Anomoemys lohiculus* (Matthew & Granger, 1923)	x	x				
**Tsaganomyidae** (Wessels et al. 2014)						
*Cyclomylus lohensis* Matthew & Granger, 1923	x	x				
*Cyclomylus biforatus* Wang, 2001	x		x			
*Cyclomylus intermedius* Wang, 2001	x	x	x			
Tsaganomyidae indet.	x	x	x			
*Coelodontomys asiaticus* Wang, 2001	x	x	x			
*Tsaganomys altaicus* Matthew & Granger, 1923	x	x	x	x	x	
**Dipodidae** (Daxner-Höck 2001, Daxner-Höck & Wu 2003, Daxner-Höck et al. 2014)						
*Allosminthus khandae* (Daxner-Höck, 2001)	x					
*Allosminthus minutus* (Daxner-Höck, 2001)		x	x			
*Heosminthus chimidae* Daxner-Höck, Badamgarav & Maridet, 2014	x	x	x	x	x	
*Heosminthus* sp.	x	x	x	x		
*Heosminthus borrae* Daxner-Höck, Badamgarav & Maridet, 2014		x	x	x	x	x
*Onjosminthus baindi* Daxner-Höck, Badamgarav & Maridet, 2014	x	x				
*Shamosminthus sodovis* Daxner-Höck, 2001	x	x				
*Shamosminthus* sp.		x				
*Shamosminthus tongi* Huang, 1992			x			
*Bohlinosminthus parvulus* (Bohlin, 1946)	x	x	x	x	x	x
*Parasminthus* cf. *tangingoli* Bohlin, 1946			x	x		
*Parasminthus debruijni* Lopatin, 1999			x	x		
*Parasminthus* cf. *asiaecentralis* Bohlin, 1946			x	x		
*Plesiosminthus* sp.			x	x		x
*Plesiosminthus asiaticus* Daxner-Höck & Wu, 2003				x		
*Plesiosminthus promyarion* Schaub, 1930				x	x	
*Plesiosminthus olzi* Daxner-Höck, Badamgarav & Maridet, 2014						x
*Plesiosminthus barsboldi* Daxner-Höck & Wu, 2003						x
*Litodonomys huangheensis* Wang & Qiu, 2000			x	x	x	x
*Litodonomys lajeensis* (Li & Qiu, 1980)				x	x	x
*Heterosminthus firmus* Zazhigin & Lopatin, 2000				x	x	x
*Heterosminthus* cf. *lanzhouensis* Wang & Qiu, 2000				x	x	
*Heterosminthus* aff. *nanus* Zazhigin & Lopatin, 2000						x
**Muridae** (**Cricetidae s. l**.)and **Tachyoryctoididae** (Daxner-Höck 2000, 2015; Maridet et al. 2014; López-Guerrero et al. (2017a, 2017b, this issue)						
*Tachyoryctoides radnai* Daxner-Höck, Badamgarav & Maridet, 2015			x	x		
*Tachyoryctoides bayarmae* Daxner-Höck, Badamgarav & Maridet, 2015			x	x		
*Tachyoryctoides obrutschewi* Bohlin, 1937				x		
*Tachyoryctoides tatalgolicus* Dashzeveg, 1971				x		
*Tachyoryctoides* sp.				x	x	
*Tachyoryctoides kokonorensis* Li & Qiu, 1980						x
*Tachyoryctoides engesseri* Wang & Qiu, 2012						x
*Ayakozomys* sp.						x
*Ulaancricetodon badamae* Daxner-Höck, 2000	x	x				
*Selenomys mimicus* Matthew & Granger, 1923	x	x				
*Cricetops dormitor* Matthew & Granger, 1923	x	x				
*Cricetops minor* Wang, 1987b	x					
*Paracricetodon* sp.	x					
*Witenia* sp.		x				
*Eocricetodon meridionalis* (Wang & Meng, 1986)	x	x	x	x		
*Eucricetodon caducus* (Shevyreva, 1967)	x	x				
*Eucricetodon asiaticus* Matthew & Granger, 1923	x	x				
*Eucricetodon ﻿cf.﻿occasionalis* Lopatin, 1996		x				
*Eucricetodon bagus* Gomes Rodrigues et al., 2012		x	x	x		
*Eucricetodon jilantaiensis* Gomes Rodrigues at al., 2012		x	x			
*Eucricetodon* sp.			x			
Cricetidae indet.	x	x	x	x		
*Bagacricetodon tongi* Gomes Rodrigues at al., 2012			x	x		
*Aralocricetodon schokensis* Bendukidze, 1993			x	x		
*Argyromys cicigei* nov. spec. López-Guerrero, Zhang and Daxner-Höck (in prep)			x			
*Primus* sp.						x
*Democricetodon sui* Maridet et al., 2011						x
**Creodonta** (Morlo & Nagel 2007, Nagel & Morlo 2003)						
*Hyaenodon* cf. *mongoliensis* (Dashzeveg, 1964)	x					
*Hyaenodon* cf. *incertus* Dashzeveg, 1985	x	x				
*Hyaenodon pervagus* Matthew and Granger 1924b	x	x				
*Hyaenodon eminus* Matthew & Granger, 1925a		x				
cf. *Hyaenodon gigas* Dashzeveg, 1985		x				
Hyaenodontidae indet.		x	x	x		
**Carnivora** (Morlo & Nagel 2007, Nagel & Morlo 2003)						
*Amphicynodon teilhardi* (Matthew & Granger, 1924b)	x	x				
aff. *Amphicynodon* sp.		x				
*Amphicynodon* sp.		x	x			
*Amphicticeps shackelfordi* Matthew & Granger, 1924b		x		x		
*Shandgolictis elegans* Hunt, 1998		x	x			
*Asiavorator altidens* Spassov & Lange-Badré, 1995		x		x		
cf. *Asiavorator* sp.		x	x			
*Nimravus mongoliensis* (Gromova, 1959)		x				
*Palaeogale* sp.		x	x			
Carnivora indet.		x				x
**Leptictida** (Morlo & Nagel 2002)						
cf. *Ergilictis* sp. Lopatin, 1997		x				
*Didymoconus colgatei* Matthew & Granger, 1924b	x	x	x			
*Didymoconus berkey* Matthew & Granger, 1924b		x		x		
Didymoconidae indet.				x		
**Perissodactyla** (Heissig 2007)						
*Paraceratherium* sp.				x		
cf. *Benaratherium* sp.				x		
*Aceratherium* (*Alicornops*) cf. *pauliacense* (Richard, 1937)				x		
Elasmotheriini indet.				x		
cf. *Hoploaceratherium gobiense* (Beliajeva, 1960)						x
cf. *Caementodon* sp.						x
**Ruminantia** (Vislobokova & Daxner-Höck 2002)						
*Lophiomeryx angarae* Matthew & Granger, 1925b	x					
*Lophiomeryx* sp.	x					
*Praetragulus gobiae* (Matthew & Granger, 1925b)	x	x				
*Miomeryx* sp.	x	x				
*Gobimeryx dubius* Trofimov, 1957	x					
*Gobimeryx* sp.	x	x				
*Pseudomeryx gobiensis* Trofimov, 1957	x	x				
*Pseudogelocus mongolicus* Vislobokova & Daxner-Höck, 2002	x	x				
*Pseudomeryx* sp.	x	x				
*Prodremotherium* sp.		x				
*Paragelocus* aff. *scotti* Schlosser, 1902		x	x			
*Eumeryx culminis* Matthew & Granger, 1924a	x					
*Eumeryx* sp.		x	x			
*Dremotherium* cf. *guthi* Jehenne, 1987			x			
*Amphitragulus* sp.				x		
Bovidae gen. 1			x			
Bovidae gen. 2				x		
*Palaeohypsodontus* sp.			x	x		
? *Gobiocerus* sp.				x		
Ruminantia indet.	x	x	x	x		x
**Amphibia and Reptilia** (Böhme 2007)						
**Anura**						
Pelobatidae (aff. *Uldzinia*)	x					
Anura indet.	x					
**Squamata**						
Squamata indet.	x	x				
*Tinosaurus* sp.	x					
Acrodonta indet.		x				
*Lacerta* sp. 1	x	x				
*Lacerta* sp. 2	x	x				
*Lacerta* sp. 3		x				
Lacertidae indet.	x					
Scincomorpha indet.	x					
Melanosaurini indet.		x				
*Calamagras* sp.	x					
**Gastropoda** (Stworzewicz 2007; Neubauer et al. 2013)						
*Pupoides steklovi* Prysjazhnjuk, Devjatkin, Badamgarav & Liskun, 1975	x				x	
?*Strobilops* sp.						x
*Vallonia* cf. *lepida* (Reuss, 1849)	x		x			
*Vallonia stworzewiczae* Neubauer, Harzhauser, Daxner-Höck & Piller, 2013	x		x		x	
*Vallonia* sp.			x	x		x
*Vertigo* cf. *bicolumellata* Steklov and Tsytovich, 1967	x		x			
*Gastrocopta devjatkini* Prysjazhnjuk, Devjatkin, Badamgarav & Liskun, 1975	x		x		x	
*Gastrocopta* cf. *mongolica* Prysjazhnjuk, Devjatkin, Badamgarav & Liskun, 1975	x		x			
*Gastrocopta shandgolica* Prysjazhnjuk, Devjatkin, Badamgarav & Liskun, 1975	x		x			
*Gastrocopta tuvaense* Steklov, 1967			x		x	
*Gastrocopta valentini* Stworzewicz, 2007	x				x	

### Letter zone A

The lower Hsanda Gol Fm. correlates with Chron C12 r and the upper part of C13 (Kraatz and Geisler 2010; Sun and Windley 2015) and ranges from the Eocene/Oligocene boundary (EOB) at ∼34 Ma to basalt I at ∼31.5 Ma. Key fossils of letter zone A (Harzhauser et al. 2017, this issue) were recovered from the upper part of the lower Hsanda Gol beds, which correlate with Chron C12r.

Samples: TGR-A/13+14; TGL-A/1+2; HL-A/1+2; TAT-C/1-3; TAT-D/1; TAT-037; SHG-C/1+2; GRAB-II. The range is ∼33 to ∼31.5 Ma (early Oligocene/early Rupelian/early Hsandagolian) (Fig. 31).

### Letter zone B

Fossils of letter zone B are present in upper Hsanda Gol beds above basalt I (∼31.5 Ma). The upper boundary is built by sequences of the Hsanda Gol and Loh Fms., which include fossils of letter zone C.

Samples: TGR-B/1; TGR-AB/21, TGR-AB/22; TGL-A/11; UNCH-A/3+4B; DEL-B/7+8; TAT-054; TAT-E/3; TAT-038; TAT-C/6+7; SHG-A/6, SHG-A/9, SHG-A/12-15; SHG-A/15-20; SHG-AB/12; SHG-AB/17-20; IKH-A/1-4; IKH-B/2. The range is ∼31.5 to ∼28 Ma (early Oligocene/late Rupelian/late Hsandagolian).

### Letter zone C

Fossils of letter zone C are present in sediments of the upper Hsanda Gol Fm. and/or lower Loh Fm., which correlate with Chron C9n–C8n.2n (section TGR-C; Sun and Windley 2015) and with radiometric ages of basalt II (27–28 Ma) from sections ABO-A and TAR-A.

Samples: TGR-C/1+2; TGR-C/5-7; ABO-A/3; ABO-083; TAR-A/2; TGW-A/1; TGW-A/2a+b; TAT-055.

The range is ∼28 to 25.6 Ma (late Oligocene/early Chattian/latest Hsandagolian).

### Letter Zone C1

Hsanda Gol or Loh sediments with fossils of letter zone C1 are correlated with Chrons C8n.2n–C7n.2n (section TGR-C, above sediment layer TGR-C/11; Sun and Windley 2015).

Samples: TGW-A/3+4; TGW-A/5; HTE-057; HTSE-009; HTSE-013; DEL-B/12; RHN-A/6; RHN-A/7; RHN-A/8-9; RHN-A/10; RHN-023; RHN-019; TAT-043; TAT-044; TAT-E/22; TAT-027; TAT-051/1-2; TAT-052/1; SHG-AB top.; LOH-C/1; LOH-B/3; IKH-A/5; IKH-B/5.

The range is 25.6 to 24 Ma (late Oligocene/late Chattian/Tabenbulukian).

### Letter zone C1-D

Sediments of the upper Hsanda Gol Fm. or lower Loh Fm. comprising fossils of letter zone C1-D mark the uppermost Oligocene above letter zone C1 and below letter zone D.

Samples: HTS-056/1-3; RHN-021+022; RHN-A/11; TAT-E/32; TAT-052/2.

The estimated range of letter zone C1-D is 24 to ∼23 Ma (late Oligocene/late Chattian/Tabenbulukian).

### Letter zone D

The lower Loh Fm. with fossils of letter zone D is demonstrated as being early Miocene by the occurrence of *Democricetodon sui* Maridet et al. 2011, which has its first appearance (FAD) at 22.6 Ma (top of Chron C6Cn.1n) in the type locality S-II site XJ99005 of the Tieersihabahe section, Junggar Basin, China (Meng et al. 2006, 2008, 2013).

Samples: LUS-027-029; LOG-A/1; HTE-008; HTE-009; HTE-014-018; HTE-005; HTE-007; HTE-12/6; HTE-012/8; HTE*; HTE-012; HTE-12/7; UNCH-A/3+4; RHN-A/12; RHN-020.

The estimated range is ∼23 to ∼21 Ma (early Miocene/Aquitanian/Xiejian).

## Fossil record and dental morphology of Marsupialia, Eulipotyphla, and Rodentia from the Oligocene and early Miocene of the studied area

Here, we introduce into the fossil record of the Oligocene and lowermost Miocene (Table 19); younger assemblages are not considered in this issue. The fossils were collected from 70 fossil horizons of 20 geological sections and 6 fossil points in the Valley of Lakes. The recovered fossils encompass Gastropoda (Stworzewicz 2007; Neubauer et al. 2013), Anura and Squamata (Böhme 2007), Creodonta, Carnivora and Leptictida (Morlo and Nagel 2002, 2007; Nagel and Morlo 2003), Perissodactyla (Heissig 2007), and Ruminantia (Vislobokova and Daxner-Höck 2002). The prevailing part of fossils—about 98% of more than 19,000 fossils—represents small mammals, of which 135 species-level taxa were counted. This small mammal dominance, however, results from wet screening of large samples.

Among small mammals, the order Rodentia dominates in genus, species, and specimen numbers, followed by Lagomorpa and Eulipotyphla and Marsupialia. Rodentia encompass the families Aplodontidae, Sciuridae, Eomyidae, Ctenodactylidae, Cylindrodontidae, Tsaganomyidae, Dipodidae, Cricetidae s. l., and Tachyoryctoididae. Together, they comprise 85 species-level taxa. Lagomorpha are represented by the families Leporidae, Palaeolagidae, and Ochotonidae, altogether with 23 species-level taxa. Eulipotyphla are represented by the families Erinaceidae, Soricidae, and Talpidae, together 25 species-level taxa. Additionally, two Marsupialia species of the family Didelphidae occur.

In this chapter, the richest small mammal collection ever found in Mongolia is illustrated by SEM images (Figs. 32, 33, 34, 35, 36, 37, 38, 39, 40, 41, 42, 43, 44, 45, 46, 47, 48, 49, 50, 51, 52, 53, 54, 55, 56, 57, 58, 59, 60, 61, and 62). We give an overview of the diversity; show the manifold dental structures of marsupials, eulipotyphlans, and rodents; and provide a first impression of species, which are named in fossil lists or serve as index fossils for biostratigraphy. Fossils which indicate Taxon Range Zones and Abundance Subzones (Harzhauser et al. 2017, this issue) are written in bold letters (see list of figured species, below). Fossil descriptions are not included in this chapter; for more detailed information, we refer on the original descriptions and included references. Other fossil groups, such as gastropods, lower vertebrates, large mammals, lagomorphs, and the large-sized rodents Tsaganomyidae, are not figured in this paper. The figured teeth (SEM images) of marsupials, eulipotyphlans, and rodents are roughly arranged in systematic order. The figure captions include the taxon name, collection and inventory number, the locality, section, fossil layer, the age of the sample, respective letter zone, and the author who identified or described the fossils. For better comparison, all right-side fossils are mirrored (they are figured as if they were from the left side), and these numbers are underlined (e.g. Fig. 32b = right M1 of *Asiadelphis zaissanensis*). A scale bar shows the magnification of fossils.

The figured species are

Order Marsupialia

Family Didelphidae:

*Asiadelphis zaissanensis* (Fig. 32a–f)

*Asiadelphis tjutkovae* (Fig. 32g)

Order Eulipotyphla

Family Erinaceidae:

*Exallerix pustulatus* (Fig. 32h–j)

*Zaraalestes minutus* (Fig. 33a–l)

*Zaraalestes* sp. (Fig. 33m–p)

***Amphechinus taatsiingolensis*** (Fig. 34a–n)

*Amphechinus minutissimus* (Fig. 35a–i)

***Amphechinus major*** (Fig. 35j–q)

*Palaeoscaptor gigas* (Fig. 36a–b)

*Palaeoscaptor tenuis* (Fig. 36c–n

*Palaeoscaptor acridens* (Fig. 37a–g)

*Palaeoscaptor* cf. *rectus* (Fig. 37h–n)

Family Soricidae:

*Gobisorex kingae* (Fig. 38a–i)

*Taatsiinia hoeckorum* (Fig. 39a–f)

*Tavoonyia altaica* (Fig. 39/g–n)

Family Talpidae:

*Mongolopala tathue* (Fig. 40a–f)

Order Rodentia

Family Aplodontidae:

*Ninamys kazimierzi* (Fig. 41a–e)

*Ninamys arboraptus* (Fig. 41f–h)

*Prosciurus* ? *mongoliensis* (Fig. i–k)

*Promeniscomys* cf. *sinensis* (Fig. 41l–r)

*Proansomys badamae sp. nov.* (Fig. 42a–h)

Family Sciuridae:

*Kherem shandgoliensis* (Fig. 42i–j)

Pteromyini indet. (Fig. 42k–m)

*Plesiosciurus* aff. *sinensis* (Fig. 42n–p)

Family Cylindrodontidae:

*Anomoemys lohiculus* (Fig. 43a–f)

*Ardynomys* sp. (Fig. 43g–l)

Family Ctenodactylidae:

*Tataromys minor longidens* (Fig. 44a–j)

*Tataromys sigmodon* (Fig. 44k–s)

*Tataromys plicidens* (Fig. 44t–zz)

*Karakoromys decessus* (Fig. 45a–i)

***Huangomys frequens*** (Fig. 45j–p)

*Yindirtemys birgeri* (Fig. 46a–d)

***Yindirtemys deflexus*** (Fig. 46e–k)

*Yindirtemys suni* (Fig. 46/l–q)

*Yindirtemys shevyrevae* (Fig. 47a–h)

*Yindirtemys* aff. *ulantatalensis* (Fig. 47i–j)

*Prodistylomys* nov. spec. 1 (in prep.) (Fig. 47k)

*Prodistylomys* nov. spec. 2 (in prep.) (Fig. 47l–o)

Family Eomyidae:

*Eomys* aff. *orientalis* (Fig. 48a–c)

*Eomys* cf. *orientalis* (Fig. 48d)

cf. *Asianeomys bolligeri* (Fig. 48e–j)

*Asianeomys dangheensis* (Fig. 48k–s)

Family Dipodidae:

*Heosminthus chimidae* (Fig. 49a–g)

*Heosminthus borrae* (Fig. 49h–q)

*Plesiosminthus asiaticus* (Fig. 50a–c)

*Plesiosminthus promyarion* (Fig. 50d–h)

*Plesiosminthus barsboldi* (Fig. 50i–k)

*Plesiosminthus olzi* (Fig. 50l–s)

*Onjosminthus baindi* (Fig. 51a–g)

*Bohlinosminthus parvulus* (Fig. 51h–p)

*Parasminthus debruijni* (Fig. 52a–d)

*Parasminthus* cf. *tangingoli* (Fig. 52e–j)

*Parasminthus* cf. *asiaecentralis* (Fig. 52k–l)

*Litodonomys huangheensis* (Fig. 53a–d)

*Litodonomys jajeensis* (Fig. 53e–h)

***Allosmintus khandae*** (Fig. 54a–e)

*Allosminthus minutus* (Fig. 54f–k)

*Shamosminthus sodovis* (Fig. 54l–p)

*Shamosminthus tongi* (Fig. 54q)

*Heterosminthus* aff. *nanaus* (Fig. 54r–s)

*Heterosminthus firmus*)

*Heterosminthus* cf. *lanzhouensis* (Fig. 54y–zz)

Family Cricetidae s.l.:

***Cricetops dormitor*** (Fig. 55a–e)

*Cricetops minor* (Fig. 55f)

*Selenomys mimicus* (Fig. 55g–h)

*Eucricetodon asiaticus* (Fig. 56a–f)

*Eucricetodon caducus* (Fig. 56g–l)

*Ulaancricetodon badamae* (Fig. 56m–p)

*Eucricetodon bagus* (Fig. 57a–f)

*Eucricetodon jilantaiensis* (Fig. 57g–j)

*Eucricetodon cf. occasionalis* (Fig. 57k–n)

*Paracricetodon* sp./*Witenia* sp. (Fig. 57o–p)

*Eocricetodon meridionalis* (Fig. 58a–d)

*Bagacricetodon tongi* (Fig. 58e–h)

*Democricetodon sui* (Fig. 58i–l)

*Aralocricetodon shokensis* (Fig. 59a–f)

*Argyromys* nov. spec. (Fig. 59g–j)

Family Tachyoryctoididae

*Tachyoryctoids bayarmae* (Fig. 60a–d)

*Tachyoryctoides radnai* (Fig. 60e–f)

*Ayakozomys* sp.(Fig. 60g–h)

*Tachyoryctoides obrutschewi* (Fig. 61a–d)

*Tachyoryctoides tatalgolicus* (Fig. 61e–h)

***Tachyoryctoides kokonorensis*** (Fig. 62a–e)

*Tachyoryctoides engesseri* (Fig. 62f–h)

## Conclusions

The Taatsiin Gol and Taatsiin Tsagaan Nuur region, part of the Valley of Lakes, yields Oligocene and Miocene sediment deposits. They are very important in several respects. First, the sequences of the Hsanda Gol and Loh Fms. contain a rich mammalian fauna and provide unique evidence of mammal evolution and climatic changes (Harzhauser et al. 2016). Second, the Cenozoic strata are intercalated with basalt flows, and the ^40^Ar/^39^Ar data of these basalts constrain the time of sediment deposition. Thus, basalt ages and Mongolian letter zones enable a composite age chronology for the studied area (Höck et al. 1999; Daxner-Höck et al. 2010).

From Luuny Yas in the northwest to Ihk Argalatyn Nuur in the east (∼101–102° longitude), 20 sections and 6 fossil localities were investigated in detail (Table 3, Fig. 3). The description of sections are original, comprising lithology, sediment structures and thicknesses of sediment layers, illustrations of the localities/sections, the GPS positions, faunal lists of the fossil horizons, biozonation, radiometric ages of imbedded basalts, and magnetostratigraphic data (Figs. 4, 5, 6, 7, 8, 9, 10, 11, 12, 13, 14, 15, 16, 17, 18, 19, 20, 21, 22, 23, 24, 25, 26, 27, 28, and 29).

The composite sequence includes four formations from bottom to top: The lowermost fluvio-lacustrine sequence is named Tsagan Ovo Fm. It is overlain by red clay and silt of the Hsanda Gol Fm., which itself is divided by basalt I (31.5 Ma) into the lower and upper Hsanda Gol beds. Upsection, fluvial deposits of the Loh Fm. follow, which are locally covered by pebbles of the Tuyn Gol Fm. Basalt II flows, dated at ∼27 Ma, contact sediments of the Hsanda Gol and Loh Fms, as evidenced in sections ABO-A and TAR-A, respectively. Most basalt II occurrences with ages between ∼25 and ∼28 Ma do not have contact with fossil beds (Tables 1and 2). The upper parts of several sections, which are built up by the Loh and Tuyn Gol Fms. and comprise fossils younger than lowermost Miocene, are not considered in this study.

Magnetostratigraphic measurements of the TGR sections show that the Tsagan Ovo Fm. corresponds with Chrons C15r–C13r, an age range of >35–34 Ma, which is late Eocene. The lower Hsanda Gol strata and basalt I correspond with the palaeomagnetic polarity chrons C13r–C12r, an age range of ∼34–31.2 Ma (Kraatz and Geisler 2010; Sun and Windley 2015), which is early Oligocene. Thus, the boundary between the Tsagan Ovo and Hsanda Gol Fms. corresponds with the Eocene-Oligocene boundary (EOB). The boundary between the Hsanda Gol and Loh Fms. is heterochronous. Locally, Hsanda Gol sediments range to the latest Oligocene (e.g. section TAT-E; Fig. 21); in other regions, sedimentation of the Loh Fm. started in the early late Oligocene (e.g. section TAR-A; Fig. 18).

We sampled more than 19,000 mammal fossils from 70 individual fossil layers, yielding a total of 176 mammal species, mostly small mammals. The representation of large mammals, lower vertebrates, and gastropods is comparably poor.

This unique dataset enables evaluation and formalization of the Mongolian letter zones A, B, C, C1, C1-D, and D (Harzhauser et al. 2017, this issue). The biostratigraphic data from Oligocene and early Miocene sequences, the ^40^Ar/^39^ Ar ages of basalts I and II (Tables 1 and 2 and Höck et al. 1999), and magnetostratigraphic measurements (Kraatz and Geisler 2010; Sun and Windley 2015) help correlate sections and fossil sites with the Geomagnetic Polarity Time Scale GPTS (Gradstein et al. 2012) and assess the precise ages of mammal faunas and time ranges of Mongolian letter zones (Figs. 30 and 31).

Importantly, the δ^13^C and δ^18^O isotope values of authigenic carbonate in calcrete horizons and analyses of mammal community structures reflect changes of the palaeoclimate during the Oligocene and early Miocene (Richoz et al. 2017, this issue; Harzhauser et al. 2016, accepted).

The manifold dental morphology is illustrated by SEM images of teeth from marsupials, insectivores, and rodents (Figs. 32, 33, 34, 35, 36, 37, 38, 39, 40, 41, 42, 43, 44, 45, 46, 47, 48, 49, 50, 51, 52, 53, 54, 55, 56, 57, 58, 59, 60, 61, and 62), and Table 19 lists all investigated fossil taxa and the respective stratigraphic ranges.

## Electronic supplementary material

Below is the link to the electronic supplementary material.

ESM 1 (DOCX 25.1 kb)
