# Supplementary material for: Oligocene stratigraphy across the Eocene and Miocene boundaries in the Valley of Lakes (Mongolia)
Source: Paleobiodivers Paleoenviron. 2017 Mar 7;97(1):111–218. doi: 10.1007/s12549-016-0257-9 (PMC5367740; doi:10.1007/s12549-016-0257-9)
Supplement: Supplementary file 1 — (DOCX 25.1 kb) [file 12549_2016_257_MOESM1_ESM.docx]

|  | locality | code section/  locality | code sample | Coordinates | letter  zone |
| --- | --- | --- | --- | --- | --- |
| Fig. 4 | Luuny Yas | LUS | LUS-027 | N 45° 32’05.5”  E 100°56’49.9” | D |
| Fig. 4 | Luuny Yas | LUS | LUS-028 | N 45° 32’06.4”  E 100°56’54.5“ | D |
| Fig. 4 | Luuny Yas | LUS | LUS-029 | N 45° 32’02.8  E 100°56’51.5” | D |
| Fig. 5 | Luugar Khudag | LOG-A | LOG-A/1 | N 45°32’09.6”  E 101°00’51.5” | D |
| Fig. 6 | Abzag Ovo | ABO-A | ABO-A/3 | N 45°34’25.4”  E 101°03’49.7” | C |
| Fig. 7 | Toglorhoi | TGW-A | TGW-A/1-2 |  | C |
| Fig. 7 | Toglorhoi | TGW-A | TGW-A/3-4 |  | C1 |
| Fig. 7 | Toglorhoi | TGW-A | TGW-A/5 | N 45°22’37.6”  E 101°05’49.2” | C1 |
| Fig. 8 | Khongil | HL-A | HL-A/1-2 | N 45°27’40.4”  E 101°09’18.5” | A |
| Fig. 9 | Huch Teeg | RHN-A | RHN-A/12 | N 45°29’29.9”  E 101°12’17.1” | D |
| Fig. 9 | Huch Teeg | RHN-A | RHN-020 | N 45°29’29.8”  E 101°12’18.3” | D |
| Fig. 9 | Huch Teeg | RHN-A | RHN-021; RHN-A/11 | N 45°29’30.6”  E 101°12’19.1” | C1-D |
| Fig. 9 | Huch Teeg | RHN-A | RHN-019; RHN-A/10 | N 45°29’30.5”  E 101°12’20.2” | C1 |
| Fig. 9 | Huch Teeg | RHN-A | RHN-A/7; RHN-A/8-9 | N 45°29’36.0”  E 101°12’22.2” | C1 |
| Fig. 9 | Huch Teeg | RHN-A | RHN-023 | N 45°29’33.6”  E 101°12’30.0” | C1 |
| Fig. 10  Fig. 11d | Hotuliin Teeg | HTE | HTE-057 | N 45°28’54.2”  E 101°12’26.2” | C1 |
| Fig. 11c | Hotuliin Teeg | HTE | HTE-008;HTE-003; HTE-009; HTE-014-018 | N 45°29’07.4”  E 101°11’58.9” | D |
|  | Hotuliin Teeg | HTE | HTE-007 | N 45°29’08.2”  E 101°11’49.3” | D |
| Fig. 11b | Hotuliin Teeg | HTE | HTE-005; HTE-12/6;  HTE-12/8 | N 45°29’09.7“  E 101°11’49.0” | D  D |
| Fig. 11b | Hotuliin Teeg | HTE | HTE-012; HTE-12/7 | N 45°29’11.9”  E 101°11’49.3” | D |
| Fig. 11f | Hotuliin Teeg | HTSE | HTSE-009 | N 45°28’49.2”  E 101°11’55.0” | C1 |
| Fig. 11f | Hotuliin Teeg | HTSE | HTSE-013 | N 45°28’49.9”  E 101°11’57.2” | C1 |
| Fig. 11e | Hotuliin Teeg | HTS | HTS-056/1+2 | N 45°28’53.2”  E 101°11’34.9” | C1-D |
| Fig. 11e | Hotuliin Teeg | HTS | HTS-056/3 | N 45°28’54.5”  E 101°11’36.3” | C1-D |
| Fig. 11a  Fig. 12a-d | Unkheltseg | UNCH-A | UNCH-A/3B+4B | N 45°27’40.1”  E 101°12’04.4” | B |
| Fig. 11a  Fig. 12a-d | Unkheltseg | UNCH-A | UNCH-A/3+4 | N 45°27’40.1”  E 101°12’04.4” | D |
| Fig. 13 | Taatsiin Gol (south) | TGR-C | TGR-C/1 | N 45°23’10.9”  E 101°14’34.9” | C |
| Fig. 13 | Taatsiin Gol (south) | TGR-C‘ | TGR-C‘/1 | N 45°23’12.3”  E 101°14’35.4” | C |
|  | Taatsiin Gol (right) | TGR-ZO | TGR-ZO/1+2 | N 45°24’13.5”  E 101°15’53.0” | ?B |
| Fig. 14-15 | Taatsiin Gol (right) | TGR-B‘ | TGR-B/1 | N 45°24’47.3”  E 101°15’23.2” | B |
| Fig. 14 | Taatsiin Gol (right) | TGR-AB | TGR-AB (basis) | N 45°25’08.8”  E 101°15’39.2” | Eocene |
| Fig. 14-15 | Taatsiin Gol (right) | TGR-AB | TGR-AB/21 | N 45°24’41.1”  E 101°15’24.7” | B |
| Fig. 14 | Taatsiin Gol (right) | TGR-AB | TGR-AB/22 |  | B |
| Fig. 14 | Taatsiin Gol (right) | TGR-A | TGR-A/13+14 | N 45°25’12.5”  E 101°15’44.3” | A |
| Fig. 16 | Taatsiin Gol (left) | TGL-A | TGL-A/1+2 | N 45°26’57.4”  E 101°16’20.9” | A |
| Fig. 17 | Taatsiin Gol (left) | TGL-A‘ | TGL-A/11 |  | B |
| Fig. 18+19 | Unzing Churum | TAR-A | TAR-A/2 | N 45°31’14.4”  E 101°18’19.2” | C |
| Fig. 20 | Del | DEL-B | DEL-B/7+8 | N 45°27’10.2”  E 101°22’22.3” | B |
| Fig. 20 | Del | DEL-B | DEL-B/12 |  | C1 |
| Fig. 21-22 | Tatal Gol |  |  |  |  |
| Fig. 22a | Tatal Gol | TAT | TAT-051/2 | N 45°18’08.2”  E 101°37’09.3” | C1 |
| Fig. 22a | Tatal Gol | TAT | TAT-051/1 |  | C1 |
| Fig. 22a | Tatal Gol | TAT | TAT-054 | N 45°18’07.6”  E 101°37’09.7” | B |
| Fig. 22a | Tatal Gol | TAT | TAT-052/2 | N 45°18’09.4”  E 101°37’14.5” | C1-D |
| Fig. 22a | Tatal Gol | TAT | TAT-052/1 |  | C1 |
| Fig. 22a | Tatal Gol | TAT-E | TAT-E/32 | N 45°18’12.6”  E 101°37’15.7” | C1-D |
| Fig. 21a | Tatal Gol | TAT-E | TAT-E/27 |  | C1 |
| Fig. 21a | Tatal Gol | TAT-E | TAT-E/22 |  | C1 |
| Fig. 22b | Tatal Gol | TAT-E | TAT-044 | N 45°18’00.5”  E 101°37’20.6” | C1 |
| Fig. 22b | Tatal Gol | TAT-E | TAT-043 | N 45°17’59.8”  E 101°37’17.1” | C1 |
| Fig. 22b | Tatal Gol | TAT-E | TAT-055 | N 45°17’59.0”  E 101°37’16.6” | C |
| Fig. 22b | Tatal Gol | TAT-E | TAT-E/3 | N 45°14’58.2”  E 101°37’16.6” | B |
|  | Tatal Gol | TAT | TAT-038 | N 45°17’56.0”  E 101°37’10.9” | B |
|  | Tatal Gol | TAT | TAT-037 | N 45°17’54.1”  E 101°37’11.7” | A |
| Fig. 22c | Tatal Gol | TAT-D | TAT-D/1 | N 45°17’52.2”  E 101°37’18.5” | A |
| Fig. 23 | Tatal Gol | TAT-C | TAT-C/1-3 | N 45°18’19.5”  E 101°38’00.0” | A |
| Fig. 23 | Tatal Gol | TAT-C | TAT-C/6-7 |  | B |
| Fig. 24 | Hsanda Gol | SHG-C | SHG-C/1-2 | N 45°15’49.9”  E 101°43’04.9” | A |
| Fig. 25 | Hsanda Gol | SHG-A | SHG-A/6-20 |  | B |
| Fig. 25 | Hsanda Gol | SHG-AB | SHG-AB/15-20 |  | B |
| Fig. 25 | Hsanda Gol | SHG-AB | SHG-top |  | C1 |
| Fig. 24 | Hsanda Gol | SHG-D | SHG-D/12 | N 45°16’11.8”  E 101°45’55.9” | sandstone |
| Fig. 25 | Hsanda Gol | SHG-D | SHG-D/12-26 |  | B |
|  | Loh | LOH-C | LOH-C/1 |  | C1 |
| Fig. 26 | Loh | LOH-B | LOH-B/3 | N 45°17’04.9”  E 101°47’22.7” | C1 |
| Fig. 27 | Talyn Churum | GRAB | GRAB-II | N 45°16’50.4”  E 101°57’28.4” | A |
| Fig. 28 | Ikh Argalatyn Nuruu | IKH-A | IKH-A/1  IKH-A/2-4 | N 45°17’48.4”  E 102°04’57.2” | B |
| Fig. 29 | Ikh Argalatyn Nuruu | IKH-A | IKH-A/5 | N 45°17’49.1”  E 102°05’00.7” | C1 |
| Fig. 28 | Ikh Argalatyn Nuruu | IKH-B | IKH-B/2 | N 45°17’32.6”  E 102°05’34.2” | B |
| Fig. 28 | Ikh Argalatyn Nuruu | IKH-B | IKH-B/5 |  | C1 |

Supplementary data:

Overview of the investigated (figured) sections/fossil sites, fossil samples, the respective codes, coordinates and letter zones are given.
